# Global and regional progress towards reduced burden of oral conditions between 2019 and 2023: a systematic analysis for the Global Burden of Disease Study 2023

**DOI:** 10.1016/S2468-2667(26)00143-X

**Published:** 2026-08-18

**Authors:** GBD 2023 Oral Disorders Collaborators, Eduardo Bernabe, Wagner Marcenes, Abdullah, Hazim S Ababneh, Belayneh Jejaw Abate, Hedayat Abbastabar, Wael M Abdel-Rahman, Sherief Abd-Elsalam, Hassan A. Abdou, Meriem Abdoun, Rizwan Suliankatchi Abdulkader, Syed Hani Abidi, Richard Gyan Aboagye, Lucas Guimarães Abreu, Sawsan Abuhammad, Anirudh Balakrishna Acharya, Swetha Acharya, Lawan Hassan Adamu, Tajudeen Adesanmi Adebisi, Babatope Oluwadamilare Adebiyi, Kamoru Ademola Adedokun, Aanuoluwapo Adeyimika Afolabi, Muhammad Sohail Afzal, Percival Delali Agordoh, Salome Wumpini Agordoh, Trushdeep Agrawal, Piyush Agrawal, Tauseef Ahmad, Sajjad Ahmad, Aqeel Ahmad, Suhaib Ahmad, Khabir Ahmad, Ali Ahmadi, Naveed Ahmed, Shakeel Ahmed, Ali Ahmed, Ayman Ahmed, Kenni Wojujutari Ajele, Kasuni Hm Akalanka, Sara Akeel, Damilare Michael Akintunde, Hammad Akram, Chisom Joyqueenet Akunna, Timothy Olukunle Aladelusi, Yazan Al-Ajlouni, Mohammed Sultan Al-Ak’hali, Mohammad Khursheed Alam, Mostafa Alam, Ruaa A Alamoudi, Amer Hashim Al Ani, Salah Al Awaidy, Mohammed Albashtawy, Mohammed S Aldossary, Abdelazeem M Algammal, Saeed M. M Alghamdi, Mohammed Ahmed Alghauli, Amidu Alhassan, Ashraf Alhumaidi, Taha Hussein Hussein Ali, Kamran Ali, Syed Shujait Ali, Montaha Al-Iede, Morteza Alipour, Behrouz Alizadeh Savareh, Sabah Al-Marwani, Sadeq A Al-Maweri, Essam Ahmed Al-Moraissi, Mohmmad Minwer Alnaeem, Khaldoon Aied Alnawafleh, Margret Beaula Alocious Sukumar, Nivaldo Alonso, Mohammad Alqudah, Mohammad R Alqudimat, Ahmed Yaseen Alqutaibi, Mustafa Haleem Alsaadi, Mohammed Amjed Alsaegh, Anas Alsalhani, Ali Salman Al-Shami, Zaid Altaany, Awais Altaf, Hany Aly, Karem H Alzoubi, Nada A Alzunaidy, Uchenna Anderson Amaechi, Mcking Izeiza Amedari, Priscilla O Ameh, Junaid Amin, Saeed Amini, Mohammad Hosein Amirzade-Iranaq, Hubert Amu, Abhishek Anil, A.h. Sruthi Anil Kumar, Kabilan Annadurai, Saeid Anvari, Anayochukwu Edward Anyasodor, Jalal Arabloo, Mosab Arafat, Gururaj Arakeri, Aleksandr Y Aravkin, Demelash Areda, Jorge Arias De La Torre, Mohammad Saquib Ashraf, Javed Ashraf, Tahira Ashraf, Esma Asil, Saira Atif, Samuel Babajide Atoyebi, Nyan Min Aung, Manal Awad, Usman Ayub Awan, Sana Javaid Awan, Ashish Awasthi, Aneesa Ayoob, Somayyeh Azimi, Domenico Azzolino, Muhammad Badar, Huma Bader Ul Ain, Ashish D Badiye, Riadh Badraoui, Khlood K Baghlaf, Dania Bahdila, Mohammad Hassan Baig, Atif Amin Baig, Mohamad Amin Bakhshali, Marina Balabekova, Raju Balaji, Wondu Feyisa Balcha, Abdulrahman A. A Balhaddad, Yatan Pal Singh Balhara, Maciej Banach, Morteza Banakar, Salam Bani Hani, Palash Chandra Banik, Farhat Bano, Md. Abul Barkat, Rocío Barrios-Rodríguez, Amadou Barrow, Shahid Bashir, Azadeh Bashiri, Mohammad-Mahdi Bastan, Hagar Abd El Naby Bastawy, Kavita Batra, Oyundari Batsaikhan, Babak Behnam, Almaz Nibret Belay, Enku Shiferaw Belayneh, Nahom Girma Belete, Uzma Iqbal Belgaumi, Ibrahim Olajide Bello, Ghaith Benhsen, Alemshet Yirga Berhie, Ghalia Y Bhadila, Pooja Bhadoria, Pankaj Bhardwaj, Sonu Bhaskar, Ajay Nagesh Bhat, Ashok Kumar Bhati, Nada Binmadi, Tone Bjørge, Trupti Bodhare, Srinivasa Rao Bolla, Souad Bouaoud, Abdelhakim Bouyahya, Christopher Boxe, Maria Laura Braccini Fagundes, Dejana Braithwaite, Nikolay Ivanovich Briko, Michael Eric Bryan, Lin-Lin Bu, Sayed Muhammad Ata Ullah Shah Bukhari, Yu Cao, Giulia Carreras, Andrea Carugno, Sandra Carvalho, Felix Carvalho, Pamela Roxana Chacón-Uscamaita, Sandip Chakraborty, Vijay Kumar Chattu, Anis Ahmad Chaudhary, Sirshendu Chaudhuri, Akhilanand Chaurasia, Junhao Chen, Meng Xuan Chen, Mirza Farhana Iqbal Chowdhury, Dinh-Toi Chu, Sanjay C J, Natalia Cruz-Martins, Ferran Cuenca-Martínez, Omid Dadras, Namrata Dagli, Lina Dahabiyeh, Neha Dahiya, Mouni Damineni, Samuel Demissie Darcho, Riya Dave, Mauro Henrique Nogueira Guimarães De Abreu, Emina Dervišević, Premnath Dhanaraj, Amol S Dhane, Antonio Di Guardo, Elangovan Dilipan, Sadia Din, Orlando Luiz Do Amaral Júnior, Sushil Dohare, Cátia Domingues, Francesco Dondi, Lingkang Dong, Ojas Prakashbhai Doshi, Taiwo Omotayo Dosumu, Jiang Du, Mi Du, Vikash Kumar Dubey, Manisha Dubey, Siddhartha Dutta, Arkadiusz Marian Dziedzic, Venkata Ravikanth Eddula, Aziz Eftekharimehrabad, Ebrahim Eini, Michael Ekholuenetale, Seraphine Mojoko Eko, Abdelbaset Mohamed Elasbali, Amine Elbouzidi, Mohamed Elhoumed, Omar Abdelsadek Abdou Elmeligy, Ashraf A El-Metwally, Rami Elmorsi, Abdelfatteh EL Omri, Iman El Sayed, Nadia Bassuoni Elsharkawy, Abdelgawad Salah Eltahawy, Maha El Tantawi, Heba Emad, Purushoth Ethiraj, Devaraj Ezhilarasan, Amanda Ugochi Ezirim, Adeniyi Francis Fagbamigbe, Ayesha Fahim, Nour Fakih, Ebrahim Farashi, Muhammad Amber Fareed, Mohsen A Farghaly, Anees Ahmed Farooq Mohammed, Fatemeh Farshad, Alireza Feizkhah, Guangshou Feng, Maria Pia Ferraz, Nuno Ferreira, Getahun Fetensa, Tommaso Filippini, Florian Fischer, James L Fisher, Carlos Forner Álvarez, Delfin Lovelina Francis, Takeshi Fukumoto, Peter Andras Gaal, Amol Ramchandra Gadbail, Piyada Gaewkhiew, Márió Gajdács, Silvano Gallus, Fernando Jr. Barroga Garcia, Tsion S Gebre, Nsikakabasi Samuel George, Ahmad Ghashghaee, Sama Ghoba, Nasim Gholizadeh, Mahsa Ghorbani, Elena Ghotbi, Thinh Gia Gia Nguyen, Paramjit Singh Gill, Anil Kumar Goel, Marília Goettems, Awais Gohar, Gamze Gökulu, Pouya Goleij, Mohsen Golkar, Shailesh Madhukar Gondivkar, Divya Gopinath, Giuseppe Gorini, Barbara Niegia Garcia Goulart, Changhao Gu, Giovanni Guarducci, Bhuvaneshwari Gunasekar, Sunanda Gupta, Swati Gupta, Bhawna Gupta, Sapna Gupta, Bruno Gutiérrez, Heliasadat Haeriboroojeni, Khalid Rehman Hakeem, Esam Halboub, Pritam Halder, Ibrahim Ismael Hamarash, Raz Mohammed Hama Salih, Mohamed Hamed, Samer Hammoudeh, Abdelrahman M Hamouda, Mohd Anul Haq, Faizul Hasan, Shamimul Hasan, Towhid Hasan, Hamidreza Hasani, Nada Tawfig Hashim, Ahmad Shoeb Hashmi, Md Saquib Hasnain, Simon I Hay, Fei He, Mohammad Heidari, Golnaz Heidari, Narmin Mohammedsaeed Helal, Brenda Yuliana Herrera-Serna, Kamran Heydaryan, Kamal Hezam, Nguyen Quoc Hoan, Matthew Hobbs, Ramesh Holla, Syed Billal Hossain, Mehdi Hosseinzadeh, Sorin Hostiuc, Weijun Huang, Fernando N Hugo, Bing-Fang Hwang, Liliya Ibragimova, Khalid S Ibrahim, Jibran Ikram, Olayinka Stephen Ilesanmi, Irena M Ilic, Milena D Ilic, Danish Iqbal, Bilal Irfan, Md Rabiul Islam, Gaetano Isola, Hiroshi Ito, Chidozie Declan Iwu, Haitham Jahrami, Ayushi Jain, Ammar Abdulrahman Jairoun, Mihajlo Jakovljevic, Sahbanathul Missiriya Jalal, Reza Jalilzadeh Yengejeh, Rajiv Janardhanan, Sathish Kumar Jayapal, Ruwan Duminda Jayasinghe, Wonjeong Jeong, Nathan T Jibat, Nitin Joseph, Krupal Joshi, Farahnaz Joukar, Easter Joury, Jacek Jerzy Jozwiak, Hema Shree K, Thenmozhi K, Zubair Kabir, Vidya Kadashetti, Adel Kadri, Pradnya Vishal Kakodkar, Eswar Kandaswamy, Kehinde Kazeem Kanmodi, Kamala Kannan, Rami S Kantar, Neila Karagezova, Indira Karibayeva, Mohmed Isaqali Karobari, Tomasz M Karpiński, Gebrehiwot G Kassa, Birhanu Kassie Abeje, Joonas H Kauppila, Stella Adejoke Kayode, Awoke Amare Kebede, Yousef Saleh Khader, Himanshu Khajuria, Raees Khan, Zahid Khan, Hafiz Nadeem Khan, Muhammad Umer Khan, Muhammad Khan, Sameer Uttamaro Khasbage, Khalid A Kheirallah, Feriha Fatima Khidri, Yihyun Kim, Min Seo Kim, Sung Min Kim, Ruth W Kimokoti, Sanjay Kini B, Adnan Kisa, Tobias Kleinjung, Shivakumar KM, Prakash Babu Kodali, Farzad Kompani, Archana Koul, Kewal Krishan, Bindu Krishnan, Nitya Krishnasamy, Omar Kujan, Anit Kujur, Mukhtar Kulimbet, Emmanuel Kumah, Manoj Kumar, Jogender Kumar, Pradeep Kumar, Jeevan Kumar, Pawan Kumar, Sumeet Kumar, Ashok Kumar, Kamal Kumar, Neeraj Kumar, Dewesh Kumar, Sandeep Kumar, Jibin Kunjavara, Om P Kurmi, Pramod Kumar Kushawaha, Dian Kusuma, Timo Lajunen, Dharmesh Kumar Lal, Ratilal Lalloo, Tamás Lantos, Giusy Rita Maria La Rosa, Savita Lasrado, Seung Won Lee, Kakithakara Vajravelu Leela, Elvynna Leong, Mengyi LI, Zhengrui Li, Ming-Chieh Li, Ambreen Liaqat, Liu Liu, Erand Llanaj, Madeeha Shahzad Lodhi, Mariia Lopatkina, Anna Lövgren, Shanjie Luan, Alessandra Lugo, Ellina Lytvyak, Hawraz Ibrahim M. Amin, Siyao Ma, Kevin Sheng-Kai Ma, Monika Machoy-Rakoczy, Máté Mackei, Inamul Hasan Madar, Ahmed A Madfa, Mohammed Magdy Abd El Razek, Mari Kannan Maharajan, Nozad Hussein Mahmood, Trisha Mallick, Iqra Manzoor, Tahir Maqbool, Vijaya Marakala, Priyadharshini Marckasagayam, Stefano Masi, Manu Raj Mathur, Medha Mathur, Khurshid A Mattoo, Jacek Matys, Jitendra Kumar Meena, Riffat Mehboob, Vini Mehta, Dalia E Meisha, Satish Melwani, Ritesh G Menezes, Tomislav Mestrovic, Andrea Michelerio, Giuseppe Minervini, Tridip Mitra, Kanisha Mittal, Hashem Mohamadian, Nouh Saad Mohamed, Fatima Awad Elkarim Elfaki Mohamed, Yahaya Mohammed, Yugal Kishore Mohanta, Ali H Mokdad, Himel Mondal, Paula Moraga, Rafael Silveira Moreira, Mahdis Morovvati, Rohith Motappa, Amin Mousavi Khaneghah, Ahmed Msherghi, Rabia Mubarak, Sumaira Mubarik, Christopher J L Murray, Satta Muthu Murugan, David Musoke, Sherzad Ibrahim Mustafa, Saravanan Muthupandian, Kiran P P Nagdeo, Ganesh R Naik, Lekshmi M Nair, Prasad Nalabothu, Rizwan Naqvi, Shumaila Nargus, Gustavo G Nascimento, Mohammad Zakaria Nassani, Zuhair S Natto, Zakira Naureen, Kinjal Nayak, Biswa Prakash Nayak, Javad Nazari, Sudan Prasad Neupane, Sumaiya Nezam, Long Hoang Nguyen, Tham Thi Nguyen, Cuong Tat Nguyen, Duy Cao Nguyen, Vikram Niranjan, Lawrence Achilles Nnyanzi, Mohammed Noushad, Nazik Nurelhuda, Bogdan Oancea, Musa Kolapo Oladejo, Yinka Doris Oluwafemi, Esteban Ortiz-Prado, Mahesh P A, Alicia Padron-Monedero, Jagadish Rao Padubidri, Sujogya Kumar Panda, Keerthi Panneer Selvam, Georgios D Panos, Shahina Pardhan, Romil R Parikh, Sun Jae Park, Pragnesh Parmar, Sameena Parveen, Eleonora Pascucci, Mitesh Patel, Shivani Patel, Mehali Lalitbhai Patel, Jay Patel, Vipin Patidar, Shankargouda Patil, Shrikant Pawar, Olumuyiwa James Peter, Pawel Piotr Pietkiewicz, Thantrira Porntavetus, Amit Porwal, Priyanka Porwal, Sergio I Prada, Harsh Priya, Luís Proença, Pooja Puri, Bharathi M Purohit, Nameer Hashim Qasim, Asma Saleem Qazi, Xiang Qi, Cheng Quan, Nuzul Qur’aniati, Venkatraman Radhakrishnan, Raghu Anekal Radhakrishnan, Fakher Rahim, Mosiur Rahman, Hakim Rahmoune, Amina Suleiman Rajah, Rafał Rakoczy, Venkitachalam Ramanarayanan, Shakthi Kumaran Ramasamy, Ramasubburayan Ramasamy, Lokesh Rana, Shailendra Singh Rana, Kumuda Rao, Sowmya J Rao, Mithun Rao, Prateek Rastogi, Shree Rath, Devarajan Rathish, Tapash Ranjan Rautray, Sujith Sri Surya Ravi, Salman Rawaf, Muhammad Liaquat Raza, Elrashdy M Redwan, Abdul Rehman, Bhagwan Narayan Rekadwad, Nazila Rezaei, Abanoub Riad, Maria Jesus Rios-Blancas, Hermano Alexandre Lima Rocha, Chanapong Rojanaworarit, Mario Romandini, Rohini Ruhil, Khaled Saad, Korosh Saber, Fatemeh Sadeghi-Ghyassi, Mohammad Reza Saeb, Umar Saeed, Amirhossein Sahebkar, Fatemeh Saheb Sharif-Askari, Vaibhav Sahni, Durgesh Prasad Sahoo, Ambika Sahoo, Alaka Sahoo, Ankita Saikia, Joseph W Sakshaug, Luciane B Salaroli, Samreen Saleem, Firdaus Samad, Fasineh Shekuba Samura, Abdallah M Samy, Sathish Sankar, Andari Sarasati, Shilpa Sarawagi, Sachin C Sarode, Gargi Sachin Sarode, Michele Sassano, Aanchal Satija, Maheswar Satpathy, Jennifer Saulam, Amar Saurabh, Benedikt Michael Schaarschmidt, Falk Schwendicke, Amin Sedigh, Siddharthan Selvaraj, Subramanian Senthilkumaran, Mostafa Shaban, Arsalan Shahri, Hamid R Shahsavari, Muhammad Shahzad, Masood Ali Shaikh, Muhammad Aaqib Shamim, Alfiya Shamsutdinova, Mohammed Shannawaz, Nadim Sharif, Amin Sharifan, Kamlesh Sharma, Bereket Beyene Shashamo, Mahabalesh Shetty, Premalatha K Shetty, Nzooma Munkwangu Shimaponda-Mataa, Shirindokht Shirazi, Km Shivangi, Zahra Shokati Eshkiki, Deborah Oluwaseun Shomuyiwa, Sinegugu Nosipho Shongwe, Seyed Afshin Shorofi, Sunil Shrestha, Suleiman Adeiza Shuaibu, Gustavo Correia Basto Da Silva, Luís Manuel Lopes Rodrigues Silva, Jasvinder A Singh, Harpreet Singh, Surjit Singh, Samer Singh, Prince Kumar Singh, Siddharth Singh, Ragini D Singh, Amit Singh, Abhinav Singh, Richa Singhania, Gisha Sivan, Gowri Sivaramakrishnan, Chandan S N, Farrukh Sobia, Ranjan Solanki, Monica Charlotte Charlotte Solomon, Saraswathy Sreeram, Kannan Sridharan, Quirin D Strotzer, Mahwish Suhaib, Marjia Sultana, Jing Sun, Seyyed Mohammad Tabatabaei, Biniyam Beyene Tabor, Santosh Kumar Tadakamadla, Jyothi Tadakamadla, Amir Tahavvori, Jabeen Taiba, Iman M Talaat, Mircea Tampa, Benjamin Kye Jyn Tan, Shynar Tanabayeva, Abdelghani Tbakhi, Reem Temsah, Abdulkarim Temsah, Dufera Rikitu Terefa, Tigist Derebe Tesfahun, Azimeraw Arega Tesfu, Ismaeel Tharwat, Manuel Sebastian Thomas, Amir Tiyuri, Madi Tleshev, Razie Toghroli, Arole Darwin Touko, Marcos Roberto Tovani-Palone, Thang Huu Tran, Indang Trihandini, Mike Tuffour Amirikah, Biruk Shalmeno Tusa, Saeed Ullah, Srikanth Umakanthan, Bhaskaran Unnikrishnan, Amedin Aliyi Usso, Olivier Uwishema, Hande Uzunçıbuk, Orsolya Varga, Siddhartha Alluri Varma, Vishnu Priya Veeraraghavan, Prabhakar Veginadu, U Venkatesh, Nguyen-Kieu Viet-Nhi, Vignesh Vikram, Victor E Villalobos-Daniel, Ave Voit, Simona Ruxandra Volovat, Rashim Wadhwa, Liang Wang, Wanzhou Wang, Zhikang Wang, Tati Suryati Warouw, Chenxuan Wei, Desalegn Desalegn Weldebrhan, Nuwan Darshana Wickramasinghe, Andrew Awuah Wireko, Dawit Habte Woldeyes, Shulan Wu, Patlolla Sriram Yadav, Saba Yahoo, Natsuhiko Yamada, Kivanc Yangi, Thomas Kidanemariam Yewodiaw, Siyan Yi, Dong Keon Yon, Monal Yuwanati, Vesna Zadnik, Mubashir Zafar, Muhammad Sohail Zafar, Iman Zare, Mohammed G M Zeariya, Zhi-Jiang Zhang, Jianhui Zhao, Lei Zheng, Hafsa Zia, Zahraa Zibara, Magdalena Zielińska, Shaher H Zyoud, Kanyin Liane Ong

**Affiliations:** A Institute of Dentistry, Queen Mary University of London, London, UK; C Medicine Department, Rawalpindi Medical University, Rawalpindi, Pakistan; D Department of Radiation Oncology, Massachusetts General Hospital, Boston, MA, USA; E University of Gondar Comprehensive Specialized Hospital, University of Gondar, Gondar, Ethiopia; F Advanced Diagnostic and Interventional Radiology Research Center (ADIR), Tehran University of Medical Sciences, Tehran, Iran; G Department of Medical Laboratory Science, University of Sharjah, Sharjah, United Arab Emirates; H Department of Tropical Medicine and Infectious Diseases, Tanta University, Egypt, Tanta, Egypt; I Faculty of Medicine, Capital (Helwan) University, Cairo, Egypt; J Department of Medicine, University of Setif Algeria, Sétif, Algeria; K Department of Health, University of Setif Algeria, Sétif, Algeria; L National Institute of Epidemiology, Indian Council of Medical Research, Chennai, India; M Department of Biomedical Sciences, Nazarbayev University School of Medicine, Astana, Kazakhstan; N Department of Family and Community Health, University of Health and Allied Sciences, Ho, Ghana; O School of Population Health, University of New South Wales, Sydney, NSW, Australia; P Department of Pediatric Dentistry, Federal University of Minas Gerais, Belo Horizonte, Brazil; Q Department of Nursing, University of Sharjah, Sharjah, United Arab Emirates; R Department of Maternal and Child Health Nursing, Jordan University of Science and Technology, Irbid, Jordan; S Department of Restorative Dentistry, University of Sharjah, Sharjah, United Arab Emirates; T Department of Oral Pathology and Microbiology, JSS Academy of Higher Education and Research, Mysuru, India; U Department of Human Anatomy, Federal University Dutse, Dutse, Nigeria; V Department of Anatomy, Bayero University Kano, Kano, Nigeria; W Department of Microbiology, Ladoke Akintola University, Osogbo, Nigeria; X Department of NMC Healthcare, Independent Consultant, Sharjah, United Arab Emirates; Y Department of Pediatrics, University of Calgary, Calgary, AB, Canada; Z School of Public Health, University of the Western Cape, Bellville, South Africa; AA Department of Immunology, Roswell Park Comprehensive Cancer Center, Buffalo, NY, USA; AB Graduate Program Division, University at Buffalo, Buffalo, NY, USA; AC Technical Services Directorate, MSI Nigeria Reproductive Choices, Abuja, Nigeria; AD Department of Life Sciences, University of Management and Technology, Lahore, Pakistan; AE Department of Nutrition and Dietetics, University of Health and Allied Sciences, Ho, Ghana; AF Operations Department, World Vision, Kete Krachi, Ghana; AG Sujan Surgical Cancer Hospital & Amravati Cancer Foundation, Amravati, India; AH Division of Medical Research, SRM Institute of Science and Technology, Kattankulathur, India; AI School of Public Health, Zhejiang University, Hangzhou, China; AJ Department of Community Health Sciences, Sohail University, Karachi, Pakistan; AK Department of Health and Biological Sciences, Abasyn University, Peshawar, Pakistan; AL Department of Natural Sciences, Lebanese American University, Beirut, Lebanon; AM College of Medicine, Shaqra University, Shaqra, Saudi Arabia; AN Department of General Surgery, School of Surgery Wales, Swansea, UK; AO King Khaled Eye Specialist Hospital and Research Center, Riyadh, Saudi Arabia; AP Department of Epidemiology and Biostatistics, Shahrekord University of Medical Sciences, Shahrekord, Iran; AQ Department of Epidemiology, Shahid Beheshti University of Medical Sciences, Tehran, Iran; AR Department of Assistance Medical Sciences, University of Tabuk, Tabuk, Saudi Arabia; AS Department of Computer Science, Jazan University, Jazan, Saudi Arabia; AT Department of Pharmacy Practice, Riphah Institute of Pharmaceutical Sciences, Islamabad, Pakistan; AU Division of Infectious Diseases and Global Public Health (idgph), University of California San Diego, San Diego, CA, USA; AV Institute of Endemic Diseases, University of Khartoum, Khartoum, Sudan; AW Pan-African One Health Institute (PAOHI), Kigali, Rwanda; AX Department of Social Sciences, North-West University, South Africa, Mafikeng, South Africa; AY Rural Health Research Institute, Charles Sturt University, Orange, NSW, Australia; AZ Faculty of Dentistry, Oral Diagnostic Sciences, King Abdulaziz University, Jeddah, Saudi Arabia; BA Medecins Sans Frontieres - Holland (Emergency Desk), Doctors Without Borders, Omdurman, Sudan; BB Department of Community Medicine, Ahmadu Bello University, Zaria, Nigeria; BC Department of Infection Prevention & Control, Baylor Scott & White Health, Frisco, TX, USA; BD VentureBlick; BE Department of Public Health, The Intercountry Centre for Oral Health (ICOH) for Africa, Jos, Nigeria; BF Department of Public Health, Federal Ministry of Health, Garki, Nigeria; BG Department of Oral and Maxillofacial Surgery, University of Ibadan, Ibadan, Nigeria; BH Department of Oral and Maxillofacial Surgery, University College Hospital, Ibadan, Ibadan, Nigeria; BI Department of Rehabilitation, Montefiore Medical Center, Bronx, NY, USA; BJ Department of Epidemiology, Columbia University, New York, NY, USA; BK Department of Preventive Dental Sciences, Jazan University, Jazan, Saudi Arabia; BL Preventive Dentistry Department, Jouf University, Sakaka, Saudi Arabia; BM Department of Oral and Maxillofacial Surgery, Shahid Beheshti University of Medical Sciences, Tehran, Iran; BN Faculty of Dentistry- Endodontic Department, King Abdulaziz University, Jeddah, Saudi Arabia; BO Clinical Sciences Department, Ajman University, Ajman, United Arab Emirates; BP Department of Communicable Diseases, Ministry of Health, Muscat, Oman; BQ Middle East, Eurasia, and Africa Influenza Stakeholders Network, Muscat, Oman; BR Department of Community and Mental Health, Al al-Bayt University, Mafraq, Jordan; BS General Directorate of Research and Studies, Ministry of Health, Riyadh, Saudi Arabia; BT Department of Bacteriology, Immunology, and Mycology, Suez Canal University, Ismailia, Egypt; BU Clinical Technology Department, Faculty of Applied Medical Sciences, Mecca, Saudi Arabia; BV Faculty of Dentistry, Ibb University, Ibb, Yemen; BW Department of Adult Health, University of Cape Coast, Cape Coast, Ghana; BX Faculty of Dentistry, Ibn Al-Nafis University for Medical Sciences, Sana’A, Yemen; BY Department of Oral and Maxillofacial Surgery, Xi’an Jiaotong University, Xi’An, China; BZ Department of Statistics and Informative, Salahaddin University-Erbil, Erbil, Iraq; CA College of Dental Medicine, Qatar University, Doha, Qatar; CB Center for Biotechnology and Microbiology, University of Swat, Swat, Pakistan; CC University of Tabuk, Tabuk, Saudi Arabia; CD The School of Medicine, The University of Jordan, Amman, Jordan; CE Biomedical Physics Group, University of Hamburg, Hamburg, Germany; CF Department of Allied Medical Sciences, Shahid Beheshti University of Medical Sciences, Tehran, Iran; CG Independent Consultant, Amman, Jordan; CH Department of Oral and Maxillofacial Surgery, Thamar University, Thamar, Yemen; CI Department of Clinical Nursing, Al-Zaytoonah University of Jordan, Amman, Jordan; CJ Nursing Faculty, University of Tabuk, Tabuk, Saudi Arabia; CK SRM School of Public Health, Chennai, India; CL Department of Surgery, University of São Paulo, São Paulo, Brazil; CM Department of Pathology and Microbiology, Jordan University of Science and Technology, Irbid, Jordan; CN Department of CTAG, King Hussein Cancer Center, Amman, Jordan; CO American University of the Middle East, Egaila, Kuwait; CP Department of Prosthodontics and Implant Dentistry, Taibah University, Madinah, Saudi Arabia; CQ Department of Prosthodontics and Implant Dentistry, Ibb University, Ibb, Yemen; CR College of Medicine, Jaber Ibn Hayyan University for Medical and Pharmaceutical Sciences, Kufa, Iraq; CS College of Dental Medicine, University of Sharjah, Sharjah, United Arab Emirates; CT Research Institute for Medical and Health Sciences, University of Sharjah, Sharjah, United Arab Emirates; CU Department of Dentistry, Vision Colleges, Riyadh, Saudi Arabia; CV Medical College, Hama University, Hama, Syria; CW Department of Pharmacology, Sana’a University, Sanaa, Yemen; CX Department of Basic Medical Sciences, Universiti Malaysia Sarawak, Kota Samarahan, Malaysia; CY Department of Basic Sciences, Faculty of Medicine, Irbid, Jordan; CZ Health Science Division, Higher Collage of Technology, Abu Dhabi, United Arab Emirates; DA Institute of Molecular Biology and Biotechnology (IMBB), The University of Lahore, Lahore, Pakistan; DB Faculty of Health Sciences, Equator University of Science and Technology, Uganda, Masaka, Uganda; DC Department of Pediatrics, Cleveland Clinic, Cleveland, OH, USA; DD Department of Pharmacy Practice and Pharmacotherapeutics, University of Sharjah, Sharjah, United Arab Emirates; DE Department of Food Science and Human Nutrition, College of Agriculture and Food, Qassim University, Buraydah, Saudi Arabia; DF Evaluation Unit, Global Alliance for Vaccines and Immunisations, Geneva, Switzerland; DG John D. Bower School of Population Health Science, University of Mississippi Medical Center, Jackson, MS, USA; DH Preventive and Community Dentistry, Obafemi Awolowo University, Ile-Ife, Nigeria; DI Department of Preventive Dentistry, University of Jos, Jos, Nigeria; DJ Department of Preventive Dentistry, Jos University Teaching Hospital, Jos, Nigeria; DK Department of Physiotherapy, University of Hail, Hail, Saudi Arabia; DL Clinical Sciences - Orthopaedic, University of Science Malaysia, Kota Baharu, Malaysia; DM Department of Health and Management Sciences, Khomein University of Medical Sciences, Khomein, Iran; DN Department of Oral Medicine, School of Dentistry, Isfahan University of Medical Sciences, Isfahan, Iran; DO Department of Population and Behavioural Sciences, University of Health and Allied Sciences, Ho, Ghana; DP Department of Pharmacology, All India Institute of Medical Sciences, Bhubaneswar, India; DQ Division of Medical Research, Sri Ramaswamy Memorial Institute of Science and Technology, Chengalpattu, India; DR Department of Public Health, The Apollo University, Chittoor, India; DS Regenerative Medicine, Organ Procurement and Transplantation Multi-Disciplinary Center, Guilan University of Medical Sciences, Rasht, Iran; DT Health Management and Economics Research Center, Iran University of Medical Sciences, Tehran, Iran; DU Al Ain University, Al Ain University, Abu Dhabi, United Arab Emirates; DV Department of Head and Neck Oncology, HCG Cancer Hospital, Bengaluru, India, Bengaluru, India; DW Department of Applied Mathematics, University of Washington, Seattle, WA, USA; DX Institute for Health Metrics and Evaluation, University of Washington, Seattle, WA, USA; DY Department of Health Metrics Sciences, School of Medicine, University of Washington, Seattle, WA, USA; DZ College of Art and Science, Ottawa University, Surprise, AZ, USA; EA Care in Long Term Conditions Research Division, King’s College London, London, UK; EB Department of Grupo 15, CIBER Epidemiology and Public Health (CIBERESP), Madrid, Spain; EC Department of Medical Laboratory Sciences, Riyadh ELM University, Riyadh, Saudi Arabia; ED Department of Oral Public Health, Health Services Academy, Islamabad, Pakistan; EE Public Health Department, Health Services Academy, Islamabad, Pakistan; EF Department of Biostatistics, Sakarya University, Sakarya, Türkiye; EG Department of Nutrition and Dietetics, Ankara University, Ankara, Türkiye; EH CMH Lahore Medical College & Institute of Dentistry, National University of Medical Sciences (NUMS), Lahore, Pakistan; EI School of Statistics and Actuarial Science, University of the Witwatersrand, Johannesburg, South Africa; EJ Department of Oral Biological Science, University of Dental Medicine, Mandalay, Myanmar; EK Dental Unit, U Hla Tun’s Hospice Cancer Foundation, Mandalay, Myanmar; EL Department of Dental Medicine, University of Sharjah, Sharjah, United Arab Emirates; EM Department of Medical Laboratory Technology, The University of Haripur, Haripur, Pakistan; EN Department of Biostatistics, CSIR-Central Drug Research Institute, Lucknow, India; EO Department of Biostatistics, Academy of Scientific and Innovative Research (AcSIR), Ghaziabad, India; EP Department of Public Health Dentistry, Amrita Institute of Medical Sciences, Kochi, India; EQ International Research Collaborative- Health and Equity, The University of Western Australia, Perth, WA, Australia; ER Geriatric Unit, Fondazione IRCCS Ca’ Granda Ospedale Maggiore Policlinico, Milan, Italy; ES Directorate of Quality Assurance, Gomal University, Dera Ismail Khan, Pakistan; ET Department of Food & Health, The University of Lahore, Lahore, Pakistan; EU Department of Forensic Science, Government Institute of Forensic Science Nagpur, Nagpur, India; EV Rashtrasant Tukadoji Maharaj Nagpur University, Nagpur, India; EW Faculty of Medicine of Tunis, University Tunis El Manar, Tunis, Tunisia; EX Department of Biology, University of Hail, Hail, Saudi Arabia; EY Pediatric Dentistry Department, King Abdulaziz University, Jeddah, Saudi Arabia; EZ Department of Pediatric Dentistry, Faculty of Dentistry, King Abdulaziz University, Jeddah, Saudi Arabia; FA Department of Pharmacy, Yonsei University, Incheon, South Korea; FB Applied College, University of Tabuk, Tabuk, Saudi Arabia; FC Department of Medical Informatics, Mashhad University of Medical Sciences, Mashhad, Iran; FD Department of Pathological Physiology, Kazakh National Medical University, Almaty, Kazakhstan; FE Saveetha Medical College and Hospital, Saveetha University, Chennai, India; FF Department of Midwifery, Bahir Dar University, Bahir Dar, Ethiopia; FG Department of Restorative Dental Sciences, Imam Abdulrahman Bin Faisal University, Dammam, Saudi Arabia; FH National Drug Dependence Treatment Centre, All India Institute of Medical Sciences, New Delhi, India; FI Department of Hypertension, Medical University of Lodz, Lodz, Poland; FJ Polish Mothers’ Memorial Hospital Research Institute, Lodz, Poland; FK Dental Research Center, Tehran University of Medical Sciences, Tehran, Iran; FL Health Policy Research Center, Shiraz University of Medical Sciences, Shiraz, Iran; FM Irbid National University, Irbid National University, Jordan, Jordan; FN Department of Noncommunicable Diseases, Bangladesh University of Health Sciences, Dhaka, Bangladesh; FO Center for Higher Studies and Research, Bangladesh University of Professionals, Dhaka, Bangladesh; FP Biochemistry Department, University of Karachi, Karachi, Pakistan; FQ Department of Pharmaceutics, University of Hafr Al Batin, Hafr Al Batin, Saudi Arabia; FR Department of Medicine Preventive and Public Health, University of Granada, Granada, Spain; FS Department of Public and Environmental Health, University of The Gambia, Banjul, The Gambia; FT Department of Epidemiology, University of Florida, Gainesville, FL, USA; FU University Institute of Food Science and Technology, The University of Lahore, Lahore, Pakistan; FV Department of Health Information Management, Shiraz University of Medical Sciences, Shiraz, Iran; FW Non-Communicable Diseases Research Center, Tehran University of Medical Sciences, Tehran, Iran; FX School of Medicine, Iran University of Medical Sciences, Tehran, Iran; FY Department of Restorative Dentistry, University of Hail, Hail, Saudi Arabia; FZ Endodontic Department, Al-Azhar University, Cairo, Egypt; GA Department of Medical Education, University of Nevada Las Vegas, Las Vegas, NV, USA; GB Department of Public Health and Health Policy, Hiroshima University, Hiroshima, Japan; GC Scientific Research and Development, Avicenna Biotech Research, Clarksburg, MD, USA; GD Floret, Advanced Genomics & Bioinformatics Research Center, Lagos, Nigeria; GE College of Medicine and Health Science, Bahir Dar University, Bahir Dar, Ethiopia; GF Department of General Surgery, Plastic Surgery, St. Paul’s Hospital Millennium Medical College, Addis Ababa, Ethiopia; GG School of Public Health, Haramaya University, Dire Dawa, Ethiopia; GH Department of Oral Pathology and Microbiology, Krishna Vishwa Vidyapeeth (Deemed to be University), Karad, India; GI Department of Oral Medicine and Diagnostic Science, King Saud University, Riyadh, Saudi Arabia; GJ Department of Pathology, University of Helsinki, Helsinki, Finland; GK Independent Consultant, Barcelona, Spain; GL Department of Nursing, Bahir Dar University, Bahir Dar, Ethiopia; GM Department of Anatomy, All India Institute of Medical Sciences, Rishikesh, India; GN National Institute for Implementation Research on Non Communicable Diseases, Indian Council of Medical Research, Jodhpur, India; GO Global Health Neurology Lab, NSW Brain Clot Bank, Sydney, NSW, Australia; GP Division of Cerebrovascular Medicine and Neurology, National Cerebral and Cardiovascular Center, Suita, Japan; GQ Department of General Medicine, Manipal Academy of Higher Education, Mangalore, India; GR Department of Oral Diagnostic Sciences, King Abdulaziz University, Jeddah, Saudi Arabia; GS Department of Global Public Health and Primary Care, University of Bergen, Bergen, Norway; GT Cancer Registry of Norway, Oslo, Norway; GU Department of Community and Family Medicine, All India Institute of Medical Sciences, Ramanathapuram, India; GV Department of Biomedical Sciences, Nazarbayev University, Astana, Kazakhstan; GW Department of Medicine, University Ferhat Abbas of Setif, Setif, Algeria; GX Department of Epidemiology and Preventive Medicine, University Hospital Saadna Abdenour, Setif, Algeria; GY Laboratory of Human Pathology Biology, University Mohammed V, Rabat, Morocco; GZ Department of Earth, Environment, and Equity, Howard University, Washington, DC, USA; HA Department of Stomatology, Federal University of Santa Maria, Santa Maria, Brazil; HB Cancer Population Sciences Program, University of Florida Health Cancer Center, Gainesville, FL, USA; HC Department of Epidemiology and Evidence-Based Medicine, I.M. Sechenov First Moscow State Medical University, Moscow, Russia; HD Nuffield Department of Medicine, University of Oxford, Oxford, UK; HE Cancer Program, Broad Institute of MIT and Harvard, Cambridge, MA, USA; HF Department of Oral & Maxillofacial - Head Neck Oncology, Wuhan University, Wuhan, China; HG Department of Biological Sciences, National University of Medical Sciences (NUMS), Rawalpindi, Pakistan; HH Faculty of Dentistry, National University of Singapore, Singapore, Singapore; HI Institute for Cancer Research, Prevention and Clinical Network, Florence, Italy; HJ Department of Medicine and Surgery, University of Insubria, Varese, Italy; HK Psychological Research Center (cipsi), University of Minho, Braga, Portugal; HL Research Unit on Applied Molecular Biosciences (ucibio), University of Porto, Porto, Portugal; HM Posgrado de Medicina (postgraduate Program in Medicine), Universidad Cientifica del Sur, Lima, Peru; HN State Disease Investigation Laboratory, Animal Resources Development Department, Agartala, India; HO Department of Public Health and Health Sciences, Tennessee State University (TSU), Nashville, TN, USA; HP Department of Community Medicine, Datta Meghe Institute of Medical Sciences, Sawangi, India; HQ Department of Biology, Imam Mohammad Ibn Saud Islamic University (IMSIU), Riyadh, Saudi Arabia; HR Department of Public Health, Indian Institute of Public Health, Hyderabad, India; HS Department of Oral Medicine and Radiology, King George’s Medical University, Lucknow, India; HT Department of Urology, The Second Affiliated Hospital of Kunming Medical University, Kunming, China; HU School of Dentistry, University of Michigan, Ann Arbor, MI, USA; HV Department of Surgery, Johns Hopkins University, Baltimore, MD, USA; HW The Interdisciplinary Research Group on Biomedicine and Health, VNU International School (VNUIS), Hanoi, Viet Nam; HX Faculty of Applied Sciences, VNU International School (VNUIS), Hanoi, Viet Nam; HY JSS Dental College & Hospital, Jagadguru Sri Shivarathreeswara Academy of Health Education and Research, Mysore, India; HZ Life and Health Sciences Research Institute (ICVS), University of Minho, Braga, Portugal; IA Institute for Research and Innovation in Health (i3s), University of Porto, Porto, Portugal; IB Faculty of Physical Therapy, University of Valencia, Valencia, Spain; IC Center for Immigrant and Refugee Health, Public Health Institute, Los Angeles, CA, USA; ID Karnavati Scientific Research Centre, Karnavati Scientific Research Centre, Gandhinagar, India; IE School of Pharmacy, The University of Jordan, Amman, Jordan; IF Division of Delivery Research/implementation Research, Indian Council of Medical Research, Delhi, India; IG Department of Dentistry, Four States Dental Care, Joplin, MO, USA; IH Department of Public Health, Haramaya University, Harar, Ethiopia; II Amedabad Dental College and Hospital, Gujarat University, Ahmedabad, India; IJ Department of Community and Preventive Dentistry, Federal University of Minas Gerais, Belo Horizonte, Brazil; IK Department of Health Policy & Health Services Research, Boston University, Boston, MA, USA; IL Department of Forensic Medicine, University of Sarajevo, Sarajevo, Bosnia and Herzegovina; IM Department of Biotechnology, Karunya Institute of Technology and Sciences in INDIA, Coimbatore, India; IN Research and Development Cell, Dr. D. Y. Patil Vidyapeeth, Pune (Deemed to be University), Pune, India; IO Unit of Dermatology, La Sapienza University, Rome, Italy; IP Department of IDI, IRCCS, Rome, Italy; IQ Department of Physiology, Saveetha University, Chennai, India; IR Department of Computer Engineering, Gachon University, Seongnam-Si, South Korea; IS Department of Physiology, Chulalongkorn University, Bangkok, Thailand; IT Department of Public Health, Federal University of Santa Maria, Santa Maria, Brazil; IU Department of Public Health, Jazan University, Jazan, Saudi Arabia; IV Laboratory of Drug Development and Technologies, University of Coimbra, Coimbra, Portugal; IW Department of Nuclear Medicine, ASST Spedali Civili di Brescia and Università degli Studi di Brescia, Brescia, Italy; IX Shanghai Sixth People’s Hospital Affiliated To Shanghai Jiao Tong University School of Medicine, Shanghai Jiao Tong University, Shanghai, China; IY Independent Consultant, Bridgewater, NJ, USA; IZ Department of Maternal and Child Health, Bowen University Iwo, Iwo, Nigeria; JA Department of Pathology, China Medical University, Shenyang, China; JB School of Stomatology, Shandong University, Jinan, China; JC Department of Periodontology, Shandong University, Jinan, China; JD Indian Institute of Technology (BHU), Varanasi, Indian Institute of Technology (BHU), Varanasi, India; JE Department of Hepatology, Sanjay Gandhi Postgraduate Institute of Medical Sciences, Lucknow, India; JF Department of Pharmacology, All India Institute of Medical Sciences, Rajkot, India; JG Department of Conservative Dentistry With Endodontics, Medical University of Silesia, Katowice, Poland; JH Department of Dermatology, Venereology and Leprology, Apollo Institute of Medical Sciences and Research Chittoor, Chittoor, India; JI Department of Biochemistry, Ege University, Izmir, Türkiye; JJ Nanobiomedicine International Research Center, Azerbaijan Medical University, Baku, Azerbaijan; JK Independent Consultant, Ahvaz, Iran; JL Faculty of Science and Health, University of Portsmouth, Hampshire, UK; JM Department of Biomedical Sciences, University of Buea, Buea, Cameroon; JN Department of Clinical Laboratory Sciences, Jouf University, Al-Qurayyat, Saudi Arabia; JO Faculty of Sciences, University Mohammed the First, Oujda, Morocco; JP National Institute of Public Health Research, Ministry of Health, Nouakchott, Mauritania; JQ Pediatric Dentistry and Dental Public Health Department, Alexandria University, Alexandria, Egypt; JR College of Public Health and Health Informatics, King Saud bin Abdulaziz University for Health Sciences, Riyadh, Saudi Arabia; JS King Abdullah International Medical Research Center, Riyadh, Saudi Arabia; JT MD Anderson Cancer Center Department of Plastic Surgery, University of Texas, Houston, TX, USA; JU Surgical Research Section, Hamad Medical Corporation, Doha, Qatar; JV Biomedical Informatics and Medical Statistics Department, Alexandria University, Alexandria, Egypt; JW Department of Maternal and Child Health Nursing, Jouf University, Sakaka, Saudi Arabia; JX Faculty of Veterinary Medicine, Damanhour University, Damanhur, Egypt; JY Department of Pediatric Dentistry and Dental Public Health, Alexandria University, Alexandria, Egypt; JZ Alamein International University, Alamein City, Egypt; KA College of Nursing, Imam Mohammad Ibn Saud Islamic University (IMSIU), Riyadh, Saudi Arabia; KB Department of Medicine, University of Texas, San Antonio, TX, USA; KC Department of Pharmacology, Saveetha University, Chennai, India; KD Department of Biochemistry and Forensic Science, Federal University of Technology Owerri, Owerri, Nigeria; KE Department of Epidemiology and Medical Statistics, University of Ibadan, Ibadan, Nigeria; KF Research Centre for Healthcare and Community, Coventry University, Coventry, UK; KG Tanner College of Dental Medicine, University of Pikeville, Pikeville, KY, USA; KH School of Dental Medicine, BAU International University Batumi, Batumi, Georgia; KI National Cancer Center, Head and Neck Surgical Oncology Program, National Institute of Health, Bethesda, MD, USA; KJ College of Dentistry, Ajman University Ajman UAE, Ajman, United Arab Emirates; KK Pediatric Institute, Cleveland Clinic, Cleveland, OH, USA; KL Dentistry Research Institute, Tehran University of Medical Sciences, Tehran, Iran; KM Department of Social Medicine and Epidemiology, Guilan University of Medical Sciences, Rasht, Iran; KN Department of Chemistry, Duke University, Durham, NC, USA; KO i3S - Instituto de Investigação e Inovação em Saúde, University of Porto, Porto, Portugal; KP Department of FEUP - Faculdade de Engenharia Da Universidade Do Porto, University of Porto, Porto, Portugal; KQ Department of Social Sciences, University of Nicosia, Nicosia, Cyprus; KR Department of Nursing, Wollega University, Nekemte, Ethiopia; KS Department of Biomedical, Metabolic and Neural Sciences, University of Modena and Reggio Emilia, Modena, Italy; KT School of Public Health, University of California Berkeley, Berkeley, CA, USA; KU Institute of Public Health, Charité Universitätsmedizin Berlin (Charité Medical University Berlin), Berlin, Germany; KV James Cancer Hospital at the Ohio State University, Ohio State University, Columbus, OH, USA; KX Faculty of Physiotherapy, University of Valencia, Valencia, Spain; KY Saveetha Institute of Medical and Technical Sciences, Saveetha University, Chennai, India; KZ Department of Dermatology, Kyoto Prefectural University of Medicine, Kyoto, Japan; LA Health Services Management Training Centre, Semmelweis University, Budapest, Hungary; LB Department of Applied Social Sciences, Sapientia Hungarian University of Transylvania, Târgu-Mureş, Romania; LC Department of Dentistry, Government Medical College, Nagpur, India; LD Department of Community Dentistry, Mahidol University, Ratchathewi, Thailand; LE Population and Patient Health Group, King’s College London, London, UK; LF Department of Public Health, University of Szeged, Szeged, Hungary; LG Department of Medical Epidemiology, Mario Negri Institute for Pharmacological Research, Milan, Italy; LH Department of Health Policy and Administration, University of the Philippines Manila, Manila, Philippines; LI Cochlear Center for Hearing and Public Health, Johns Hopkins University, Philadelphia, PA, USA; LJ Institute of Public Health, Jagiellonian University Medical College, Krakow, Poland; LK School of Medicine and Population Health, University of Sheffield, Sheffield, UK; LL School of Public Health, Qazvin University of Medical Sciences, Qazvin, Iran; LM Department of Dermatology, Mazandaran University of Medical Sciences, Sari, Iran; LN Orthodontics Department, Mashhad University of Medical Sciences, Mashhad, Iran; LO Obstetrics and Gynecology Department, Shahid Beheshti University of Medical Sciences, Tehran, Iran; LP International University of Health and Welfare, International University of Health and Welfare, Narita, Japan; LQ Warwick Medical School, University of Warwick, Coventry, UK; LR Department of Radiation Oncology, All India Institute of Medical Sciences, Bathinda, India; LS Department of Social and Preventive Dentistry, Federal University of Pelotas, Pelotas, Brazil; LT University Institute of Public Health, The University of Lahore, Lahore, Pakistan; LU Department of Pediatrics, Mersin City Training and Research Hospital, Mersin, Türkiye; LV Department of Genetics, Sana Institute of Higher Education, Sari, Iran; LW Universal Scientific Education and Research Network (usern), Kermanshah University of Medical Sciences, Kermanshah, Iran; LX Department of Oral Medicine & Radiology, Government Dental College & Hospital, Nagpur, India; LY College of Dentistry, Ajman University, Ajman, United Arab Emirates; LZ Oncological Network, Prevention and Research Institute, Institute for Cancer Research, Prevention and Clinical Network, Florence, Italy; MA Postgraduate Program in Epidemiology, Federal University of Rio Grande do Sul, Porto Alegre, Brazil; MB Department of Gastroenterology, Cangnan Hospital of Traditional Chinese Medicine, Wenzhou, China; MC Medical Directorate, Local Health Authority of Ferrara, Ferrara, Italy; MD Community and Family Medicine, Central Armed Police Forces Institute of Medical Sciences (AIIMS-CAPFIMS), Delhi, India; ME Department of Microbiology, Rama Medical College Hospital and Research Centre, Kanpur, India; MF Department of Public Health, Torrens University Australia, Melbourne, VIC, Australia; MG Department of Toxicology, Shriram Institute for Industrial Research, Delhi, India; MH Faculty of Health, University of the Valley, Cali, Colombia; MI Oral and Maxillofacial Surgery Department, Shahid Beheshti University of Medical Sciences, Tehran, Iran; MJ Department of Biological Sciences, King Abdulaziz University, Jeddah, Saudi Arabia; MK Department of Maxillofacial Surgery and Diagnostic Sciences, Jazan University, Jazan, Saudi Arabia; ML Department of Community Medicine, Post Graduate Institute of Medical Education and Research, Chandigarh, India; MM Centre for Community Medicine, All India Institute of Medical Sciences, New Delhi, India; MN Department of Electrical Engineering, Salahaddin University-Erbil, Erbil, Iraq; MO Training Center, Kurdistan Higher Council of Medical Specialties, Sulaymaniyah, Iraq; MP Oral & Maxillofacial Rehabilitation Department, King Abdulaziz University, Jeddah, Saudi Arabia; MQ Department of Fixed Prosthodontics, Cairo University, Cairo, Egypt; MR Artificial Intelligence Research and Innovation Hub, Hamad Medical Corporation, Doha, Qatar; MS Qatar University, Doha, Qatar; MT Department of Neurosurgery, Mayo Clinic, Rochester, USA; MU Department of Computer and Information Science, Majmaah University, Al Majmaah, Saudi Arabia; MV Faculty of Nursing, Chulalongkorn University, Bangkok, Thailand; MW Faculty of Dentistry, Jamia Millia Islamia, New Delhi, India; MX Department of Food Technology and Nutrition Science, Noakhali Science and Technology University, Noakhali, Bangladesh; MY Department of Ophthalmology, Alborz University of Medical Sciences, Karaj, Iran; MZ Department of Periodontics, RAK Medical & Health Sciences University, Ras Al Khaimah, United Arab Emirates; NA Department of Oral Rehabilitation, University of Khartoum, Khartoum, Sudan; NB Department of Periodontia & Community Dentistry, Aligarh Muslim University, Aligarh, India; NC Department of Pharmaceutical Technology, Adamas University, Kolkata, India; ND Department of Oral and Maxillofacial Surgery, Key Laboratory of Shaanxi Province for Craniofacial Precision Medicine Research, Xi’An, China; NE Community-Oriented Nursing Midwifery Research Center, Shahrekord University of Medical Sciences, Shahrekord, Iran; NF Independent Consultant, Santa Clara, CA, USA; NG Departamento de Salud Oral (Department of Oral Health), Universidad Autónoma de Manizales (Autonomous University of Manizales), Manizales, Colombia; NH Department of Medical Biochemical Analysis, Cihan University-Erbil, Erbil, Iraq; NI Department of Microbiology, Taiz University, Taiz, Yemen; NJ School of Medicine, Nankai University, Tianjin, China; NK School of Dentistry, Hanoi Medical University, Hanoi, Viet Nam; NL Faculty of Health, University of Canterbury, Christchurch, New Zealand; NM Kasturba Medical College Mangalore, Manipal Academy of Higher Education, Manipal, India; NN Department of Public Health, University of Science and Technology Chittagong (USTC), Chattogram, Bangladesh; NO Department of Public Health, Daffodil International University (DIU), Dhaka, Bangladesh; NP School of Engineering and Technology, Duy Tan University, Da Nang, Viet Nam; NQ Department of AI, Galgotias University, Greater Noida, India; NR Department of Legal Medicine and Bioethics, Carol Davila University of Medicine and Pharmacy, Bucharest, Romania; NS Department of Clinical Legal Medicine, National Institute of Legal Medicine Mina Minovici, Bucharest, Romania; NT Department of Otorhinolaryngology Head and Neck Surgery, Shanghai Jiao Tong University, Shanghai, China; NU Department of Preventive and Social Dentistry, Federal University of Rio Grande do Sul, Porto Alegre, Brazil; NV Department of Occupational Safety and Health, China Medical University, Taiwan, Taichung, Taiwan; NW Science and Technology Park, Kazakh National Medical University, Almaty, Kazakhstan; NX Department of Biology, University of Zakho, Zakho, Iraq; NY Department of Cardiovascular Medicine, Cleveland Clinic, Cleveland, OH, USA; NZ West Africa RCC, Africa Centre for Disease Control and Prevention, Abuja, Nigeria; OA Department of Community Medicine, University College Hospital, Ibadan, Ibadan, Nigeria; OB Faculty of Medicine, University of Belgrade, Belgrade, Serbia; OC Faculty of Medical Sciences, University of Kragujevac, Kragujevac, Serbia; OD Department of Health Information Management, Buraydah Private Colleges, Al-Qassim, Saudi Arabia; OE University of Michigan, Ann Arbor, MI, USA; OF School of Pharmacy, BRAC University, Dhaka, Bangladesh; OG Department of General Surgery and Medical-Surgical Specialties, University of Catania, Catania, Italy; OH Department of Hospital Medicine, University of Tsukuba, Tsukuba, Japan; OI School of Health Systems and Public Health, University of Washington, Seattle, WA, USA; OJ College of Medicine and Health Sciences, Arabian Gulf University, Manama, Bahrain; OK Government Hospitals, Manama, Bahrain; OL Department of Oral Pathology and Microbiology, King George’s Medical University, Lucknow, India; OM Department of Health and Safety, Dubai Municipality, Dubai, United Arab Emirates; ON Section of Economic & Social Sciences, Humanities & Arts, The World Academy of Sciences UNESCO-TWAS, Trieste, Italy; OO Shaanxi University of Technology, Hanzhong, China; OP Department of Nursing, King Faisal University, Al-Ahsa, Saudi Arabia; OQ Department of Environmental Engineering, Islamic Azad University, Ahvaz, Iran; OR Division of Medical Research, Sri Ramaswamy Memorial Institute of Science and Technology, Kattankulathur, India; OS The Medical City for Military and Security Services School, Muscat, Oman; OT Department of Oral Medicine and Periodontology, University of Peradeniya, Peradeniya, Sri Lanka; OU Department of Oral Medicine and Periodontology, Saveetha University, Chennai, India; OV National Cancer Control Institute, National Cancer Center, Goyang-Si, South Korea; OW Department of Health Economics and Policy Management, Johns Hopkins University, Baltimore, MD, USA; OX Department of Community Medicine, Manipal Academy of Higher Education, Mangalore, India; OY Institute of National Importance, All India Institute of Medical Sciences, Rajkot, India; OZ Gastrointestinal and Liver Diseases Research Center, Guilan University of Medical Sciences, Rasht, Iran; PA Caspian Digestive Disease Research Center, Guilan University of Medical Sciences, Rasht, Iran; PB Division of Dentistry, University of Manchester, Manchester, UK; PC Department of Psychiatry, University of Oxford, Oxford, UK; PD Department of Family Medicine and Public Health, University of Opole, Opole, Poland; PE Department of Pathology, Saveetha University, Chennai, India; PF School of Computer Science and Engineering, RV University, Bengaluru, India; PG Research Department, TobaccoFree Research Institute Ireland, Dublin, Ireland; PH School of Public Health, University College Cork, Cork, Ireland; PI Department of Oral and Maxillofacial Pathology and Microbiology, Krishna Vishwa Vidyapeeth Deemed to be University, Karad, India; PJ Faculty of Science, Al-Baha University, Saudi Arabia, Al-Baha, Saudi Arabia; PK Independent Consultant, Pune, India; PL Department of Periodontology, Louisiana State University Health Sciences Center, New Orleans, LA, USA; PM Office of the Executive Director, Cephas Health Research Initiative Inc., Ibadan, Nigeria; PN Department of Public Health, Thomas Adewumi University, Oko, Nigeria; PO Center for Marine and Aquatic Research, Saveetha University, Chennai, India; PP The Hansjörg Wyss Department of Plastic and Reconstructive Surgery, New York University, New York, NY, USA; PQ School of Dentistry, Kazakh National Medical University, Almaty, Kazakhstan; PR Department of Health Policy and Community Health, Georgia Southern University, Statesboro, GA, USA; PS Department of Research Management, Research Institute of Cardiology and Internal Diseases, Almaty, Kazakhstan; PT Chair and Department of Medical Microbiology, Poznan University of Medical Sciences, Poznan, Poland; PU Department of Biomedical Sciences, Aksum University, Aksum, Ethiopia; PV Clinical Anatomy Department, Debre Tabor University, Addis Ababa, Ethiopia; PW Surgery Research Unit, University of Oulu, Oulu, Finland; PX Department of Molecular Medicine and Surgery, Karolinska Institute, Stockholm, Sweden; PY Center for Molecular Biosciences and Medical Sciences, University of Medical Sciences, Ondo, Ondo, Nigeria; PZ Department of Public Health Nutrition and Nursing Related, Bahir Dar University, Bahir Dar, Ethiopia; QA Department of Public Health, Jordan University of Science and Technology, Irbid, Jordan; QB Amity Institute of Forensic Sciences, Amity University, Noida, India; QC Department of Cardiology, University of South Wales, Treforest, UK; QD Department of Cardiology, University of Buckingham, Buckingham, UK; QE Department of Biotechnology and Applied Clinical Sciences, University of L’Aquila, L’Aquila, Italy; QF Riphah School of Computing & Innovation, Riphah International University, Lahore, Pakistan; QG Center of Excellence in Precision Medicine and Digital Health, Chulalongkorn University, Bangkok, Thailand; QH Department of Pharmacology, All India Institute of Medical Sciences, Raipur, India; QI Department of Biochemistry, Liaquat University Of Medical and Health Sciences, Jamshoro, Pakistan; QJ Department of Biomedical Informatics, Korea University, Seoul, South Korea; QK Cardiovascular Disease Initiative, Broad Institute of MIT and Harvard, Cambridge, MA, USA; QL Massachusetts General Hospital, Boston, MA, USA; QM Department of Transdisciplinary Medicine, Seoul National University Hospital, Seoul, South Korea; QN Healthcare AI Research Institute, Seoul National University Hospital, Seoul, South Korea; QO Health and Healing Research, Education, and Service, Inc., Boston, MA, USA; QP Millennium Prevention, Inc., Westwood, MA, USA; QQ Department of Community Medicine, Manipal Academy of Higher Education, Manipal, India; QR School of Health Sciences, Kristiania University College, Oslo, Norway; QS Department of International Health and Sustainable Development, Tulane University, New Orleans, LA, USA; QT Department of Otorhinolaryngology Head and Neck Surgery, University of Zürich, Zurich, Switzerland; QU Department of Public Health Dentistry, Krishna Vishwa Vidyapeeth (Deemed to be University), Karad, India; QV Department of Public Health and Community Medicine, Central University of Kerala, Kasaragod, India; QW Center for Tobacco Control Research and Education, University of California San Francisco, San Francisco, CA, USA; QX Children’s Medical Center, Tehran University of Medical Sciences, Tehran, Iran; QY Amity Institute of Public Health and Hospital Administration, Amity University, Noida, India; QZ Department of Anthropology, Panjab University, Chandigarh, India; RA Department of Physiology, Sree Balaji Medical College and Hospital, Chennai, India; RB Department of Oral Biology, Saveetha University, Chennai, India; RC Dental School, The University of Western Australia, Perth, WA, Australia; RD Rajendra Institute of Medical Sciences, Ministry of Health and Family Welfare, Ranchi, India; RE Health Research Institute, Al Farabi Kazakh National University, Almaty, Kazakhstan; RF Department of Health Administration and Education, University of Education, Winneba, Ghana; RG Lady Hardinge Medical College, Lady Hardinge Medical College, New Delhi, India; RH Department of Pediatrics, Post Graduate Institute of Medical Education and Research, Chandigarh, India; RI Wits Advanced Drug Delivery Platform Research Unit, University of the Witwatersrand, Johannesburg, South Africa; RJ Wits Infectious Diseases and Oncology Research Institute, University of the Witwatersrand, Johannesburg, South Africa; RK Department of Biomedical Sciences, The Apollo University, Chittoor, India; RL Department of Community Medicine & School of Public Health, Post Graduate Institute of Medical Education and Research, Chandigarh, India; RM Department of Medicine and Surgery, Mayo Clinic, Rochester, MN, USA; RN Department of Biochemistry, All India Institute of Medical Sciences, Bhopal, India; RO Department of Mathematics, Amity University Haryana, Gurugram, India; RP Dow Medical College, Dow University of Health Sciences, Karachi, Pakistan; RQ Department of Community Medicine, Rajendra Institute of Medical Sciences, Ranchi, India; RR Faculty of Education, Shree Guru Gobind Singh Tricentenary University, Gurugram, India; RS Nursing & Midwifery Research Department (NMRD), Hamad Medical Corporation, Doha, Qatar; RT Lincoln Institute of Rural and Coastal Health, University of Lincoln, Lincoln, UK; RU Department of Medicine, McMaster University, Hamilton, ON, Canada; RV Department of Microbiology, Central University of Punjab, Bathinda, India; RW Department of Public Health and Epidemiology, Khalifa University of Science and Technology, Abu Dhabi, United Arab Emirates; RX Faculty of Public Health, University of Indonesia, Depok, Indonesia; RY Department of Psychology, University of Helsinki, Helsinki, Finland; RZ Department of Psychology, Norwegian University of Science and Technology, Trondheim, Norway; SA Indian Council of Medical Research, New Delhi, India; SB School of Dentistry, The University of Queensland, Brisbane, QLD, Australia; SC Department of Medical Physics and Informatics, University of Szeged, Szeged, Hungary; SD Department of Clinical and Experimental Medicine, University of Catania, Catania, Italy; SE Department of Otorhinolaryngology, Father Muller Medical College, Mangalore, India; SF Department of Precision Medicine, Sungkyunkwan University, Suwon-Si, South Korea; SG Department of Microbiology, Sri Ramaswamy Memorial Institute of Science and Technology, Chengalpattu, India; SH Faculty of Science, Universiti Brunei Darussalam (University of Brunei Darussalam), Bandar Seri Begawan, Brunei; SI Department of Psychiatry & Human Behavior, University of California Irvine, Irvine, CA, USA; SJ School of Medicine, Shanghai Jiao Tong University, Shanghai, China; SK Department of Health Promotion and Health Education, National Taiwan Normal University, Taipei, Taiwan; SL Clinical Sciences, Shaqra University, Sharqa, Saudi Arabia; SM Department of Evidence-Based Stomatology, Clinical Research Center for Stomatology, Chengdu, China; SN Faculty of Medicine OWL, Bielefeld University, Bielefeld, Germany; SO B. Atchabarov SRI FAM, Kazakh National Medical University, Almaty, Kazakhstan; SP Faculty of Medicine, Umeå University, Umeå, Sweden; SQ School of Basic Medical Science, Shandong University, Jinan, China; SR Department of Medicine, University of Alberta, Edmonton, AB, Canada; SS Tulane University, New Orleans, LA, USA; ST Faculty of Pharmacy, Tishk International University, Erbil, Iraq; SU Department of Pharmaceutical Science, University of Eastern Piedmont, Novara, Italy; SV Faculty of Dental Medicine and Oral Health Sciences, McGill University, Montreal, QC, Canada; SW Center for Global Health, University of Pennsylvania, Philadelphia, PA, USA; SX Department of Periodontology, Pomeranian Medical University, Szczecin, Poland; SY Department of Physiology and Biochemistry, University of Veterinary Medicine Budapest, Budapest, Hungary; SZ Center for Integrative Omics Data Science (ciods), Yenepoya University, Mangalore, India; TA College of Dentistry, University of Hail, Hail, Saudi Arabia; TB Thamar University, Dhamar, Yemen; TC Ophthalmology Department, Dr. Soliman Fakeeh Hospital, Riyadh, Saudi Arabia; TD Discipline of Pharmacy, James Cook University, Cairns, QLD, Australia; TE Research Center, Cihan University-Sulaimaniya, Sulaymaniyah, Iraq; TF Maternal and Child Health Division (MCHD), International Centre for Diarrhoeal Disease Research, Bangladesh, Dhaka, Bangladesh; TG University Institute of Radiological Sciences and Medical Imaging Technology, The University of Lahore, Lahore, Pakistan; TH Institute of Molecular Biology and Biotechnology, The University of Lahore, Lahore, Pakistan; TI College of Medicine and Health Sciences, National University of Science and Technology, Oman, Sohar, Oman; TJ Saveetha Institute of Basic Medical Sciences (sibms), Saveetha University, Chennai, India; TK Department of Clinical and Experimental Medicine, University of Pisa, Pisa, Italy; TL Department of Health Policy Research, Public Health Foundation of India, Gurugram, India; TM Institute of Population Health Sciences, University of Liverpool, Liverpool, UK; TN Department of Community Medicine, Geetanjali Medical College and Hospital in Udaipur India, Udaipur, India; TO Department of Prosthetic Dental Sciences, Jazan University, Jazan, Saudi Arabia; TP Department of Dental Surgery, Wroclaw Medical University, Wrocław, Poland; TQ Department of Preventive Oncology, All India Institute of Medical Sciences, New Delhi, India; TR National Heart, Lung, and Blood Institute, Bethesda, MD, USA; TS Research and Development Department, Lahore Medical Research Center, Lahore, Pakistan; TT Department of Dental Research Cell, Dr. D. Y. Patil Vidyapeeth, Pune (Deemed to be University), Pune, India; TU Dental Public Health Department, Faculty of Dentistry, King Abdulaziz University, Jeddah, Saudi Arabia; TV Turning Point, Eastern Health Clinical School, Monash University, Melbourne, VIC, Australia; TW Department of Pathology, Imam Abdulrahman Bin Faisal University, Dammam, Saudi Arabia; TX University Centre Varazdin, University North, Varazdin, Croatia; TY Dermatology Unit, Fondazione IRCCS Policlinico San Matteo, Pavia, Italy; TZ Multidisciplinary Department of Medical-Surgical and Dental Specialties, University of Campania Luigi Vanvitelli, Naples, Italy; UA Saveetha Dental College and Hospitals, Saveetha University, Chennai, India; UB Boston University School of Public Health, Boston University, Boston, MA, USA; UC Department of Health Education and Promotion, Ahvaz Jundishapur University of Medical Sciences, Ahvaz, Iran; UD Molecular Biology Unit, Sirius Training and Research Centre, Khartoum, Sudan; UE Bio-Statistical and Molecular Biology Department, Sirius Training and Research Centre, Khartoum, Sudan; UF Clinical Nutrition Department, College of Nursing and Health Sciences, Jazan University, Jazan, Saudi Arabia; UG Medical Microbiology Department, Usmanu Danfodiyo University, Sokoto, Sokoto, Nigeria; UH Medical Microbiology Department, Usmanu Danfodiyo University Teaching Hospital, Sokoto, Nigeria; UI Department of Applied Biology, University of Science and Technology Meghalaya, Ri-Bhoi, India; UJ Department of Physiology, All India Institute of Medical Sciences, Deoghar, India; UK Computer, Electrical, and Mathematical Sciences and Engineering Division, King Abdullah University of Science and Technology, Thuwal, Saudi Arabia; UL Department of Public Health, Oswaldo Cruz Foundation, Recife, Brazil; UM Department of Public Health, Federal University of Pernambuco, Recife, Brazil; UN School of Medicine, Tehran University of Medical Sciences, Tehran, Iran; UO Faculty of Biotechnologies (biotech), ITMO University, Saint Petersburg, Russia; UP Department of Radiology, University of Tripoli, Tripoli, Libya; UQ PMAS Arid Agriculture University Rawalpindi, Rawalpindi, Pakistan; UR Unit of Pharmacotherapy, Epidemiology and Economics, University of Groningen (Rijksuniversiteit Groningen), Groningen, Netherlands; US Department of Epidemiology and Biostatistics, Wuhan University, Wuhan, China; UT Centre for Early Childhood Caries Research, Sri Ramachandra University, Chennai, India; UU Santosh University, New Delhi, India; UV School of Public Health, Makerere University, Kampala, Uganda; UW Department of Pathology and Microbiology, Duhok University, Duhok, Iraq; UX Department of Medical Laboratory Technology FAMS, University of Tabuk, Tabuk, Saudi Arabia; UY Prince Fahad Bin Sultan Chair for Biomedical Research, University of Tabuk, Tabuk, Saudi Arabia; UZ Department of Epidemiology, NYU School of Global Public Health, New York University, New York, NY, USA; VA College of Medicine and Public Health, Flinders University, Adelaide, SA, Australia; VB Department of Computer Science and IT, Torrens University, Adelaide, SA, Australia; VC Community Health Centre, National Health Mission, Vellanikkara, India; VD Department of Oral and Craniomaxillofacial Surgery, University of Basel, Basel, Switzerland; VE Department of Pediatrics Oral Health and Orthodontics, University of Basel, Basel, Switzerland; VF School of Dentistry, University of Utah, Salt Lake City, UT, USA; VG Department of Restorative and Prosthetic Dental Sciences, Dar Al Uloom University, Riyadh, Saudi Arabia; VH Department of Dental Public Health, King Abdulaziz University, Jeddah, Saudi Arabia; VI Department of Biological Sciences and Chemistry, University of Nizwa, Nizwa, Oman; VJ Kent State University, Kent State University, Kent, OH, USA; VK Department of Pediatrics, Arak University of Medical Sciences, Arak, Iran; VL National Centre for Suicide Research and Prevention, University of Oslo, Oslo, Norway; VM Oral Health Centre of Expertise in Rogaland, Oral Health Center of Expertise in Rogaland, Stavanger, Norway; VN HSC, Cancer Institute, West Virginia University, Morgantown, WV, USA; VO Milken Institute School of Public Health, George Washington University, Washington Dc, DC, USA; VP Institute for Advanced Study in Technology, Ton Duc Thang University, Ho Chi Minh City, Viet Nam; VQ International Institute of Training and Research (instar), University of Medicine and Pharmacy, Vietnam National University (UMP-VNU), Hanoi, Viet Nam; VR Institute for Global Health Innovations, Duy Tan University, Da Nang, Viet Nam; VS School of Medicine, Health Research Institute, University of Limerick, Limerick, Ireland; VT Center for Public Health, Teesside University, Middlesbrough, UK; VU Department of Social and Preventive Medicine, University of Malaya, Kuala Lumpur, Malaysia; VV School of Health Sciences, Hamdan Bin Mohammed Smart University, Dubai, United Arab Emirates; VW Dalla Lana School of Public Health, University of Toronto, Toronto, ON, Canada; VX Department of Applied Economics and Quantitative Analysis, University of Bucharest, Bucharest, Romania; VY Bioinformatics Department, National Institute of Research and Development for Biological Sciences, Bucharest, Romania; VZ Department of Zoology, King Saud University, Riyadh, Saudi Arabia; WA Department of Microbiology, University of Medical Sciences, Ondo, Ondo, Nigeria; WB One Health Research Group, Universidad De Las Américas, Quito, Ecuador; WC Department of Respiratory Medicine, Jagadguru Sri Shivarathreeswara Academy of Health Education and Research, Mysore, India; WD National School of Public Health, Institute of Health Carlos III, Madrid, Spain; WE Department of Forensic Medicine and Toxicology, Manipal Academy of Higher Education, Mangalore, India; WF Centre for Biotechnology, Siksha ‘O’ Anusandhan Deemed to be University, Bhubaneswar, India; WG SRM School of Public Health, Faculty of Medicine and Health Sciences, Sri Ramaswamy Memorial Institute of Science and Technology, Chengalpattu, India; WH Division of Ophthalmology & Visual Sciences, University of Nottingham, Nottingham, UK; WI First Department of Ophthalmology, Aristotle University of Thessaloniki, Thessaloniki, Greece; WJ Vision and Eye Research Institute, Anglia Ruskin University, Cambridge, UK; WK Division of Health Policy and Management, University of Minnesota, Minneapolis, MN, USA; WL Medical Big Data Research Center, Seoul National University, Seoul, South Korea; WM Department of Forensic Medicine and Toxicology, All India Institute of Medical Sciences, Hyderabad, India; WN Department of Life Sciences and Public Health, Catholic University of Sacred Heart, Rome, Italy; WO Department of Computer Science and Bioscience, Marwadi University, Rajkot, India; WP Ingenus Pharmaceuticals, LLC, Long Island University, Brooklyn, Fairfield, NJ, USA; WQ Department of Physical Therapy, Right Motion Physical Therapy PT PC., Woodbury, NY, USA; WR Faculty of Medicine and Health, University of Leeds, Leeds, UK; WS College of Nursing, All India Institute of Medical Sciences, Deoghar, India; WT College of Dental Medicine, Roseman University of Health Sciences, South Jordan, UT, USA; WU Department of Genetics, Yale University, New Haven, CT, USA; WV Department of Mathematical and Computer Sciences, University of Medical Sciences, Ondo, Ondo, Nigeria; WW Research Workgroup, Polish Dermatoscopy Group, Poznan, Poland; WX Skin Cancer Unit, Zwierzyniecka Medical Center, Poznan, Poland; WY Department of Dental Public Health, Pushpagiri Institute of Medical Sciences, Tiruvella, India; WZ Centro de Investigaciones Clinicas (Clinical Research Center), Valle del Lili Foundation (Fundación Valle del Lili), Cali, Colombia; XA Department of Centro PROESA, Universidad ICESI, Cali, Colombia; XB Centre for Dental Education and Research, All India Institute of Medical Sciences, New Delhi, India; XC Egas Moniz Center for Interdisciplinary Research (ciiem), Instituto Universitário Egas Moniz, Almada, Portugal; XD Department of Medical Instrumentation Techniques Engineering, Al-Rafidain University, Baghdad, Iraq; XE Department of Mathematics and Informatics, Luhansk Taras Shevchenko National University, Luhansk, Ukraine; XF Rory Meyers College of Nursing, New York University, New York, NY, USA; XG Xiangya Hospital, Central South University, Changsha, China; XH Faculty of Nursing, Universitas Airlangga, Surabaya, Indonesia; XI Research Center in Advancing Community Healthcare, Surabaya, Indonesia; XJ Department of Medical Oncology, Cancer Institute (W.I.A), Chennai, India; XK Manipal College of Dental Sciences, Manipal Academy of Higher Education, Manipal, India; XL Oman Dental College, Muscat, Oman; XM Department of Medical Biology, Kocaeli University, Izmit, Türkiye; XN Internal Medical Faculty, Osh State University, Osh, Kyrgyzstan; XO Department of Population Science and Human Resource Development, University of Rajshahi, Rajshahi, Bangladesh; XP Faculty of Medicine, University of Setif Algeria, Setif, Algeria; XQ Lirssei Research Lab, University of Setif Algeria, Setif, Algeria; XR Department of Nursing Science, Nigerian Institute of Medical Research, Kano, Nigeria; XS Department of Chemical Engineering and Processing, West Pomeranian University of Technology in Szczecin, Szczecin, Poland; XT Department of Radiology, Stanford University, Stanford, CA, USA; XU Centre for Marine and Aquatic Research, Saveetha University, Chennai, India; XV All India Institute of Medical Sciences, Bilaspur, India; XW Department of Dentistry, All India Institute of Medical Sciences, Bathinda, India; XX Nitte (deemed To Be University), AB Shetty Memorial Institute of Dental Sciences, Nitte University, Mangalore, India; XY Department of Oral Pathology, Microbiology and Forensic Odontology, Sharavathi Dental College and Hospital, Shimogga, India; XZ Department of Medicine, All India Institute of Medical Sciences, Bhubaneswar, India; YA Department of Family Medicine, Rajarata University of Sri Lanka, Anuradhapura, Sri Lanka; YB Siksha ‘O’ Anusandhan University, Siksha ‘O’ Anusandhan Deemed to be University, Bhubaneswar, India; YC Department of Primary Care and Public Health, Imperial College London, London, UK; YD Department of Academic Public Health England, Public Health England, London, UK; YE Department of Infection Prevention & Control, King Saud bin Abdulaziz University for Health Sciences, Riyadh, Saudi Arabia; YF Department of Biological Sciences, King Abdulaziz University, Jeddah, Egypt; YG Department of Protein Research, Research and Academic Institution, Alexandria, Egypt; YH Department of Pathological Sciences, Ajman University, Ajman, United Arab Emirates; YI Yenepoya Research Centre, Yenepoya University, Mangalore, India; YJ Department of Public Health, Masaryk University, Brno, Czech Republic; YK Faculty of Medicine, National Autonomous University of Mexico, Mexico City, Mexico; YL Department of Digital Health, Carlos Slim Foundation, Mexico City, Mexico; YM Community Health Department, Federal University of Ceará, Fortaleza, Brazil; YN Department of Population Health, Hofstra University, Hempstead, NY, USA; YO Ninth People’s Hospital, Shanghai Jiao Tong University, Shanghai, China; YP Faculty of Medicine, Assiut University, Assiut, Egypt; YQ Department of Medical Physics, Isfahan University of Medical Sciences, Isfahan, Iran; YR Tabriz University of Medical Sciences, Tabriz, Iran; YS Department of Pharmaceutical Chemistry, International Medical University, Gdańsk, Poland; YT Széchenyi István University, Győr, Hungary; YU University College, Korea University, Seoul, South Korea; YV Center for Global Health Research, Saveetha University, Chennai, India; YW Biotechnology Research Center, Mashhad University of Medical Sciences, Mashhad, Iran; YX Sharjah Institute of Medical Sciences, University of Sharjah, Sharjah, United Arab Emirates; YY Department of Radiation Oncology, All India Institute of Medical Sciences, New Delhi, India; YZ Community Medicine and Family Medicine, All India Institute of Medical Sciences, Bibinagar, Hyderabad, India; ZA Institute of Medical Sciences and SUM Hospital, Siksha ‘O’ Anusandhan Deemed to be University, Bhubaneswar, India; ZB Paediatric Dentistry, UWA Dental School, The University of Western Australia, Perth, WA, Australia; ZC LMU-Munich, Munich, Germany; ZD Institute for Employment Research, Nuremberg, Germany; ZE Department of Nutrition, Federal University of Espirito Santo, Vitória, Brazil; ZF Department of Human Nutrition & Dietetics, Health Services Academy, Islamabad, Pakistan; ZG Entomology VectorLink, Njala University, Freetown, Sierra Leone; ZH Department of Entomology, Ain Shams University, Cairo, Egypt; ZI Medical Ain Shams Research Institute (MASRI), Ain Shams University, Cairo, Egypt; ZJ Department of Genomics and Biotechnology, Madha Medical College and Research Institute, Chennai, India; ZK Department of Oral Medicine, Universitas Airlangga, Surabaya, Indonesia; ZL National Dental Research Institute Singapore, National Dental Centre Singapore, Singapore, Singapore; ZM Health Services and Systems Research Programme, Duke-NUS Medical School, Singapore, Singapore; ZN Department of Oral Pathology and Microbiology, Dr. D. Y. Patil Vidyapeeth, Pune (Deemed to be University), Pune, India; ZO Department of Medical and Surgical Sciences, University of Bologna, Bologna, Italy; ZP Clinical Studies and Trials Unit (Development Research), Indian Council of Medical Research, New Delhi, India; ZQ UGC Centre of Advanced Study in Psychology, Utkal University, Bhubaneswar, India; ZR Udyam-Global Association for Sustainable Development, Bhubaneswar, India; ZS Department of Food Processing and Nutrition, Karnataka State Akkamahadevi Women University, Vijayapura, India; ZT Carnegie Mellon University, Carnegie Mellon University, Pittsburgh, PA, USA; ZU Institute of Radiology, Neuroradiology and Interventional Therapy, University of Erlangen-Nuremberg, Bayreuth, Germany; ZV Clinic for Conservative Dentistry, Periodontology and Digital Dentistry, University Hospital Munich, Munich, Germany; ZW Operating Room Department, Khomein University of Medical Sciences, Khomein, Iran; ZX Department of Medical Education, Tehran University of Medical Sciences, Tehran, Iran; ZY Faculty of Dentistry, University of Puthisastra, Phnom Penh, Cambodia; ZZ Emergency Department, Manian Medical Centre, Erode, India; AAA Faculty of Nursing, Cairo University, Cairo, Egypt; AAB Jouf University, Sakaka, Saudi Arabia; AAC Dental Materials Research Center, Mashhad University of Medical Sciences, Mashhad, Iran; AAD Department of Chemistry, Institute for Advanced Studies in Basic Sciences (IASBS), Zanjan, Iran; AAE Faculty of Dentistry, Zarqa University, Zarqa, Jordan; AAF Institute of Basic Medical Sciences, Khyber Medical University, Peshawar, Pakistan; AAG Independent Consultant, Karachi, Pakistan; AAH Department of Pharmacology, All India Institute of Medical Sciences, Jodhpur, India; AAI Science Department, Kazakh National Medical University, Almaty, Kazakhstan; AAJ Department of Microbiology and Molecular Genetics, University of Vermont, Burlington, VT, USA; AAK Department of Microbiology, Jahangirnagar University, Dhaka, Bangladesh; AAL Department for Evidence-Based Medicine and Evaluation, University for Continuing Education Krems, Krems, Austria; AAM Department of Chemistry, Graphic Era (Deemed to be University), Dehradun, India; AAN Department of Nursing, Arba Minch University, Arba Minch, Ethiopia; AAO Alva’s Insitute of Medical Sciences & Research Centre, Rajiv Gandhi University of Health Sciences, Moodubidire, India; AAP Manipal College of Dental Sciences, Mangalore, Manipal Academy of Higher Education, Manipal, India; AAQ Department of Biomedical Sciences, University of Zambia, Lusaka, Zambia; AAR Department of Orthodontics, Boston University, Boston, MA, USA; AAS School of Biotechnology, Gautam Buddha University, Gautam Buddh Nagar, India; AAT Clinical Sciences Research Institute, Ahvaz Jundishapur University of Medical Sciences, Ahvaz, Iran; AAU Department of Health Promotion and Behavior, University System of Georgia, Athens, GA, USA; AAV Department of Public Health Medicine, University of KwaZulu-Natal, Durban, South Africa; AAW Department of Medical-Surgical Nursing, Mazandaran University of Medical Sciences, Sari, Iran; AAX Department of Nursing and Health Sciences, Flinders University, Adelaide, SA, Australia; AAY Department of Research and Academics, Kathmandu Cancer Center, Bhaktapur, Nepal; AAZ Person-Centered Research, Monash University, Box Hill, VIC, Australia; ABA Department of Pharmaceutical Microbiology and Biotechnology, Usmanu Danfodiyo University, Sokoto, Sokoto, Nigeria; ABB Faculty of Dentistry, Federal University of Minas Gerais, Belo Horizonte, Brazil; ABC Sport Physical Activity and Health Research & Innovation Center (sprint), Polytechnic Institute of Guarda, Guarda, Portugal; ABD Department of RISE Health, University of Beira Interior, Covilhã, Portugal; ABE School of Medicine, Baylor College of Medicine, Houston, TX, USA; ABF Department of Medicine Service, US Department of Veterans Affairs (VA), Houston, TX, USA; ABG School of Pharmaceutical Sciences, Faculty of Pharmacy, IFTM University, Moradabad, India; ABH Institute of Medical Sciences, Banaras Hindu University, Varanasi, India; ABI Buck Institute for Research on Aging, Novato, CA, USA; ABJ The Institute for Drug Research, The Hebrew University of Jerusalem, Jerusalem, Israel; ABK Mehta Family School of Biosciences and Biomedical Engineering, Indian Institute of Technology Indore, Indore, India; ABL Department of Biochemistry, All India Institute of Medical Sciences, Rajkot, India; ABM Department of Laboratory Medicine, All India Institute of Medical Sciences, New Delhi, India; ABN Department of Dentistry, All India Institute of Medical Sciences, Bhopal, India; ABO Department of Dental Public Health, University of California San Francisco, San Francisco, CA, USA; ABP Four States Dental Care, Neosho, MO, USA; ABQ Medical Research, SRM Medical College Hospital and Research Centre, Sri Ramaswamy Memorial Institute of Science and Technology, Chengalpettu, India; ABR Bahrain Defence Force Royal Medical Services, Bahrain Defence Force Royal Medical Services in Bahrain, Riffa, Bahrain; ABS Department of Oral and Maxillofacial Surgery, Jagadguru Sri Shivarathreeswara University, Mysore, India; ABT Department of Systemic Pathology, Touro College of Osteopathic Medicine, Middletown, NY, USA; ABU Department of Pathology, American University of the Caribbean School of Medicine, Cupecoy, Saint Martin; ABV Department of Oral Pathology, Manipal Academy of Higher Education, Manipal, India; ABW Department of Pathology, Manipal Academy of Higher Education, Manipal, India; ABX Department of Pharmacology and Therapeutics, Arabian Gulf University, Manama, Bahrain; ABY Department of Diagnostic and Interventional Radiology, University Hospital Regensburg, Regensburg, Germany; ABZ Hospital Administration, Sanjay Gandhi Postgraduate Institute of Medical Sciences, Lucknow, India; ACA Hospital Administration, King George’s Medical University, Lucknow, India; ACB Institute of Nutrition and Food Science, University of Dhaka, Dhaka, Bangladesh; ACC Data Science Institute, University of Technology Sydney, Sydney, NSW, Australia; ACD Heart Research Institute, University of Sydney, Sydney, QLD, Australia; ACE Applied Biomedical Research Center, Mashhad University of Medical Sciences, Mashhad, Iran; ACF Children’s Hospital of Philadelphia, University of Pennsylvania, Philadelphia, PA, USA; ACG School of Dentistry and Oral Health, Griffith University, Gold Coast, QLD, Australia; ACH Department of Dentistry and Oral Health, La Trobe University, Bendigo, VIC, Australia; ACI Feinstein Institutes for Medical Research, Urmia University of Medical Sciences, Manhasset, NY, USA; ACJ Department of Environmental, Agricultural and Occupational Health, University of Nebraska Medical Center, Omaha, NE, USA; ACK Clinical Sciences Department, University of Sharjah, Sharjah, United Arab Emirates; ACL Department of Pathology, Alexandria University, Alexandria, Egypt; ACM Department of Dermatology, Carol Davila University of Medicine and Pharmacy, Bucharest, Romania; ACN Department of Dermato-Venereology, Dr. Victor Babes Clinical Hospital of Infectious Diseases and Tropical Diseases, Bucharest, Romania; ACO Yong Loo Lin School of Medicine, National University of Singapore, Singapore, Singapore; ACP Department of Otorhinolaryngology–head & Neck Surgery, Singapore General Hospital, Singapore, Singapore; ACQ Department of Medicine, Kazakh National Medical University, Almaty, Kazakhstan; ACR Department of Cell Therapy and Applied Genomics, King Hussein Cancer Center, Amman, Jordan; ACS College of Pharmacy, Alfaisal University, Riyadh, Saudi Arabia; ACT Dental Department, Specialized Medical Center Hospital, Riyadh, Saudi Arabia; ACU Outpatient Department, Wollega University, Bedele Town, Ethiopia; ACV Department of Public Health, Wollega University, Nekemte, Ethiopia; ACW Department of Economics, The American University in Cairo, Cairo, Egypt; ACX Department of Conservative Dentistry and Endodontics, Manipal Academy of Higher Education, Mangalore, India; ACY Cardiovascular Diseases Research Center, School of Dentistry, Birjand University of Medical Sciences, Birjand, Iran; ACZ School of Dentistry, Birjand University of Medical Sciences, Birjand, Iran; ADA Department of Prosthetic Dentistry, Kazakh National Medical University, Almaty, Kazakhstan; ADB Department of Social Determinants of Health, Hormozgan University of Medical Sciences, Bandar Abbas, Iran; ADC Global Surgery Department, Data Science Center for the Study of Surgery, Injury and Equity in Africa, Buea, Cameroon; ADD Department of Internal Medicine, University of Medicine and Pharmacy at Ho Chi Minh City, Ho Chi Minh City, Viet Nam; ADE Department of Business Analytics, University of Massachusetts Dartmouth, Dartmouth, MA, USA; ADF Harvard T.h. Chan School of Public Health, Harvard University, Boston, MA, USA; ADG Department of Epidemiology and Biostatistics, Haramaya University, Harar, Ethiopia; ADH International Center for Chemical and Biological Sciences, University of Karachi, Karachi, Pakistan; ADI Department of Biology and Biochemistry, University of Houston, Houston, TX, USA; ADJ Independent Consultant, St. Augustine, Trinidad and Tobago; ADK North Central Regional Health Authority, Champ Fleurs, Trinidad and Tobago; ADL College of Health and Medical Sciences, Haramaya University, Harar, Ethiopia; ADM Research and Education Department, Oli Health Magazine Organization (OHMO), Kigali, Rwanda; ADN Department of Orthodontics, University of Trakya, Edirne, Türkiye; ADO Department of Public Health and Epidemiology, University of Debrecen, Debrecen, Hungary; ADP Department of Periodontology, Krishna Vishwa Vidyapeeth (Deemed to be University), Karad, India; ADQ Centre of Molecular Medicine and Diagnostics, Saveetha University, Chennai, India; ADR School of Dentistry and Medical Sciences, Charles Sturt University, Wagga Wagga, NSW, Australia; ADS Population Oral Health, University of Sydney, Sydney, NSW, Australia; ADT Department of Community Medicine and Family Medicine, All India Institute of Medical Sciences, Gorakhpur, India; ADU College of Medicine, Taipei Medical University, Taipei, Taiwan; ADV Henry S.goldman School of Dental Medicine, Boston University, Boston, MA, USA; ADW Centro de Investigación en Nutrición y Salud, National Institute of Public Health, Mexico City, Mexico; ADX Health Initiative of the Americas, University of California Berkeley, Berkeley, CA, USA; ADY Institute of Family Medicine and Public Health, University of Tartu, Tartu, Estonia; ADZ Section for Oral Health, Heidelberg University Hospital, Heidelberg, Germany; AEA Department of Medical Oncology, University of Medicine and Pharmacy “Grigore T Popa” Iasi, Iaşi, Romania; AEB Department of Medical Oncology, Regional Institute of Oncology, Iaşi, Romania; AEC Department of Education, Akal University, Bathinda, India; AED Marshall Centre for Interventions in Infectious Disease, The University of Western Australia, Perth, WA, Australia; AEE School of Agriculture and Food Sustainability, The University of Queensland, Brisbane, QLD, Australia; AEF National Institute of Health Data Science, Peking University, Beijing, China; AEG Stomatology Hospital, Zhejiang University, Hangzhou, China; AEH Health Unit Organization, National Research and Innovation Agency (BRIN), Bogor, Indonesia; AEI Department of Adult Health Nursing/school of Nursing, Mekelle University, Mekelle, Ethiopia; AEJ Department of Community Medicine, Rajarata University of Sri Lanka, Anuradhapura, Sri Lanka; AEK Research Organisation, Inter-Continental Omni-Research in Medicine Collaborative, Berlin, Germany; AEL Department of Anatomy, Histology, and Embryology, Bahir Dar University, Bahir Dar, Ethiopia; AEM Health Policy and Management Department, Johns Hopkins University, Baltimore, MD, USA; AEN School of Public Health Sciences and Technology, Malla Reddy Vishwavidyapeeth, Hyderabad, India; AEO Department of Community Medicine, Apollo Institute of Medical Sciences and Research, Hyderabad, India; AEP Center for Postgraduate Education and Training, National Center for Child Health and Development, Tokyo, Japan; AEQ Bloomberg School of Public Health, Johns Hopkins University, Baltimore, MD, USA; AER Department of Neurosurgery, St. Joseph’s Hospital and Medical Center, Phoenix, AZ, USA; AES Institute of Public Health, University of Gondar, Gondar, Ethiopia; AET Saw Swee Hock School of Public Health, National University of Singapore, Singapore, Singapore; AEU KHANA Center for Population Health Research, Phnom Penh, Cambodia; AEV Department of Pediatrics, Kyung Hee University, Seoul, South Korea; AEW Department of Epidemiology and Cancer Registry Sector, Institute of Oncology Ljubljana, Ljubljana, Slovenia; AEX Department of Family and Community Medicine, University of Hail, Hail, Saudi Arabia; AEY Department of Clinical Sciences, Ajman University, Ajman, United Arab Emirates; AEZ School of Dentistry, University of Jordan, Amman, Jordan; AFA Research and Development Department, Sina Medical Biochemistry Technologies, Shiraz, Iran; AFB Department of Public Health, University of Hail, Hail, Saudi Arabia; AFC Department of Zoology and Entomology, Al-Azhar University, Cairo, Egypt; AFD School of Public Health, Wuhan University, Wuhan, China; AFE Department of Epidemiology and Biostatistics, Zhejiang University, Hangzhou, China; AFF Department of Dentistry, National University of Singapore, Singapore, Singapore; AFG Department of Epidemiology, University of Washington, Seattle, WA, USA; AFH Gilbert and Rose-Marie Chagoury School of Medicine, Lebanese American University, Byblos, Lebanon; AFI Department of Biochemistry and Pharmacogenomics, Medical University of Warsaw, Warsaw, Poland; AFJ Department of Building Engineering and Environment, Palestine Technical University (Kadoorie), Tulkarem, Palestine; AFK Department of Civil Engineering and Sustainable Structures, Palestine Technical University (Kadoorie), Tulkarem, Palestine

## Abstract

**Background:**

WHO's Global Oral Health Action Plan (GOHAP) targets include a 10% relative reduction in the combined prevalence of main oral conditions by 2030. The first GOHAP evaluation was planned for 2023, with subsequent evaluations scheduled for 2026 and 2030. The aim of this systematic analysis of the Global Burden of Diseases, Injuries, and Risk Factors Study (GBD) 2023 was to evaluate global and regional progress (2019–23) towards this target and related complementary indicators.

**Methods:**

Using modelled estimates from epidemiological surveys and registries in 204 countries and territories, we estimated the age-standardised prevalence of untreated caries in deciduous and permanent teeth, severe periodontitis, edentulism, other oral disorders and orofacial clefts, and the age-standardised incidence of lip and oral cavity cancer. Progress towards GOHAP targets was measured by comparing the percentage change from 2019 to 2023 in the above indicators against the stated GOHAP target of a 10% relative reduction.

**Findings:**

Globally, the age-standardised prevalence of main oral conditions declined (−1·55% [95% uncertainty interval −2·32 to −0·82]) between 2019 and 2023, but global cases increased by 3·07% (2·32 to 3·75), from 3·62 billion (3·35 to 3·95) to 3·73 billion (3·45 to 4·06). Among complementary indicators, reductions in age-standardised prevalence were observed for untreated caries in permanent teeth (−1·99% [−2·49 to −1·40]) whereas the age-standardised incidence of lip and oral cavity cancer increased (8·35% [0·13 to 16·32]), as did the age-standardised prevalence of orofacial clefts (2·25% [0·77 to 3·83]). Progress was heterogeneous across GBD super-regions, with south Asia having the largest reductions in the age-standardised prevalence of main oral conditions (−3·34% [−4·95 to −1·80]) and untreated caries in deciduous teeth (−9·09% [−17·62 to −0·08]). Sub-Saharan Africa showed the largest increase in prevalent cases of main oral conditions (9·51% [8·09–10·94]) and age-standardised incidence of lip and oral cavity cancer (14·86% [1·19–32·45]). The high-income super-region had an increase in the age-standardised prevalence of main oral conditions (1·24% [0·56–1·96]) and untreated caries in deciduous teeth (9·21% [6·60–11·89]).

**Interpretation:**

Our modelled estimates of global trends in the prevalence of oral conditions from 2019 to 2023 suggest that early progress is modest and, at the current pace, insufficient to meet the 2030 GOHAP targets. Accelerated, equity-focused prevention and integration of essential oral health-care services within primary care and universal health coverage are needed, alongside strengthened oral cancer control and congenital anomaly care.

**Funding:**

The Gates Foundation.

## Introduction

Oral diseases are a foundational yet persistently underprioritised component of global health, with far-reaching implications for quality of life, health equity, and health systems worldwide.^1^, ^2^, ^3^ Despite being largely preventable, the burden of oral diseases has remained high over the past three decades,^4^ and its economic and social costs underscore the urgent need for robust, equitable global strategies.^5^ Recognition of oral diseases within global public health agendas, including WHO's Global Oral Health Action Plan (GOHAP) and related policy initiatives,^6^, ^7^ reflects an imperative to embed oral disease prevention and health care in broader health systems to achieve sustainable improvements in population health.^8^, ^9^, ^10^

The Global Burden of Diseases, Injuries, and Risk Factors Study (GBD) provides one of the few globally comparable resources for estimating the oral disease burden across locations and over time.^11^, ^12^ Data from GBD 2019 were instrumental in setting evidence-based targets within the WHO GOHAP 2023–2030. GOHAP is a call to action that outlines 11 global targets, six strategic objectives, and 100 recommended actions to be achieved by 2030.^6^ The GOHAP monitoring framework establishes two key overarching global targets: to achieve universal coverage for oral health-care services (target A) and to reduce the oral disease burden (target B). Specifically, target B calls for a relative reduction of 10% by 2030 in the combined global prevalence of the main oral conditions over the life course. Ten complementary indicators accompany overarching global target B, six of which relate to reductions in the prevalence of untreated caries of deciduous teeth (indicator B.4), untreated caries of permanent teeth (B.5), severe periodontitis (B.6), edentulism (B.7), and orofacial clefts (B.9), and reductions in the incidence of lip and oral cavity cancer (B.8). WHO used data from GBD 2019 to establish baseline measures for the GOHAP targets. The first evaluation of GOHAP was scheduled for 2023, with follow-up assessments planned for 2026 and 2029–30.^6^

**Research in context**

**Evidence before this study**

Oral conditions are highly prevalent and contribute to substantial disability globally, but international reporting has often been fragmented, focusing on individual diseases or select regions. Previous systematic analyses for the Global Burden of Diseases, Injuries, and Risk Factors Study (GBD) have generated comparable global estimates for oral conditions. GBD 2010 showed that increases in prevalence and disease burden were largely driven by population growth and ageing, offsetting improvements in age-standardised rates. GBD 2015 reported little progress towards achieving the joint World Dental Federation/WHO goals of reducing oral disease levels by 2020. GBD 2017 quantified the inequalities in burden by World Bank income groups. GBD 2019 estimates supported the development of evidence-based targets within the WHO Global Oral Health Action Plan (GOHAP) 2023–2030. GBD 2021 estimates reported global, regional, and national trends in the burden of oral conditions through 2021; however, these analyses predated, or were not organised around, the GOHAP monitoring framework.

**Added value of this study**

Using data from GBD 2023, we provide the first assessment explicitly aligned with the GOHAP monitoring framework and evaluate early progress during its first evaluation period (ie, 2019–23). We estimated prevalent cases and age-standardised prevalence for untreated caries in deciduous and permanent teeth, severe periodontitis, edentulism, other oral disorders, orofacial clefts, and incident cases and age-standardised incidence for lip and oral cavity cancer. We report the percentage change from 2019 to 2023 with 95% uncertainty intervals at global and GBD super-region levels. Compared with previous GBD analyses, which have emphasised long-term trends, this study reframes GBD outputs into a short-cycle accountability assessment for GOHAP, identifying where progress is occurring, stalling, or reversing.

**Implications of all the available evidence**

Progress towards achieving one of the two overarching GOHAP targets of a 10% reduction in global prevalence of main oral conditions by 2030 has been uneven across the seven GBD super-regions. Successfully achieving all the WHO GOHAP targets will require accelerated, equity-focused prevention and integration of essential oral health-care services within universal health coverage, alongside targeted action for oral cancer control and cleft care in regions with rising burden.

A comprehensive understanding of current trends in oral conditions is essential to drive urgent action and inform long-term policy planning. The aim of this analysis, conducted as part of GBD 2023, was to evaluate global and regional progress towards the GOHAP targets for the prevalence of oral conditions between 2019 and 2023. Country-level progress was not evaluated due to the global scope of the GOHAP targets.

## Methods

### Study overview

This study was designed as a monitoring and accountability analysis of progress towards the GOHAP targets, rather than as a causal assessment of epidemiological change or policy effectiveness. Accordingly, the GBD estimates should be interpreted as modelled approximations that integrate diverse data sources and rely on explicit statistical assumptions. Additionally, the short observation window (2019–23), chosen to align with the GOHAP initial monitoring period, might limit the ability to detect structural changes in disease patterns and should be understood as reflecting early progress rather than long-term trends.

This study was developed as part of the GBD Collaborator Network and GBD Protocol,^13^ with support from the GBD Secretariat, the Institute for Health Metrics and Evaluation (IHME), and the GBD Oral Disorders Collaborator Network under IHME ID 6181. Ethical approval was not required for this study. The complete GBD methodology is available in the primary reports of GBD 2023 and their accompanying appendices.^14^, ^15^ Our study complies with the GATHER statement,^8^ and the GATHER checklist is available in the appendix (p 38).

### Data sources

Seven oral conditions were evaluated: untreated caries in deciduous teeth; untreated caries in permanent teeth; severe periodontitis; edentulism; other oral disorders; lip and oral cavity cancer; and orofacial clefts. Case definitions and corresponding ICD-10 codes are shown in table 1. The term main oral conditions refers to the combined group of untreated caries, severe periodontitis, edentulism, and other oral disorders.

**Table 1:** Case definitions and key modelling inputs for each oral condition

	**Untreated caries in deciduous teeth**	**Untreated caries in permanent teeth**	**Severe periodontitis**	**Edentulism**	**Other oral disorders**	**Lip and oral cavity cancer**	**Orofacial clefts**
Reference definition	Coronal cavity at dentin level or root cavity in cementum	Coronal cavity at dentin level or root cavity in cementum	PPD ≥6 mm (CPITN 4 or CPI 4 or PPD >5 mm) or CAL ≥6 mm	No remaining permanent teeth from clinical oral examination	Dental, tongue, or jaw disorders not included elsewhere	Malignant neoplasms of the lips and oral cavity	Isolated cleft palate and cleft palate with or without cleft lip
ICD-10 code	K02–K02.9	K02–K02.9	K05–K06.9	K08.0–K08.499	K00–K01.1, K03–K04.99, K07–K08, K08.8–K14.9, M26–M27.9	C00–C07, C08–C08.9	Q35–Q37.9
Alternative definitions	Percentage of DMFT >0 with DT/DMFT ratio	Percentage of DMFT >0 with DT/DMFT ratio	CAL ≥5 mm, CAL ≥4 mm, or PPD ≥5 mm	No remaining permanent teeth from self-reports	None	None	None
Data harmonisation: cross-walking using MR-BRT	Alternative to reference definition for people aged <20 years	Alternative to reference definition for people aged <20 years	Alternative to reference definition	Used before GBD 2021, but not since then	None	Cancer registry data to vital registration data	Sources excluded to sources that included chromosomal diagnosis
Location-level covariates	LN-LDI and high prevalence of SSB intake	LN-LDI and high prevalence of SSB intake	LN-LDI, smoking prevalence, and high plasma glucose prevalence	LN-LDI, smoking prevalence, and high plasma glucose prevalence	None	HAQ index	HAQ index, LN-LDI, folic acid per capita, composite fortification standard, and folic acid inclusion
Correction for edentulism	No	Yes	Yes	No	No	No	No

CAL=clinical attachment loss. CPI=community periodontal index. CPITN=community periodontal index of treatment need. DMFT=decayed, missing, and filled teeth. DT=decayed teeth. GBD=Global Burden of Diseases, Injuries, and Risk Factors Study. HAQ=Healthcare Access and Quality. LN-LDI=natural logarithm of the lag-distributed income per capita. MR-BRT=meta-regression-Bayesian, regularised, trimmed. PPD=probing pocket depth. SSB=sugar-sweetened beverages.

Data on untreated caries, severe periodontitis, and edentulism were identified through systematic searches of PubMed, Embase, the Latin American and Caribbean Health Sciences Literature, the Scientific Electronic Library Online, and the Global Health Data Exchange. Data on lip and oral cavity cancer were obtained from population-based cancer registries and vital registration systems. Data on orofacial clefts were derived from birth defects registries, hospital records, insurance claims, and published literature. Data sources used to produce GBD 2023 estimates are listed in the GBD 2023 Sources Tool. More details of the data-seeking approach and data coverage for each oral condition are provided in the appendix (p 4).

### Data processing and disease modelling

Data processing included age–sex splitting, harmonisation to reference definitions, covariate adjustment, and correction for edentulism and comorbidity correction (table 1). Input data on all oral conditions except for lip and oral cavity cancer were analysed with Disease Modelling Meta-Regression (DisMod-MR 2.1) to estimate prevalence, incidence, and mortality by year, age, and sex across 204 countries and territories. For locations with no data inputs, DisMod-MR 2.1 applied a cascading approach across five hierarchical levels (global, GBD super-region, region, country, and subnational), using data from higher levels and country-specific covariates as priors for lower-level estimates.^14^ For lip and oral cavity cancer, mortality was estimated with the Cause of Death Ensemble model (also known as CODEm) to generate cause-specific mortality by age, sex, location, and year. Cancer incidence was derived from these mortality estimates using mortality-to-incidence ratios modelled with spatiotemporal Gaussian process regression.^16^ Percentage changes are reported to two decimal places whereas counts of cases, prevalence, and incidence are reported to three significant figures.

### Assessment of progress towards GOHAP targets

To align with the GOHAP overarching global target and complementary indicators, we report the count of prevalent cases and age-standardised prevalence for all oral conditions except for lip and oral cavity cancer, for which the count of incident cases and age-standardised incidence rate were reported. Age standardisation (per 100 000 population) was done with the GBD standard population to account for variations in both population size and age structure.^17^ Estimates for main oral conditions were adjusted for comorbidity with a microsimulation of 20 000 individuals per location-age-sex-year group. Each condition was assigned on the basis of its marginal prevalence, assuming independence, and combined prevalence was calculated as the proportion of individuals with at least one condition, approximated by 1–Π (1–*p*_i_), where *p*_i_ is the prevalence of each individual oral condition.

We assessed global and regional progress towards GOHAP targets by comparing the percentage change from 2019 to 2023 in the number of prevalent or incident cases, as well as in age-standardised prevalence or incidence, against the stated GOHAP target of a 10% relative reduction. Uncertainty was propagated throughout the estimation process. Estimates represent the mean value across 250 draws from the estimate's distribution, with 95% uncertainty intervals (UIs) calculated as the 2·5th and 97·5th percentile values across the draws. To reduce computing power and time across the estimation process, the number of draws was reduced from 500 in previous GBD iterations to 250 for GBD 2023. Simulations confirmed that estimates and uncertainty were not affected by this reduction.^14^ A percentage change between 2019 and 2023 was considered negligible if its 95% UI included zero.

Countries were grouped into 21 regions according to geographical proximity and epidemiological similarity and subsequently grouped into seven super-regions based on patterns of causes of death. The GBD super-regions are: central Europe, eastern Europe, and central Asia; high income (including North America, western Europe, and Asia Pacific); Latin America and the Caribbean; north Africa and the Middle East; south Asia; southeast Asia, east Asia, and Oceania; and sub-Saharan Africa (appendix figure S1).

### Role of the funding source

The funder of this study had no role in study design, data collection, data analysis, data interpretation, or the writing of the report.

## Results

For each condition, we first present global estimates of prevalent or incident cases, along with variations across super-regions. We then report global age-standardised prevalence or incidence, again highlighting differences across super-regions.

**Figure 1:**
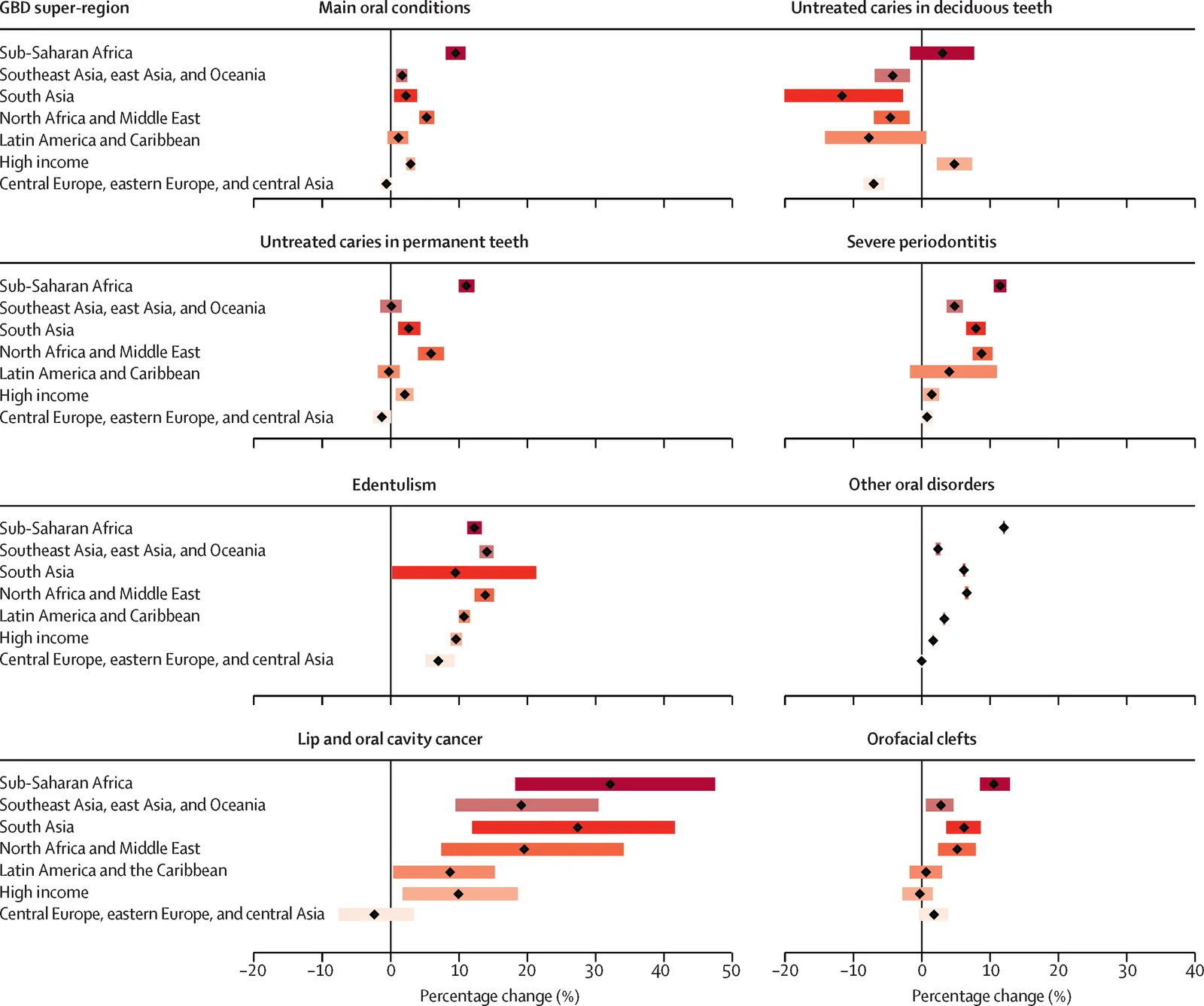
Percentage changes between 2019 and 2023 in prevalent or incident cases of oral conditions, by GBD super-region Bars represent 95% uncertainty intervals. Main oral conditions include untreated caries, severe periodontitis, edentulism, and other oral disorders only. Prevalent cases are shown for all oral conditions except for lip and oral cavity cancer, for which incident cases are shown. The count of prevalent or incident cases, and their corresponding 95% uncertainty intervals, for each oral condition in 2019 and 2023 are shown in the appendix (table S1). GBD=Global Burden of Diseases, Injuries, and Risk Factors Study.

Between 2019 and 2023, global cases of main oral conditions increased by 3·07% (95% UI 2·32–3·75), from 3·62 billion (3·35–3·95) to 3·73 billion (3·45–4·06; table 2). Cases increased across almost all super-regions, ranging from 1·68% (0·79–2·42) in southeast Asia, east Asia, and Oceania to 9·51% (8·09–10·94) in sub-Saharan Africa, except for central Europe, eastern Europe, and central Asia and Latin America and the Caribbean, where no meaningful change was observed (figure 1; appendix table S1).

**Table 2:** Global prevalent or incident cases in 2019 and 2023 and percentage changes in global cases over 2019–23 for oral conditions

		**Prevalent or incident cases in 2019, millions (95% UI)**	**Prevalent or incident cases in 2023, millions (95% UI)**	**Percentage change in cases (95% UI)**
Main oral conditions		3620 (3350 to 3950)	3730 (3450 to 4060)	3·07% (2·32 to 3·75)
	Untreated caries in deciduous teeth	533 (444 to 631)	509 (428 to 590)	−4·56% (−8·51 to −0·43)
	Untreated caries in permanent teeth	2200 (1920 to 2560)	2260 (1980 to 2630)	2·78% (2·19 to 3·41)
	Severe periodontitis	1030 (879 to 1190)	1090 (915 to 1260)	5·73% (4·57 to 6·91)
	Edentulism	336 (289 to 397)	373 (324 to 440)	11·09% (9·64 to 12·93)
	Other oral disorders	148 (142 to 154)	154 (149 to 160)	4·56% (4·42 to 4·69)
Lip and oral cavity cancer		0·363 (0·315 to 0·422)	0·430 (0·367 to 0·505)	18·4% (9·76 to 26·9)
Orofacial clefts		4·05 (3·30 to 4·94)	4·26 (3·46 to 5·20)	5·14% (3·63 to 6·78)

Main oral conditions include untreated caries, severe periodontitis, edentulism, and other oral disorders only. Prevalent cases of main oral conditions do not match the sum of prevalent cases of individual conditions because some conditions coexist within the same individuals. Data are shown as age-standardised prevalence for all oral conditions, except for lip and oral cavity cancer, which is shown as age-standardised incidence. Cases are presented to three significant figures and percentage changes are reported to two decimal places. UI=uncertainty interval.

**Figure 2:**
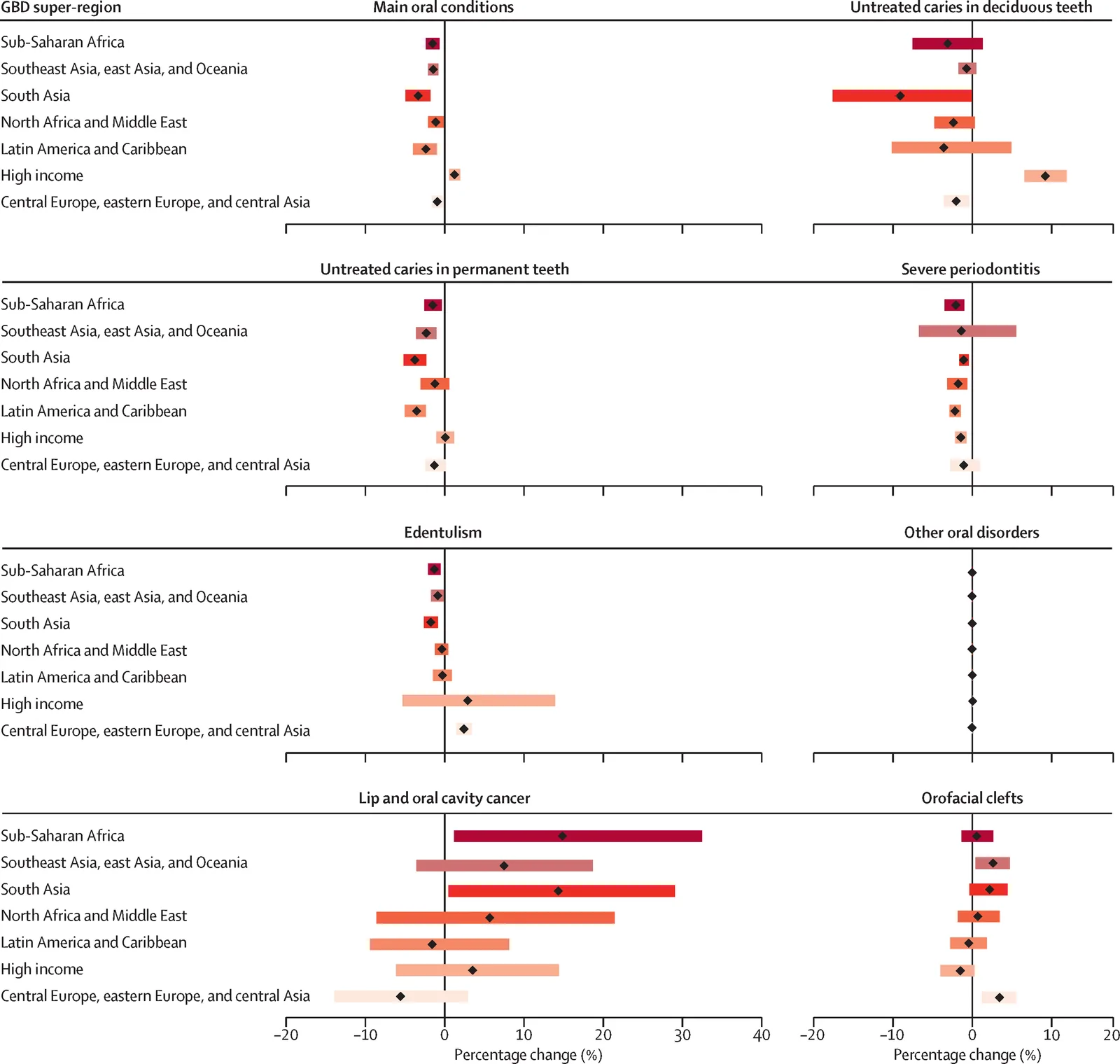
Percentage changes between 2019 and 2023 in age-standardised prevalence or incidence of oral conditions, by GBD super-region Bars represent 95% uncertainty intervals. Main oral conditions include untreated caries, severe periodontitis, edentulism, and other oral disorders only. Age-standardised prevalence is shown for all oral conditions except for lip and oral cavity cancer, for which age-standardised incidence is shown. The age-standardised prevalence or age-standardised incidence, and their corresponding 95% uncertainty intervals, for each oral condition in 2019 and 2023 are shown in the appendix (table S2). GBD=Global Burden of Diseases, Injuries, and Risk Factors Study.

The global age-standardised prevalence of main oral conditions decreased by –1·55% (95% UI –2·32 to –0·82) between 2019 and 2023, from 45 900 (42 400 to 50 200) per 100 000 population to 45 200 (41 800 to 49 100) per 100 000 population (table 3). Reductions were observed in most super-regions, ranging from –0·94% (–1·63 to –0·22) in central Europe, eastern Europe, and central Asia to –3·34% (–4·95 to –1·80) in south Asia, while an increase occurred in the high-income super-region (1·24% [0·56 to 1·96]; figure 2; appendix table S2).

**Table 3:** Global age-standardised prevalence or age-standardised incidence in 2019 and 2023 and percentage changes in global age-standardised estimates over 2019–23 for oral conditions

		**Age-standardised prevalence or incidence in 2019, per 100 000 (95% UI)**	**Age-standardised prevalence or incidence in 2023, per 100 000 (95% UI)**	**Percentage change in age-standardised estimate (95% UI)**
Main oral conditions		45 900 (42 400 to 50 200)	45 200 (41 800 to 49 100)	−1·55% (−2·32 to −0·82)
	Untreated caries in deciduous teeth	7590 (6330 to 8980)	7310 (6160 to 8450)	−3·68% (−7·78 to 0·48)
	Untreated caries in permanent teeth	27 600 (24 100 to 32 200)	27 100 (23 600 to 31 600)	−1·99% (−2·49 to −1·40)
	Severe periodontitis	12 500 (10 600 to 14 400)	12 400 (10 400 to 14 300)	−0·69% (−2·07 to 0·98)
	Edentulism	4070 (3510 to 4820)	4090 (3550 to 4820)	0·47% (−0·70 to 1·62)
	Other oral disorders	1860 (1780 to 1930)	1860 (1780 to 1930)	−0·01% (−0·04 to 0·01)
Lip and oral cavity cancer		4·37 (3·78 to 5·08)	4·73 (4·03 to 5·56)	8·35% (0·13 to 16·32)
Orofacial clefts		53·0 (43·3 to 64·6)	54·2 (44·1 to 66·3)	2·25% (0·77 to 3·83)

Main oral conditions include untreated caries, severe periodontitis, edentulism, and other oral disorders only. Prevalent cases of main oral conditions do not match the sum of prevalent cases of individual conditions because some conditions coexist within the same individuals. Data are shown as age-standardised prevalence for all oral conditions, except for lip and oral cavity cancer, which is shown as age-standardised incidence. Cases are presented to three significant figures and percentage changes are reported to two decimal places. UI=uncertainty interval.

Globally, prevalent cases of untreated caries in deciduous teeth decreased by 4·56% (95% UI –8·51 to –0·43) between 2019 and 2023, from 533 million (444 to 631) to 509 million (428 to 590; table 2). This change was driven by reductions in south Asia (–11·67% [–20·05 to –2·76]), central Europe, eastern Europe, and central Asia (–7·06% [–8·54 to –5·50]), north Africa and the Middle East (–4·6% [–7·00 to –1·84]) and southeast Asia, east Asia, and Oceania (–4·24% [–6·85 to –1·74]; figure 1; appendix table S1). Cases increased by 4·79% (2·24–7·33) in the high-income super-region.

The global age-standardised prevalence of untreated caries in deciduous teeth remained unchanged (–3·68% [95% UI –7·78 to 0·48]), affecting 7310 (6160 to 8450) people per 100 000 population in 2023 (table 3). Reductions in age-standardised prevalence were observed in south Asia (–9·09% [–17·62 to –0·08]) and central Europe, eastern Europe, and central Asia (–2·03% [–3·59 to –0·41]) whereas an increase was observed in the high-income super-region (9·21% [6·60 to 11·89]; figure 2; appendix table S2).

Globally, prevalent cases of untreated caries in permanent teeth increased by 2·78% (95% UI 2·19–3·41) between 2019 and 2023, from 2·20 billion (1·92–2·56) to 2·26 billion (1·98–2·63; table 2). This change was driven by increases in sub-Saharan Africa (11·07% [9·96–12·22]), north Africa and the Middle East (5·92% [4·01–7·79]), south Asia (2·65% [1·13–4·33]) and the high-income super-region (2·06% [0·75–3·32]; figure 1; appendix table S1).

The global age-standardised prevalence of untreated caries in permanent teeth decreased by –1·99% (95% UI –2·49 to –1·40), from 27 600 (24 100 to 32 200) per 100 000 population in 2019 to 27 100 (23 600 to 31 600) per 100 000 population in 2023 (table 3). Reductions in age-standardised prevalence were observed in south Asia (–3·77% [–5·18 to –2·33]), Latin America and the Caribbean (–3·54% [–5·04 to –2·36]), southeast Asia, east Asia, and Oceania (–2·32% [–3·59 to –1·06]), and sub-Saharan Africa (–1·51% [–2·58 to –0·38]; figure 2; appendix table S2).

Globally, prevalent cases of severe periodontitis increased by 5·73% (95% UI 4·57–6·91) between 2019 and 2023, from 1·03 billion (0·879–1·19) to 1·09 billion (0·915–1·26; table 2). Cases increased in sub-Saharan Africa (11·49% [10·55–12·34]), north Africa and the Middle East (8·75% [7·48–10·33]), South Asia (7·92% [6·55–9·30]), southeast Asia, east Asia, and Oceania (4·82% [3·68–5·98]), and the high-income super-region (1·47% [0·21–2·51]; figure 1; appendix table S1).

The global age-standardised prevalence of severe periodontitis remained unchanged (–0·69% [95% UI –2·07 to 0·98]), at 12 400 (10 400 to 14 300) per 100 000 population in 2023 (table 3). Reductions were observed across all super-regions, ranging from –2·17% (–2·85 to –1·44) in Latin America and the Caribbean to –1·09% (–1·62 to –0·46) in south Asia, except in central Europe, eastern Europe, and central Asia, and in southeast Asia, east Asia, and Oceania (figure 2; appendix table S2).

Globally, prevalent cases of edentulism increased by 11·09% (95% UI 9·64–12·93) between 2019 and 2023, from 336 million (289–397) to 373 million (324–440; table 2). Cases increased in all super-regions, from 6·97% (5·10–9·32) in central Europe, eastern Europe, and central Asia to 13·85% (12·31–15·11) in north Africa and the Middle East (figure 1; appendix table S1).

The global age-standardised prevalence of edentulism remained unchanged (0·47% [95% UI –0·70 to 1·62]), at 4090 (3550 to 4820) per 100 000 population in 2023 (table 3). Reductions were observed in south Asia (–1·75% [–2·59 to –0·84]), sub-Saharan Africa (–1·29% [–2·07 to –0·54]), and southeast Asia, east Asia, and Oceania (–0·88% [–1·69 to –0·12]) while an increase was observed in central Europe, eastern Europe, and central Asia (2·43% [1·47 to 3·42]; figure 2; appendix table S2).

Globally, prevalent cases of other oral disorders increased by 4·56% (95% UI 4·42–4·69) between 2019 and 2023, from 148 million (142–154) to 154 million (149–160; table 2). Cases increased across all super-regions, ranging from 1·68% (1·59–1·78) in the high-income super-region to 12·02% (11·91–12·14) in sub-Saharan Africa, except for central Europe, eastern Europe, and central Asia, where no meaningful change was observed (figure 1; appendix table S1).

The global age-standardised prevalence of other oral disorders remained unchanged between 2019 and 2023 (–0·01% [95% UI –0·04 to 0·01]), at 1860 (1780 to 1930) per 100 000 population in 2023 (table 3). An increase of 0·05% (0·03–0·07) was observed in the high-income super-region (figure 2; appendix table S2).

Globally, incident cases of lip and oral cavity cancer increased by 18·4% (95% UI 9·76–26·9) between 2019 and 2023, from 0·363 million (0·315–0·422) to 0·430 million (0·367–0·505; table 2). Incident cases increased across all super-regions, ranging from 8·70% (3·41–15·19) in Latin America and the Caribbean to 32·12% (18·25–47·44) in sub-Saharan Africa, except for central Europe, eastern Europe and central Asia, where no meaningful change was observed (figure 1; appendix table S1).

The global age-standardised incidence of lip and oral cavity cancer increased by 8·35% (95% UI 0·13–16·32), from 4·37 (3·78–5·08) per 100 000 population in 2019 to 4·73 (4·03–5·56) per 100 000 population in 2023 (table 3). This change was largely driven by increases in sub-Saharan Africa (14·86% [1·19–32·45]) and south Asia (14·33% [0·47–29·05]; figure 2; appendix table S2).

Globally, prevalent cases of orofacial clefts increased by 5·14% (95% UI 3·63–6·78) between 2019 and 2023, from 4·05 million (3·30–4·94) to 4·26 million (3·46–5·20; table 2). Cases increased in sub-Saharan Africa (10·54% [8·53–12·88]), south Asia (6·19% [3·58–8·59]), north Africa and the Middle East (5·19% [2·43–7·92]), and southeast Asia, east Asia, and Oceania (2·81% [0·61–4·63]; figure 1; appendix table S1).

The global age-standardised prevalence of orofacial clefts increased by 2·25% (95% UI 0·77–3·83), from 53·0 (43·3–64·6) per 100 000 population in 2019 to 54·2 (44·1–66·3) per 100 000 population in 2023 (table 3). Increases were observed in central Europe, eastern Europe, and central Asia (3·44% [1·20–5·56]) and southeast Asia, east Asia and Oceania (2·61% [0·42–4·75]; figure 2; appendix table S2).

## Discussion

Coinciding with the first planned assessment of GOHAP targets, this analysis shows a modest improvement in the combined prevalence of main oral conditions that masks a still-expanding global burden. Against the GOHAP target of a 10% reduction by 2030, the combined age-standardised prevalence of main oral conditions fell by –1·55% between 2019 and 2023, yet cases rose by 3·07% to 3·73 billion, signalling that population growth and ageing are outpacing current prevention efforts and oral health-care service capacity.^18^

Although most complementary targets are off track, it is important to recognise that they are not equally responsive to short-term policy change or health-system reorganisation. Thus, expectations for measurable progress should differ accordingly. Only untreated caries in permanent teeth showed a measurable decline (−1·99%), while lip and oral cavity cancers (8·35%) and orofacial clefts (2·25%) showed noticeable increases. This pattern is particularly salient given that, although they are less prevalent than the main oral conditions, lip and oral cavity cancer and orofacial clefts are the only oral conditions in GBD directly associated with mortality. Although these changes might reflect true epidemiological shifts, they could also be the result of improved surveillance, registry coverage, or reporting. This is particularly important for cancers and congenital anomalies, for which ascertainment sensitivity varies across regions.

Progress towards the GOHAP targets was found to be highly uneven across regions, underscoring the need for region-specific policy responses rather than one-size-fits-all approaches. South Asia had the largest decline in the overarching target (−3·34%), a pace that need to be sustained to meet the 2030 targets, alongside reductions in several complementary indicators (ie, untreated caries in deciduous and permanent teeth, severe periodontitis, and edentulism). However, the incidence of lip and oral cavity cancer in south Asia increased markedly (14·33%). The GBD cause list includes heterogeneous oral conditions with distinct aetiologies and management needs, complicating the identification of shared intervention targets or prioritisation based solely on caseload.

Sub-Saharan Africa showed the largest increase in cases of main oral conditions (9·51%), alongside increasing incidence of lip and oral cavity cancer (14·86%), highlighting the need for accelerated prevention, workforce and service expansion, and equity-focused financing in this super-region. Accelerating and upscaling evidence-based interventions to reduce tobacco use, betel nut consumption, and harmful alcohol use are therefore imperative in both sub-Saharan Africa and south Asia.^6^

The high-income super-region showed an increase in the overarching target (1·24%) and a marked increase in untreated caries in deciduous teeth (9·21%), suggesting that greater resource availability does not necessarily translate into improved outcomes. Persistent structural and behavioural barriers could contribute to limited progress, including the influence of commercial determinants, increasingly cariogenic dietary environments, cessation of community water fluoridation, and gaps in preventive coverage for early childhood caries. These patterns suggest the potential value of strengthening regulatory action on unhealthy products, aligning fiscal and food policies, and improving access to evidence-based preventive services in early life.^2^, ^9^, ^19^

We emphasise that the modest improvements in the combined age-standardised prevalence are offset by demographic growth, leading to an overall increase in the absolute burden of the main oral conditions. Age-standardised prevalence should not be interpreted in isolation; it needs to be considered alongside changes in case numbers, as reliance on a single metric could create a misleading perception of progress and foster complacency in policy and practice. The growing caseloads indicate that, by 2023, health-care systems were managing a larger population with oral conditions requiring treatment than in 2019. These trends have implications for workforce planning and financing of universal oral health-care coverage.

Our findings reinforce the policy case for integrated oral and non-communicable disease action encompassing prevention, early detection, and timely access to treatment and rehabilitative care.^2^, ^9^, ^19^, ^20^, ^21^ There is a need to accelerate integrated preventive strategies to shift disease trajectories sufficiently by 2030. Priority areas include strengthening tobacco and alcohol control to reduce lip and oral cavity cancer, severe periodontitis, and orofacial clefts (through reduced consumption during pregnancy), alongside implementing policies to reduce sugar intake and prevent dental caries.^7^, ^19^, ^22^ However, prevention alone is insufficient to address the large proportion of the global population with an unmet need for oral health care. Countries must expand essential oral health-care packages, including pain management, minimally invasive treatment, and affordable rehabilitative care.^8^, ^9^ Worsening indicators in specific conditions demand targeted, context-specific responses. The increasing incidence of lip and oral cavity cancer calls for robust early detection and referral systems in high-growth regions (eg, sub-Saharan Africa), while increasing orofacial clefts underscore the need for strengthened congenital anomaly surveillance and equitable access to reparative and long-term rehabilitative services.^20^

Caution is warranted when interpreting changes of a small magnitude over short time intervals, as oral conditions take time to develop. Relatively wide uncertainty intervals, often spanning zero, suggest that observed changes might reflect statistical uncertainty rather than true epidemiological shifts. Accordingly, these estimates are better understood as indicative of broad patterns (or signals), with more robust conclusions requiring longer-term trends and consistent directional changes.

Limitations in the quality and availability of primary data sources in some super-regions or time periods remain a recurring challenge, underscoring the need to strengthen data collection systems.^23^, ^24^ The COVID-19 pandemic disrupted the implementation of epidemiological surveys worldwide, potentially delaying the availability of data for more recent years. This was particularly true for the main oral conditions. As a result, the observed trends relied largely on historical data inputs, with few primary data sources available for 2020–23. Additionally, heterogeneity in data collection methods and case definitions across input sources requires the use of cross-walking and meta-regression adjustments to improve data harmonisation and expand data coverage. As a result, the observed temporal trends might reflect both true changes in morbidity and detection artefacts.

Beyond this, the quantification of uncertainty around estimates is a key feature of GBD. Fully accounting for the range of uncertainties inherent in burden estimation remains an ongoing challenge, especially since uncertainty and statistical variation cannot be fully eliminated. Uncertainty arising from data inputs, data harmonisation processes, and meta-regression modelling results in broad 95% UIs, which complicates the interpretation of trends over time and comparison across locations.

It is also important to note that the 2019 prevalence estimates reported in the *WHO Global Oral Health Status Report*^25^ (baseline assessment) were derived from UN population data, whereas GBD relies on internally modelled population estimates. Although methodological differences between these approaches can lead to variation in prevalence estimates, these differences were relatively small for 2019.^12^ Although GBD estimates are increasingly being used to inform policy and monitor progress at a global level, they should be interpreted with caution. They are modelled outputs that integrate heterogeneous data sources, and are therefore subject to assumptions, data gaps, and methodological adjustments. As such, they are better understood as tools for benchmarking and broad trend analysis rather than precise measures of programme performance.

Turning to future directions, with each GBD iteration, we aim to include additional oral conditions and expand geographical coverage, while continuously integrating new input sources, refining methods for data harmonisation, and enhancing methods to quantify uncertainty in estimates. These ongoing efforts will improve the accuracy of estimates for the upcoming assessments of GOHAP targets scheduled for 2026 and 2030. Collaboration with the WHO Oral Health programme is helping to advance methods for the surveillance and monitoring of oral conditions.^26^, ^27^, ^28^ A key outcome of this work has been the alignment of case definitions for untreated caries and edentulism in the STEPS (WHO STEPwise approach to non-communicable disease risk factor surveillance) survey oral health module with GBD definitions. Over time, this approach will create a valuable source of primary sources for inclusion in the GBD dataset.

Another important area of work is the quantification of the burden of main oral conditions attributable to key behavioural factors for non-communicable diseases, including smoking, sugar intake, and alcohol consumption. This process involves systematically searching for and appraising evidence that quantifies the association between each behavioural factor and oral conditions for inclusion in the GBD database of metabolic, behavioural, and environmental risk–outcome pairs used in modelling. These estimates allow us to evaluate alternative scenarios that can inform policy decisions.

At the estimated 2019–23 rate of change, meeting the 2030 GOHAP target of a 10% reduction in oral disease burden by 2030 appears to be unlikely globally unless rates of improvement accelerate. The growing caseloads indicate that, by 2023, health systems were managing a larger population with oral conditions requiring oral health-care services than in 2019. Some super-regions, such as south Asia, might be able to meet their targets, but this alone is unlikely to substantially influence global estimates. Meeting the 2030 targets will require both faster risk reduction and service expansion to keep pace with population growth and ageing.

### GBD 2023 Oral Disorders Collaborators

### Affiliations

### Contributors

### Data sharing

To download citations and metadata for the input data sources used in the GBD 2023 analyses presented in this study, please visit the GBD 2023 Sources Tool (https://ghdx.healthdata.org/gbd-2023/sources).

## Declaration of interests

S Bhaskar reports grants or contracts from the Japan Society for the Promotion of Science (JSPS), Grant-in-Aid for Scientific Research (KAKENHI), Grant ID 23KF0126; and JSPS and the Australian Academy of Science, JSPS International Fellowship, Grant ID P23712; payment or honoraria from the Japan Stroke Society, Japan, for an invited keynote presentation at the 50th Annual Meeting of the Japan Stroke Society, Osaka; and Semey Medical University, Kazakhstan, for an invited professorship and teaching course in neurocardiovascular therapy; support for attending meetings or travel, or both, from the Japan Stroke Society, Japan; travel award for invited keynote presentation at the 50th Annual Meeting of the Japan Stroke Society, Osaka, and Asialink, The University of Melbourne; travel support for opening invited keynote at the 31st Asialink Leaders Foundation Week Program 2026, Melbourne; leadership or fiduciary role in Rotary District 9675, Sydney, Australia (District Chair, Diversity, Equity, Inclusion & Belonging); Global Health & Migration Hub Community, Global Health Hub Germany, Berlin, Germany (Chair, Founding Member and Manager); *PLOS One*, *BMC Neurology*, *Frontiers in Neurology*, *Frontiers in Stroke*, *Frontiers in Public Health*, *Journal of Aging Research*, *Neurology International*, *Diagnostics*, and *BMC Medical Research Methodology* (Editorial Board Member); College of Reviewers, Canadian Institutes of Health Research (CIHR), Government of Canada (Member); World Headache Society, Bengaluru, India (Director of Research); Cariplo Foundation, Milan, Italy (Expert Adviser/Reviewer); National Cerebral and Cardiovascular Center, Department of Neurology, Division of Cerebrovascular Medicine and Neurology, Suita, Osaka, Japan (Visiting Director); Cardiff University Biobank, Cardiff, UK (Member, Scientific Review Committee); Rotary Reconciliation Action Plan (Chair); and Japan Connect, Osaka, Japan (Healthcare and Medical Adviser) outside the submitted work. B Goulart reports stock or stock options in Badger Meter Inc, British American Tobacco, and Altria Group (payments made to B Goulart) outside the submitted work. I Ilic reports support for the present manuscript from the Ministry of Science, Technological Development and Innovation of the Republic of Serbia (grant number 451-03-137/2025-03/200110). K Krishan acknowledges non-financial support from the UGC Centre of Advanced Study, CAS II, awarded to the Department of Anthropology, and RUSA 2.O grant awarded by the Indian Ministry of Education to Panjab University, Chandigarh, India, outside the submitted work. G La Rosa reports grants or contracts from the Knowledge•Action•Change Tobacco Harm Reduction Programme, an independent public health organisation based in the UK and funded by Global Action to End Smoking, a US-based independent non-profit 501(c)(3) grant-making organization (G La Rosa was awarded a 2026 scholarship for a project focused on oral health; G La Rosa previously received funding through the same programme in 2024–25 for an additional oral health-related project; the content of the present paper is unrelated to those projects), outside the submitted work. M-C Li reports grants or contracts from the National Science and Technology Council, Taiwan (NSTC 113-2314-B-003-002) and the Higher Education Sprout Project of National Taiwan Normal University (M-C Li was supported by these grants); and leadership or fiduciary role as Technical Editor, Journal of the American Heart Association, outside the submitted work. K S-K Ma reports support for the present manuscript and grants or contracts from the International Team for Implantology. S Masi reports grants or contracts from Italian Ministry of University, Italian National Research Council, and Italian Ministry of Health (institutional grants for the conduction of research projects); consulting fees from Servier (personal payments for consultancy activities); payment or honoraria from Servier (personal honoraria for lectures, presentations, speakers bureaus, manuscript writing, and educational events); support for attending meetings or travel, or both, from Servier (personal support for attending congresses); participation on a data safety monitoring board or advisory board from Servier (personal payments for participation on advisory boards); and leadership or fiduciary role in European Society of Hypertension, Working Group of Hypertension and the Heart (Vice-President, unpaid), and Italian Society of Internal Medicine (President of the Tuscany-Umbria section, unpaid) outside the submitted work. R Moreira reports grants or contracts from CNPq (National Council for Scientific and Technological Development), Research Productivity Scholarship, registration number 308986/2025-3 (R Moreira is a research productivity fellow; R Moreira would like to mention the acknowledgment of this grant and its number in the Article, as it is also related to research using GBD), outside the submitted work. B Oancea reports support for the present manuscript from the Romanian National Research, Development, and Innovation Plan 2022–27, project PNRR/2022/C9/MCID/I8 number 1672173292, contract number 760231; and PNRR/2022/C9/MCID/I8 project 842027778, contract number 760096. S Panda reports support for the present manuscript from Siksha ‘O’ Anusandhan (Deemed to be University; payment of salary); grants or contracts from Central Council for Research in Homoeopathy (CCRH), Government of India (file number 17-59/2023-24, as Co-Investigator); and Indian National Science Academy (INSA), Government of India (file number HS/RC/393, as Principal Investigator) outside the submitted work. G D Panos reports payment or honoraria from Thea, Roche, and Bayer (payments made to their institution); and support for attending meetings or travel, or both, from Thea, Roche, and Bayer (payments made to the organising agency of the event) outside the submitted work. P Pietkiewicz reports payment or honoraria from Heine Optotechnik, Dermatological Society of South Africa, Fotofinder Systems, Akademia Dermatoskopii, Polpharma, Galderma, Melanoma Society South Africa, and Primary Care Dermatological Society (UK); leadership or fiduciary role in Polish Dermatoscopy Group (President) and EADV Honors and Award Committee (Member); receipt of equipment, materials, drugs, medical writing, gifts, or other services from Heine Optotechnik; and other financial or non-financial interests in SkinSkan (shareholder), outside the submitted work. A Riad reports support for the present manuscript from the Czech Health Research Council (project number: NW26J-09-00120). S Saleem reports support for the present manuscript from Health Services Academy, Islamabad, Pakistan; and payment or honoraria for lectures, presentations, speakers bureaus, manuscript writing, or educational events from Health Services Academy, Islamabad, Pakistan, outside the submitted work. B Schaarschmidt reports grants or contracts from Else Kröner-Fresenius Foundation (research grant), Deutsche Forschungsgemeinschaft (research grant), and PharmaCept (research grant); payment or honoraria from AstraZeneca (speaker), Boston Scientific (speaker/proctor), and MedMile (speaker); and support for attending meetings or travel, or both, from Bayer (travel grant), outside the submitted work. L Silva reports grants or contracts from SPRINT – Sport Physical Activity and Health Research & Innovation Center, Portugal, FCT – Fundação para a Ciência e a Tecnologia (Portuguese Foundation for Science and Technology), grant numbers UID/06185/2025, UID/PRR/06185/2025, and UID/PRR2/06185/2025; and RISE-Health, Department of Medical Sciences, Faculty of Health Sciences, University of Beira Interior, Covilhã, Portugal, outside the submitted work. J A Singh reports consulting fees from ROMTech, Atheneum, Clearview Healthcare Partners, Yale, Hulio, Horizon Pharmaceuticals/DINORA, ANI/Exeltis USA, SOBI Pharmaceuticals, Frictionless Solutions, Schipher, Crealta/Horizon, Medisys, Fidia, PK Med, Two Labs, Adept Field Solutions, Clinical Care Options, Putnam Associates, Focus Forward, Navigant Consulting, Spherix, MedIQ, Jupiter Life Science, UBM, Trio Health, Medscape, WebMD, and Practice Point Communications; the US National Institutes of Health; and the American College of Rheumatology (consultant fees paid to J A Singh for each entity); payment or honoraria from Simply Speaking (speakers bureau; J A Singh previously served as a consultant and consultant fees were paid to J A Singh); support for attending meetings or travel, or both, as past steering committee member of OMERACT (J A Singh previously received support from the organisation to attend their meeting every 2 years); participation on a data safety monitoring board or advisory board with the US Food & Drug Administration Arthritis Advisory Committee (J A Singh served previously as a member; no financial support); leadership or fiduciary role as past steering committee member of OMERACT, an international organisation that develops measures for clinical trials and receives arm's length funding from 12 pharmaceutical companies (J A Singh previously received support from the organisation to attend their meeting every 2 years); and stock or stock options in aTyr Pharmaceuticals, Atai Life Sciences, Kintara Therapeutics, Intelligent Biosolutions, Acumen Pharmaceutical, TPT Global Tech, Vaxart Pharmaceuticals, Aytu BioPharma, Adaptimmune Therapeutics, GeoVax Labs, Pieris Pharmaceuticals, Enzolytics, Seres Therapeutics, Tonix Pharmaceuticals Holding Corp, Aebona Pharmaceuticals, and Charlotte's Web Holdings (J A Singh owns stock options; J A Singh previously owned stock options in Amarin, Viking, and Moderna Pharmaceuticals), outside the submitted work. S Singh reports grants or contracts from the DTRA Biological Threat Reduction Program (HDTRA1-21-1–0036), Indian Council of Medical Research (VIR/12/2022/ECD-1), and Institute of Eminence (IoE), Banaras Hindu University (seed grant and transdisciplinary research grant; the laboratory of S Singh was partially supported by these grants), outside the submitted work. J Zhao reports support for the present manuscript from Fundamental Research Funds for the Central Universities (2024BSSXM20); Zhejiang University Qiushi Flying Eagle Program; Key Laboratory of Integrated Care for Geriatric Chronic Diseases, Kunming Medical University, School of Nursing, and the Yunnan Provincial Education Department (grant number 2024HTHLYB05); and Doctoral Student Special Program of the China Association for Science and Technology (CAST) Young Talent Support Project. M Zielińska reports financial or non-financial interests as an Alexion AstraZeneca Rare Disease employee, outside the submitted work. All other authors declare no competing interests.
