# Supplementary material for: Global and regional progress towards reduced burden of oral conditions between 2019 and 2023: a systematic analysis for the Global Burden of Disease Study 2023
Source: Lancet Public Health. 2026 Aug 18;11(9):e630–41. doi: 10.1016/S2468-2667(26)00143-X (PMC13501145; doi:10.1016/S2468-2667(26)00143-X)
Supplement: Supplementary file 1 [file mmc1.pdf]

THE LANCET
Public Health

Supplementary appendix

This appendix formed part of the original submission and has been peer reviewed. We post it as supplied by the authors.

Supplement to: GBD 2023 Oral Disorders Collaborators. Global and regional progress towards reduced burden of oral conditions between 2019 and 2023: a systematic analysis for the Global Burden of Disease Study 2023. *Lancet Public Health* 2026; **11:** e630–41.

Appendix: Supplemental materials to “Global and regional progress toward reduced burden of oral conditions: A systematic analysis for the Global Burden of Disease Study 2023”

GBD Oral Disorders Collaborators

This appendix provides further methodological details and supplemental findings for “Global and regional progress toward reduced burden of oral conditions: A systematic analysis for the Global Burden of Disease Study 2023”. The first section presents the methods relevant to oral conditions and is intended primarily for technical readers. The full GBD methodology can be read in the Appendix Methods of the GBD 2023 Disease and Injury and Risk Factor Collaborators (2025) paper.

Table of Contents

I. Oral conditions 4
- Input data 4

II. Edentulism 5
- Flowchart 5
- Key modelling assumptions: 5
- Case definition 5
- Input data and data processing 5
  - Age-sex splitting 6
  - Crosswalks in meta-regression—Bayesian, regularised, trimmed (MR-BRT) 6
- Modelling strategy 6
- Severity distributions and disability weights 7

III. Untreated caries in deciduous and permanent teeth 8
- Flowchart 8
- Key modelling assumptions: 8
- Separate estimates of caries in deciduous teeth and caries in permanent teeth 8
- Case definition 9
- Input data and data processing 9
  - Converting lifetime to current prevalence 10
  - Calculation of incidence from dmft/DMFT increment 10
  - Age and sex splitting 10
  - Crosswalks in MR-BRT 10
- Modelling strategy 12
  - DisMod model development 12
  - Correction for edentulism 13
  - Severity distributions and disability weights 13
- References 15

IV. Severe periodontitis 16
- Flowchart 16
- Key modelling assumptions: 16

Case definition 16
Input data 17
Age-sex splitting 18
Crosswalks in MR-BRT 18
.... 19
Modelling strategy 19
Correction for edentulism 19
Severity distributions and disability weights 19
References 20
V. Other oral disorders 21
VI. Lip and oral cavity cancer 22
Flowcharts 22
Key modelling assumptions: 23
Case definition 23
Input data 24
Modelling strategy 25
MIR data processing 25
MIR data modelling 25
Mortality estimation 26
Incidence estimation 27
Prevalence estimation 27
Disability estimation 28
YLD estimation 29
References 29
VII. Orofacial clefts 31
Flowchart 31
Registry flow chart 32
Key modelling assumptions: 32
Input data 33
Data processing 34
Age-sex splitting 34
Crosswalks in MR-BRT 34
Determining outliers and data thresholds 34
Modelling strategy 34
Overview 34
Location-level covariates 35
Post-model processing 35
Assigning health states and sequelae for long-term outcomes 35
Orofacial clefts 35
Case definition and associated health states 35
Crosswalks 36
Modelling strategy 36

GATHER checklist 38

Figure S1. World map of GBD super-regions and their constituent geographies. The grouping of countries reflects standard GBD analytic super-regions, not policy or WHO regions. 40

Table S1. Prevalent or incident cases in 2019 and 2023 and percent changes over 2019-2023 for oral conditions, by super-regions. 41

Table S2. Age-standardized prevalence or incidence in 2019 and 2023 and percent changes over 2019-2023 for oral conditions, by super-regions.. 44

GBD 2023 Oral Disorders Collaborators.. 47

Affiliations.. 50

Authors' Contributions 66

Providing data or critical feedback on data sources.. 66

Developing methods or computational machinery 68

Providing critical feedback on methods or results... 68

Drafting the work or revising it critically for important intellectual content...................... 71

Managing the estimation or publications process 74

I. Oral conditions

This document describes the non-fatal disease burden modelling process for GBD 2023 for each of edentulism, caries of deciduous teeth, caries of permanent teeth, chronic periodontal disease, and other oral disorders.

Input data

Data seeking and systematic literature reviews were completed for all oral disorders together, given the overlap in data types and data sources that inform the models. An initial literature review was done by the Expert Group for GBD 2010 in PubMed, Embase, Latin American and Caribbean Health Sciences (LILACS), and Scientific Electronic Library Online (SciELO), including published articles as well as the results of national and subnational reports. An updated systematic review was last completed on February 11, 2018, for GBD 2017 in PubMed and Embase. The search strings used are below:

> **PubMed:** ((((Deciduous caries[tiab]) OR (milk caries[tiab]) OR (baby caries[tiab]) OR (caries[tiab]) OR (dental health[tiab]) OR (oral health[tiab])) OR ((Permanent caries[tiab]) OR (caries prevalence[tiab]) OR (dental health[Title/Abstract]) OR (oral health[tiab])) OR ((Periodontal disease[tiab]) OR (periodontitis[tiab]) OR (periodontal[tiab])) OR ((Edentulism[tiab]) OR (edentulous[tiab]) OR (endentulousness[tiab]) OR (severe tooth loss[tiab]) OR (total tooth loss[tiab]) OR (complete tooth loss[tiab]))) AND ((prevalence[tiab]) OR (incidence[tiab])) AND (2013/06/01[PDat] : 2016/12/31[PDat]))

> **Embase:** 'deciduous caries':ab,ti OR 'milk caries':ab,ti OR 'baby caries':ab,ti OR caries:ab,ti OR 'permanent caries':ab,ti OR 'caries prevalence':ab,ti OR 'dental health':ab,ti OR 'oral health':ab,ti OR 'periodontal disease':ab,ti OR periodontitis:ab,ti OR periodontal:ab,ti OR edentulism:ab,ti OR edentulous:ab,ti OR endentulousness:ab,ti OR 'severe tooth loss':ab,ti OR 'total tooth loss':ab,ti OR 'complete tooth loss':ab,ti AND (prevalence:ab,ti OR incidence :ab,ti) AND [2008-2016]/py AND [humans]/lim AND [embase]/lim NOT [medline]/lim

For GBD 2019, we completed a targeted systematic review of LILACS and SciELO, focusing first on articles from the most recent period, from 2014 to 2018, which were subject to full-text screening. The search used for LILACS and SciELO was the same:

> **LILACS/SciELO:** "(deciduous caries OR milk caries OR baby caries OR caries OR dental health OR oral health OR permanent caries OR caries prevalence OR periodontal disease OR periodontitis OR periodontal OR edentulism OR edentulous OR edentulousness OR complete tooth loss OR tooth loss OR toothloss OR number of teeth OR dentate OR edentate) AND (prevalence OR incidence OR survey OR epidemiology)".

A total of 1696 citations were identified after deduplication, 147 were selected for full text review, and

77 new sources were extracted from the following countries: Argentina (1), Brazil (47), Chile (5), Colombia (5), Cuba (5), Ecuador (1), El Salvador (1), Honduras (1), Mexico (5), Peru (5), and Venezuela (1).

For GBD 2021, we reviewed the Global Health Data Exchange (https://ghdx.healthdata.org) for oral health surveys and national reports with oral epidemiology data. A total of 105 new sources were identified and extracted for inclusion in GBD 2021 models. We eliminated many datapoints to avoid repetition in the dataset, while striving to maintain as much data detail as possible. Redundancy tended to arise in three data descriptors: age, gender, and urbanicity. Our order of preference for maintaining detail was age, followed by gender, then urbanicity. Additionally, many of the studies presented dmft or DMFT (decayed-missing-filled teeth) scores, which represent lifetime prevalence and were often described as "caries experience". For the purposes of measuring the burden of disability from dental caries, we considered only data on current prevalence to be relevant, and thus converted lifetime prevalence data to current prevalence and incidence where possible. There were no new data sources added for GBD 2023.

II. Edentulism

Flowchart

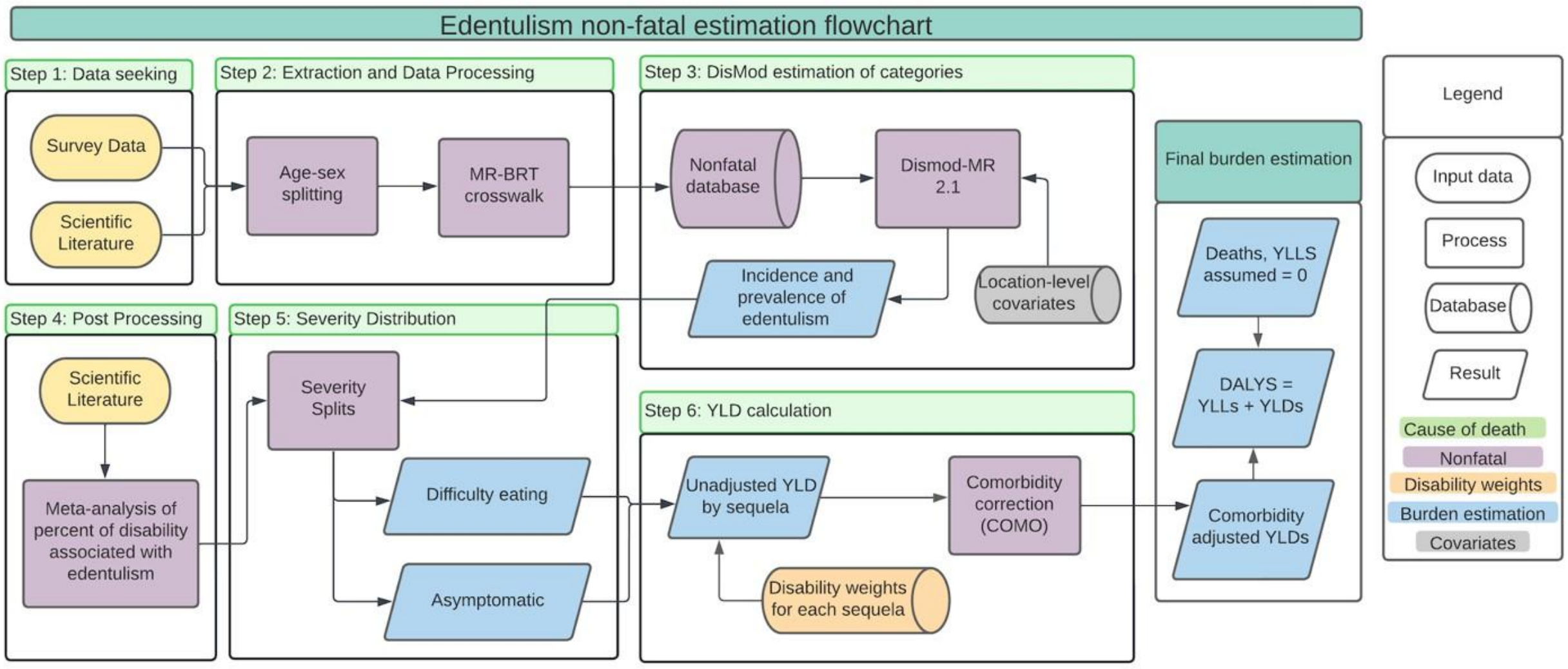

Key modelling assumptions:

- Self-reported edentulism was assumed to be equivalent to edentulism identified through clinical oral examination; therefore, no cross-walking between data sources was performed.
- Both the Incidence and prevalence of edentulism were set to zero during childhood. Incidence was permitted to increase from age 15 years onwards while remission was set to zero for all ages. Mortality and relative risk were fixed at zero for individuals younger than 30 years.
- Log-transformed lag-distributed income and the log-transformed age-standardised summary exposure value (SEV) for cardiovascular disease were included as covariates.

Case definition

The case definition of edentulism includes any individual with zero remaining permanent teeth; toothlessness of infancy is not included. The assessment of this disease includes quantification of the prevalence of the disease as well as estimation of the major sequelae: asymptomatic toothlessness and symptomatic toothlessness leading to "great difficulty in eating meat, fruits, and vegetables". A small body of evidence has begun to emerge that implicates edentulousness as predisposing individuals to increased risk for ischaemic cardiovascular events, including myocardial infarction and stroke. These data are sparse but have been included in models estimating the excess mortality of those with complete tooth loss. Given that the association is believed to be ecological rather than causal, however, edentulism has not been estimated as an underlying cause of death, and it is not included in the risk factor analysis for cardiovascular diseases.

Input data and data processing

Details of the systematic literature reviews appear earlier in this write-up. In addition to published studies, we also utilised self-report data on toothlessness from World Health Survey (WHS) for 47 countries as well as a number of national oral health surveys identified through the Global Health Data Exchange. The number of primary data sources for edentulism by super-region and year is shown in Table 1.

Table 1. Number of data sources for edentulism

Period	**Central Europe, Eastern Europe, and Central Asia**	**High-income**	**Latin America and the Caribbean**	**North Africa and Middle East**	**South Asia**	**Southeast Asia, East Asia, and Oceania**	**Sub-Saharan Africa**
<1990	0	124	0	0	0	9	2

1990-1994	0	34	0	0	0	5	0
1995-1999	1	37	0	0	0	3	1
2000-2004	18	71	17	4	6	14	24
2005-2009	19	65	29	0	6	18	13
2010-2014	11	50	39	3	8	7	4
2015-2019	5	8	2	0	1	2	3
2020-2023	0	0	0	0	0	0	0

Age-sex splitting

The first step of data processing was age splitting. For any datum that did not entirely fit within a GBD sex or age group, the observation was split to be multiple age-sex-specific datapoints based on the age and sex pattern predicted by previous DisMod-MR 2.1 models.

Crosswalks in meta-regression—Bayesian, regularised, trimmed (MR-BRT)

Prior to GBD 2021, we crosswalked self-reported (ie, WHS) data on toothlessness to the reference definition of oral examination; but at present, we no longer found a statistically significant difference between self-report and oral examination, so no adjustment of self-report data was completed.

Figure 1: Funnel plot showing logit-transformed ratio of edentulism prevalence for alternate (self-report) versus reference (oral examination)

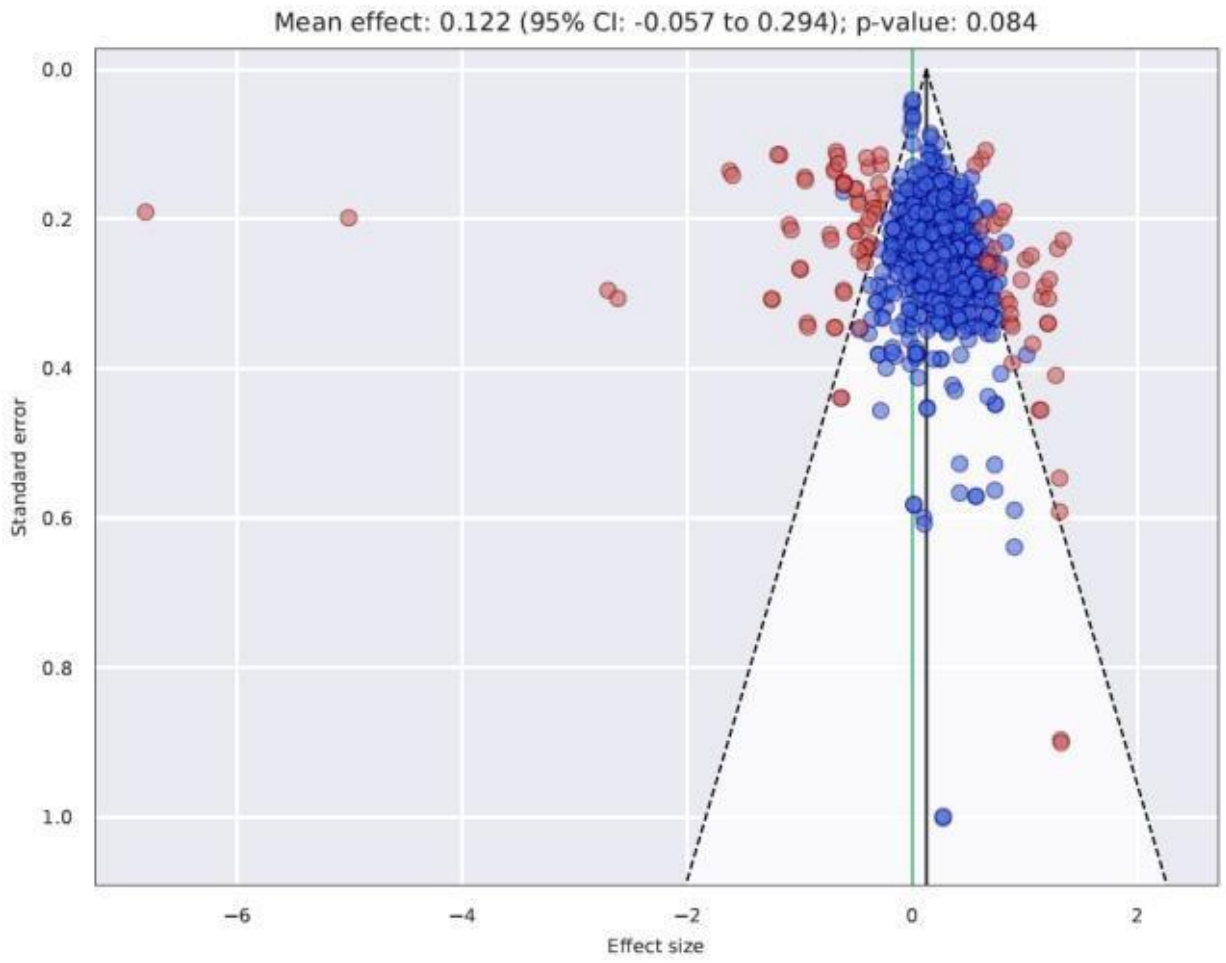

Modelling strategy

Estimates for the prevalence of edentulism were calculated for each location/year/sex/age using DisMod-MR 2.1. As would be expected for an irreversible condition, remission was fixed at zero for all ages. Mortality and relative risk were both fixed at zero before age 30, as any excess cardiovascular events resulting from severe tooth loss would not be expected at younger ages. We also assigned incidence and prevalence to be zero during childhood. Incidence was allowed to rise beginning at age 15, which was chosen based on the age at which the permanent dentition is expected to have fully formed in all individuals. The random effect limits for all locations were bounded at +/- 1.

As mentioned above, the criteria for diagnosis of edentulism are straightforward, and bias in the dataset was considered negligible. Thus, no study-level covariates were used in modelling the prevalence of edentulism. We included three location-level covariates in the model as shown in the table below.

Table 2: Covariate, parameter, beta, and exponentiated beta values for edentulism

Covariate name	Measure	Beta (UI)	Exponentiated beta (UI)
LDI (I$ per capita)	Prevalence	–0.217 (–0.225 to –0.209)	0.805 (0.798–0.811)

Age- and sex-specific SEV for smoking	Prevalence	0.048 (0.002 to 0.123)	1.05 (1.002–1.131)
Age- and sex-specific SEV for high fasting plasma glucose	Prevalence	0.263 (0.068 to 0.466)	1.301 (1.07–1.594)

Models were vetted based on the plausibility of the results, the extent to which estimates fit the data, and the plausibility of the range of estimates across location hierarchies.

Severity distributions and disability weights

The disability weight used for symptomatic toothlessness leading to “great difficulty in eating meats, fruits, and vegetables” is 0.067 (0.045–0.095) as determined by the 2010 GBD disability survey. We considered all those with severe tooth loss and no access to dentures to experience this disability. However, the proportion of those with edentulism and severe tooth loss who have dentures has not been studied extensively.

In order to estimate the proportion of edentulous individuals with no access to dentures, we completed a supplemental literature review of dentures prevalence for GBD 2010. Only six systematic surveys of dentures prevalence were identified, all in high- and middle-income countries. All were completed since 2000. After extracting the data from the studies, we performed linear regressions of denture presence and denture absence against health system access (HSA), a standardised covariate of treatment availability used in many disease estimation models. From the results of the regression, the prevalence of no dentures was calculated for all super-regions. We then completed a population-weighted average of all countries in the super-region based on 2003 populations, the average year of the dentures studies. Uncertainties for the prevalence of dentures were calculated by finding the standard deviation and standard error of the calculated prevalence values.

The estimated prevalence of dentures in each location was used to calculate the proportion of individuals with asymptomatic edentulism and severe tooth loss (ie, those who have access to dentures) and difficulty eating due to edentulism and severe tooth loss (ie, those without access to dentures). This latter sequela was included as a cause of years lost due to disability (YLDs).

III. Untreated caries in deciduous and permanent teeth

Flowchart

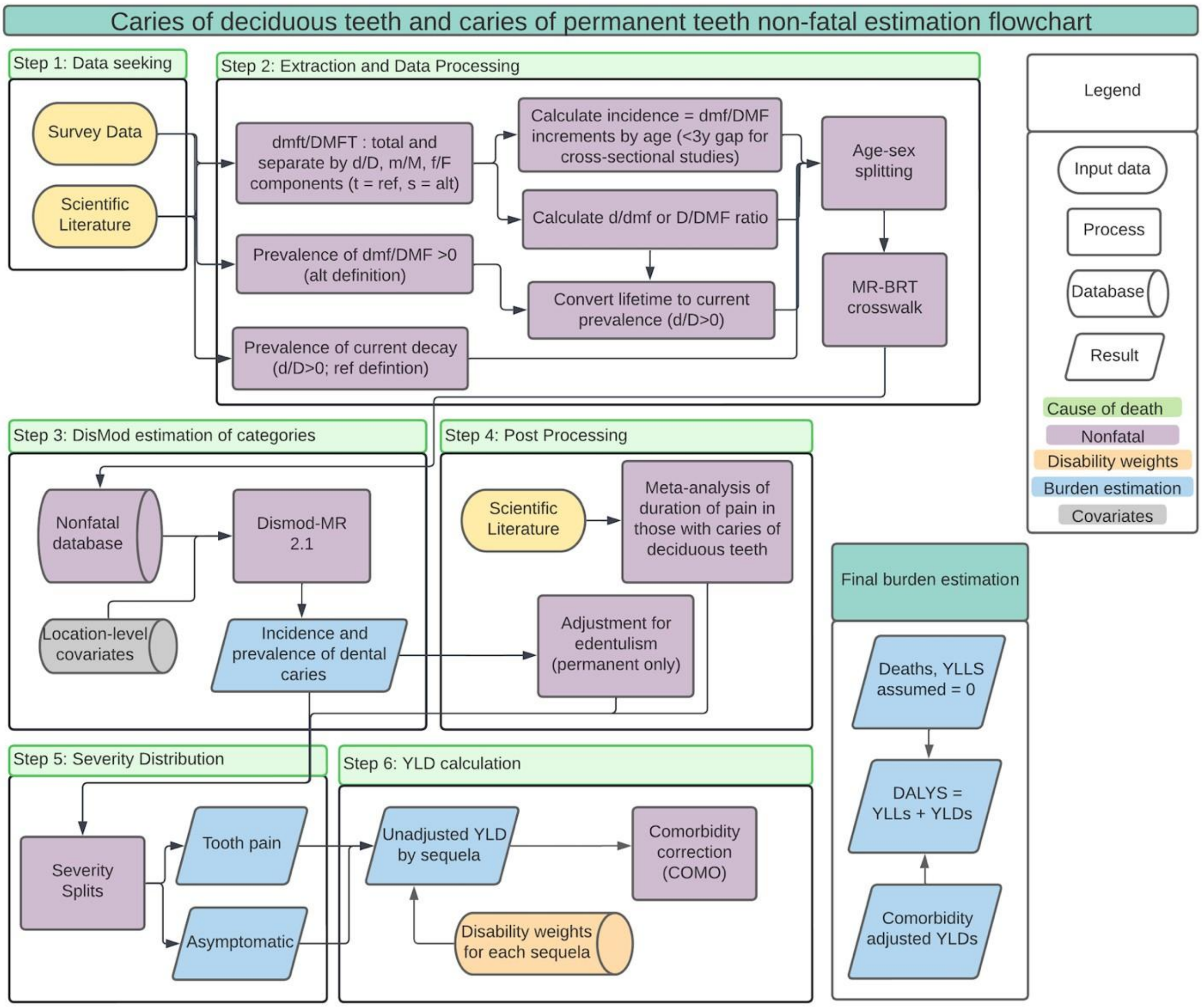

Key modelling assumptions:

- Lifetime caries prevalence (dmft/DMFT>0) was crosswalked to untreated caries prevalence (dt/DT>0) for individuals younger than 20 years.
- Excess mortality was assumed to be zero across all ages. Incident caries was set to zero in infants younger than 1 year and incident deciduous caries was assumed to be zero from age 11 onwards. For untreated caries in permanent teeth, incidence was assumed to be zero in children younger than 5 years.
- Log-transformed lag-distributed income and the log-transformed, age-and sex specific summary exposure value for high consumption of sugar-sweetened beverages were included as covariates.
- Estimates were adjusted for edentulism using GBD estimates of edentulism prevalence.

Separate estimates of caries in deciduous teeth and caries in permanent teeth

The natural histories of deciduous and permanent caries share many similarities, but they also share some important differences. Age patterns of decay in deciduous and permanent dentition are distinct, and duration of a carious lesion in deciduous teeth also tends to be shorter than an untreated episode of caries in permanent teeth. Sugar consumption and feeding with formula are both associated with development of caries in deciduous teeth, while sugar consumption is associated with the development of caries in permanent teeth. Finally, it is unclear whether the gender patterns and regional differences are the same for both caries in deciduous and permanent teeth. For all these reasons, we elected to model untreated caries in deciduous and permanent teeth as separate entities and then add the

estimates together for an overall estimation of the global burden of untreated dental caries. This is the modelling approach which has been taken in each iteration since GBD 2010.

Case definition

The case definition for dental caries is "teeth with unmistakable coronal cavity at dentin level, root cavity in cementum that feel soft or leathery to probing, temporary or permanent restorations, or missing teeth extracted due to a caries lesion". Excluded definitions include crowns with isolated cosmetic defects, stained enamel pits, or fissures without visible cavitation or softening, fluorosis, and abrasion lesions. This definition corresponds to an ICD-9 code of 521.0 and an ICD-10 code of K02.3– K02.9. Most caries are subclinical in the sense that they do not cause symptoms a majority of the time. Once a carious lesion develops, it will occasionally recede without intervention, but often it worsens with time and eventually requires either filling or extraction.

Public health dentists commonly measure dental caries using the dmft/DMFT index, which is an incremental measure of the number of affected teeth and is also a measure of an individual's lifetime prevalence of caries experience. Lowercase letters (dmft) are used for deciduous dentition and uppercase letters (DMFT) for permanent dentition. D is for decayed, M for missing, F for filled, and T for teeth. The maximum dmft score is 20 and the maximum DMFT score is 32. Furthermore, some dentists prefer to measure dental caries in terms of tooth surfaces, rather than number of teeth, and report their results using an analogous decay-missing-filled surfaces (dmfs/DMFS) index. The maximum dmfs score is 88, and the maximum DMFS score is 128 or 148 depending on whether the third molars are counted.

The DMFT index is easy to measure, and inter-rater reliability is high. However, the primary shortcoming of the DMFT is that it does not discriminate well between current (untreated disease) and past (treated disease) caries experience. Strategies we employed to maximally utilise dmf/DMF data for estimating the prevalence of burden due to permanent caries are described below.

Input data and data processing

The approach for systematic literature review is described above. The reference definition for this model was presence of one or more teeth with current decay (for prevalence), whereas each additional carious tooth was counted as a separate incident event. Tables 1 and 2 presents the number of primary data sources for untreated caries in deciduous and permanent teeth included in GBD 2023.

Table 1. Number of data sources for untreated caries in deciduous teeth

Period	Central Europe, Eastern Europe, and Central Asia	High-income	Latin America and the Caribbean	North Africa and Middle East	South Asia	Southeast Asia, East Asia, and Oceania	Sub-Saharan Africa
<1990	9	79	0	1	0	7	19
1990-1994	8	74	3	8	0	11	8
1995-1999	2	44	27	12	1	8	11
2000-2004	4	65	13	23	5	21	18
2005-2009	9	67	37	11	8	19	10
2010-2014	11	56	73	31	66	21	10
2015-2019	6	5	8	8	16	6	1
2020-2023	0	0	0	0	0	0	0

Table 2. Number of data sources for untreated caries in permanent teeth

Year	Central Europe, Eastern Europe, and	High-income	Latin America and the Caribbean	North Africa and Middle East	South Asia	Southeast Asia, East Asia, and Oceania	Sub-Saharan Africa

	Central Asia						
<1990	16	84	1	0	0	2	17
1990-1994	9	80	4	0	4	5	8
1995-1999	5	38	9	4	1	6	15
2000-2004	2	50	8	13	8	15	13
2005-2009	13	52	21	16	9	12	11
2010-2014	14	19	32	19	23	16	11
2015-2019	7	4	8	2	4	2	3
2020-2023	0	0	0	0	0	0	0

Converting lifetime to current prevalence

Many of the studies presented dmft or DMFT scores, which represent lifetime prevalence and were often described as "caries experience". For the purposes of measuring the burden of disability from dental caries, we converted lifetime prevalence data to current prevalence for individuals aged 20 years and under. We did this by multiplying the observed lifetime prevalence by the ratio of d/D to dmf/DMF. When d/dmf or D/DMF information was available from the same study, this ratio was applied. When not available from the same study, the pooled ratio from the closest matching GBD geography was used for the multiplication (country, region, super-region, global).

Calculation of incidence from dmft/DMFT increment

Whereas in the deciduous dentition, a vast majority of the dmf index is accounted for by caries, tooth loss is a major contributor to the DMF index for the permanent dentition. Caries of permanent teeth may not necessarily be the primary driver of this tooth loss, as other factors such as periodontal disease and trauma may contribute significantly. Thus, we performed the conversions of incremental dmf/DMF scores to incidence values for permanent caries only in individuals aged 20 years or less and for all ages in the case of deciduous caries. For longitudinal studies, the difference between the dmf/DMF score in the initial versus subsequent examination was taken to be equivalent to the number of incident caries over that time period. This assumes a negligible proportion of dmf/DMF increment is due to trauma in children. For cross-sectional studies examining children of different ages, we only calculated incidence when the gap in age was ≤3 years given the propensity for strong cohort effects in caries epidemiology.

Age and sex splitting

For any datum that did not entirely fit within a GBD sex or age group, the observation was split to be multiple age-sex-specific datapoints based on the age and sex pattern predicted by previous DisMod-MR 2.1 models. It is our intention to update this with each cycle of GBD.

Crosswalks in MR-BRT

We then crosswalked alternative to reference definitions. To make data comparable, we began by evaluating the number of observations of each alternate definition that matched with a corresponding observation from the reference definition. Owing to the significant heterogeneity in data on caries incidence and prevalence, we limited the comparisons to only "within"-study matches, where a match was defined as both methods of ascertainment being performed in the identical study population. The ratio of alternative to reference was calculated and logit-transformed. Standard error of the ratio was calculated using the delta method. Sex was included as a fixed effect and, for prevalence only, midpoint of age as a spline. The adjustment factors and spline plots for the crosswalks are shown below.

The funnel plot is for demonstration only; the final crosswalk was derived from the MR-BRT model represented by the spline plot in Figure 1. For caries in deciduous teeth, we also adjusted data that were DMFS-derived (ie, calculated from d/dmfs as opposed to d/dmft), even though data are comparatively sparse, because there is strong suggestion of age-specific relationship in these two measures. This sub-analysis would be strengthened by additional data. We elected to adjust rather than drop the alternate data because the age groups involved are comparatively data-sparse. We intend to focus on identifying additional data for early childhood caries for the next GBD systematic data extraction effort.

Figure 1: Funnel plot (left) and spline plot by age (right) showing logit-transformed ratio of deciduous caries prevalence for alternate (converted from d/dmf) versus reference (d>0)

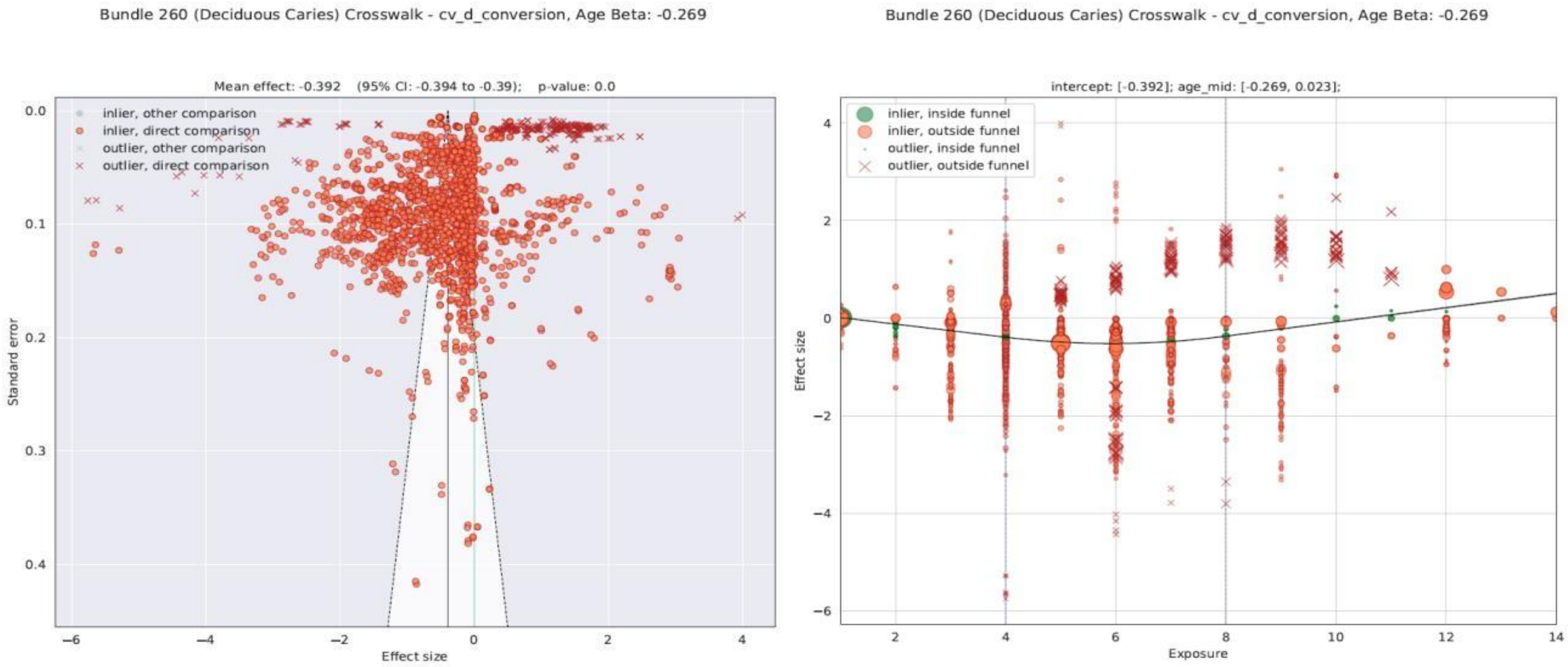

Figure 2: Funnel plot (left) and spline plot by age (right) showing logit-transformed ratio of deciduous caries incidence for alternate (dmfs measurement) versus reference (dmft measurement)

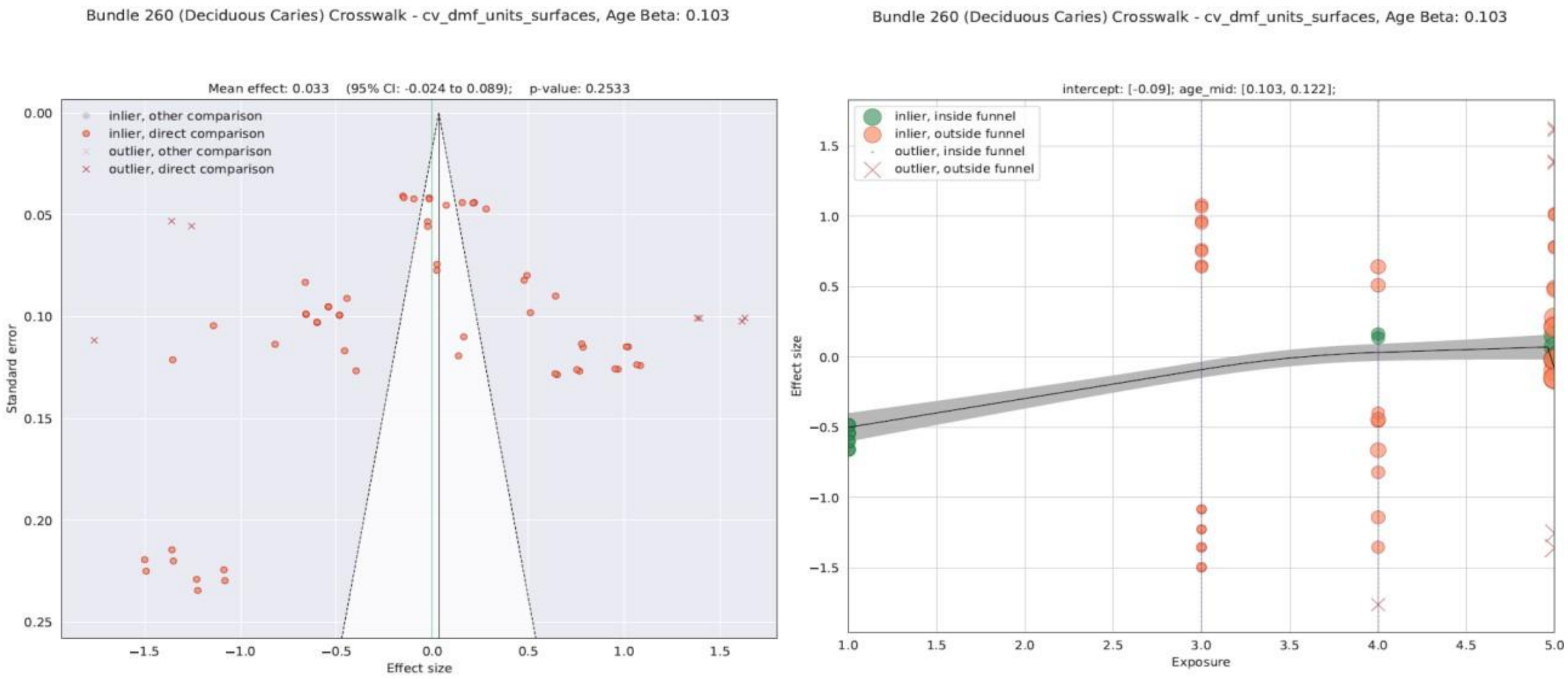

The same crosswalks were evaluated for data on caries of permanent teeth. The results of those crosswalks are shown in Figures 3 and 4, respectively.

Figure 3: Funnel plot (left) and spline plot by age (right) showing logit-transformed ratio of caries of permanent teeth prevalence for alternate (converted from D/DMF) versus reference (D>0)

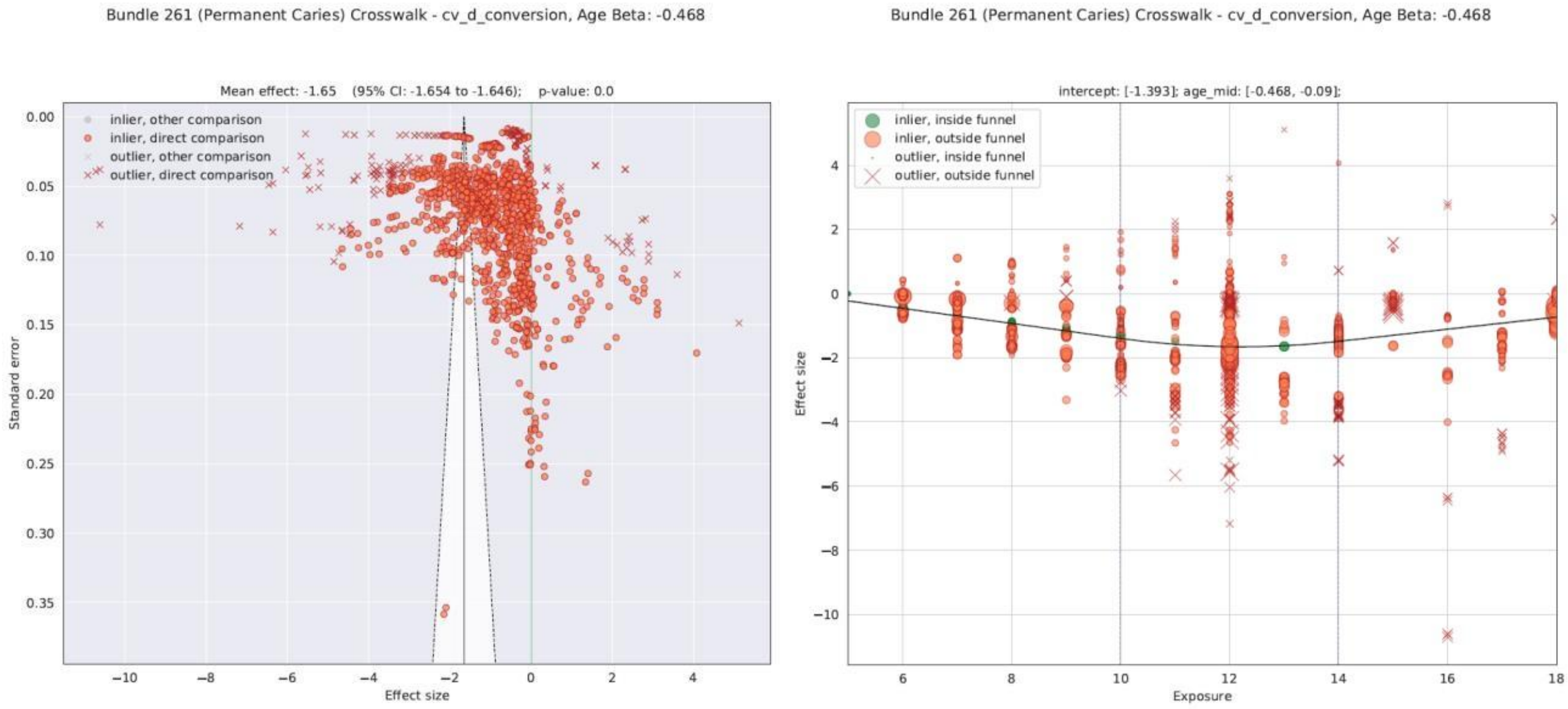

Figure 4: Funnel plot (left) and spline plot by age (right) showing logit-transformed ratio of caries of permanent teeth prevalence for alternate (DMFS measurement) versus reference (DMFT measurement)

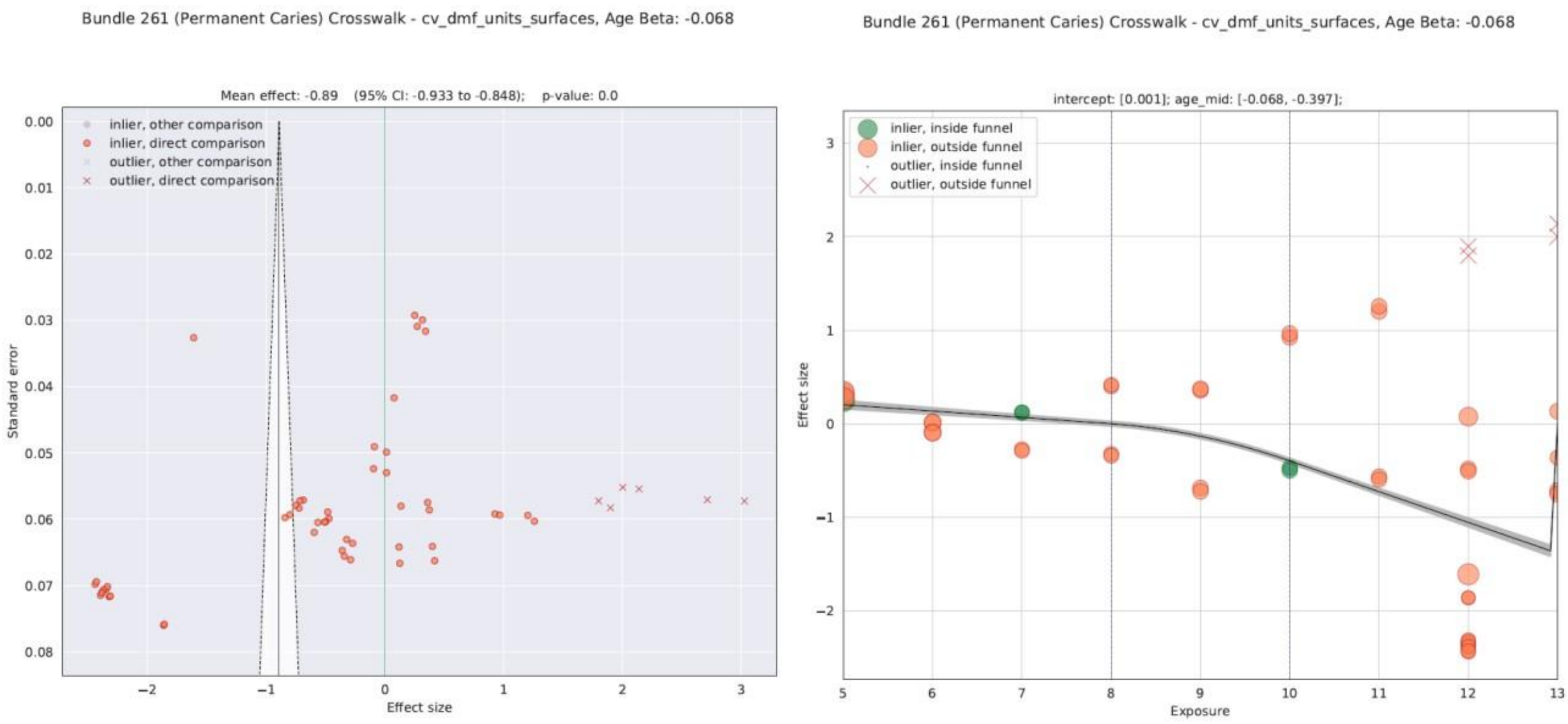

For prevalence data on caries of permanent teeth, there was a significant and age-dependent difference in prevalence data derived from measurement of surfaces as compared to teeth, so DMFS-derived prevalence data were adjusted to the reference of teeth as shown in Figure 4. As described above, the D/DMF conversion was only completed for data from children under 13 years of age because these were the only age groups from which we felt confident in deriving accurate measures of relationships between D/DMFS and D/DMFT. There were insufficient data to inform an assessment of whether or not there is a difference between cohort-based caries incidence and incidence derived from DMF increment.

Modelling strategy

DisMod model development

Serious health consequences of untreated caries were also assumed to be uncommon and death very rare. We therefore assigned excess mortality to be zero from age 0 to 100. For both types of caries, most of the model settings were similar. The primary difference was in value priors. We assumed zero incident caries in infants under 1 year old and similarly zero incident deciduous caries from age 11 onward. For caries in permanent teeth, we assumed zero incident cases in children under 5 years.

Location-level covariates were assigned separately on prevalence and incidence. Sugar availability in food from the GBD diet analysis was used as a covariate on incidence with a positive beta, while prevalence was assigned logtransformed LDI with a negative beta to reflect the association with access to dental care (Tables 3 and 4).

Table 3: Covariate, parameter, beta, and exponentiated beta values for dental caries in deciduous teeth

Covariate name	Measure	Beta (UI)	Exponentiated beta (UI)
LDI (I$ per capita)	Prevalence	–0.116 (–0.134 to –0.095)	0.89 (0.875–0.909)
Age- and sex-specific SEV for high sweetened beverages	Incidence	0.37 (0.022 to 0.878)	1.448 (1.023–2.407)

Table 4: Covariate, parameter, beta, and exponentiated beta values for dental caries in permanent teeth

Covariate name	Measure	Beta (UI)	Exponentiated beta (UI)
LDI (I$ per capita)	Prevalence	–0.171 (–0.216 to –0.131)	0.843 (0.806–0.877)
Age- and sex-specific SEV for high sweetened beverages	Incidence	0.3 (0.024 to 0.697)	1.35 (1.024–2.008)

Although studies were screened carefully during data extraction to ensure that they specified whether they were measuring untreated caries in permanent or deciduous teeth, some datapoints were marked as outliers during modelling due to their high prevalence values in young ages, as it was deemed likely that some of these studies were reporting caries in deciduous in addition to permanent teeth. As with caries in deciduous teeth, models for caries in permanent teeth were vetted based on the plausibility of the results, the extent to which estimates fit the data, and the plausibility of the range of estimates across location hierarchies.

Correction for edentulism

One systematic source of bias in the literature was the exclusion of edentate individuals from the study populations, which leads to systematic overestimation of caries prevalence when modelled over the entire population. To account for this bias, we used our GBD estimates of edentulism prevalence to adjust YLD estimates for caries in permanent teeth. Final DisMod-MR 2.1 estimates of edentulism prevalence were paired with the corresponding results for caries in permanent teeth by age group, sex, location, and year to adjust for the proportion of the population that was excluded from the denominator of caries in permanent teeth models. No adjustment was made to the estimates of caries in deciduous teeth.

Severity distributions and disability weights

As described above, the GBD definition of disability associated with symptomatic dental caries is “this person has a toothache, which causes some difficulty eating”. The disability weight associated with this condition is 0.01 (0.005–0.019), as derived from the 2010 GBD disability weights study.

Not all those with dental caries experience this disability all the time. We considered only those with active dentinal decay to experience symptomatic tooth pain. Those with caries in deciduous teeth who had undergone exfoliation or had their cavities filled were considered to have no disability. Likewise, those with caries in permanent teeth who had received fillings, had their cavities extracted, or lost a carious tooth altogether were considered to have no disability. Thus, two additional pieces of information are required to complete the calculation of YLDs: proportion with symptoms and duration of disability.

To determine which segment of the population has ongoing tooth pain and the proportion of time spent with tooth pain, we considered several different options. First, we examined the data on dental caries symptoms and disability from the Medical Expenditure Panel Survey (MEPS) conducted by the USA Department of Health and Human Services in 2000–2009. MEPS data were widely used in GBD 2010 analyses. Respondents to the survey are asked about all medical conditions. Conditions for which provider care was sought are reported by the respondents at every round, and respondents also report problems for which they did not see a provider if the symptoms were "bothering" them. Conditions can be added to the condition roster if 1) they are reported as a reason for a medical event, 2) the condition was reported as the reason for one or more disability days, or 3) the condition was "bothering" the person during the reference period. Conditions are then recorded as verbatim text and coded to ICD9CM 3rd digit codes by professional medical coders. These ICD-9 codes were mapped to GBD causes, including dental caries. From the MEPS, symptomatic caries in the previous year were reported by 48.4% (44.3–52.9) of the respondents. This number is in agreement with our DisMod-MR 2.1 estimates of 1–2 years' duration in high-income North America for permanent caries if we consider people to only have symptoms at the end of a course of caries. The two primary shortcomings of using this approach are 1) it does not provide enough detail to differentiate between the experiences of those with caries in deciduous versus permanent teeth, and 2) it indicates the proportion of those with caries who were symptomatic during the previous year, but it does not provide information on the amount of time during that year spent with symptoms (ie, one day versus 12 months). The approach described below addresses both issues.

To determine duration, we adapted the method employed by the Australian Burden of Disease (AusBoD) Study in 1996. For total duration, we used the posterior estimates of duration from final DisMod-MR 2.1 models. For those with symptoms, we split this total duration into two distinct phases of caries disability. The "initial" phase is characterised by *periodic* pain that we assigned to occur an average of one hour per day. The "terminal" phase is a period of *constant* symptoms at the end of an episode. The length of the terminal phase was determined by literature review as described by the AusBoD group. For deciduous caries, we used a study by Mason and colleagues of children in the UK presenting to a casualty ward with tooth pain.[1] The length of time each child had been experiencing tooth pain was recorded. Based on the distribution of time courses, a log-normal distribution was plotted that approximated the average duration of *constant* symptoms at 27.6 days leading up to seeking care. For permanent caries, a similar study of the tooth pain experience of adults in New Zealand who presented to hospital dental departments and an emergency clinic[2] resulted in an estimated 55.2 days spent in the terminal phase of caries. For those with severe disease, the length of time spent in the terminal phase was subtracted from the total duration to determine the amount of time spent in the initial phase. For those with mild disease, we considered the entire duration to be spent in the initial phase. These calculations were last completed as part of the GBD 2013 analysis.

To determine proportion with symptoms, we completed a supplemental literature review of tooth pain and caries. We identified a total of 21 studies with data about the prevalence of pain. The studies were grouped according to the type of dentition studied (deciduous or permanent) and the location of the study group (high-income or low- and middle-income countries). We extracted data on the proportion in each group who described symptoms of pain related to their caries as well as a subset who described their symptoms as being severe. The proportions in each group were weighted according to sample size to give estimates of the relative sizes of three groups: asymptomatic, mild, and severe. The results of this meta-analysis are illustrated in Table 5.

Table 5: Duration and distribution of severity for tooth pain due to untreated caries in deciduous and permanent teeth

	# of studies	% symptomatic of total	% severe among symptomatic	% mild of total	% severe of total	% asymptomatic of total
		Deciduous caries				
Data-rich	5	0.35	0.257	0.26	0.09	0.65
All others	4	0.555	0.438	0.312	0.243	0.445
		Permanent caries				
Data-rich	6	0.602	0.315	0.412	0.189	0.398
All others	6	0.954	0.548	0.432	0.521	0.046
		Duration of phases				

	Initial phase	1 hour per day
	Terminal phase (deciduous caries)	27.6 days
	Terminal phase (permanent caries)	55.2 days

We considered asymptomatic individuals to experience no disability. Those with mild disease spent the entire duration in the initial phase of disease (one hour of pain per day). Those with severe disease spent a majority of the duration in the initial phase followed by a period of time in the terminal phase (constant pain). YLDs were calculated by multiplying the prevalence, duration, proportion, and disability weight for each age, country, sex, and year.

References

1. C Mason, SR Porter, G Madland, J Parry. Early management of dental pain in children and adolescents. *J Dent.* Jan 1997; 25(1): 31-4.
2. RA Whyman, ET Treasure, KM Ayers. Dental disease levels and reasons for emergency clinic attendance in patients seeking relief of pain in Auckland. *NZ Dent J.* Dec 1996; 92(410): 114-7.

IV. Severe periodontitis

Flowchart

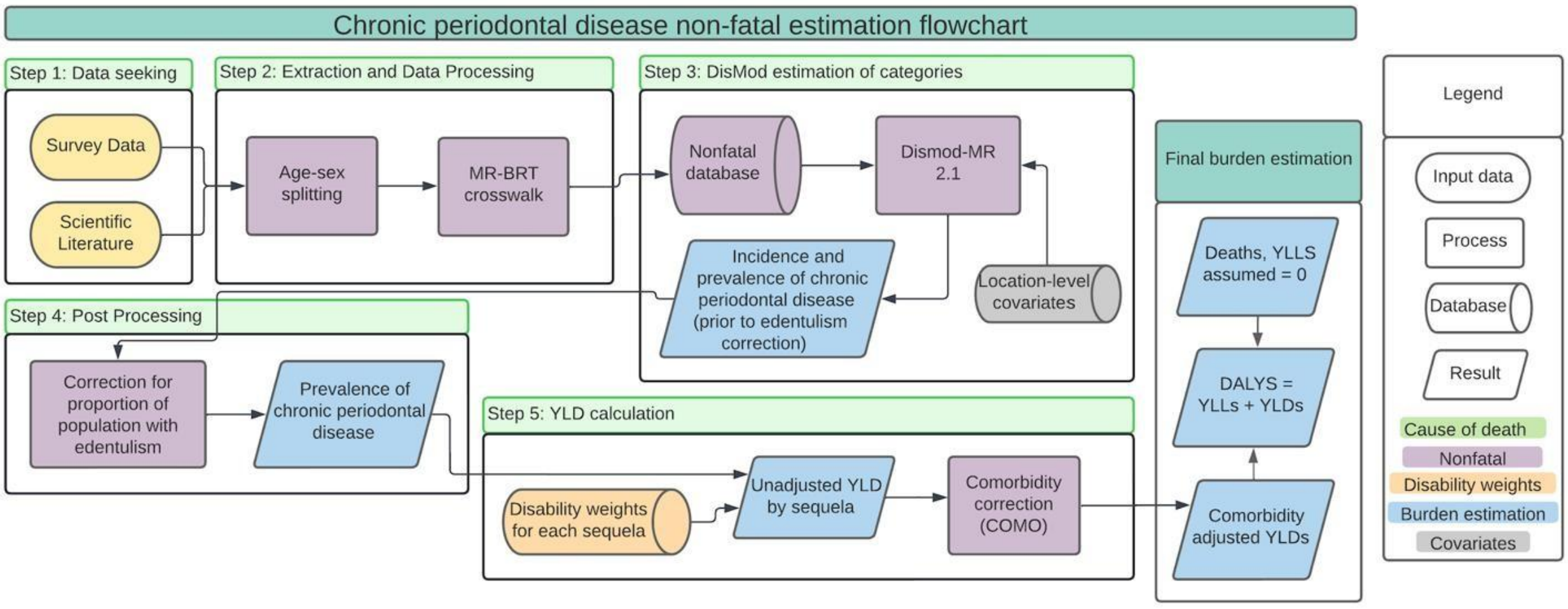

Key modelling assumptions:

- Alternative definitions (PPD≥4mm, CAL≥5mm and CAL≥4mm) were crosswalked to the reference definition of severe periodontitis (PPD≥6mm, CAL≥6mm or PPD>5mm)
- Mortality was fixed at zero, and relative risk was fixed to 1.0 for individuals younger than 30 years. Incidence and prevalence were set to zero up to age 8 years, with incidence allowed to increase from age 9 onwards. Remission was constrained between 0 and 0.05, excess mortality rate between 0 and 0.0001, and incidence between 0 and 0.05.
- Log-transformed lag-distributed income along with age-standardised summary exposure value for smoking and high fasting plasma glucose were included as covariates.
- Estimates were adjusted for edentulism using GBD estimates of edentulism prevalence.

Case definition

Chronic periodontal disease is caused by chronic bacterial infection around the teeth. Symptoms of gingivitis, the mildest form of the disease, include swelling, redness, and propensity of the gums to bleed when perturbed. If the infection is not treated appropriately, it will eventually spread below the gum line, leading to a chronic inflammatory state of the periodontal tissues. Over time, there will be loss of gingival tissue and alveolar bone destruction. Teeth will become loose and may need to be extracted.

The GBD definition of disability associated with symptomatic severe periodontal disease is "bad breath, a bad taste in the mouth, and gums that bleed a little from time to time, but which does not interfere with daily activities". The ICD-10 codes for periodontal disease are K05.0–K05.6, and the ICD-9 codes are 523.0–523.9.

Defining periodontal disease in a meaningful, reproducible manner has been an ongoing challenge for public health dentists. Attachment loss (AL) and pocket depth (PD) have emerged as the most common metrics of periodontal health measurement. AL is measured as the difference between the distance from the gingival margin to the bottom of the pocket and the distance from the cemento-enamel junction to the bottom of the pocket.

The Community Periodontal Index (CPI) is a classification system that was developed by WHO as a standardised method of periodontal health measurement. CPI classification is based on the examination of all teeth present in the mouth for absence or presence of gingival bleeding and absence or presence of periodontal pockets.[1] A standard-sized probe is used, with depth markings from 0.5 to 11.5 mm. The probe is inserted into the sulcus between a tooth and the gingiva until it meets resistance. The surrounding area is then explored with the probe to determine the maximum depth of the pocket. Multiple areas around each tooth are probed. Pocket scores range from 0 to 2 in order of increasing severity. When the CPI method was employed, we considered those with Class 2 only (pocket of 6 mm or more). Additionally, loss of attachment may be collected for specific index teeth by dividing the mouth in sextants. The two molars in each posterior sextant are paired for recording and, if one is missing,

there is no replacement. If no index tooth is present in a sextant qualifying for examination, all the teeth that are present in that sextant are examined and the highest score is recorded as the score for the sextant. We excluded studies in which the study population was reported as the number of sextants rather than the number of individuals surveyed. CPI is a modification of Community Periodontal Index of Treatment Needs (CPITN) that does not include the assessment of periodontal treatment needs. Also, Class 2 of CPI is equivalent Class 4 of CPITN.

In 2007, a new CDC proposal for gold-standard diagnosis of severe, chronic periodontitis was published.[2] This standard specified that a stricter definition of the condition should be implemented. This more exclusive definition of chronic periodontal disease includes $\geq$2 interproximal sites with AL $\geq$6mm *AND* $\geq$1 interproximal site with PD $\geq$5mm. This definition has not been adopted by GBD.

A small body of evidence has begun to emerge that implicates chronic periodontal disease as predisposing individuals to increased risk for ischaemic cardiovascular events including myocardial infarction and stroke. These data are sparse but have been included in models estimating the excess mortality of those with chronic periodontal disease. Given that the association is believed to be ecological rather than causal, however, periodontal disease has not been estimated as an underlying cause of death and it is not included in the risk factor analysis for cardiovascular diseases.

Input data

Details of the systematic review are provided above. We implemented a hierarchical preference for case definitions. We included the following definitions of severe periodontal disease commonly found in the literature:

1. CPITN – Class 4 only
2. CPI – Class 4 only
3. Clinical AL >=6 mm
4. Clinical AL >=5 mm
5. Clinical AL >=4 mm
6. Gingival pocket depth (PD) >5 mm

If more than one type of data was included in a study, our first preference was for CPITN = 4, followed by clinical AL>=6 mm, and PD >5 mm. All were considered equivalently as reference definitions with no additional crosswalking performed. For those sources that did not provide data on any of the components of CPITN Class 4, but did provide data on CPITN Class 3, CPI Class 3, AL >=5 mm, or AL>=4 mm, we utilised these data after crosswalking in MR-BRT as described below. Table 1 presents the number of primary data sources for severe periodontitis included in GBD 2023.

Table 1. Number of data sources for severe periodontitis

Period	Central Europe, Eastern Europe, and Central Asia	High-income	Latin America and the Caribbean	North Africa and Middle East	South Asia	Southeast Asia, East Asia, and Oceania	Sub-Saharan Africa
<1990	2	35	1	2	2	9	3
1990-1994	0	18	3	3	0	1	6
1995-1999	1	18	0	2	0	6	4
2000-2004	2	17	3	2	2	8	1
2005-2009	1	20	9	0	0	5	0
2010-2014	0	16	3	0	3	6	2
2015-2019	1	2	0	0	0	4	0
2020-2023	0	0	0	0	0	0	0

Age-sex splitting

For any datum that did not entirely fit within a GBD sex or age group, the observation was split to be multiple age-sex-specific datapoints based on the age and sex pattern predicted by previous DisMod-MR 2.1 models.

Crosswalks in MR-BRT

We then crosswalked alternative to reference definitions. To make data comparable, we began by evaluating the number of observations of each alternate definition that matched with a corresponding observation from the reference definition. All alternative definitions were mutually exclusive with one another, so three separate crosswalks were performed using only within-study matches, defined as both methods of ascertainment being performed in the identical study population. The ratio of alternative to reference was calculated and logit-transformed. Standard error of the ratio was calculated using the delta method. Sex was included as a fixed effect and, for prevalence only, midpoint of age as a spline. The total number of matches, the adjustment factors, and the spline plots for periodontal disease crosswalks are shown below.

Figure 1: Funnel plot (left) and spline plot by age (right) showing logit-transformed ratio of chronic periodontal diseases prevalence as measured with alternate (top = CPITN 3; middle = attachment loss >4 mm; bottom = attachment loss >5 mm) versus reference (CPITN 4, AL =>6 mm)

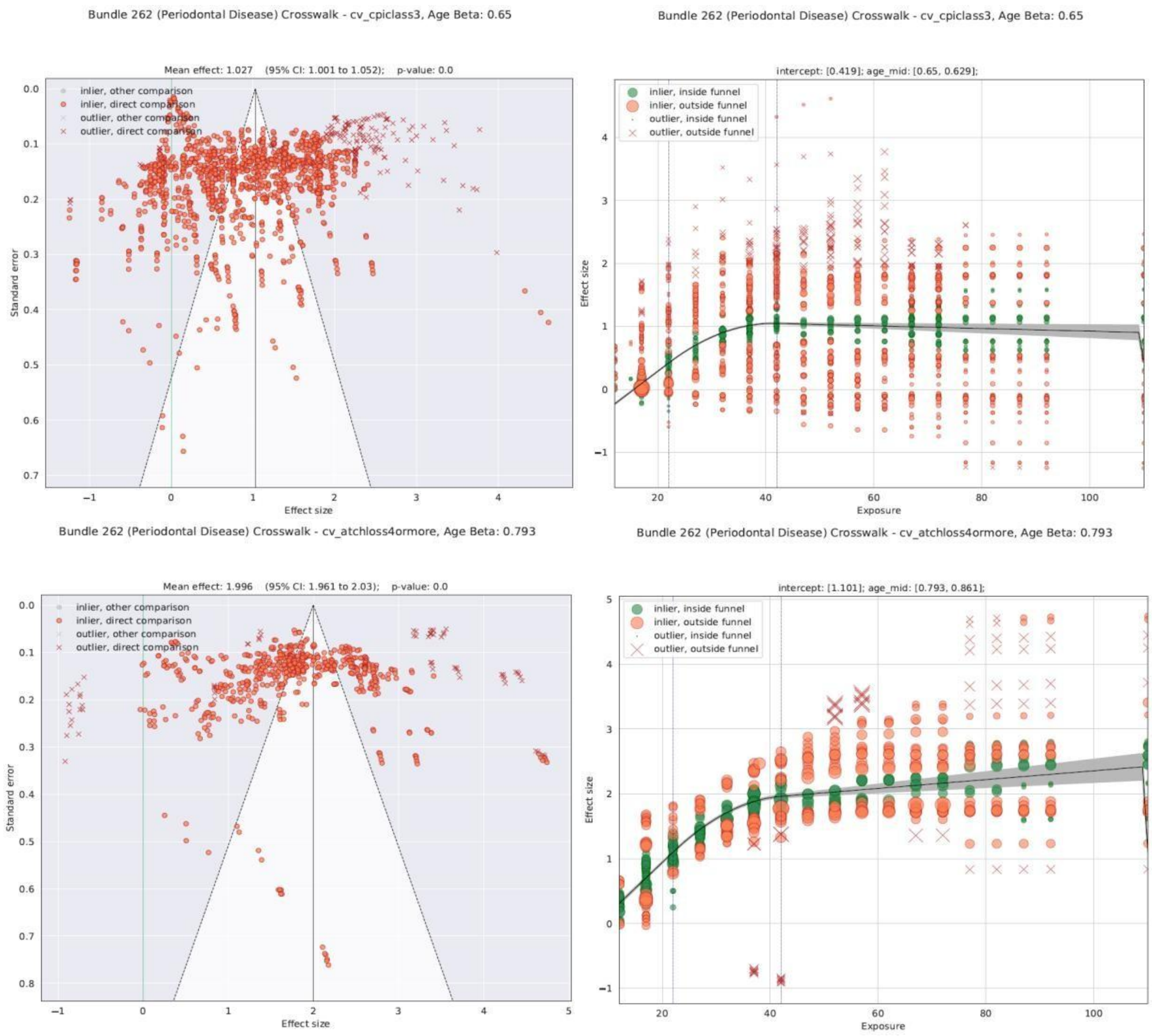

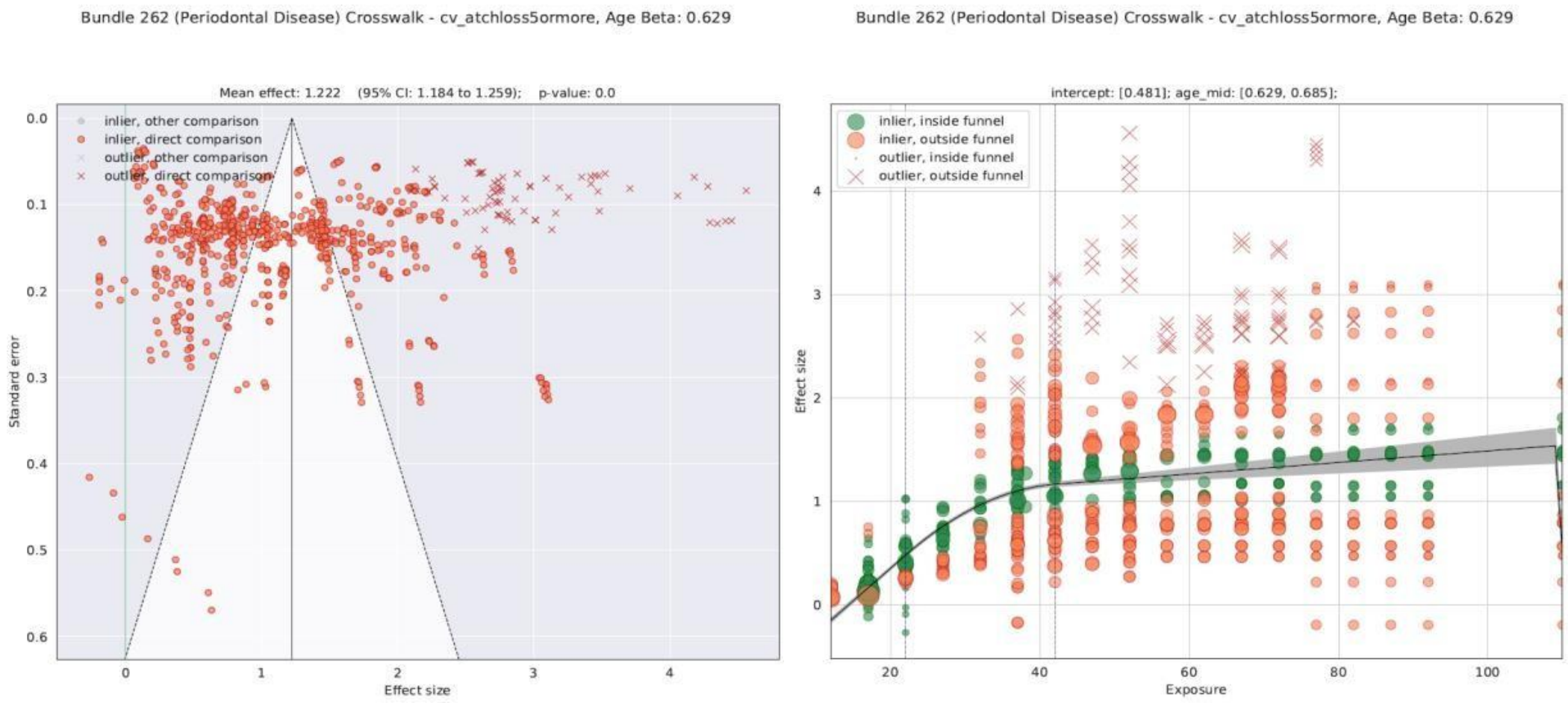

Modelling strategy

First, estimates for the prevalence of chronic periodontal disease were generated for each location/year/sex/age using DisMod-MR 2.1. Mortality was fixed to zero, and relative risk was fixed to 1.0 before age 30, as any excess cardiovascular events that occur in those with severe tooth loss would not be expected at young ages. Incidence and prevalence were assigned to be zero until age 8, as periodontal disease is largely considered to be a disease of adulthood. Incidence was allowed to rise beginning at age 9, based on the youngest age at which there was a non-zero point estimate for prevalence in the dataset. Additional bounds were assigned for incidence, remission, and excess mortality to improve plausibility in the DisMod estimates. Remission was bounded from 0 to 0.05, excess mortality rate from 0 to 0.0001, and incidence from 0 to 0.05. We considered these bounds to reasonably reflect the natural history of the disease. Three location-level covariates were used as shown in the table below.

Table 2: Covariate, parameter, beta, and exponentiated beta values for chronic periodontal diseases

Covariate name	Measure	Beta (UI)	Exponentiated beta (UI)
LDI (I$ per capita)	Prevalence	0.21 (0.156–0.261)	1.234 (1.169–1.298)
Age-standardised SEV for smoking	Prevalence	0.171 (0.01–0.464)	1.187 (1.01–1.591)
Age-standardised SEV for high fasting plasma glucose	Prevalence	0.39 (0.032–0.985)	1.476 (1.032–2.678)

Models were vetted based on the plausibility of the results, the extent to which estimates fit the data, and the plausibility of the range of estimates across location hierarchies.

Correction for edentulism

One systematic source of bias in the literature was the exclusion of edentate individuals from the study populations, which leads to systematic overestimation of periodontal disease prevalence when modelled over the entire population. To account for this bias, we used our GBD estimates of edentulism prevalence to adjust YLD estimates for chronic periodontal disease. Final DisMod-MR 2.1 estimates of edentulism prevalence were paired with the corresponding results for periodontal disease by age group, sex, location, and year to adjust for the proportion of the population that was excluded from the denominator of periodontal disease models.

Severity distributions and disability weights

We considered all estimated prevalent cases of chronic periodontal disease to experience the disability described by “bad breath, a bad taste in the mouth, and gums that bleed a little from time to time, but this does not interfere with daily activities”. The 2010 GBD disability survey differentiated between those

who experience pain and those who do not, but the calculated disability weight was the same for both forms of the condition, 0.007 (0.003–0.014).

References

1. Petersen PE, Baez RJ. Oral health surveys: basic methods. Geneva: World Health Organization; 2013.
2. RC Page and PI Eke. Case Definitions for Use in Population-Based Surveillance of Periodontitis. *J Periodont*. Jul 2007. 78(7 Suppl): 1387-99

V. Other oral disorders

Other oral disorders encompass a wide variety of dental, tongue, and jaw disorders and malformations, including all oral disorders that are not included in the case definitions of permanent or deciduous dental caries, severe periodontitis, or edentulism. All data on the prevalence of other oral disorders were obtained from the USA Medical Expenditure Panel Survey (MEPS), a nationally representative survey conducted yearly from 1996 to 2011 by the US Agency for Healthcare Research and Quality. Table 1 presents the number of primary data sources for other oral disorders included in GBD 2023.

Table 1. Number of data sources for other oral disorders

Period	**Central Europe, Eastern Europe, and Central Asia**	**High-income**	**Latin America and the Caribbean**	**North Africa and Middle East**	**South Asia**	**Southeast Asia, East Asia, and Oceania**	**Sub-Saharan Africa**
<1990	0	0	0	0	0	0	0
1990-1994	0	0	0	0	0	0	0
1995-1999	0	4	0	0	0	0	0
2000-2004	0	10	0	0	0	0	0
2005-2009	0	10	0	0	0	0	0
2010-2014	0	7	0	0	0	0	0
2015-2019	0	0	0	0	0	0	0
2020-2023	0	0	0	0	0	0	0

These data were modelled in DisMod-MR 2.1 using a prevalence-only model. Disability weights and severity distribution for these causes were also derived from MEPS.

VI. Lip and oral cavity cancer

This general framework for the GBD 2023 cancer estimation applies to all malignant neoplasms (ie, cancers) except for non-melanoma skin cancer (including basal cell carcinoma and squamous cell carcinoma); benign and in situ neoplasms (including intestinal; cervical and uterine; and other benign neoplasms); and myelodysplastic, myeloproliferative, and other haemopoietic neoplasms.

Flowcharts

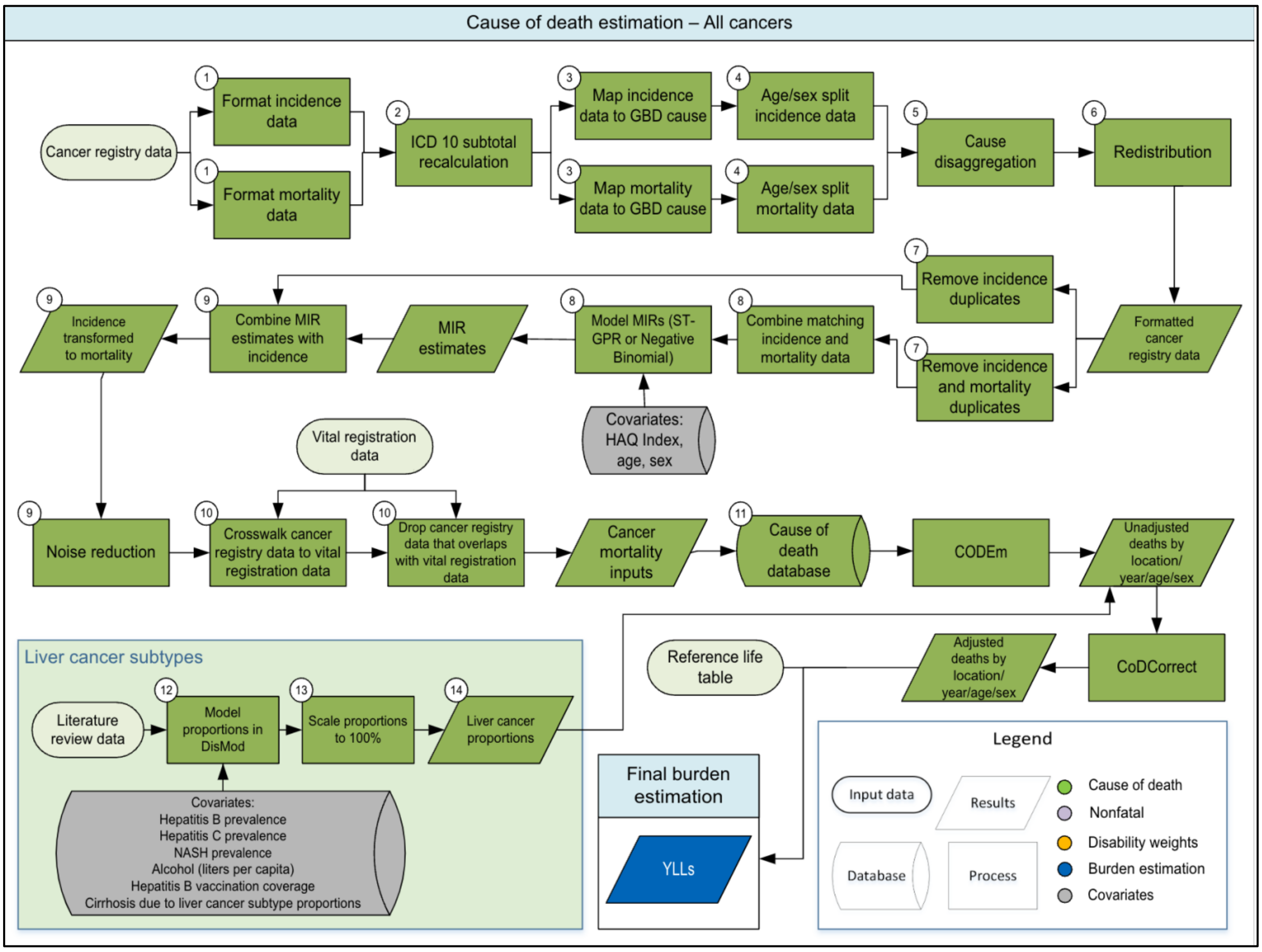

Appendix Figure 1: Flowchart of GBD cancer mortality and YLL estimation. Abbreviations: CODEm, cause of death ensemble model; DisMod-MR, disease model - Bayesian meta-regression; GBD, Global Burden of Disease study; HAQ Index, Healthcare Access and Quality Index; ICD, International Classification of Diseases; MIR, mortality-to-incidence ratio; NASH, nonalcoholic steatohepatitis; ST-GPR, spatiotemporal Gaussian process regression; YLLs, years of life lost.

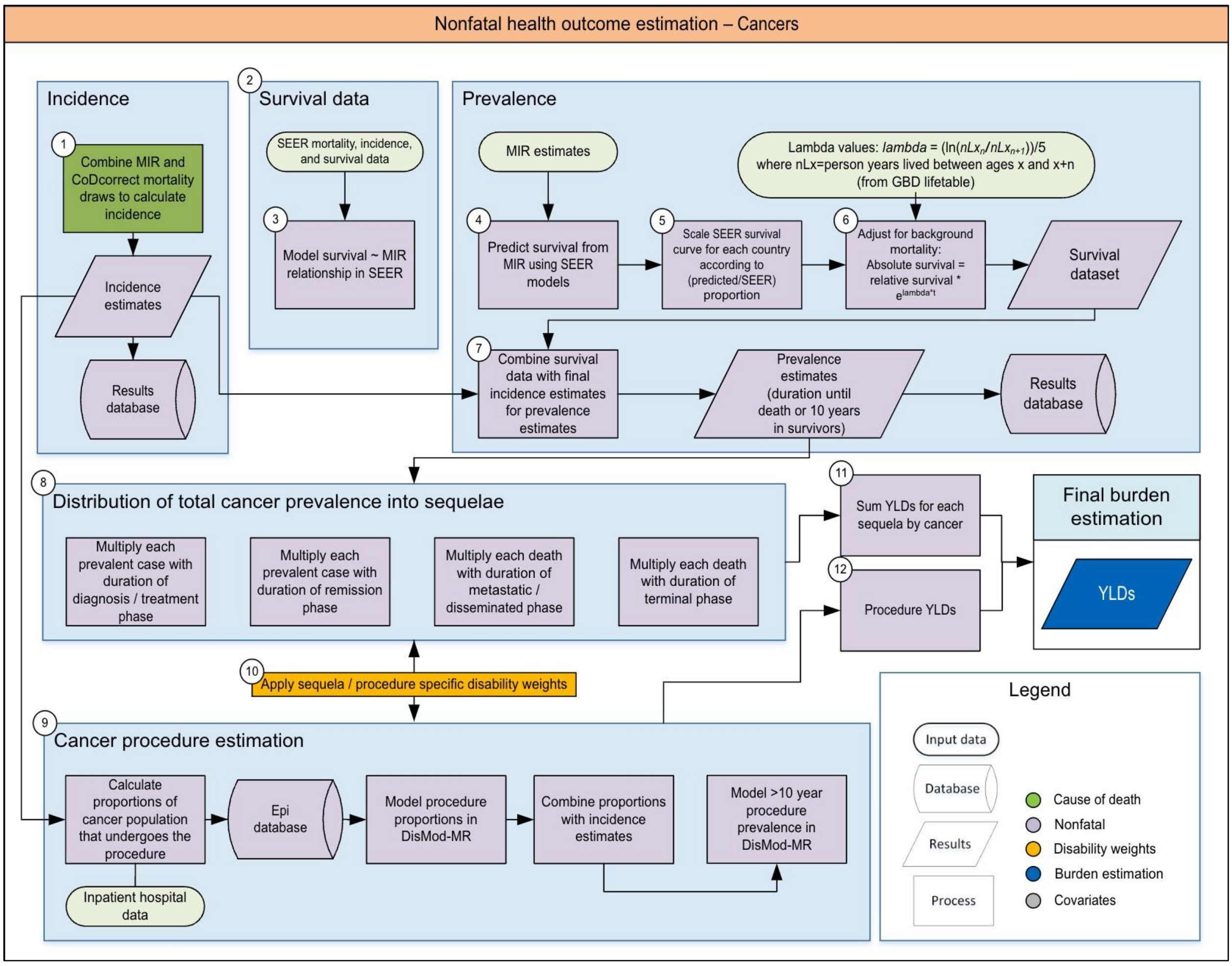

Figure 1. Flowchart of non-fatal estimation for all cancers except non-melanoma skin cancer, benign and in situ neoplasms, and myelodysplastic, myeloproliferative, and other haemopoietic neoplasms. Abbreviations: DisMod-MR, disease model – Bayesian meta-regression; GBD, Global Burden of Disease study; MIR, mortality-to-incidence ratio; SEER, Surveillance, Epidemiology, and End Results Program; YLDs, years lived with disability

Key modelling assumptions:

- Cancer registry data was crosswalked to vital registration statistics.
- Start and end age for estimation were set at 15-19 years and 95 years or more, respectively.
- Log-transformed lag-distributed income, years of education per capita, healthcare access and quality index, socio-demographic index, age-and-sex specific summary exposure value for high red meat, low fruit and low vegetables, and alcohol consumption per capita, cigarettes per capita and cumulative cigarettes (for women) were included as covariates in the mortality model whereas the healthcare access and quality index was included as covariate in the incidence model.

Case definition

For GBD 2023, incidence, prevalence, and disability are estimated for all cancers and benign neoplasms as defined in ICD-10 (C00–D49). The associated ICD codes for neoplasms estimated for GBD 2023 are listed elsewhere in the GBD summary papers. Prevalence for cancers are estimated for a maximum of ten years after incidence, as in GBD 2013, GBD 2015, GBD 2016, GBD 2017, GBD 2019, and GBD 2021.[1–5] Prevalence extending beyond the ten-year period is only estimated for permanent sequelae resulting from five treatment-related surgical procedures (cystectomy, laryngectomy, mastectomy, prostatectomy, and stoma). To estimate disability for each cancer, the prevalence is split into four sequelae representing various phases of cancer, each with a corresponding disability weight. Additional disability is estimated for the five cancers with surgical procedures.

Table 1. Clinical case definition for all cancers estimated in GBD 2023

Quantity of interest	Clinical case definition
Lip and oral cavity cancer	Malignant neoplasms of the lips and oral cavity; ICD-10 codes C00-C08.

Input data

GBD causes of death (CoD) database contains cancer mortality data originating from multiple sources, including vital registration (VR), verbal autopsy (VA), and population-based cancer registry (CR) data. The cancer registry mortality estimates that are uploaded into the CoD database stem from cancer registry incidence data that have been transformed to mortality estimates through the use of mortality-to-incidence ratios (MIRs). The number of primary data sources for lip and oral cavity cancer in GBD 2023 by super-region and year can be found in Table 1.

Table 1. Number of data sources for lip and oral cavity cancer

Period	Central Europe, Eastern Europe, and Central Asia	High-income	Latin America and the Caribbean	North Africa and Middle East	South Asia	Southeast Asia, East Asia, and Oceania	Sub-Saharan Africa
<1990	34	136	3	1	1	43	2
1990-1994	14	86	10	3	1	33	10
1995-1999	11	108	7	6	3	35	9
2000-2004	18	119	4	26	3	36	13
2005-2009	43	138	0	28	7	41	20
2010-2014	52	101	2	8	17	27	27
2015-2019	16	38	0	2	0	6	10
2020-2023	2	0	0	0	0	0	0

Most population-based cancer registries only report cancer incidence. However, if a cancer registry also reported cancer mortality, mortality data were also extracted. Cancer registry sources with matching incidence and mortality data were used in the mortality-to-incidence ratio (MIR) estimation.

To align estimates of incidence with estimates of mortality for each cancer site, ratios of mortality to incidence are modelled using matching cancer registry data. By multiplying these mortality-to-incidence ratios (MIRs) with incidence data from cancer registries where vital registration data is absent or of low quality, mortality data can be added to the cause of death modelling in CODEm. After the cause of death estimates have been finalised and passed through CoDCorrect, cancer incidence is calculated by dividing the cancer mortality estimates by the estimated MIRs. Data sources for cancer mortality are described elsewhere in the GBD summary articles. Data sources for cancer registry incidence data are limited to those that have matching mortality data or do not have overlapping vital registration data. Data sources used to estimate prevalence come from the USA SEER[6] (Surveillance, Epidemiology, and End Results Program) data for matching incidence, mortality, and survival. For a minority of cancer types not consistently captured by cancer registries, prevalence data from clinical informatics data sources are used. Data sources used to estimate the proportion of cancer patients undergoing surgical procedures and to determine sequelae duration will also be listed below.

Table 2. Data inputs for neoplasms morbidity modelling by parameter

Cause	Prevalence sources	Incidence sources	Deaths sources	All measures sources
Neoplasms	375	3146	5293	9110
Lip and oral cavity cancer	3	817	4487	5307

Modelling strategy

Estimation of cancer mortality and MIR estimation has been described elsewhere in the GBD summary articles. As both the fatal and non-fatal estimation processes utilise these same modelled MIR estimates, the MIR estimation process is detailed again below. To summarise, incidence and mortality data from cancer registries were matched by cancer, age, sex, year, and location to generate input MIRs, which were then used to obtain MIR estimates using one of two MIR modelling approaches, depending on the cancer. In the non-fatal process, these estimated MIRs were then used to transform the final GBD cancer mortality estimates into GBD incidence estimates.

MIR data processing

For all causes that existed in GBD 2023, data-cleaning steps for MIR estimation were the same as for GBD 2021. For each cancer, MIRs from locations in Healthcare Access and Quality Index (HAQ Index) quintiles 1–4 were dropped if they were below the median of MIRs from locations in HAQ Index quintile 5. We also dropped MIRs from locations in HAQ Index quintiles 1–4 if the MIRs were above an outlier threshold calculated as the third quartile + 1.5 * IQR (inter-quartile range). We dropped all MIR data that were based on fewer than 15 incident cases to avoid excessive variation in the ratio due to small numbers.

For the lower end of the age spectrum where cancers are generally rarer, we previously aggregated incidence and mortality to the youngest five-year age bin where SEER[7] reported at least 50 cases from 1990 to 2015, to avoid unstable MIR predictions in young age groups because of too few cases or deaths. For GBD 2023, instead of using the SEER-based age threshold, we allowed age groups with greater than ten observations and more than one unique location to retain their age-specific data and be modelled independently. As before, for age groups below this new threshold, the MIR estimates were then copied to all younger GBD age groups estimated for that cancer.

Since MIRs can be above 1, especially in older age groups and for cancers with low cure rates, we used the 95th percentile (by age group) of the cleaned dataset (detailed above) to cap the MIR input data. These “upper cap” values were used to allow MIRs over 1 in some age groups but to constrain the MIRs to a maximum level. The addition of new data for GBD 2023 led to slightly different upper caps compared to GBD 2021 (see upper cap values for GBD 2023 below). For GBD 2023, we no longer set a minimum upper cap of 1 (regardless of the 95th percentile) for paediatric age groups (under 20 years).

**Age group (years)**	**0–4**	**5–9**	**10–14**	**15–19**	**20–24**	**25–29**	**30–34**	**35–39**	**40–44**	**45–49**
**Upper cap**	0.678	0.750	0.793	0.961	0.954	0.798	0.752	0.812	0.859	0.866

**Age group (years)**	**50–54**	**55–59**	**60–64**	**65–69**	**70–74**	**75–79**	**80–84**	**85–89**	**90–94**	**95+**
**Upper cap**	0.893	0.925	0.973	1.02	1.06	1.16	1.28	1.41	1.61	1.84

Any MIR values over this upper cap were winsorised to the cap value. To run the logit model in ST-GPR, the input data were first divided by the upper caps to get proportional data ranging from 0 to 1. Model predictions from ST-GPR were then rescaled back to MIRs by multiplying the scaled predictions by the upper caps. To constrain the MIRs at the lower end, we used the fifth percentile of the cancer and age-specific cleaned MIR input data to winsorise all model predictions below this lower cap.

MIR data modelling

As in previous GBD cycles, MIRs for most cancers were estimated with a three-step modelling approach using the general GBD spatiotemporal Gaussian process regression (ST-GPR) approach. The first step used a linear model for logit-transformed MIR as the outcome, with covariates for HAQ

Index, sex, and categorical age group (number of categories depending on cancer type).[8] For the MIRs, this initial linear prior used the following general form:

$$\text{logit}\left(MIR_{c,a,s,t}\right) = \alpha + \beta_1(HAQIndex)_{c,t} + \beta_2 \mathrm{I}_s + \sum_{a}^{A} \beta_a \mathrm{I}_a + \epsilon$$

MIR: mortality-to-incidence ratio

c: country (or subnational for subnationally modelled locations), a: age group, t: time (years); s: sex HAQIndex: Healthcare Access and Quality Index

I: indicator variable

ϵ: error term

Results from the final linear model were used as input for spatiotemporal smoothing and a Gaussian process regression. The ST-GPR model has three main hyper-parameters that control for smoothing across time, age, and geography. These hyper-parameter values were iteratively evaluated in GBD 2019 for balancing model performance with local data availability and were unchanged for GBD 2023. The time adjustment parameter lambda (λ) aims to borrow strength from neighbouring time points (ie, the value in this year is highly correlated with the value in the previous year but less so further back in time) and was set to 0.05. The age adjustment parameter omega (ω) borrows strength from data in neighbouring age groups and was set to 0.5. The space adjustment parameter zeta (ζ) aims to borrow strength across the hierarchy of geographical locations and was set to 0.01. For the remaining parameters in the Gaussian process regression, we set amplitude to 1 (influences fluctuation from the mean function) and set the scale value to 10 (influences the time distance over which points are correlated). Additional details on ST-GPR are described elsewhere in the GBD summary papers. These models were used to obtain MIR estimates for all combinations of GBD age, sex, year, cause, and location. Datapoints were outliered manually if they clearly influenced the model in an unrealistic way. For example, a datapoint was marked as an outlier if it created a single-year, single age group spike in model predictions that was inconsistent with the trend suggested by surrounding datapoints.

Mortality estimation

Mortality estimates for each cancer were generated using the GBD CODEm approach, the methods of which have been described in previous publications. Additional details can be found in Section X of the appendix to the GBD 2023 paper, "Global burden of 292 causes of death in 204 countries and territories and 660 subnational locations, 1990–2023: a systematic analysis for the Global Burden of Disease Study 2023. Lancet". In brief, the CODEm approach is based on several principles: that all types of available data should be used, even if data quality varies; that a diverse set of plausible models with different combinations of covariates should be evaluated; that both individual models and the overall ensemble models should be tested for their predictive validity; and that the best model or sets of models should be chosen based on the out-of-sample predictive validity.

Covariates are provided for potential use in the ensemble based on a possible predictive relationship between the covariate and the specific cancer mortality, with an expected level and direction of association. Generally, Level 1 covariates have a proven strong relationship with the outcome, such as aetiological or biological roles. Level 2 covariates have a strong relationship but no known direct biological link. Level 3 covariates have a relationship that may be more distal in the causal chain, or are mediated through Level 1 or 2 covariates. The covariates provided to CODEm, as well as their level and direction, differ by cause and sex. The covariates used for GBD 2023, and differences between GBD 2021 and GBD 2023 covariates, can be found in Table 3.

Table 3: Covariates provided to CODEm for each GBD cause, by level and direction, in GBD 2023

Covariate name	Level GBD 2023	Direction GBD 2023
Cumulative Cigarettes (10 Years)	1	1
Cumulative Cigarettes (15 Years) [Data Rich, female]	1	1
Cumulative Cigarettes (20 Years)	1	1
Cumulative Cigarettes (5 Years) [Data Rich, female]	1	1

Liters of alcohol consumed per capita	1	1
Tobacco (cigarettes per capita)	1	1
Age- and sex-specific SEV for High red meat	2	1
Age- and sex-specific SEV for Low fruit	2	1
Age- and sex-specific SEV for Low vegetables	2	1
Healthcare access and quality index	2	-1
Education (years per capita)	3	-1
LDI (I$ per capita)	3	1
Socio-demographic Index	3	1

Incidence estimation

For all cancers except retinoblastoma, the final GBD cancer mortality estimates (after CoDCorrect adjustment) were transformed to incidence estimates by using the MIRs specific to that cancer cause. Final mortality estimates at the draw-specific level were divided by the modelled MIR estimates (also at the draw-specific level) to generate a distribution of draws of incidence estimates (which provides an estimated mean incidence with 95% uncertainty interval). It was assumed that uncertainty in the MIR is independent of uncertainty in the estimated mortality.

Prevalence estimation

After transforming the final GBD cancer mortality estimates to incidence estimates (step 1 in the general cancer flowchart), incidence was combined with annual relative survival estimates from one to ten years after diagnosis (step 7 in the flowchart). Previous reports suggest that the value of (1 – MIR) may serve as a proxy for five-year relative survival, with the exact correlation varying slightly by cancer type.[9] Because this correlation varies, we trained cancer-specific prediction models to estimate five-year survival from MIRs, using data from SEER. We used SEER*Stat[10] to obtain mortality,[11] incidence,[12] and relative survival[12] statistics from the nine SEER registries reporting from 1980 to 2014 (through 2014 so that all years have at least five years of follow-up time; step 2), by cancer type, sex, five-year time periods (eg, 1980–1984, 1985–1989, etc.), and five-year age groups (except combining 80+). For each cancer, we modelled five-year relative survival with MIRs calculated from SEER mortality and incidence, using a generalised linear model with a quasibinomial family and logit link, weighted by the number of index cases (step 3).

To reduce variability due to small samples, we only included MIRs based on at least 25 incident cases (except for the rarer cancers mesothelioma, acute myeloid leukaemia, and acute lymphoid leukaemia, where MIRs based on at least ten cases were included). These models were then applied to the GBD MIR estimates to predict an estimated five-year survival for each age, sex, year, and location combination (step 4). To prevent unrealistic values, predicted five-year survival values were winsorised to be between 0% and 100% survival.

To generate yearly survival estimates up to ten years, we downloaded SEER sex- and age-specific annual one- through ten-year relative survival data from persons diagnosed between 2001 and 2010 (2001 through 2010 so that all cases had at least five years of follow-up, with half having the full ten years of follow-up).[12] A proportional scalar was calculated as the predicted GBD five-year survival estimate divided by the SEER five-year survival statistic, and was then used to generate yearly survival estimates by scaling the one- to ten-year SEER curve to the GBD survival predictions under the proportional hazards assumption (step 5).

To facilitate the estimation of the total person-time survived, the estimated relative survival (the survival of cancer cases relative to those without cancer, given the expected background mortality) described above was next transformed into absolute survival estimates (the overall survival of cancer cases, including all causes of mortality; step 6 and 7 in the flowchart). To account for background mortality in the relative survival estimates, GBD 2023 lifetables were used to calculate lambda (λ) values:

$$\lambda = \frac{\ln\left(\frac{nLx_n}{nLx_{n+1}}\right)}{5}$$

nLx = person-years lived between ages x and x+n (from GBD lifetable).

GBD 2021 lifetables are described elsewhere in the GBD summary papers. Absolute survival was then calculated using an exponential survival function:

$$absolute\ survival = relative\ survival * e^{\lambda * t}$$

t = time (in years)

Absolute survival was combined with incidence to estimate the prevalence at each year after diagnosis.

Disability estimation

To estimate disability for each cancer, total prevalence is split into four sequelae: (1) diagnosis and primary therapy phase; (2) controlled phase; (3) metastatic phase; and (4) terminal phase (step 8 in the flowchart). The diagnosis and primary therapy phase represents the time from the onset of symptoms to the end of initial treatment. The controlled phase represents the time between finishing primary treatment and the earliest of either cure (defined as recurrence- and progression-free survival after ten years); death from another cause; or progression to the metastatic phase. The metastatic phase represents the time period of intensive treatment for metastatic disease, as determined for each cancer by evaluating data from SEER[6] averages (Table 4). The terminal phase represents the one-month period prior to death. Each of these four sequelae has a separate disability weight, which are the same across cancer types (Table 6. Severity distribution). Because of the long-term disability associated with certain treatment-related procedures, additional disability beyond these four sequelae is estimated for five cancers: breast cancer (disability due to mastectomy), larynx cancer (disability due to laryngectomy), colon and rectum cancer (disability due to stoma), bladder cancer (disability due to incontinence from cystectomy), and prostate cancer (disability due to either incontinence or impotence from prostatectomy).

For the purposes of calculating disability due to cancer, survivors beyond ten years were considered cured. For this group, the survivor population prevalence person-time was divided into two sequelae: (1) diagnosis and primary therapy phase; and (2) controlled phase (or remission). For the population that did not survive beyond ten years, the yearly prevalence person-time was divided into four sequelae by assigning fixed durations for each of the: (4) terminal phase, (3) disseminated/metastatic phase, and (1) diagnosis and primary therapy phase, and assigning any remaining prevalence to the (2) controlled phase (step 8 in the flowchart). The duration of these four sequelae remained the same as for GBD 2013, GBD 2015, GBD 2016, GBD 2017, GBD 2019, and GBD 2021,[1–5] with the exception of updates to other malignant neoplasms (recalculated) and other non-Hodgkin lymphoma (set equal to non-Hodgkin lymphoma). Table 4 lists the duration of each, along with the sources used to determine their length. For the diagnosis and primary therapy phase, the duration was taken from primary literature or expert opinion. For the disseminated/metastatic phase, the duration was taken from primary literature, or as the median survival time reported by SEER for the persons described in the note column.

Table 4. Duration of four prevalence sequelae by cancer

Cause	Diagnosis and primary therapy phase (months)*	Controlled phase, or remission	Disseminated/ metastatic phase (months)*	Note for disseminated/ metastatic phase	Terminal phase (months)
Lip and oral cavity cancer	5.3[13]	The remission phase duration is calculated based on the remaining time after attributing other sequelae durations.	9.3[14]	SEER Stage IVc	1.0

* Superscripts refer to references used to inform these values. Durations without a superscript are based on expert opinion.

The final procedure proportions were applied to the incident cases of the respective cancers and multiplied with the proportion of the incident population surviving for ten years to determine the incident cases of the cancer population that underwent procedures and that survived beyond ten years. These

estimates of survivors at ten years were then used as an input for DisMod-MR 2.1, with a remission specification of zero and an excess mortality rate prior of 0 to 0.1, as well as with increasing both the age of the population and the year by ten years to reflect prevalence after that population has survived ten years. The results from this model are incidence and lifetime prevalent cases of persons with these cancer-related sequelae who have survived beyond ten years.

YLD estimation

Lastly, the procedure sequelae prevalence and general sequelae prevalence were multiplied with their respective disability weights (Table 5) to obtain the number of YLDs for each sequela (steps 11 and 12 in the flowchart). The methods used to generate disability weights are described elsewhere in the GBD summary papers. In brief, disability weights were created from survey data to represent the magnitude of health loss associated with an outcome. These disability weights range from 0 (implying a state equivalent to full health) to 1 (a state equivalent to death). Summing these sequelae-specific YLDs then provides the total YLD estimate associated with each cancer cause.

Table 5. Severity distribution. Details on the severity levels for cancers and the associated disability weight with that severity.

Health state	Lay description	Disability weight (95% uncertainty interval)
Cancer, diagnosis and primary therapy *All cancers except nonmelanoma skin cancer*	This person has pain, nausea, fatigue, weight loss and high anxiety.	0.288 (0.193–0.399)
Cancer, controlled phase *All cancers except nonmelanoma skin cancer*	This person has a chronic disease that requires medication every day and causes some worry but minimal interference with daily activities.	0.049 (0.031–0.072)
Cancer, metastatic *All cancers except nonmelanoma skin cancer*	This person has severe pain, extreme fatigue, weight loss, and high anxiety.	0.451 (0.307–0.600)
Terminal phase, with medication *All cancers except nonmelanoma skin cancer*	This person has lost a lot of weight and regularly uses strong medication to avoid constant pain. The person has no appetite, feels nauseous, and needs to spend most of the day in bed.	0.540 (0.377–0.687)

References

1 Global Burden of Disease Cancer Collaboration. The Global Burden of Cancer 2013. *JAMA Oncology* 2015; **1**: 505–27.

2 Global Burden of Disease Cancer Collaboration. Global, Regional, and National Cancer Incidence, Mortality, Years of Life Lost, Years Lived With Disability, and Disability-Adjusted Life-years for 32 Cancer Groups, 1990 to 2015: A Systematic Analysis for the Global Burden of Disease Study. *JAMA Oncology* 2017; **3**: 524–48.

3 Global Burden of Disease Cancer Collaboration. Global, Regional, and National Cancer Incidence, Mortality, Years of Life Lost, Years Lived With Disability, and Disability-Adjusted Life-Years for 29 Cancer Groups, 1990 to 2016: A Systematic Analysis for the Global Burden of Disease Study. *JAMA Oncology* 2018; **4**: 1553–68.

4 Global Burden of Disease Cancer Collaboration. Global, Regional, and National Cancer Incidence, Mortality, Years of Life Lost, Years Lived With Disability, and Disability-Adjusted Life-Years for 29 Cancer Groups, 1990 to 2017: A Systematic Analysis for the Global Burden of Disease Study. *JAMA Oncology* 2019; **5**: 1749–68.

5 Global Burden of Disease Cancer Collaboration, Brian L. Gaw, Rixing Xu, *et al.* Cancer Incidence, Mortality, Years of Life Lost, Years Lived With Disability, and Disability-Adjusted Life Years for 29 Cancer Groups From 2010 to 2019: A Systematic Analysis for the Global Burden of Disease Study 2019. *JAMA Oncology* 2022; **8**: 420–44.

6 Surveillance, Epidemiology, and End Results (SEER) Program (www.Seer.Cancer.Gov) SEER*Stat Database: Incidence - SEER 18.

7 Surveillance, Epidemiology, and End Results (SEER) Program (www.seer.cancer.gov) SEER*Stat Database: Incidence - SEER 18 Regs Research Data + Hurricane Katrina Impacted Louisiana Cases, Nov 2017 Sub (1973-2015 varying) - Linked To County Attributes - Total U.S., 1969-2016 Counties, National Cancer Institute, DCCPS, Surveillance Research Program, released April 2018, based on the November 2017 submission.

8 Barber RM, Fullman N, Sorensen RJD, *et al.* Healthcare Access and Quality Index based on mortality from causes amenable to personal health care in 195 countries and territories, 1990–2015: a novel analysis from the Global Burden of Disease Study 2015. *The Lancet* 2017; **390**: 231–66.

9 Asadzadeh Vostakolaei F, Karim-Kos HE, Janssen-Heijnen MLG, Visser O, Verbeek ALM, Kiemeney LALM. The validity of the mortality to incidence ratio as a proxy for site-specific cancer survival. *Eur J Public Health* 2011; **21**: 573–7.

10 Surveillance Research Program, National Cancer Institute SEER*Stat software (seer.cancer.gov/seerstat) version 8.3.4.

11 Surveillance, Epidemiology, and End Results (SEER) Program (www.seer.cancer.gov) SEER*Stat Database: Mortality - All COD, Aggregated With State, Total U.S. (1969-2015) <Katrina/Rita Population Adjustment>, National Cancer Institute, DCCPS, Surveillance Research Program, released December 2017. Underlying mortality data provided by NCHS (www.cdc.gov/nchs).

12 Surveillance, Epidemiology, and End Results (SEER) Program (www.seer.cancer.gov) SEER*Stat Database: Incidence - SEER 9 Regs Research Data, Nov 2016 Sub (1973-2014) <Katrina/Rita Population Adjustment> - Linked To County Attributes - Total U.S., 1969-2015 Counties, National Cancer Institute, DCCPS, Surveillance Research Program, released April 2017, based on the November 2016 submission.

13 Neal RD, Din NU, Hamilton W, *et al.* Comparison of cancer diagnostic intervals before and after implementation of NICE guidelines: analysis of data from the UK General Practice Research Database. *Br J Cancer* 2014; **110**: 584–92.

14 Surveillance, Epidemiology, and End Results (SEER) Program (www.seer.cancer.gov) SEER*Stat Database: Incidence - SEER 18 Regs Research Data + Hurricane Katrina Impacted Louisiana Cases, Nov 2012 Sub (1973-2010 varying) - Linked To County Attributes - Total U.S., 1969-2011 Counties, National Cancer Institute, DCCPS, Surveillance Research Program, Surveillance Systems Branch, released April 2013, based on the November 2012 submission.

VII. Orofacial clefts

Flowchart

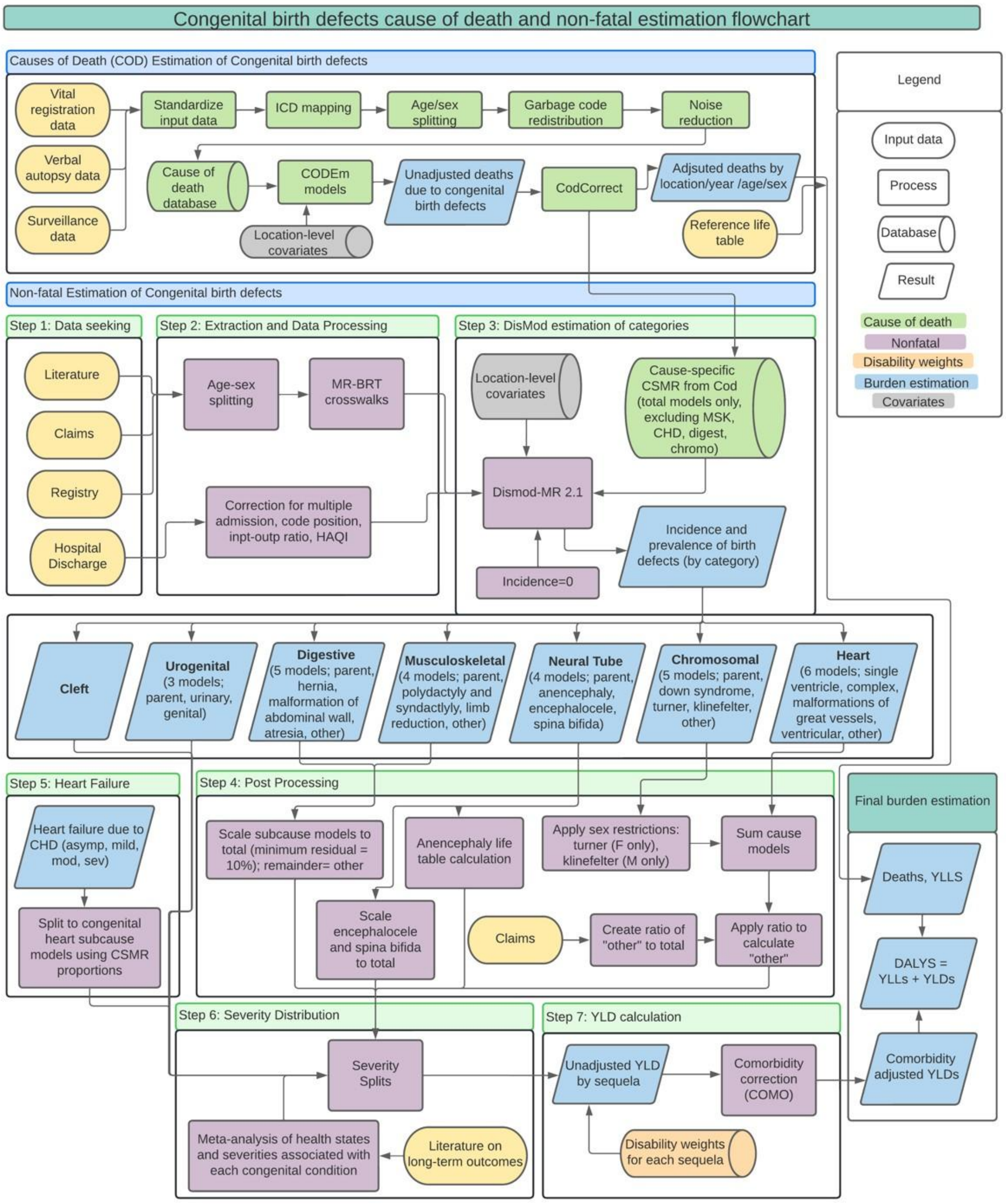

Registry flow chart

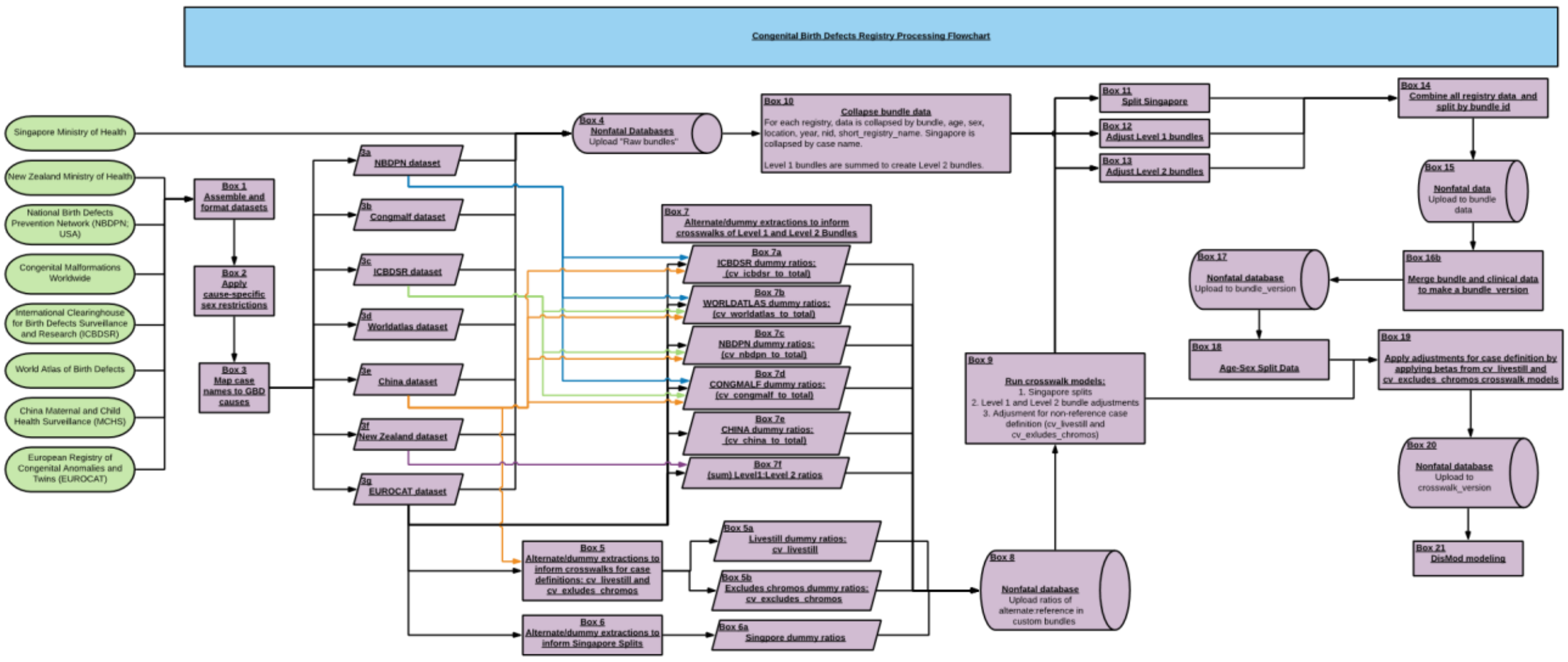

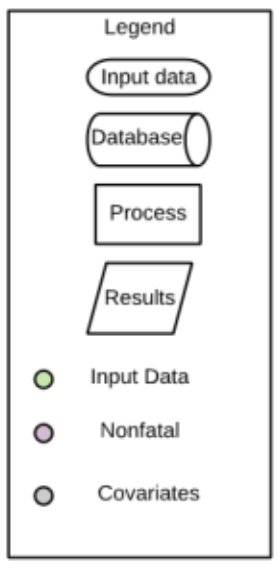

Level 1 (subcause) and Level 2 (parent) bundle list

Congenital muskuloskeletal anomalies
Level 2 bundle:
- 602 - Total muscoskeletal congenital anomalies

Level 1 bundles:
- 604 - Congenital msk - subcause - Limb reduction deficits
- 606 - Congenital msk - subcause - Polydactyly and syndactyly
- 3776 - Congenital msk - subcause - clubfoot
- 3779 - Congenital msk - subcause - hip dysplasia
- 2972 - Other musculoskeletal congenital anomalies

Neural tube defects
Level 2 bundle:
- 608 - Total neural tube defects

Level 1 bundles:
- 610 - Congenital neural - subcause - Anencephaly
- 612 - Congenital neural - subcause - Encephalocele
- 614 - Congenital neural - subcause - Spina Bifida

Congenital muskuloskeletal anomalies
Level 2 bundle:
- 620 - Total congenital digestive anomalies

Level 1 bundles:
- 622 - Congenital digestive - subcause - Congenital diaphragmatic hernia
- 624 - Congenital digestive - subcause - Congenital malformations of the abdominal wall
- 626 - Congenital digestive - subcause - Congenital atresia and/or stenosis of the digestive tract
- 2975 - Other digestive congenital anomalies

Congenital heart disease
Level 2 bundle:
- 628 - Total congenital heart defects

Level 1 bundles:
- 630 - Congenital heart - subcause - Single ventricle and single ventricle pathway heart defects
- 632 - Congenital heart - subcause - Critical malformations of great vessels, congenital valvular heart disease and patent ductus arteriosis
- 634 - Congenital heart - subcause - Ventricular septal defect and atrial septal defect
- 636 - Congenital heart - subcause - Severe congenital heart defects excluding single ventricle and single ventricle pathway
- 2978 - Other congenital cardiovascular defects

Congenital chromosomal anomalies
Level 2 bundle:
- 3029 - Total chromosomal congenital anomalies

Level 1 bundles:
- 436 - Congenital chromo -subcause - Down syndrome
- 437 - Congenital chromo - subcause - Turner syndrome
- 438 - Congenital chromo - subcause - Klinefelter syndrome
- 638 - Congenital chromo - subcause - Edward Syndrome and Patau Syndrome
- 439 - Other chromosomal anomalies, genetic syndrome and micro-deletions

Congenital urogenital anomalies
Level 1 bundles:
616 - Congenital urinary anomalies
618 - Congenital genital anomalies

Orofacial clefts
Level 1 bundle:
435 - Orofacial clefts

Other Level 1 Bundles not currently directly used in congenital birth defects modeling:
3188 - Other congenital birth defects
248 - Congenital cataract
3065 - Congenital hearing loss

Key modelling assumptions:

- Estimates from data sources that excluded chromosomal anomalies were crosswalked to align with estimates from data sources that included such anomalies.
- Incidence was fixed at zero across all ages. Remission was set to zero during the first three months of life. A maximum remission of 0.8 was set for ages 3 months to 2 years. Remission was further constrained to 0–0.07 for ages 2–5 years, 0–0.004 for ages 5–20 years, and 0–0.002 for ages 20–50 years, before being set to zero for individuals aged 50 years and older.
- Log-transformed lag-distributed income, healthcare access and quality index, folic acid per capita, and a composite indicator of fortification standards and folic acid inclusion were included as covariates.

Input data

Several types of data sources are used in the estimation of congenital anomalies: literature prevalence; with-condition mortality and excess mortality data; birth prevalence and neonatal with-condition mortality data from a number of international birth defects registries and surveillance systems; inpatient hospital and MarketScan claims data (a trusted data source) prepared internally by the GBD research team; and cause-specific mortality estimates produced by the causes of death analysis.

First, we extracted data from a number of international birth defects registries. The International Clearinghouse for Birth Defects Surveillance and Research (ICBDSR) reports birth prevalence from a number of international member registries. The World Atlas Report also published birth prevalence estimates from these international registries prior to the publication of ICBDSR reports. EUROCAT reports the birth prevalence of anomalies for a variety of locations in western Europe as reported by participating member registries. China's Maternal and Child Health Surveillance survey (MCHS) reports birth prevalence and early neonatal mortality data for all subnational locations of China. The National Birth Defects Prevention Network (NBDPN) reports birth prevalence estimates as compiled by a number of subnational registries within the USA. The Birth Defects Registry of India (BDRI) reports congenital anomalies from participating hospitals within India.

Second, we used inpatient hospital and claims data (from the USA, Taiwan, and Singapore) for all congenital anomalies causes and sub-cause models. These data were prepared centrally by the Clinical Informatics research team. Four rounds of data bias correction were employed in the processing of clinical data. This included 1) adjustment for readmission, 2) correction of primary diagnoses to all diagnoses, 3) adjustment for inpatient-to-outpatient ratio, and 4) adjustment based on Healthcare Access and Quality Index. Of note, in GBD 2017 we used congenital birth defects data only using the first two corrections but changed starting in GBD 2019 to use clinical data that had all four corrections applied. This change was facilitated by improvements in analysis of corrections by the Clinical Informatics team and was a change made across GBD.

Third, we included data from a systematic review of the available literature for all types of congenital birth defects that was completed in GBD 2015 by constructing search strings designed to capture information on the prevalence, associated mortality, and long-term health outcomes associated with each sub-category of congenital anomalies. All results were screened – first abstracts, then full-text screenings – to ensure the availability of required information and the representativeness of the reported population, and the exclusion of duplicate data also reported as part of the birth registry data inputs. Table 1 summarises the number of primary data sources for orofacial clefts included in GBD 2023 by super-region and year.

Table 1. Number of data sources for orofacial clefts

Period	Central Europe, Eastern Europe, and Central Asia	High-income	Latin America and the Caribbean	North Africa and Middle East	South Asia	Southeast Asia, East Asia, and Oceania	Sub-Saharan Africa
<1990	4	148	6	0	3	6	5
1990-1994	8	150	25	3	3	5	10
1995-1999	30	233	41	13	0	11	17
2000-2004	48	250	28	26	3	24	18
2005-2009	78	307	36	18	9	17	15
2010-2014	51	239	44	20	33	22	27
2015-2019	9	73	6	3	13	6	6
2020-2023	0	6	3	0	0	0	0

Data processing

Age-sex splitting

Starting with GBD 2019, any data that were not sex-specific or did not fit entirely within GBD age groups were age- and sex-split to fit these groups prior to modelling using empirical age- and sex-patterns derived from previous DisMod-MR 2.1 models of the same condition. This is described further below.

Crosswalks in MR-BRT

A number of the input data sources used for the estimation of congenital birth defects are known to have biases leading to under-reporting or over-reporting relative to the true prevalence of congenital anomalies among livebirths and all subsequent age groups. We used meta-regression—Bayesian, regularised, trimmed (MR-BRT)—to develop statistical models that were used to adjust non-reference data. The alternate definitions that were cross-walked are described below. The specifics of each MR-BRT crosswalk are described below (for "registry to total" crosswalks) and in the corresponding cause-specific sections (for live/stillbirth and exclusion of chromosomal conditions crosswalks).

Live/stillbirths: Where necessary, we used a crosswalk to adjust for the inclusion of stillbirths in the reported birth prevalence estimates in literature and registry data sources, as stillbirths are not included in our case definition of prevalence among livebirths. Each of these crosswalks used a log-transformed neonatal mortality rate as a dose–response (spline/linear) covariate in the crosswalks.

Exclusion of chromosomal conditions: Some sources report birth defects in isolation (ie, excluding any persons who have a coexisting genetic or chromosomal disorder). Our reference definition is the inclusion of chromosomal diagnoses. These splines did not consider any additional covariates.

Registry to total: For a subset of congenital causes, particularly congenital heart anomalies, we noted substantial differences in the lists of case definitions being reported to the various congenital registries. For each type of congenital birth defects, registries with the most complete list of reported case definitions – ie, the highest case ascertainment – were used as reference registries and were considered the gold standard for that type of congenital birth defect, or modellable entity. For each modellable entity, we used registry-specific crosswalks to adjust non-gold-standard registries to match the case ascertainment seen in the gold-standard registry. No splines were used in these crosswalks.

Determining outliers and data thresholds

Under-reporting of congenital birth defects is common and can vary by source, location, year, sex, and age. In order to have an empirical, systematic approach to outliering of data, we adapted the non-zero floor approach used by the GBD cause-specific mortality analysis. After all age-sex splitting and crosswalking was complete, the first step was to calculate median absolute deviation (MAD) for the age group of birth, where registry and literature data were combined with all clinical data for the early neonatal age group (0–6 days). The thresholds chosen were –0.5 MAD and +3 MAD, with any data outside of these bounds being identified as outliers. This was determined based on the right-skewed distribution observed in most of the congenital data and the expert prior that under-reporting is far more prevalent than over-reporting – and therefore the bias is asymmetric. In any case where the lower MAD bound was negative, we used a threshold of 0.

For most models, we calculated the MADs using only the EUROCAT data, which we found to be the most reliable source for prevalence of congenital disorders.

To evaluate data for older age groups, we employed two approaches. First, we outliered data from any location-year-source that was outliered for the first stage MAD algorithm. Second, using all clinical and literature data, we developed a model with fixed effects by age to estimate implied MAD bounds for each non-zero age group and again applied the same thresholds of –0.5 MAD and +3 MAD.

Modelling strategy

Overview

We have made no substantive changes in the modelling strategy from GBD 2021. All available input data were utilised in a series of DisMod-MR 2.1 models to estimate the prevalence of each category of congenital anomalies across the full life course for each location/age/sex combination. Incidence was set to zero for all congenital models, as congenital conditions occur at the time of birth and by GBD case definition, congenital cases do not occur after birth. Remission was allowed only in the models of a select subset of causes for which surgical intervention or spontaneous remission can completely

eliminate the disability due to that congenital condition. Cause-specific priors and slope priors were used to guide biologically plausible DisMod-MR 2.1 estimates of excess mortality and remission where applicable.

For most of the congenital birth defects causes, we ran DisMod-MR 2.1 models of all defects combined (termed "parent" models). This allowed us to use data on all anomalies within each cause as well as to leverage CSMR results from the GBD cause of death (CoD) analysis. When CSMR data were used as an input, DisMod-MR 2.1 pairs each CSMR datum with a matching prevalence datapoint by age, sex, location, and year. After matching, CSMR is divided by prevalence to calculate an implied excess mortality rate (EMR) datum. All EMR data are then used in driving the model. Of note, EMR data are not calculated when prevalence data are of broader than GBD age groups or are for both sexes combined.

We used CSMR as input to all of the models except congenital heart anomalies, chromosomal abnormalities, digestive congenital anomalies, congenital musculoskeletal and limb anomalies, and urogenital congenital anomalies.

Location-level covariates

Location-level covariates were used in each of the congenital DisMod-MR 2.1 models based on published information about the risk factors for these birth defects. Folic acid availability was used as a covariate on prevalence for all neural tube defects models and a subset of the congenital musculoskeletal anomalies models. A folic acid fortification covariate was used in the neural tube defects and cleft models, which was modelled based on data from the Global Fortification Data Exchange. The legality of abortion was used as a covariate on prevalence for conditions in which prenatal diagnosis is commonly available and the prognosis is severe enough to cause a high rate of termination of pregnancy following prenatal diagnosis: these include all chromosomal conditions and a subset of the congenital heart anomalies. Maternal consumption of alcohol during pregnancy as a proportion of all pregnancies was used as a covariate on prevalence for all congenital heart defects. The proportion of livebirths by mothers age 35+ was used as a covariate on all chromosomal models. Across many of the congenital models, the Healthcare Access and Quality Index covariate was used to guide the global pattern of with-condition mortality and excess mortality, as was the natural log of the lag-distributed income per capita (LN-LDI). For most of the severe congenital conditions, the mortality associated with the condition is highly dependent on access to adequate surgical interventions and other medical care during the first hours, weeks, and years of life.

Post-model processing

For those causes with a parent model (neural tube defects), we then squeezed the sum of the specific sub-cause prevalence estimates to these total prevalence estimates to ensure internal consistency of our cause-level and sub-cause estimates. The prevalence of other heart, musculoskeletal, and digestive anomalies was derived by reducing the total envelope model for each cause by its sub-causes to derive the difference that was attributable to other anomalies in that category.

Assigning health states and sequelae for long-term outcomes

To determine the distribution of health outcomes associated with the congenital causes, we performed a review of available literature on the long-term health outcomes of survivors in cohorts born with each type of congenital malformation. For conditions requiring surgical intervention shortly after birth to ensure survival, the health states included in the disability weight calculations correspond to the postsurgery outcomes reported in cohorts of individuals born with these life-threatening congenital conditions. Where data were available from multiple cohorts, we pooled these cohorts together to calculate the proportion of individuals with each health state. Where data on the joint distribution of the long-term health outcomes were not available, we assumed independence of each long-term health outcome. Combined disability weights were calculated for all necessary combinations of existing disability weights.

Orofacial clefts

Case definition and associated health states

Orofacial clefts include isolated cleft lip, isolated cleft palate, and combined cleft lip and cleft palate. Cleft lip is an opening in the upper lip that may extend into the nose, and with cleft palate, the roof of the mouth contains an opening into the nose. Both conditions are the result of the tissues of the face not joining properly during development. The GBD case definition of orofacial clefts includes isolated

cleft palate, which corresponds to ICD-10 codes Q35.2, Q35.3, Q35.5, Q35.6, Q35.7, Q35.8, and Q35.9, and cleft palate with or without cleft lip, which corresponds to ICD-10 codes Q36.0, Q36.1, Q36.9, Q37.1, Q37.5, Q37.8, and Q37.9. Craniofacial clefts that do not include the oropharynx are excluded.

These conditions can be successfully treated by surgery, which is typically done during the first few months or years of life but may occasionally be completed later in life. The sequelae associated with orofacial clefts are disfigurement level 1, disfigurement level 2, and disfigurement level 2 with speech problems. Additionally, a proportion of the population with orofacial clefts is considered to be asymptomatic. In the absence of data, we assumed the proportion of each is equal.

Crosswalks

The MR-BRT crosswalk results are shown below.

Table 1: MR-BRT crosswalk betas for alternate definitions (reference = livebirths including those with chromosomal anomalies)

Crosswalk	Beta	Standard error
Excluding chromosomal diagnoses adjustment	–0.055	0.012

Figure 1: MR-BRT crosswalk of alternate definition (livebirths and stillbirths included) with spline on logtransformed neonatal mortality rate

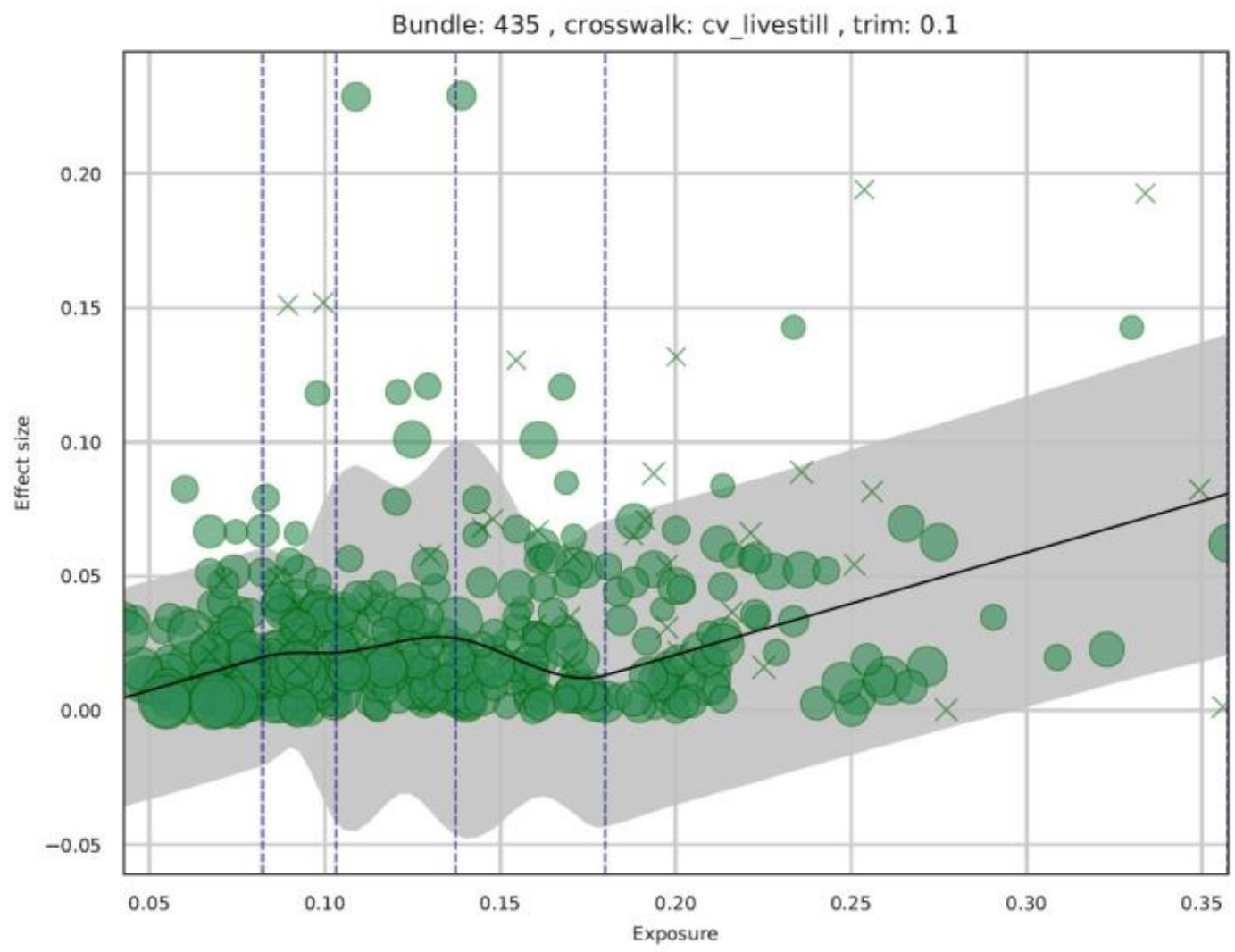

Modelling strategy

The DisMod-MR 2.1 model of orofacial clefts had random effects on prevalence limited to ±0.8, as we expected limited variation in birth prevalence of orofacial clefts. The model settings allow increased smoothness on both EMR and remission (maximum xi=5.0) to fit steep changes in the rates mortality and remission during the first few years of life.

Incidence was set to zero for all ages. Remission was set to zero for the first three months of life, as cleft lip and/or palate are rarely corrected in the first few months of life. A maximum remission of 0.8 was set for ages 3 months to 2 years, the age range in which cleft repair is most commonly performed, allowing up to 75% of cleft cases to be repaired between 3 months and 2 years of age. Remission was bounded from 0 to 0.07 for ages 2–5 years, 0–0.004 for ages 5–20 years, then bounded from 0–0.002 for ages 20– 50 years, and set at zero for ages 50+ years. These limits on remission reflect our priors that up to 20% of remaining cleft cases are repaired between 2 and 5 years of age, another 5% may be repaired between 5 and 20 years of age, and a maximum 5% of remaining cases are surgically repaired between ages 20 and 50 years.

Priors on EMR were set at a maximum of 2.5 for the early neonatal period, 0.01 for ages 5–10, and 0.000001 for ages 10+. These limits on excess mortality reflect our priors that up to 5% of individuals with orofacial clefts die in the first week of life, up to 5% die in the following three weeks, up to 20% die in the next 11 months, another maximum of 20% before 5 years of ages, and a maximum of 5% of the remaining individuals die between ages 5 and 10 years.

Table 2: Location-level covariate effects

Covariate name	Measure	Beta value	Exponentiated value
Healthcare Access and Quality Index	Prevalence	–0.000097 (–0.00019 to – 0.000015)	1.00 (1.00–1.00)
Folic acid, unadjusted (μg)	Prevalence	–0.00016 (–0.00026 to –0.000071)	1.00 (1.00–1.00)
Composite fortification standard and folic acid inclusion	Prevalence	–0.0077 (–0.014 to –0.0015)	0.99 (0.99–1.00)
LN-LDI (I$ per capita)	EMR	–0.75 (–0.75 to –0.75)	0.47 (0.47–0.47)

GATHER checklist

Checklist of information that should be included in new reports of global health estimates

Item #	Checklist item	Reported on page #
**Objectives and funding**		
**1**	Define the indicator(s), populations (including age, sex, and geographic entities), and time period(s) for which estimates were made.	6
**2**	List the funding sources for the work.	2
**Data Inputs**		
For all data inputs from multiple sources that are synthesized as part of the study:		
**3**	Describe how the data were identified and how the data were accessed.	6 and Appendix 1
**4**	Specify the inclusion and exclusion criteria. Identify all ad-hoc exclusions.	Appendix 1
**5**	Provide information on all included data sources and their main characteristics. For each data source used, report reference information or contact name/institution, population represented, data collection method, year(s) of data collection, sex and age range, diagnostic criteria or measurement method, and sample size, as relevant.	Appendix 1
**6**	Identify and describe any categories of input data that have potentially important biases (e.g., based on characteristics listed in item 5).	Appendix 1
For data inputs that contribute to the analysis but were not synthesized as part of the study:		
**7**	Describe and give sources for any other data inputs.	Appendix 1
For all data inputs:		
**8**	Provide all data inputs in a file format from which data can be efficiently extracted (e.g., a spreadsheet rather than a PDF), including all relevant meta-data listed in item 5. For any data inputs that cannot be shared because of ethical or legal reasons, such as third-party ownership, provide a contact name or the name of the institution that retains the right to the data.	Accessible from: https://ghdx.healthdata.org/gbd-results-tool
**Data analysis**		
**9**	Provide a conceptual overview of the data analysis method. A diagram may be helpful.	Appendix 1
**10**	Provide a detailed description of all steps of the analysis, including mathematical formulae. This description should cover, as relevant, data cleaning, data pre-processing, data adjustments and weighting of data sources, and mathematical or statistical model(s).	Appendix 1
**11**	Describe how candidate models were evaluated and how the final model(s) were selected.	Appendix 1
**12**	Provide the results of an evaluation of model performance, if done, as well as the results of any relevant sensitivity analysis.	Appendix 1
**13**	Describe methods for calculating uncertainty of the estimates. State which sources of uncertainty were, and were not, accounted for in the uncertainty analysis.	Page 7
**14**	State how analytic or statistical source code used to generate estimates can be accessed.	Page 8
**Results and Discussion**		
**15**	Provide published estimates in a file format from which data can be efficiently extracted.	Accessible from: https://gh

		dx.healthdata.org/gbd-results-tool
**16**	Report a quantitative measure of the uncertainty of the estimates (e.g. uncertainty intervals).	Pages 8-10, Tables & Figures
**17**	Interpret results in light of existing evidence. If updating a previous set of estimates, describe the reasons for changes in estimates.	10-11
**18**	Discuss limitations of the estimates. Include a discussion of any modelling assumptions or data limitations that affect interpretation of the estimates.	Page 11 and Appendix 1

This checklist should be used in conjunction with the GATHER statement and Explanation and Elaboration document, found on gather-statement.org

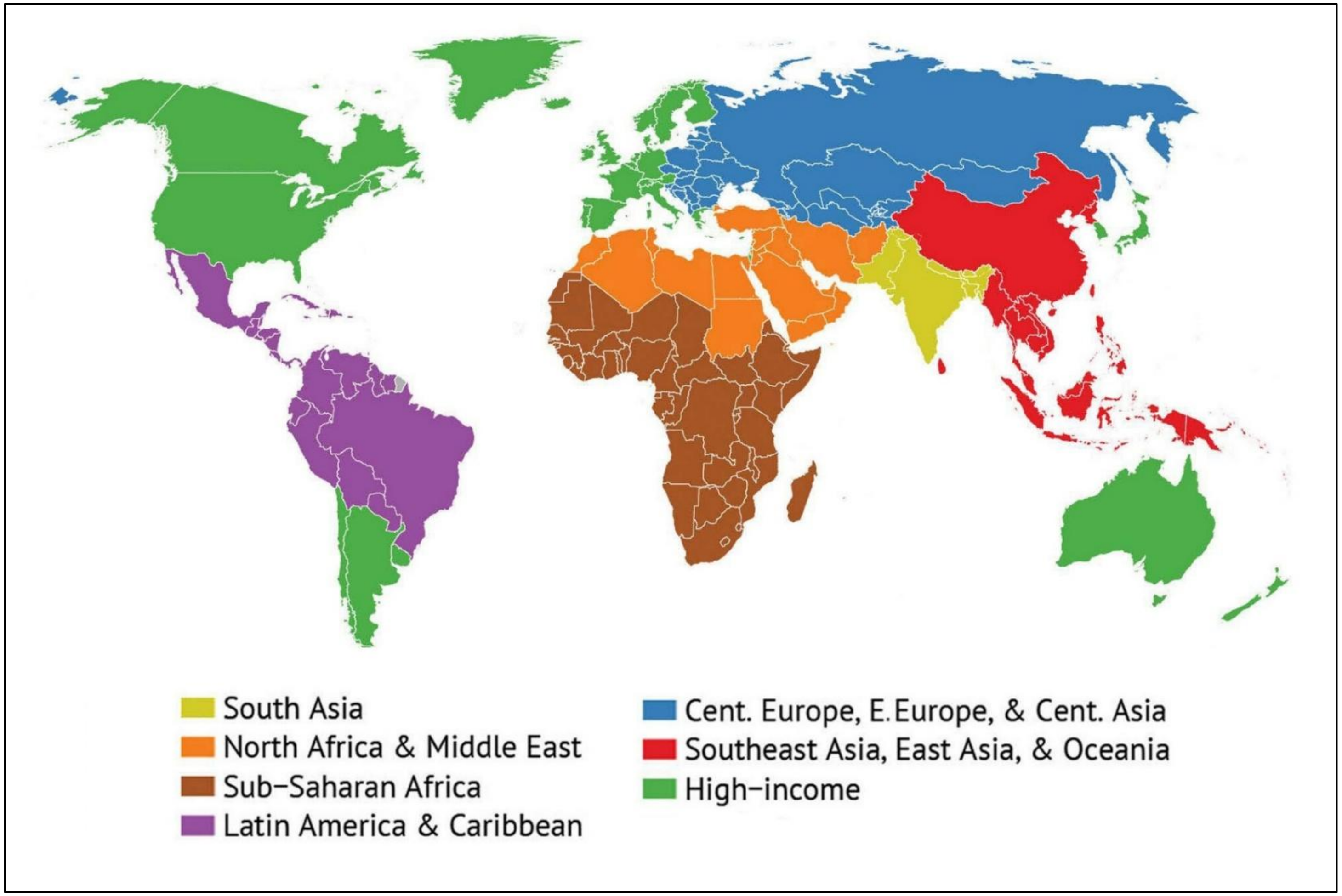

Figure S1. World map of GBD super-regions and their constituent geographies. The grouping of countries reflects standard GBD analytic super-regions, not policy or WHO regions.

Table S1. Prevalent or incident cases in 2019 and 2023 and percent changes over 2019-2023 for oral conditions, by super-regions.

	Prevalent or incident cases in 2019, millions (95% UI)	Prevalent or incident cases in 2023, millions (95% UI)	Percent change 2019-2023 (95% UI)
**Main oral conditions**[a]			
Central Europe, Eastern Europe and Central Asia	233 (216, 253)	232 (214, 251)	-0·61 (-1·38, 0·14)
High-income	491 (457, 528)	506 (471, 543)	2·91 (2·28, 3·54)
Latin America and the Caribbean	305 (283, 328)	308 (286, 333)	1·15 (-0·46, 2·54)
North Africa and Middle East	303 (276, 335)	319 (290, 351)	5·26 (4·22, 6·37)
South Asia	899 (825, 978)	919 (842, 1000)	2·24 (0·49, 3·86)
Southeast Asia, East Asia and Oceania	942 (861, 1030)	958 (878, 1050)	1·68 (0·79, 2·42)
Sub-Saharan Africa	449 (408, 498)	492 (451, 543)	9·51 (8·09, 10·94)
**Untreated caries of deciduous teeth**			
Central Europe, Eastern Europe and Central Asia	25·2 (20·8, 29·9)	23·4 (19·4, 27·8)	-7·06 (-8·54, -5·5)
High-income	33·8 (27·2, 41·3)	35·5 (28·7, 42·7)	4·79 (2·24, 7·33)
Latin America and the Caribbean	35·8 (29·3, 42·5)	33 (27·7, 38·3)	-7·74 (-14·13, 0·61)
North Africa and Middle East	48·7 (41·2, 57·1)	46·5 (39·1, 54·4)	-4·6 (-7, -1·84)
South Asia	145 (122, 173)	128 (107, 147)	-11·67 (-20·05, -2·76)
Southeast Asia, East Asia and Oceania	132 (110, 157)	127 (106, 152)	-4·24 (-6·85, -1·74)
Sub-Saharan Africa	112 (92·1, 133)	115 (95·4, 134)	3·04 (-1·67, 7·63)
**Untreated caries of permanent teeth**			
Central Europe, Eastern Europe and Central Asia	152 (131, 173)	150 (130, 171)	-1·28 (-2·59, 0·04)
High-income	298 (267, 333)	304 (273, 339)	2·06 (0·75, 3·32)
Latin America and the Caribbean	198 (175, 226)	197 (172, 228)	-0·25 (-1·89, 1·29)
North Africa and Middle East	203 (172, 239)	215 (183, 251)	5·92 (4·01, 7·79)
South Asia	554 (479, 653)	569 (491, 665)	2·65 (1·13, 4·33)
Southeast Asia, East Asia and Oceania	525 (447, 622)	525 (448, 623)	0·12 (-1·53, 1·61)

Sub-Saharan Africa	271 (231, 319)	301 (257, 353)	11·07 (9·96, 12·22)
**Severe periodontitis**			
Central Europe, Eastern Europe and Central Asia	58·7 (45·9, 72·8)	59·1 (46·3, 73·3)	0·79 (-0·09, 1·67)
High-income	151 (126, 175)	153 (126, 180)	1·47 (0·21, 2·51)
Latin America and the Caribbean	83·8 (72·1, 98·1)	87·2 (72·9, 102)	4·02 (-1·7, 11)
North Africa and Middle East	66·1 (52·9, 81·7)	71·9 (57·4, 89)	8·75 (7·48, 10·33)
South Asia	296 (249, 342)	320 (267, 371)	7·92 (6·55, 9·3)
Southeast Asia, East Asia and Oceania	293 (242, 347)	307 (255, 360)	4·82 (3·68, 5·98)
Sub-Saharan Africa	85·1 (70·4, 101)	94·9 (78·5, 112)	11·49 (10·55, 12·34)
**Edentulism**			
Central Europe, Eastern Europe and Central Asia	38·7 (34, 45)	41·4 (36·1, 48)	6·97 (5·1, 9·32)
High-income	80·2 (65·9, 97·7)	87·9 (72·6, 107)	9·56 (8·8, 10·42)
Latin America and the Caribbean	44 (38·7, 49·9)	48·8 (42·6, 55·4)	10·73 (9·96, 11·59)
North Africa and Middle East	27 (22·6, 32·7)	30·7 (25·7, 37)	13·85 (12·31, 15·11)
South Asia	39·3 (32·5, 47·6)	43 (37·2, 50·7)	9·46 (0·20, 21·3)
Southeast Asia, East Asia and Oceania	90 (75·6, 109)	103 (87, 124)	14·07 (13, 15·02)
Sub-Saharan Africa	16·7 (13·3, 20·3)	18·7 (15, 22·8)	12·24 (11·23, 13·32)
**Other oral disorders**			
Central Europe, Eastern Europe and Central Asia	8·59 (8·25, 8·94)	8·59 (8·26, 8·95)	-0·01 (-0·28, 0·25)
High-income	22 (21·3, 22·9)	22·4 (21·6, 23·2)	1·68 (1·59, 1·78)
Latin America and the Caribbean	11·2 (10·7, 11·6)	11·5 (11·1, 12)	3·31 (3·15, 3·45)
North Africa and Middle East	11·1 (10·6, 11·6)	11·9 (11·3, 12·3)	6·6 (6·36, 6·82)
South Asia	33·5 (32·2, 35)	35·5 (34·1, 37·2)	6·18 (6·04, 6·32)
Southeast Asia, East Asia and Oceania	43·5 (41·7, 45·4)	44·5 (42·7, 46·5)	2·39 (2·06, 2·71)
Sub-Saharan Africa	17·9 (17·2, 18·7)	20·1 (19·2, 21)	12·02 (11·91, 12·14)
**Lip and oral cavity cancer**			

Central Europe, Eastern Europe and Central Asia	0·029 (0·026, 0·0318)	0·0283 (0·0252, 0·0313)	-2·39 (-7·66, 3·36)
High-income	0·0881 (0·0767, 0·102)	0·0969 (0·0833, 0·111)	9·92 (1·74, 18·63)
Latin America and the Caribbean	0·0156 (0·0142, 0·017)	0·017 (0·0153, 0·0186)	8·7 (3·41, 15·19)
North Africa and Middle East	0·00601 (0·0048, 0·00762)	0·00718 (0·00563, 0·00908)	19·54 (7·41, 34·1)
South Asia	0·139 (0·111, 0·173)	0·177 (0·14, 0·228)	27·36 (11·96, 41·55)
Southeast Asia, East Asia and Oceania	0·071 (0·0622, 0·0821)	0·0846 (0·0729, 0·0988)	19·12 (9·48, 30·36)
Sub-Saharan Africa	0·0143 (0·0112, 0·0185)	0·0189 (0·0148, 0·0239)	32·12 (18·25, 47·44)
**Orofacial clefts**			
Central Europe, Eastern Europe and Central Asia	0·161 (0·13, 0·192)	0·164 (0·133, 0·195)	1·79 (-0·37, 3·86)
High-income	0·29 (0·235, 0·352)	0·289 (0·232, 0·355)	-0·27 (-2·85, 1·59)
Latin America and the Caribbean	0·203 (0·167, 0·244)	0·204 (0·166, 0·247)	0·64 (-1·73, 2·94)
North Africa and Middle East	0·522 (0·427, 0·637)	0·549 (0·449, 0·669)	5·19 (2·43, 7·92)
South Asia	1·6 (1·27, 1·98)	1·7 (1·35, 2·11)	6·19 (3·58, 8·59)
Southeast Asia, East Asia and Oceania	0·72 (0·589, 0·872)	0·74 (0·595, 0·904)	2·81 (0·61, 4·63)
Sub-Saharan Africa	0·556 (0·446, 0·67)	0·614 (0·492, 0·747)	10·54 (8·53, 12·88)

Main oral conditions include untreated caries, severe periodontitis, edentulism, and other oral disorders only. Prevalent cases of main oral conditions do not match the sum of prevalent cases of individual conditions because some conditions coexist. Data are shown as prevalence for all oral conditions, except lip and oral cavity cancer, which are shown as incidence. Super-regions with rising numbers of prevalent or incident cases but declining age-standardised prevalence or incidence are shown in grey. All data are presented to three significant figures. UI=uncertainty interval.

Table S2. Age-standardized prevalence or incidence in 2019 and 2023 and percent changes over 2019-2023 for oral conditions, by super-regions.

	Age-standardised prevalence/incidence[b] in 2019, per 100,000 (95% UI)	Age-standardised prevalence/incidence[b] in 2023, per 100,000 (95% UI)	Percent change 2019-2023 (95% UI)
**Main oral conditions[a]**			
Central Europe, Eastern Europe and Central Asia	51600 (47500, 56500)	51100 (47100, 55900)	-0·94 (-1·63, -0·22)
High-income	40200 (37000, 43600)	40700 (37500, 44100)	1·24 (0·56, 1·96)
Latin America and the Caribbean	51500 (48000, 55500)	50300 (46600, 54400)	-2·37 (-4·01, -1·01)
North Africa and Middle East	51000 (46800, 55900)	50500 (46200, 55200)	-1·12 (-2·09, -0·12)
South Asia	50900 (46800, 54900)	49200 (45400, 53400)	-3·34 (-4·95, -1·80)
Southeast Asia, East Asia and Oceania	41700 (38100, 46000)	41100 (37500, 45200)	-1·44 (-2·09, -0·79)
Sub-Saharan Africa	45100 (41500, 49100)	44400 (40900, 48500)	-1·52 (-2·35, -0·66)
**Untreated caries of deciduous teeth**			
Central Europe, Eastern Europe and Central Asia	9020 (7450, 10700)	8840 (7330, 10400)	-2·03 (-3·59, -0·41)
High-income	5450 (4390, 6610)	5950 (4830, 7120)	9·21 (6·60, 11·89)
Latin America and the Caribbean	7400 (6070, 8780)	7130 (6000, 8250)	-3·59 (-10·15, 4·94)
North Africa and Middle East	7530 (6360, 8810)	7350 (6190, 8580)	-2·39 (-4·79, 0·30)
South Asia	8110 (6800, 9610)	7380 (6180, 8440)	-9·09 (-17·62, -0·08)
Southeast Asia, East Asia and Oceania	8500 (7030, 10000)	8430 (7010, 9960)	-0·72 (-1·75, 0·50)
Sub-Saharan Africa	6810 (5590, 8100)	6600 (5470, 7690)	-3·11 (-7·51, 1·31)
**Untreated caries of permanent teeth**			
Central Europe, Eastern Europe and Central Asia	33200 (28800, 38400)	32800 (28400, 38100)	-1·29 (-2·41, 0·15)
High-income	25000 (22100, 28800)	25100 (22200, 28700)	0·06 (-1·04, 1·18)
Latin America and the Caribbean	32600 (29000, 37200)	31500 (27500, 36500)	-3·54 (-5·04, -2·36)
North Africa and Middle East	33800 (28900, 39000)	33400 (28700, 38500)	-1·24 (-3·05, 0·58)

South Asia	30800 (26900, 35900)	29600 (25700, 34400)	-3·77 (-5·18, -2·33)
Southeast Asia, East Asia and Oceania	22900 (19400, 27600)	22400 (19000, 27000)	-2·32 (-3·59, -1·06)
Sub-Saharan Africa	28600 (24900, 33100)	28100 (24600, 32500)	-1·51 (-2·58, -0·38)
**Severe periodontitis**			
Central Europe, Eastern Europe and Central Asia	10400 (8020, 13100)	10300 (7870, 13000)	-1·07 (-2·78, 0·97)
High-income	9730 (7970, 11500)	9590 (7680, 11500)	-1·45 (-2·16, -0·74)
Latin America and the Caribbean	13400 (11500, 15700)	13100 (11000, 15400)	-2·17 (-2·85, -1·44)
North Africa and Middle East	11800 (9580, 14300)	11500 (9380, 14000)	-1·78 (-3·14, -0·65)
South Asia	17500 (14800, 20100)	17300 (14500, 19900)	-1·09 (-1·62, -0·46)
Southeast Asia, East Asia and Oceania	10500 (8710, 12500)	10400 (8580, 12200)	-1·38 (-6·71, 5·55)
Sub-Saharan Africa	11900 (10000, 13800)	11700 (9780, 13600)	-2·07 (-3·5, -1·00)
**Edentulism**			
Central Europe, Eastern Europe and Central Asia	6120 (5400, 7080)	6270 (5520, 7210)	2·43 (1·47, 3·42)
High-income	4000 (3320, 4830)	4120 (3420, 4960)	2·91 (-5·32, 13·94)
Latin America and the Caribbean	7370 (6480, 8350)	7350 (6430, 8330)	-0·27 (-1·49, 0·91)
North Africa and Middle East	5970 (5030, 7150)	5950 (5010, 7100)	-0·35 (-1·22, 0·44)
South Asia	2910 (2460, 3520)	2860 (2480, 3350)	-1·75 (-2·59, -0·84)
Southeast Asia, East Asia and Oceania	3390 (2850, 4100)	3360 (2850, 4030)	-0·88 (-1·69, -0·12)
Sub-Saharan Africa	2880 (2350, 3440)	2850 (2340, 3390)	-1·29 (-2·07, -0·54)
**Other oral disorders**			
Central Europe, Eastern Europe and Central Asia	1860 (1790, 1940)	1860 (1790, 1940)	-0·03 (-0·08, 0·03)
High-income	1850 (1790, 1920)	1850 (1780, 1920)	0·05 (0·03, 0·07)
Latin America and the Caribbean	1860 (1790, 1940)	1860 (1790, 1940)	0·01 (-0·01, 0·03)
North Africa and Middle East	1850 (1770, 1920)	1850 (1770, 1920)	-0·02 (-0·06, 0·02)
South Asia	1860 (1790, 1940)	1860 (1790, 1940)	0 (-0·03, 0·02)

Southeast Asia, East Asia and Oceania	1860 (1790, 1940)	1860 (1780, 1940)	-0·03 (-0·06, 0·02)
Sub-Saharan Africa	1860 (1780, 1940)	1860 (1780, 1940)	0·01 (-0·02, 0·03)
**Lip and oral cavity cancer**			
Central Europe, Eastern Europe and Central Asia	4·64 (4·15, 5·09)	4·38 (3·9, 4·85)	-5·56 (-13·94, 2·97)
High-income	4·54 (3·96, 5·25)	4·7 (4·07, 5·4)	3·52 (-6·12, 14·41)
Latin America and the Caribbean	2·6 (2·35, 2·83)	2·56 (2·31, 2·8)	-1·57 (-9·41, 8·12)
North Africa and Middle East	1·35 (1·08, 1·72)	1·42 (1·13, 1·8)	5·68 (-8·59, 21·42)
South Asia	9·38 (7·49, 11·7)	10·7 (8·5, 13·8)	14·33 (0·47, 29·05)
Southeast Asia, East Asia and Oceania	2·58 (2·25, 2·98)	2·77 (2·38, 3·24)	7·48 (-3·56, 18·66)
Sub-Saharan Africa	2·57 (1·99, 3·32)	2·95 (2·3, 3·73)	14·86 (1·19, 32·45)
**Orofacial clefts**			
Central Europe, Eastern Europe and Central Asia	41·3 (33·6, 49·2)	42·7 (34·6, 50·3)	3·44 (1·2, 5·56)
High-income	28·1 (22·8, 34·1)	27·7 (22·3, 34·1)	-1·51 (-4·03, 0·28)
Latin America and the Caribbean	35·7 (29·4, 42·9)	35·6 (29·1, 43)	-0·44 (-2·78, 1·82)
North Africa and Middle East	85·5 (69·9, 104)	86·1 (70·4, 105)	0·69 (-1·83, 3·43)
South Asia	88·5 (70·7, 110)	90·4 (72·1, 113)	2·18 (-0·33, 4·47)
Southeast Asia, East Asia and Oceania	34·7 (28·4, 41·9)	35·6 (28·8, 43·2)	2·61 (0·42, 4·75)
Sub-Saharan Africa	48·3 (38·6, 59)	48·6 (39, 59·6)	0·55 (-1·36, 2·65)

Main oral conditions include untreated caries, severe periodontitis, edentulism, and other oral disorders only. Prevalent cases of main oral conditions do not match the sum of prevalent cases of individual conditions because some conditions coexist. Data are shown as prevalence for all oral conditions, except lip and oral cavity cancer, which are shown as incidence. Super-regions with rising numbers of prevalent or incident cases but declining age-standardised prevalence or incidence are shown in grey. All data are presented to three significant figures. UI=uncertainty interval.

GBD 2023 Oral Disorders Collaborators

Eduardo Bernabe*, Wagner Marcenes, Abdullah, Hazim S Ababneh, Belayneh Jejaw Abate, Hedayat Abbastabar, Wael M Abdel-Rahman, Sherief Abd-Elsalam, Hassan A. Abdou, Meriem Abdoun, Rizwan Suliankatchi Abdulkader, Syed Hani Abidi, Richard Gyan Aboagye, Lucas Guimarães Abreu, Sawsan Abuhammad, Anirudh Balakrishna Acharya, Swetha Acharya, Lawan Hassan Adamu, Tajudeen Adesanmi Adebisi, Babatope Oluwadamilare Adebiyi, Kamoru Ademola Adedokun, Aanuoluwapo Adeyimika Afolabi, Muhammad Sohail Afzal, Percival Delali Agordoh, Salome Wumpini Agordoh, Trushdeep Agrawal, Piyush Agrawal, Tauseef Ahmad, Sajjad Ahmad, Aqeel Ahmad, Suhaib Ahmad, Khabir Ahmad, Ali Ahmadi, Naveed Ahmed, Shakeel Ahmed, Ali Ahmed, Ayman Ahmed, Kenni Wojujutari Ajele, Kasuni Hm Akalanka, Sara Akeel, Damilare Michael Akintunde, Hammad Akram, Chisom Joyqueenet Akunna, Timothy Olukunle Aladelusi, Yazan Al-Ajlouni, Mohammed Sultan Al-Ak'hali, Mohammad Khursheed Alam, Mostafa Alam, Ruaa A Alamoudi, Amer Hashim Al Ani, Salah Al Awaidy, Mohammed Albashtawy, Mohammed S Aldossary, Abdelazeem M Algammal, Saeed M. M Alghamdi, Mohammed Ahmed Alghauli, Amidu Alhassan, Ashraf Alhumaidi, Taha Hussein Hussein Ali, Kamran Ali, Syed Shujait Ali, Montaha Al-Iede, Morteza Alipour, Behrouz Alizadeh Savareh, Sabah Al-Marwani, Sadeq A Al-Maweri, Essam Ahmed Al-Moraissi, Mohmmad Minwer Alnaeem, Khaldoon Aied Alnawafleh, Margret Beaula Alocious Sukumar, Nivaldo Alonso, Mohammad Alqudah, Mohammad R Alqudimat, Ahmed Yaseen Alqutaibi, Mustafa Haleem Alsaadi, Mohammed Amjed Alsaegh, Anas Alsalhani, Ali Salman Al-Shami, Zaid Altaany, Awais Altaf, Hany Aly, Karem H Alzoubi, Nada A Alzunaidy, Uchenna Anderson Amaechi, Mcking Izeiza Amedari, Priscilla O Ameh, Junaid Amin, Saeed Amini, Mohammad Hosein Amirzade-Iranaq, Hubert Amu, Abhishek Anil, A.h. Sruthi Anil Kumar, Kabilan Annadurai, Saeid Anvari, Anayochukwu Edward Anyasodor, Jalal Arabloo, Mosab Arafat, Gururaj Arakeri, Aleksandr Y Aravkin, Demelash Areda, Jorge Arias De La Torre, Mohammad Saquib Ashraf, Javed Ashraf, Tahira Ashraf, Esma Asil, Saira Atif, Samuel Babajide Atoyebi, Nyan Min Aung, Manal Awad, Usman Ayub Awan, Sana Javaid Awan, Ashish Awasthi, Aneesa Ayoob, Somayyeh Azimi, Domenico Azzolino, Muhammad Badar, Huma Bader Ul Ain, Ashish D Badiye, Riadh Badraoui, Khlood K Baghlaf, Dania Bahdila, Mohammad Hassan Baig, Atif Amin Baig, Mohamad Amin Bakhshali, Marina Balabekova, Raju Balaji, Wondu Feyisa Balcha, Abdulrahman A. A Balhaddad, Yatan Pal Singh Balhara, Maciej Banach, Morteza Banakar, Salam Bani Hani, Palash Chandra Banik, Farhat Bano, Md. Abul Barkat, Rocío Barrios-Rodríguez, Amadou Barrow, Shahid Bashir, Azadeh Bashiri, Mohammad-Mahdi Bastan, Hagar Abd El Naby Bastawy, Kavita Batra, Oyundari Batsaikhan, Babak Behnam, Almaz Nibret Belay, Enku Shiferaw Belayneh, Nahom Girma Belete, Uzma Iqbal Belgaumi, Ibrahim Olajide Bello, Ghaith Benhsen, Alemshet Yirga Berhie, Ghalia Y Bhadila, Pooja Bhadoria, Pankaj Bhardwaj, Sonu Bhaskar, Ajay Nagesh Bhat, Ashok Kumar Bhati, Nada Binmadi, Tone Bjørge, Trupti Bodhare, Srinivasa Rao Bolla, Souad Bouaoud, Abdelhakim Bouyahya, Christopher Boxe, Maria Laura Braccini Fagundes, Dejana Braithwaite, Nikolay Ivanovich Briko, Michael Eric Bryan, Lin-Lin Bu, Sayed Muhammad Ata Ullah Shah Bukhari, Yu Cao, Giulia Carreras, Andrea Carugno, Sandra Carvalho, Felix Carvalho, Pamela Roxana Chacón-Uscamaita, Sandip Chakraborty, Vijay Kumar Chattu, Anis Ahmad Chaudhary, Sirshendu Chaudhuri, Akhilanand Chaurasia, Junhao Chen, Meng Xuan Chen, Mirza Farhana Iqbal Chowdhury, Dinh-Toi Chu, Sanjay C J, Natalia Cruz-Martins, Ferran Cuenca-Martínez, Omid Dadras, Namrata Dagli, Lina Dahabiyeh, Neha Dahiya, Mouni Damineni, Samuel Demissie Darcho, Riya Dave, Mauro Henrique Nogueira Guimarães De Abreu, Emina Dervišević, Premnath Dhanaraj, Amol S Dhane, Antonio Di Guardo, Elangovan Dilipan, Sadia Din, Orlando Luiz Do Amaral Júnior, Sushil Dohare, Cátia Domingues, Francesco Dondi, Lingkang Dong, Ojas Prakashbhai Doshi, Taiwo Omotayo Dosumu, Jiang Du, Mi Du, Vikash Kumar Dubey, Manisha Dubey, Siddhartha Dutta, Arkadiusz Marian Dziedzic, Venkata Ravikanth Eddula, Aziz Eftekharimehrabad, Ebrahim Eini, Michael Ekholuenetale, Seraphine Mojoko Eko, Abdelbaset Mohamed Elasbali, Amine Elbouzidi, Mohamed Elhoumed, Omar Abdelsadek Abdou Elmeligy, Ashraf A El-Metwally, Rami

Elmorsi, Abdelfatteh EL Omri, Iman El Sayed, Nadia Bassuoni Elsharkawy, Abdelgawad Salah Eltahawy, Maha El Tantawi, Heba Emad, Purushoth Ethiraj, Devaraj Ezhilarasan, Amanda Ugochi Ezirim, Adeniyi Francis Fagbamigbe, Ayesha Fahim, Nour Fakih, Ebrahim Farashi, Muhammad Amber Fareed, Mohsen A Farghaly, Anees Ahmed Farooq Mohammed, Fatemeh Farshad, Alireza Feizkhah, Guangshou Feng, Maria Pia Ferraz, Nuno Ferreira, Getahun Fetensa, Tommaso Filippini, Florian Fischer, James L Fisher, Carlos Forner Álvarez, Delfin Lovelina Francis, Takeshi Fukumoto, Peter Andras Gaal, Amol Ramchandra Gadbail, Piyada Gaewkhiew, Márió Gajdács, Silvano Gallus, Fernando Jr. Barroga Garcia, Tsion S Gebre, Nsikakabasi Samuel George, Ahmad Ghashghaee, Sama Ghoba, Nasim Gholizadeh, Mahsa Ghorbani, Elena Ghotbi, Thinh Gia Gia Nguyen, Paramjit Singh Gill, Anil Kumar Goel, Marília Goettems, Awais Gohar, Gamze Gökulu, Pouya Goleij, Mohsen Golkar, Shailesh Madhukar Gondivkar, Divya Gopinath, Giuseppe Gorini, Barbara Niegia Garcia Goulart, Changhao Gu, Giovanni Guarducci, Bhuvaneshwari Gunasekar, Sunanda Gupta, Swati Gupta, Bhawna Gupta, Sapna Gupta, Bruno Gutiérrez, Heliasadat Haeriboroojeni, Khalid Rehman Hakeem, Esam Halboub, Pritam Halder, Ibrahim Ismael Hamarash, Raz Mohammed Hama Salih, Mohamed Hamed, Samer Hammoudeh, Abdelrahman M Hamouda, Mohd Anul Haq, Faizul Hasan, Shamimul Hasan, Towhid Hasan, Hamidreza Hasani, Nada Tawfig Hashim, Ahmad Shoeb Hashmi, Md Saquib Hasnain, Simon I Hay, Fei He, Mohammad Heidari, Golnaz Heidari, Narmin Mohammedsaeed Helal, Brenda Yuliana Herrera-Serna, Kamran Heydaryan, Kamal Hezam, Nguyen Quoc Hoan, Matthew Hobbs, Ramesh Holla, Syed Billal Hossain, Mehdi Hosseinzadeh, Sorin Hostiuc, Weijun Huang, Fernando N Hugo, Bing-Fang Hwang, Liliya Ibragimova, Khalid S Ibrahim, Jibran Ikram, Olayinka Stephen Ilesanmi, Irena M Ilic, Milena D Ilic, Danish Iqbal, Bilal Irfan, Md Rabiul Islam, Gaetano Isola, Hiroshi Ito, Chidozie Declan Iwu, Haitham Jahrami, Ayushi Jain, Ammar Abdulrahman Jairoun, Mihajlo Jakovljevic, Sahbanathul Missiriya Jalal, Reza Jalilzadeh Yengejeh, Rajiv Janardhanan, Sathish Kumar Jayapal, Ruwan Duminda Jayasinghe, Wonjeong Jeong, Nathan T Jibat, Nitin Joseph, Krupal Joshi, Farahnaz Joukar, Easter Joury, Jacek Jerzy Jozwiak, Hema Shree K, Thenmozhi K, Zubair Kabir, Vidya Kadashetti, Adel Kadri, Pradnya Vishal Kakodkar, Eswar Kandaswamy, Kehinde Kazeem Kanmodi, Kamala Kannan, Rami S Kantar, Neila Karagezova, Indira Karibayeva, Mohmed Isaqali Karobari, Tomasz M Karpiński, Gebrehiwot G Kassa, Birhanu Kassie Abeje, Joonas H Kauppila, Stella Adejoke Kayode, Awoke Amare Kebede, Yousef Saleh Khader, Himanshu Khajuria, Raees Khan, Zahid Khan, Hafiz Nadeem Khan, Muhammad Umer Khan, Muhammad Khan, Sameer Uttamaro Khasbage, Khalid A Kheirallah, Feriha Fatima Khidri, Yihyun Kim, Min Seo Kim, Sung Min Kim, Ruth W Kimokoti, Sanjay Kini B, Adnan Kisa, Tobias Kleinjung, Shivakumar KM, Prakash Babu Kodali, Farzad Kompani, Archana Koul, Kewal Krishan, Bindu Krishnan, Nitya Krishnasamy, Omar Kujan, Anit Kujur, Mukhtar Kulimbet, Emmanuel Kumah, Manoj Kumar, Jogender Kumar, Pradeep Kumar, Jeevan Kumar, Pawan Kumar, Sumeet Kumar, Ashok Kumar, Kamal Kumar, Neeraj Kumar, Dewesh Kumar, Sandeep Kumar, Jibin Kunjavara, Om P Kurmi, Pramod Kumar Kushawaha, Dian Kusuma, Timo Lajunen, Dharmesh Kumar Lal, Ratilal Lalloo, Tamás Lantos, Giusy Rita Maria La Rosa, Savita Lasrado, Seung Won Lee, Kakithakara Vajravelu Leela, Elvynna Leong, Mengyi LI, Zhengrui Li, Ming-Chieh Li, Ambreen Liaqat, Liu Liu, Erand Llanaj, Madeeha Shahzad Lodhi, Mariia Lopatkina, Anna Lövgren, Shanjie Luan, Alessandra Lugo, Ellina Lytvyak, Hawraz Ibrahim M. Amin, Siyao Ma, Kevin Sheng-Kai Ma, Monika Machoy-Rakoczy, Máté Mackei, Inamul Hasan Madar, Ahmed A Madfa, Mohammed Magdy Abd El Razek, Mari Kannan Maharajan, Nozad Hussein Mahmood, Trisha Mallick, Iqra Manzoor, Tahir Maqbool, Vijaya Marakala, Priyadharshini Marckasagayam, Stefano Masi, Manu Raj Mathur, Medha Mathur, Khurshid A Mattoo, Jacek Matys, Jitendra Kumar Meena, Riffat Mehboob, Vini Mehta, Dalia E Meisha, Satish Melwani, Ritesh G Menezes, Tomislav Mestrovic, Andrea Michelerio, Giuseppe Minervini, Tridip Mitra, Kanisha Mittal, Hashem Mohamadian, Nouh Saad Mohamed, Fatima Awad Elkarim Elfaki Mohamed, Yahaya Mohammed, Yugal Kishore Mohanta, Ali H Mokdad, Himel Mondal, Paula Moraga, Rafael Silveira Moreira, Mahdis Morovvati, Rohith Motappa, Amin Mousavi Khaneghah, Ahmed Msherghi, Rabia Mubarak, Sumaira Mubarik, Christopher J L Murray, Satta Muthu Murugan,

David Musoke, Sherzad Ibrahim Mustafa, Saravanan Muthupandian, Kiran P. P Nagdeo, Ganesh R Naik, Lekshmi M Nair, Prasad Nalabothu, Rizwan Naqvi, Shumaila Nargus, Gustavo G Nascimento, Mohammad Zakaria Nassani, Zuhair S Natto, Zakira Naureen, Kinjal Nayak, Biswa Prakash Nayak, Javad Nazari, Sudan Prasad Neupane, Sumaiya Nezam, Long Hoang Nguyen, Tham Thi Nguyen, Cuong Tat Nguyen, Duy Cao Nguyen, Vikram Niranjan, Lawrence Achilles Nnyanzi, Mohammed Noushad, Nazik Nurelhuda, Bogdan Oancea, Musa Kolapo Oladejo, Yinka Doris Oluwafemi, Esteban Ortiz-Prado, Mahesh P A, Alicia Padron-Monedero, Jagadish Rao Padubidri, Sujogya Kumar Panda, Keerthi Panneer Selvam, Georgios D Panos, Shahina Pardhan, Romil R Parikh, Sun Jae Park, Pragnesh Parmar, Sameena Parveen, Eleonora Pascucci, Mitesh Patel, Shivani Patel, Mehali Lalitbhai Patel, Jay Patel, Vipin Patidar, Shankargouda Patil, Shrikant Pawar, Olumuyiwa James Peter, Pawel Piotr Pietkiewicz, Thantrira Porntavetus, Amit Porwal, Priyanka Porwal, Sergio I Prada, Harsh Priya, Luís Proença, Pooja Puri, Bharathi M Purohit, Nameer Hashim Qasim, Asma Saleem Qazi, Xiang Qi, Cheng Quan, Nuzul Qur'aniati, Venkatraman Radhakrishnan, Raghu Anekal Radhakrishnan, Fakher Rahim, Mosiur Rahman, Hakim Rahmoune, Amina Suleiman Rajah, Rafał Rakoczy, Venkitachalam Ramanarayanan, Shakthi Kumaran Ramasamy, Ramasubburayan Ramasamy, Lokesh Rana, Shailendra Singh Rana, Kumuda Rao, Sowmya J Rao, Mithun Rao, Prateek Rastogi, Shree Rath, Devarajan Rathish, Tapash Ranjan Rautray, Sujith Sri Surya Ravi, Salman Rawaf, Muhammad Liaquat Raza, Elrashdy M Redwan, Abdul Rehman, Bhagwan Narayan Rekadwad, Nazila Rezaei, Abanoub Riad, Maria Jesus Rios-Blancas, Hermano Alexandre Lima Rocha, Chanapong Rojanaworarit, Mario Romandini, Rohini Ruhil, Khaled Saad, Korosh Saber, Fatemeh Sadeghi-Ghyassi, Mohammad Reza Saeb, Umar Saeed, Amirhossein Sahebkar, Fatemeh Saheb Sharif-Askari, Vaibhav Sahni, Durgesh Prasad Sahoo, Ambika Sahoo, Alaka Sahoo, Ankita Saikia, Joseph W Sakshaug, Luciane B Salaroli, Samreen Saleem, Firdaus Samad, Fasineh Shekuba Samura, Abdallah M Samy, Sathish Sankar, Andari Sarasati, Shilpa Sarawagi, Sachin C Sarode, Gargi Sachin Sarode, Michele Sassano, Aanchal Satija, Maheswar Satpathy, Jennifer Saulam, Amar Saurabh, Benedikt Michael Schaarschmidt, Falk Schwendicke, Amin Sedigh, Siddharthan Selvaraj, Subramanian Senthilkumaran, Mostafa Shaban, Arsalan Shahri, Hamid R Shahsavari, Muhammad Shahzad, Masood Ali Shaikh, Muhammad Aaqib Shamim, Alfiya Shamsutdinova, Mohammed Shannawaz, Nadim Sharif, Amin Sharifan, Kamlesh Sharma, Bereket Beyene Shashamo, Mahabalesh Shetty, Premalatha K Shetty, Nzooma Munkwangu Shimaponda-Mataa, Shirindokht Shirazi, Km Shivangi, Zahra Shokati Eshkiki, Deborah Oluwaseun Shomuyiwa, Sinegugu Nosipho Shongwe, Seyed Afshin Shorofi, Sunil Shrestha, Suleiman Adeiza Shuaibu, Gustavo Correia Basto Da Silva, Luís Manuel Lopes Rodrigues Silva, Jasvinder A Singh, Harpreet Singh, Surjit Singh, Samer Singh, Prince Kumar Singh, Siddharth Singh, Ragini D Singh, Amit Singh, Abhinav Singh, Richa Singhania, Gisha Sivan, Gowri Sivaramakrishnan, Chandan S N, Farrukh Sobia, Ranjan Solanki, Monica Charlotte Solomon, Saraswathy Sreeram, Kannan Sridharan, Quirin D Strotzer, Mahwish Suhaib, Marjia Sultana, Jing Sun, Seyyed Mohammad Tabatabaei, Biniyam Beyene Tabor, Santosh Kumar Tadakamadla, Jyothi Tadakamadla, Amir Tahavvori, Jabeen Taiba, Iman M Talaat, Mircea Tampa, Benjamin Kye Jyn Tan, Shynar Tanabayeva, Abdelghani Tbakhi, Reem Temsah, Abdulkarim Temsah, Dufera Rikitu Terefa, Tigist Derebe Tesfahun, Azimeraw Arega Tesfu, Ismaeel Tharwat, Manuel Sebastian Thomas, Amir Tiyuri, Madi Tleshev, Razie Toghroli, Arole Darwin Touko, Marcos Roberto Tovani-Palone, Thang Huu Tran, Indang Trihandini, Mike Tuffour Amirikah, Biruk Shalmeno Tusa, Saeed Ullah, Srikanth Umakanthan, Bhaskaran Unnikrishnan, Amedin Aliyi Usso, Olivier Uwishema, Hande Uzunçıbuk, Orsolya Varga, Siddhartha Alluri Varma, Vishnu Priya Veeraraghavan, Prabhakar Veginadu, U Venkatesh, Nguyen-Kieu Viet-Nhi, Vignesh Vikram, Victor E Villalobos-Daniel, Ave Voit, Simona Ruxandra Volovat, Rashim Wadhwa, Liang Wang, Wanzhou Wang, Zhikang Wang, Tati Suryati Warouw, Chenxuan Wei, Desalegn Desalegn Weldebrhan, Nuwan Darshana Wickramasinghe, Andrew Awuah Wireko, Dawit Habte Woldeyes, Shulan Wu, Patlolla Sriram Yadav, Saba Yahoo, Natsuhiko Yamada, Kivanc Yangi, Thomas Kidanemariam Yewodiaw, Siyan Yi, Dong Keon Yon, Monal Yuwanati, Vesna Zadnik, Mubashir Zafar, Muhammad Sohail Zafar, Iman Zare, Mohammed G M Zeariya, Zhi-

Jiang Zhang, Jianhui Zhao, Lei Zheng, Hafsa Zia, Zahraa Zibara, Magdalena Zielińska, Shaher H Zyoud, Kanyin Liane Ong**.

*First author
**Senior author

Affiliations

Institute of Dentistry (Prof E Bernabe PhD, Prof W Marcenes PhD), Queen Mary University of London, London, UK; Care in Long Term Conditions Research Division (J Arias De La Torre PhD), King's College London, London, UK; Medicine Department (A . MD), Rawalpindi Medical University, Rawalpindi, Pakistan; Department of Radiation Oncology (H S Ababneh MD), Massachusetts General Hospital, Boston, MA, USA (M Kim MD); University of Gondar Comprehensive Specialized Hospital (B J Abate MPH), Institute of Public Health (T K Yewodiaw MPH), University of Gondar, Gondar, Ethiopia; Advanced Diagnostic and Interventional Radiology Research Center (ADIR) (H Abbastabar PhD), Dental Research Center (M Banakar PhD), Non-Communicable Diseases Research Center (M Bastan MD, N Rezaei MD), Dentistry Research Institute (F Farshad DDS), Children's Medical Center (Prof F Kompani MD), School of Medicine (M Morovvati MD), Department of Medical Education (A Sedigh PhD), Tehran University of Medical Sciences, Tehran, Iran; Department of Medical Laboratory Science (Prof W M Abdel-Rahman PhD), Department of Nursing (Prof S Abuhammad PhD), Department of Restorative Dentistry (A B Acharya PhD), College of Dental Medicine (M A Alsaegh PhD), Research Institute for Medical and Health Sciences (M A Alsaegh PhD), Department of Pharmacy Practice and Pharmacotherapeutics (Prof K H Alzoubi PhD), Department of Dental Medicine (Prof M Awad PhD), Sharjah Institute of Medical Sciences (F Saheb Sharif-Askari PhD), Clinical Sciences Department (Prof I M Talaat PhD), University of Sharjah, Sharjah, United Arab Emirates; Department of Tropical Medicine and Infectious Diseases (S Abd-Elsalam PhD), Tanta University, Egypt, Tanta, Egypt; Faculty of Medicine (H Abdou MD), Capital (Helwan) University, Cairo, Egypt; Department of Medicine (Prof M Abdoun PhD), Department of Health (Prof M Abdoun PhD), University of Setif Algeria, Sétif, Algeria; National Institute of Epidemiology (R Abdulkader PhD), Indian Council of Medical Research, Chennai, India; Department of Biomedical Sciences (S Abidi PhD), Nazarbayev University School of Medicine, Astana, Kazakhstan; Department of Family and Community Health (R G Aboagye MPH), Department of Nutrition and Dietetics (P D Agordoh MPhil), Department of Population and Behavioural Sciences (H Amu PhD), University of Health and Allied Sciences, Ho, Ghana; School of Population Health (R G Aboagye MPH), University of New South Wales, Sydney, NSW, Australia; Department of Pediatric Dentistry (Prof L Abreu PhD), Department of Community and Preventive Dentistry (Prof M N G De Abreu PhD), Faculty of Dentistry (Prof G C B D Silva PhD), Federal University of Minas Gerais, Belo Horizonte, Brazil; Department of Maternal and Child Health Nursing (Prof S Abuhammad PhD), Department of Pathology and Microbiology (Prof M Alqudah MD), Department of Public Health (Prof Y S Khader PhD, Prof K A Kheirallah PhD), Jordan University of Science and Technology, Irbid, Jordan; Department of Oral Pathology and Microbiology (S Acharya PhD), JSS Academy of Higher Education and Research, Mysuru, India; Department of Human Anatomy (L H Adamu PhD), Federal University Dutse, Dutse, Nigeria; Department of Anatomy (L H Adamu PhD), Bayero University Kano, Kano, Nigeria; Department of Microbiology (T A Adebisi BSc), Ladoke Akintola University, Osogbo, Nigeria; Department of NMC Healthcare (T A Adebisi BSc), Independent Consultant, Sharjah, United Arab Emirates; Department of Pediatrics (B O Adebiyi PhD), University of Calgary, Calgary, AB, Canada; School of Public Health (B O Adebiyi PhD), University of the Western Cape, Bellville, South Africa; Department of Immunology (K A Adedokun MSc), Roswell Park Comprehensive Cancer Center, Buffalo, NY, USA; Graduate Program Division (K A Adedokun MSc), University at Buffalo, Buffalo, NY, USA; Technical Services Directorate (A A

Afolabi MPH), MSI Nigeria Reproductive Choices, Abuja, Nigeria; Department of Life Sciences (M S Afzal PhD), University of Management and Technology, Lahore, Pakistan; Operations Department (S W Agordoh MPH), World Vision, Kete Krachi, Ghana; Sujan Surgical Cancer Hospital & Amravati Cancer Foundation, Amravati, India (T Agrawal MBBS); Division of Medical Research (P Agrawal PhD), SRM Institute of Science and Technology, Kattankulathur, India; School of Public Health (T Ahmad PhD), Stomatology Hospital (Z Wang MS), Department of Epidemiology and Biostatistics (J Zhao MD), Zhejiang University, Hangzhou, China; Department of Community Health Sciences (T Ahmad PhD), Sohail University, Karachi, Pakistan; Department of Health and Biological Sciences (S Ahmad PhD), Abasyn University, Peshawar, Pakistan; Department of Natural Sciences (S Ahmad PhD), Lebanese American University, Beirut, Lebanon; College of Medicine (A Ahmad PhD), Shaqra University, Shaqra, Saudi Arabia; Department of General Surgery (S Ahmad MD), School of Surgery Wales, Swansea, UK; King Khaled Eye Specialist Hospital and Research Center, Riyadh, Saudi Arabia (Prof K Ahmad PhD); Department of Epidemiology and Biostatistics (Prof A Ahmadi PhD), Community-Oriented Nursing Midwifery Research Center (M Heidari PhD), Shahrekord University of Medical Sciences, Shahrekord, Iran; Department of Epidemiology (Prof A Ahmadi PhD), Department of Oral and Maxillofacial Surgery (M Alam MSc, M Golkar MSc), Department of Allied Medical Sciences (B Alizadeh Savareh PhD), Obstetrics and Gynecology Department (E Ghotbi MD), Oral and Maxillofacial Surgery Department (H Haeriboroojeni DMD), Shahid Beheshti University of Medical Sciences, Tehran, Iran; Department of Assistance Medical Sciences (N Ahmed PhD), Nursing Faculty (K A Alnawafleh PhD), Applied College (A A Baig PhD), Department of Medical Laboratory Technology FAMS (Prof S Muthupandian PhD), Prince Fahad Bin Sultan Chair for Biomedical Research (Prof S Muthupandian PhD), University of Tabuk, Tabuk, Saudi Arabia (S Ali PhD); Department of Computer Science (S Ahmed PhD), Department of Preventive Dental Sciences (Prof M Al-Ak'hali PhD, A K Bhati MDS), Department of Public Health (S Dohare MD, F Sobia PhD), Department of Maxillofacial Surgery and Diagnostic Sciences (E Halboub PhD, S Parveen PhD), Department of Prosthetic Dental Sciences (K A Mattoo MD, A Porwal PhD), Clinical Nutrition Department, College of Nursing and Health Sciences (F A E E Mohamed PhD), Jazan University, Jazan, Saudi Arabia; Department of Pharmacy Practice (A Ahmed PhD), Riphah Institute of Pharmaceutical Sciences, Islamabad, Pakistan; Division of Infectious Diseases and Global Public Health (idgph) (A Ahmed PhD), University of California San Diego, San Diego, CA, USA; Institute of Endemic Diseases (A Ahmed PhD), Department of Oral Rehabilitation (N T Hashim PhD), University of Khartoum, Khartoum, Sudan; Pan-African One Health Institute (PAOHI), Kigali, Rwanda (A Ahmed PhD); Department of Social Sciences (K W Ajele PhD), North-West University, South Africa, Mafikeng, South Africa; Rural Health Research Institute (K H Akalanka PhD, A E Anyasodor PhD), Charles Sturt University, Orange, NSW, Australia; Faculty of Dentistry, Oral Diagnostic Sciences (S Akeel PhD), Faculty of Dentistry- Endodontic Department (Prof R A Alamoudi MS), Pediatric Dentistry Department (K K Baghlaf PhD, G Y Bhadila PhD, N M Helal MPH), Department of Pediatric Dentistry, Faculty of Dentistry (D Bahdila DMSc), Department of Oral Diagnostic Sciences (N Binmadi PhD), Department of Biological Sciences (Prof K R Hakeem PhD), Oral & Maxillofacial Rehabilitation Department (M Hamed DSc), Dental Public Health Department, Faculty of Dentistry (D E Meisha DScD), Department of Dental Public Health (Prof Z S Natto DrPH), King Abdulaziz University, Jeddah, Saudi Arabia; Medecins Sans Frontieres - Holland (Emergency Desk) (D M Akintunde MPH), Doctors Without Borders, Omdurman, Sudan; Department of Community Medicine (D M Akintunde MPH), Ahmadu Bello University, Zaria, Nigeria; Department of Infection Prevention & Control (H Akram MD), Baylor Scott & White Health, Frisco, TX, USA; VentureBlick, (H Akram MD); Department of Public Health (C J Akunna DMD), The Intercountry Centre for Oral Health (ICOH) for Africa, Jos, Nigeria; Department of Public Health (C J Akunna DMD), Federal Ministry of Health, Garki, Nigeria; Department of Oral and Maxillofacial Surgery (T O Aladelusi FWACS), Department of Epidemiology and Medical Statistics (A F Fagbamigbe PhD), University of

Ibadan, Ibadan, Nigeria; Department of Oral and Maxillofacial Surgery (T O Aladelusi FWACS), Department of Community Medicine (O S Ilesanmi PhD), University College Hospital, Ibadan, Ibadan, Nigeria; Department of Rehabilitation (Y Al-Ajlouni MD), Montefiore Medical Center, Bronx, NY, USA; Department of Epidemiology (Y Al-Ajlouni MD), Columbia University, New York, NY, USA; Preventive Dentistry Department (Prof M K Alam PhD), Department of Maternal and Child Health Nursing (N B Elsharkawy PhD), Jouf University, Sakaka, Saudi Arabia (M Shaban PhD); Clinical Sciences Department (A H Al Ani FRCS), College of Dentistry (D Gopinath PhD), Department of Pathological Sciences (A Rehman PhD), Department of Clinical Sciences (Prof M S Zafar PhD), Ajman University, Ajman, United Arab Emirates; Department of Communicable Diseases (S Al Awaidy MSc), Ministry of Health, Muscat, Oman; Middle East, Eurasia, and Africa Influenza Stakeholders Network, Muscat, Oman (S Al Awaidy MSc); Department of Community and Mental Health (Prof M Albashtawy PhD), Al al-Bayt University, Mafraq, Jordan; General Directorate of Research and Studies (M S Aldossary MClinDent), Ministry of Health, Riyadh, Saudi Arabia; Department of Bacteriology, Immunology, and Mycology (Prof A M Algammal PhD), Suez Canal University, Ismailia, Egypt; Clinical Technology Department (S M Alghamdi PhD), Faculty of Applied Medical Sciences, Mecca, Saudi Arabia; Faculty of Dentistry (M A Alghauli PhD), Department of Prosthodontics and Implant Dentistry (Prof A Alqutaibi PhD), Ibb University, Ibb, Yemen; Department of Adult Health (A Alhassan MS), University of Cape Coast, Cape Coast, Ghana; Faculty of Dentistry (A Alhumaidi DDS), Ibn Al-Nafis University for Medical Sciences, Sana'A, Yemen; Department of Oral and Maxillofacial Surgery (A Alhumaidi DDS), Xi'an Jiaotong University, Xi'An, China; Department of Statistics and Informative (Prof T H Ali PhD), Department of Electrical Engineering (Prof I Hamarash PhD), Salahaddin University-Erbil, Erbil, Iraq; College of Dental Medicine (Prof K Ali PhD, S A Al-Maweri PhD), Qatar University, Doha, Qatar (S Hammoudeh DrPH); Center for Biotechnology and Microbiology (S Ali PhD), University of Swat, Swat, Pakistan; The School of Medicine (M Al-Iede MD), School of Pharmacy (Prof L Dahabiyeh PhD), The University of Jordan, Amman, Jordan; Biomedical Physics Group (M Alipour BSc), University of Hamburg, Hamburg, Germany; Independent Consultant, Amman, Jordan (S Al-Marwani MSc); Department of Oral and Maxillofacial Surgery (Prof E A Al-Moraissi PhD), Thamar University, Thamar, Yemen; Department of Clinical Nursing (M M Alnaeem PhD), Al-Zaytoonah University of Jordan, Amman, Jordan; SRM School of Public Health, Chennai, India (M Alocious Sukumar PhD); Department of Surgery (N Alonso MD), University of São Paulo, São Paulo, Brazil; Department of CTAG (Prof M Alqudah MD), Department of Cell Therapy and Applied Genomics (A Tbakhi MD), King Hussein Cancer Center, Amman, Jordan; American University of the Middle East, Egaila, Kuwait (M R Alqudimat PhD); Department of Prosthodontics and Implant Dentistry (Prof A Alqutaibi PhD), Taibah University, Madinah, Saudi Arabia; College of Medicine (M H Alsaadi MD), Jaber Ibn Hayyan University for Medical and Pharmaceutical Sciences, Kufa, Iraq; Department of Dentistry (A Alsalhani MSc), Vision Colleges, Riyadh, Saudi Arabia; Medical College (A Alsalhani MSc), Hama University, Hama, Syria; Department of Pharmacology (A S Al-Shami MS), Sana'a University, Sanaa, Yemen; Department of Basic Medical Sciences (A S Al-Shami MS), Universiti Malaysia Sarawak, Kota Samarahan, Malaysia; Department of Basic Sciences (Z Altaany PhD), Faculty of Medicine, Irbid, Jordan; Health Science Division (Z Altaany PhD), Higher Collage of Technology, Abu Dhabi, United Arab Emirates; Institute of Molecular Biology and Biotechnology (IMBB) (A Altaf PhD, S J Awan PhD, M Khan PhD, M S Lodhi PhD), Department of Food & Health (H Bader Ul Ain PhD), University Institute of Food Science and Technology (S Bashir PhD), University Institute of Public Health (A Gohar PhD, S Nargus PhD), University Institute of Radiological Sciences and Medical Imaging Technology (I Manzoor MPhil), Institute of Molecular Biology and Biotechnology (T Maqbool PhD), The University of Lahore, Lahore, Pakistan; Faculty of Health Sciences (A Altaf PhD), Equator University of Science and Technology, Uganda, Masaka, Uganda; Department of Pediatrics (Prof H Aly MD), Pediatric Institute (M A Farghaly PhD), Department of Cardiovascular Medicine (J Ikram MD), Cleveland Clinic, Cleveland,

OH, USA; Department of Food Science and Human Nutrition, College of Agriculture and Food (N A Alzunaidy PhD), Qassim University, Buraydah, Saudi Arabia; Evaluation Unit (U A Amaechi BDS), Global Alliance for Vaccines and Immunisations, Geneva, Switzerland; John D. Bower School of Population Health Science (M I Amedari MPH), University of Mississippi Medical Center, Jackson, MS, USA; Preventive and Community Dentistry (M I Amedari MPH), Obafemi Awolowo University, Ile-Ife, Nigeria; Department of Preventive Dentistry (P O Ameh DDS), University of Jos, Jos, Nigeria; Department of Preventive Dentistry (P O Ameh DDS), Jos University Teaching Hospital, Jos, Nigeria; Department of Physiotherapy (J Amin MSc), Department of Biology (Prof R Badraoui PhD), Department of Restorative Dentistry (Prof H A E N Bastawy PhD), College of Dentistry (Prof A A Madfa PhD), Department of Family and Community Medicine (M Zafar PhD), Department of Public Health (M G M Zeariya PhD), University of Hail, Hail, Saudi Arabia; Clinical Sciences - Orthopaedic (J Amin MSc), University of Science Malaysia, Kota Baharu, Malaysia; Department of Health and Management Sciences (S Amini PhD), Operating Room Department (A Sedigh PhD), Khomein University of Medical Sciences, Khomein, Iran; Department of Oral Medicine, School of Dentistry (M Amirzade-Iranaq DDS), Department of Medical Physics (K Saber PhD), Isfahan University of Medical Sciences, Isfahan, Iran; Department of Pharmacology (A Anil MD), Department of Medicine (S Rath MBBS), All India Institute of Medical Sciences, Bhubaneswar, India; Division of Medical Research (A Anil Kumar PhD, T Mitra MSc), Department of Microbiology (K V Leela MD, S Ravi DSc), SRM School of Public Health, Faculty of Medicine and Health Sciences (K Panneer Selvam PhD), Sri Ramaswamy Memorial Institute of Science and Technology, Chengalpattu, India; Department of Public Health (K Annadurai PhD), Department of Biomedical Sciences (J Kumar PhD), The Apollo University, Chittoor, India; Regenerative Medicine, Organ Procurement and Transplantation Multi-Disciplinary Center (S Anvari MD), Department of Social Medicine and Epidemiology (A Feizkhah MD), Gastrointestinal and Liver Diseases Research Center (F Joukar PhD), Caspian Digestive Disease Research Center (F Joukar PhD), Guilan University of Medical Sciences, Rasht, Iran; Health Management and Economics Research Center (J Arabloo PhD), School of Medicine (M Bastan MD), Iran University of Medical Sciences, Tehran, Iran; Al Ain University (Prof M Arafat PhD), Al Ain University, Abu Dhabi, United Arab Emirates; Department of Head and Neck Oncology (Prof G Arakeri PhD), HCG Cancer Hospital, Bengaluru, India, Bengaluru, India; Department of Applied Mathematics (A Y Aravkin PhD), Institute for Health Metrics and Evaluation (A Y Aravkin PhD, S Ghoba MS, Prof S I Hay DSc, T Mestrovic PhD, Prof A H Mokdad PhD, Prof C J L Murray DPhil, K L Ong PhD), Department of Health Metrics Sciences, School of Medicine (A Y Aravkin PhD, Prof S I Hay DSc, Prof A H Mokdad PhD, Prof C J L Murray DPhil), School of Health Systems and Public Health (C Iwu MPH), Department of Epidemiology (H Zia BDS), University of Washington, Seattle, WA, USA; College of Art and Science (D Areda PhD), Ottawa University, Surprise, AZ, USA; Department of Grupo 15 (J Arias De La Torre PhD), CIBER Epidemiology and Public Health (CIBERESP), Madrid, Spain; Department of Medical Laboratory Sciences (M Ashraf PhD), Riyadh ELM University, Riyadh, Saudi Arabia; Department of Oral Public Health (J Ashraf PhD), Public Health Department (J Ashraf PhD), Department of Human Nutrition & Dietetics (S Saleem PhD), Health Services Academy, Islamabad, Pakistan; Department of Biostatistics (T Ashraf PhD), Sakarya University, Sakarya, Türkiye; Department of Nutrition and Dietetics (E Asil PhD), Ankara University, Ankara, Türkiye; CMH Lahore Medical College & Institute of Dentistry (Prof S Atif PhD), National University of Medical Sciences (NUMS), Lahore, Pakistan; School of Statistics and Actuarial Science (S B Atoyebi MSc), Wits Advanced Drug Delivery Platform Research Unit (Prof P Kumar PhD), Wits Infectious Diseases and Oncology Research Institute (Prof P Kumar PhD), University of the Witwatersrand, Johannesburg, South Africa; Department of Oral Biological Science (N Aung MSc), University of Dental Medicine, Mandalay, Myanmar; Dental Unit (N Aung MSc), U Hla Tun's Hospice Cancer Foundation, Mandalay, Myanmar; Department of Medical Laboratory Technology (U Awan PhD), The University of Haripur, Haripur, Pakistan; Department of Biostatistics (A

Awasthi PhD), CSIR-Central Drug Research Institute, Lucknow, India; Department of Biostatistics (A Awasthi PhD), Academy of Scientific and Innovative Research (AcSIR), Ghaziabad, India; Department of Public Health Dentistry (A Ayoob MDS, V Ramanarayanan MDS), Amrita Institute of Medical Sciences, Kochi, India; International Research Collaborative- Health and Equity (S Azimi PhD), Dental School (O Kujan PhD), Paediatric Dentistry, UWA Dental School (A Saikia PhD), Marshall Centre for Interventions in Infectious Disease (Prof L Wang PhD), The University of Western Australia, Perth, WA, Australia; Geriatric Unit (D Azzolino PhD), Fondazione IRCCS Ca' Granda Ospedale Maggiore Policlinico, Milan, Italy; Directorate of Quality Assurance (M Badar PhD), Gomal University, Dera Ismail Khan, Pakistan; Department of Forensic Science (A D Badiye PhD), Government Institute of Forensic Science Nagpur, Nagpur, India; Rashtrasant Tukadoji Maharaj Nagpur University, Nagpur, India (A D Badiye PhD); Faculty of Medicine of Tunis (Prof R Badraoui PhD), University Tunis El Manar, Tunis, Tunisia; Department of Pharmacy (M Baig PhD), Yonsei University, Incheon, South Korea; Department of Medical Informatics (M Bakhshali PhD, S Tabatabaei PhD), Orthodontics Department (M Ghorbani DDS), Biotechnology Research Center (Prof A Sahebkar PhD), Dental Materials Research Center (A Shahri DDS), Applied Biomedical Research Center (S Tabatabaei PhD), Mashhad University of Medical Sciences, Mashhad, Iran; Department of Pathological Physiology (Prof M Balabekova DSc), Science and Technology Park (Prof L Ibragimova PharmD), School of Dentistry (N Karagezova BSc), B. Atchabarov SRI FAM (M Lopatkina PhD), Science Department (A Shamsutdinova MD), Department of Medicine (S Tanabayeva PhD), Department of Prosthetic Dentistry (M Tleshev MSc), Kazakh National Medical University, Almaty, Kazakhstan; Saveetha Medical College and Hospital (R Balaji PhD, A Farooq Mohammed MPT, Prof M Karobari PhD), Department of Physiology (E Dilipan PhD), Department of Pharmacology (Prof D Ezhilarasan PhD), Saveetha Institute of Medical and Technical Sciences (D Francis MD, B Gunasekar PhD), Department of Oral Medicine and Periodontology (Prof R D Jayasinghe MS), Department of Pathology (H K PhD), Center for Marine and Aquatic Research (K Kannan PhD), Department of Oral Biology (N Krishnasamy PhD), Saveetha Institute of Basic Medical Sciences (sibms) (P Marckasagayam PhD), Saveetha Dental College and Hospitals (G Minervini PhD, M Tovani-Palone PhD, M Yuwanati MDS), Centre for Marine and Aquatic Research (R Ramasamy PhD), Center for Global Health Research (Prof A Sahebkar PhD), Centre of Molecular Medicine and Diagnostics (Prof V Veeraraghavan PhD), Saveetha University, Chennai, India; Department of Midwifery (W F Balcha MSc, A A Tesfu MSc), College of Medicine and Health Science (A N Belay MSc, T D Tesfahun MSc), Department of Nursing (A Y Berhie MSc), Department of Public Health Nutrition and Nursing Related (A A Kebede MSc), Department of Anatomy, Histology, and Embryology (D H Woldeyes MSc), Bahir Dar University, Bahir Dar, Ethiopia; Department of Restorative Dental Sciences (A A Balhaddad PhD), Department of Pathology (Prof R G Menezes MD), Imam Abdulrahman Bin Faisal University, Dammam, Saudi Arabia; National Drug Dependence Treatment Centre (Prof Y Balhara MD), Centre for Community Medicine (P Halder MD), Department of Preventive Oncology (J K Meena MD), Centre for Dental Education and Research (H Priya MDS, B M Purohit MDS), Department of Radiation Oncology (V Sahni MDS), Department of Laboratory Medicine (A Singh PhD), All India Institute of Medical Sciences, New Delhi, India; Department of Hypertension (Prof M Banach PhD), Medical University of Lodz, Lodz, Poland; Polish Mothers' Memorial Hospital Research Institute, Lodz, Poland (Prof M Banach PhD); Health Policy Research Center (M Banakar PhD), Department of Health Information Management (A Bashiri PhD), Shiraz University of Medical Sciences, Shiraz, Iran; Irbid National University (S Bani Hani PhD), Irbid National University, Jordan, Jordan; Department of Noncommunicable Diseases (P C Banik MPhil), Bangladesh University of Health Sciences, Dhaka, Bangladesh; Center for Higher Studies and Research (P C Banik MPhil), Bangladesh University of Professionals, Dhaka, Bangladesh; Biochemistry Department (F Bano PhD), International Center for Chemical and Biological Sciences (S Ullah MSc), University of Karachi, Karachi, Pakistan; Department of

Pharmaceutics (M A Barkat PhD), University of Hafr Al Batin, Hafr Al Batin, Saudi Arabia; Department of Medicine Preventive and Public Health (R Barrios-Rodríguez PhD), University of Granada, Granada, Spain; Department of Public and Environmental Health (A Barrow MPH), University of The Gambia, Banjul, The Gambia; Department of Epidemiology (A Barrow MPH, D Braithwaite PhD), University of Florida, Gainesville, FL, USA; Endodontic Department (Prof H A E N Bastawy PhD), Department of Zoology and Entomology (M G M Zeariya PhD), Al-Azhar University, Cairo, Egypt; Department of Medical Education (K Batra PhD), University of Nevada Las Vegas, Las Vegas, NV, USA; Department of Public Health and Health Policy (O Batsaikhan PhD), Hiroshima University, Hiroshima, Japan; Scientific Research and Development (B Behnam MD), Avicenna Biotech Research, Clarksburg, MD, USA; Floret, Advanced Genomics & Bioinformatics Research Center, Lagos, Nigeria (B Behnam MD); Department of General Surgery, Plastic Surgery (E Belayneh MD), St. Paul's Hospital Millennium Medical College, Addis Ababa, Ethiopia; School of Public Health (N G Belete MPH), Haramaya University, Dire Dawa, Ethiopia; Department of Oral Pathology and Microbiology (U I Belgaumi MD), Department of Public Health Dentistry (Prof S KM PhD), Department of Periodontology (S A Varma MDS), Krishna Vishwa Vidyapeeth (Deemed to be University), Karad, India; Department of Oral Medicine and Diagnostic Science (Prof I Bello PhD), Department of Zoology (M K Oladejo MSc), King Saud University, Riyadh, Saudi Arabia; Department of Pathology (Prof I Bello PhD), Department of Psychology (Prof T Lajunen PhD), University of Helsinki, Helsinki, Finland; Independent Consultant, Barcelona, Spain (G Benhsen MSc); Department of Anatomy (P Bhadoria PhD, P Bhadoria PhD), All India Institute of Medical Sciences, Rishikesh, India; National Institute for Implementation Research on Non Communicable Diseases (Prof P Bhardwaj MD), Indian Council of Medical Research, Jodhpur, India; Global Health Neurology Lab (S Bhaskar MD), NSW Brain Clot Bank, Sydney, NSW, Australia; Division of Cerebrovascular Medicine and Neurology (S Bhaskar MD), National Cerebral and Cardiovascular Center, Suita, Japan; Department of General Medicine (A N Bhat MD), Department of Community Medicine (N Joseph MD, R Motappa MD), Department of Forensic Medicine and Toxicology (Prof J Padubidri MD, Prof P Rastogi MD), Department of Conservative Dentistry and Endodontics (M S Thomas MDS), Manipal Academy of Higher Education, Mangalore, India; Department of Global Public Health and Primary Care (Prof T Bjørge PhD), University of Bergen, Bergen, Norway; Cancer Registry of Norway, Oslo, Norway (Prof T Bjørge PhD); Department of Community and Family Medicine (Prof T Bodhare MD), All India Institute of Medical Sciences, Ramanathapuram, India; Department of Biomedical Sciences (S Bolla PhD), Nazarbayev University, Astana, Kazakhstan; Department of Medicine (Prof S Bouaoud DrPH), University Ferhat Abbas of Setif, Setif, Algeria; Department of Epidemiology and Preventive Medicine (Prof S Bouaoud DrPH), University Hospital Saadna Abdenour, Setif, Algeria; Laboratory of Human Pathology Biology (Prof A Bouyahya PhD), University Mohammed V, Rabat, Morocco; Department of Earth, Environment, and Equity (C Boxe PhD), Howard University, Washington, DC, USA; Department of Stomatology (Prof M Braccini Fagundes PhD), Department of Public Health (O Do Amaral Júnior PhD), Federal University of Santa Maria, Santa Maria, Brazil; Cancer Population Sciences Program (D Braithwaite PhD), University of Florida Health Cancer Center, Gainesville, FL, USA; Department of Epidemiology and Evidence-Based Medicine (Prof N I Briko DSc), I.M. Sechenov First Moscow State Medical University, Moscow, Russia; Nuffield Department of Medicine (M E Bryan BSc), Department of Psychiatry (E Joury PhD), University of Oxford, Oxford, UK; Cancer Program (M E Bryan BSc), Cardiovascular Disease Initiative (M Kim MD), Broad Institute of MIT and Harvard, Cambridge, MA, USA; Department of Oral & Maxillofacial - Head Neck Oncology (Prof L Bu PhD), Department of Epidemiology and Biostatistics (Prof S Mubarik PhD), School of Public Health (Prof Z Zhang PhD), Wuhan University, Wuhan, China; Department of Biological Sciences (S Bukhari MS, R Khan PhD, A S Qazi PhD), National University of Medical Sciences (NUMS), Rawalpindi, Pakistan; Faculty of Dentistry (Y Cao BSc), Yong Loo Lin School of Medicine (B Tan MBBS), Saw Swee Hock School of Public Health (Prof S Yi PhD),

Department of Dentistry (Prof L Zheng MD), National University of Singapore, Singapore, Singapore; Oncological Network, Prevention and Research Institute (G Gorini MD), Institute for Cancer Research, Prevention and Clinical Network, Florence, Italy (G Carreras PhD); Department of Medicine and Surgery (A Carugno PhD), University of Insubria, Varese, Italy; Psychological Research Center (cipsi) (Prof S Carvalho PhD), Life and Health Sciences Research Institute (ICVS) (Prof N Cruz-Martins PhD), University of Minho, Braga, Portugal; Research Unit on Applied Molecular Biosciences (ucibio) (Prof F Carvalho PhD), Institute for Research and Innovation in Health (i3s) (Prof N Cruz-Martins PhD), Department of FEUP - Faculdade de Engenharia Da Universidade Do Porto (Prof M P Ferraz PhD), University of Porto, Porto, Portugal; Posgrado de Medicina (postgraduate Program in Medicine) (P Chacón-Uscamaita DDS), Universidad Cientifica del Sur, Lima, Peru; State Disease Investigation Laboratory (S Chakraborty MVSc), Animal Resources Development Department, Agartala, India; Department of Public Health and Health Sciences (Prof V Chattu PhD), Tennessee State University (TSU), Nashville, TN, USA; Department of Community Medicine (Prof V Chattu PhD), Datta Meghe Institute of Medical Sciences, Sawangi, India; Department of Biology (A A Chaudhary PhD), College of Nursing (H Emad PhD), Imam Mohammad Ibn Saud Islamic University (IMSIU), Riyadh, Saudi Arabia; Department of Public Health (S Chaudhuri MD), Indian Institute of Public Health, Hyderabad, India; Department of Oral Medicine and Radiology (Prof A Chaurasia MD), Department of Oral Pathology and Microbiology (A Jain MDS), Hospital Administration (M Suhaib MHA), King George's Medical University, Lucknow, India; Department of Urology (J Chen MD), The Second Affiliated Hospital of Kunming Medical University, Kunming, China; School of Dentistry (M Chen DDS, C Wei PhD), University of Michigan, Ann Arbor, MI, USA (B Irfan MS); Department of Surgery (M Chowdhury MD), Department of Health Economics and Policy Management (N T Jibat MD), Health Policy and Management Department (S Wu MS), Bloomberg School of Public Health (N Yamada MD), Johns Hopkins University, Baltimore, MD, USA; The Interdisciplinary Research Group on Biomedicine and Health (D Chu PhD), Faculty of Applied Sciences (D Chu PhD), VNU International School (VNUIS), Hanoi, Viet Nam; JSS Dental College & Hospital (S C J MDS), Department of Respiratory Medicine (Prof M P A DNB), Jagadguru Sri Shivarathreeswara Academy of Health Education and Research, Mysore, India; Faculty of Physical Therapy (F Cuenca-Martínez PhD), Faculty of Physiotherapy (C Forner Álvarez MSc), University of Valencia, Valencia, Spain; Center for Immigrant and Refugee Health (O Dadras PhD), Public Health Institute, Los Angeles, CA, USA; Karnavati Scientific Research Centre (N Dagli MA), Karnavati Scientific Research Centre, Gandhinagar, India; Division of Delivery Research/implementation Research (N Dahiya MD), Indian Council of Medical Research, Delhi, India; Department of Dentistry (M Damineni DDS), Four States Dental Care, Joplin, MO, USA; Department of Public Health (S D Darcho MPH), Department of Epidemiology and Biostatistics (B S Tusa MPH), College of Health and Medical Sciences (A Usso MSc), Haramaya University, Harar, Ethiopia; Amedabad Dental College and Hospital (R Dave BDS), Gujarat University, Ahmedabad, India; Department of Health Policy & Health Services Research (Prof M N G De Abreu PhD), Boston University School of Public Health (K Mittal MPH), Department of Orthodontics (S Shirazi DDS), Henry S.goldman School of Dental Medicine (V Vikram MDS), Boston University, Boston, MA, USA; Department of Forensic Medicine (Prof E Dervišević PhD), University of Sarajevo, Sarajevo, Bosnia and Herzegovina; Department of Biotechnology (P Dhanaraj PhD), Karunya Institute of Technology and Sciences in INDIA, Coimbatore, India; Research and Development Cell (A S Dhane MBA), Department of Dental Research Cell (Prof V Mehta PhD), Department of Oral Pathology and Microbiology (Prof S C Sarode PhD, Prof G S Sarode PhD), Dr. D. Y. Patil Vidyapeeth, Pune (Deemed to be University), Pune, India; Unit of Dermatology (A Di Guardo MD), La Sapienza University, Rome, Italy; Department of IDI (A Di Guardo MD), IRCCS, Rome, Italy; Department of Computer Engineering (S Din PhD), Gachon University, Seongnam-Si, South Korea; Department of Physiology (S Din PhD), Faculty of Nursing (F Hasan PhD), Center of Excellence in Precision

Medicine and Digital Health (Prof M Khan PhD, R Naqvi PhD, Prof T Porntavetus PhD), Chulalongkorn University, Bangkok, Thailand; Laboratory of Drug Development and Technologies (C Domingues MSc), University of Coimbra, Coimbra, Portugal; Department of Nuclear Medicine (F Dondi MD), ASST Spedali Civili di Brescia and Università degli Studi di Brescia, Brescia, Italy; Shanghai Sixth People's Hospital Affiliated To Shanghai Jiao Tong University School of Medicine (L Dong MD), Department of Otorhinolaryngology Head and Neck Surgery (W Huang PhD), School of Medicine (Z Li PhD), Ninth People's Hospital (Prof M Romandini PhD), Shanghai Jiao Tong University, Shanghai, China; Independent Consultant, Bridgewater, NJ, USA (O P Doshi MS); Department of Maternal and Child Health (T O Dosumu MSc), Bowen University Iwo, Iwo, Nigeria; Department of Pathology (Prof J Du PhD), China Medical University, Shenyang, China; School of Stomatology (M Du PhD), Department of Periodontology (M Du PhD), School of Basic Medical Science (S Luan MD), Shandong University, Jinan, China; Indian Institute of Technology (BHU), Varanasi (Prof V K Dubey PhD), Indian Institute of Technology (BHU), Varanasi, India; Department of Hepatology (M Dubey PhD), Hospital Administration (M Suhaib MHA), Sanjay Gandhi Postgraduate Institute of Medical Sciences, Lucknow, India; Department of Pharmacology (S Dutta MD), Institute of National Importance (K Joshi MD), Department of Biochemistry (R D Singh PhD), All India Institute of Medical Sciences, Rajkot, India; Department of Conservative Dentistry With Endodontics (A M Dziedzic DSc), Medical University of Silesia, Katowice, Poland; Department of Dermatology, Venereology and Leprology (V Eddula MD), Apollo Institute of Medical Sciences and Research Chittoor, Chittoor, India; Department of Biochemistry (Prof A Eftekharimehrabad PhD), Ege University, Izmir, Türkiye; Nanobiomedicine International Research Center (Prof A Eftekharimehrabad PhD), Azerbaijan Medical University, Baku, Azerbaijan; Independent Consultant, Ahvaz, Iran (E Eini MSD); Faculty of Science and Health (M Ekholuenetale PhD), University of Portsmouth, Hampshire, UK; Department of Biomedical Sciences (S M Eko PhD), University of Buea, Buea, Cameroon; Department of Clinical Laboratory Sciences (A M Elasbali PhD), Jouf University, Al-Qurayyat, Saudi Arabia; Faculty of Sciences (A Elbouzidi PhD), University Mohammed the First, Oujda, Morocco; National Institute of Public Health Research (M Elhoumed PhD), Ministry of Health, Nouakchott, Mauritania; Pediatric Dentistry and Dental Public Health Department (Prof O A A Elmeligy PhD), Biomedical Informatics and Medical Statistics Department (Prof I El Sayed PhD), Department of Pediatric Dentistry and Dental Public Health (Prof M El Tantawi PhD), Department of Pathology (Prof I M Talaat PhD), Alexandria University, Alexandria, Egypt; College of Public Health and Health Informatics (Prof A A El-Metwally PhD), Department of Infection Prevention & Control (Prof M Raza PhD), King Saud bin Abdulaziz University for Health Sciences, Riyadh, Saudi Arabia; King Abdullah International Medical Research Center, Riyadh, Saudi Arabia (Prof A A El-Metwally PhD); MD Anderson Cancer Center Department of Plastic Surgery (R Elmorsi MD), University of Texas, Houston, TX, USA; Surgical Research Section (A EL Omri PhD), Artificial Intelligence Research and Innovation Hub (S Hammoudeh DrPH), Nursing & Midwifery Research Department (NMRD) (J Kunjavara PhD), Hamad Medical Corporation, Doha, Qatar; Faculty of Veterinary Medicine (Prof A S Eltahawy PhD), Damanhour University, Damanhur, Egypt; Alamein International University, Alamein City, Egypt (Prof M El Tantawi PhD); Department of Medicine (P Ethiraj PhD), University of Texas, San Antonio, TX, USA; Department of Biochemistry and Forensic Science (A U Ezirim PhD), Federal University of Technology Owerri, Owerri, Nigeria; Research Centre for Healthcare and Community (A F Fagbamigbe PhD), Coventry University, Coventry, UK; Tanner College of Dental Medicine (Prof A Fahim PhD), University of Pikeville, Pikeville, KY, USA; School of Dental Medicine (N Fakih BS), BAU International University Batumi, Batumi, Georgia; National Cancer Center, Head and Neck Surgical Oncology Program (E Farashi MD), National Institute of Health, Bethesda, MD, USA; College of Dentistry (M Fareed PhD), Ajman University Ajman UAE, Ajman, United Arab Emirates; Department of Chemistry (G Feng PhD), Duke University, Durham, NC, USA; i3S - Instituto de Investigação e Inovação em Saúde (Prof M P Ferraz PhD), University of

Porto, Porto, Portugal; Department of Social Sciences (Prof N Ferreira PhD), University of Nicosia, Nicosia, Cyprus; Department of Nursing (G Fetensa MSc), Department of Public Health (D R Terefa MSc), Wollega University, Nekemte, Ethiopia; Department of Biomedical, Metabolic and Neural Sciences (Prof T Filippini PhD), University of Modena and Reggio Emilia, Modena, Italy; School of Public Health (Prof T Filippini PhD), Health Initiative of the Americas (Prof V E Villalobos-Daniel PhD), University of California Berkeley, Berkeley, CA, USA; Institute of Public Health (F Fischer PhD), Charité Universitätsmedizin Berlin (Charité Medical University Berlin), Berlin, Germany; James Cancer Hospital at the Ohio State University (J L Fisher PhD), Ohio State University, Columbus, OH, USA; Department of Dermatology (Prof T Fukumoto PhD), Kyoto Prefectural University of Medicine, Kyoto, Japan; Health Services Management Training Centre (Prof P A Gaal PhD), Semmelweis University, Budapest, Hungary; Department of Applied Social Sciences (Prof P A Gaal PhD), Sapientia Hungarian University of Transylvania, Târgu-Mureş, Romania; Department of Dentistry (A R Gadbail PhD), Government Medical College, Nagpur, India; Department of Community Dentistry (P Gaewkhiew PhD), Mahidol University, Ratchathewi, Thailand; Department of Public Health (M Gajdács PhD), Department of Medical Physics and Informatics (T Lantos PhD), University of Szeged, Szeged, Hungary; Department of Medical Epidemiology (S Gallus PhD, A Lugo PhD), Mario Negri Institute for Pharmacological Research, Milan, Italy; Department of Health Policy and Administration (Prof F B Garcia PhD), University of the Philippines Manila, Manila, Philippines; Cochlear Center for Hearing and Public Health (T S Gebre MD), Johns Hopkins University, Philadelphia, PA, USA; Institute of Public Health (N S George MPH), Jagiellonian University Medical College, Krakow, Poland; School of Medicine and Population Health (N S George MPH), University of Sheffield, Sheffield, UK; School of Public Health (A Ghashghaee BSc), Qazvin University of Medical Sciences, Qazvin, Iran; Department of Dermatology (N Gholizadeh MD), Department of Medical-Surgical Nursing (S Shorofi PhD), Mazandaran University of Medical Sciences, Sari, Iran; International University of Health and Welfare (T G Gia Nguyen MD), International University of Health and Welfare, Narita, Japan; Warwick Medical School (Prof P S Gill DM), University of Warwick, Coventry, UK; Department of Radiation Oncology (Prof A K Goel MD), Department of Dentistry (S S Rana MDS), All India Institute of Medical Sciences, Bathinda, India; Department of Social and Preventive Dentistry (Prof M Goettems PhD), Federal University of Pelotas, Pelotas, Brazil; Department of Pediatrics (G Gökulu PhD), Mersin City Training and Research Hospital, Mersin, Türkiye; Department of Genetics (P Goleij MSc), Sana Institute of Higher Education, Sari, Iran; Universal Scientific Education and Research Network (usern) (P Goleij MSc), Kermanshah University of Medical Sciences, Kermanshah, Iran; Department of Oral Medicine & Radiology (S M Gondivkar PhD), Government Dental College & Hospital, Nagpur, India; Postgraduate Program in Epidemiology (Prof B N G Goulart DSc), Department of Preventive and Social Dentistry (F N Hugo PhD), Federal University of Rio Grande do Sul, Porto Alegre, Brazil; Department of Gastroenterology (C Gu MD), Cangnan Hospital of Traditional Chinese Medicine, Wenzhou, China; Medical Directorate (G Guarducci MD), Local Health Authority of Ferrara, Ferrara, Italy; Community and Family Medicine (S Gupta MD), Central Armed Police Forces Institute of Medical Sciences (AIIMS-CAPFIMS), Delhi, India; Department of Microbiology (S Gupta MD), Rama Medical College Hospital and Research Centre, Kanpur, India; Department of Public Health (B Gupta PhD), Torrens University Australia, Melbourne, VIC, Australia; Department of Toxicology (S Gupta PhD), Shriram Institute for Industrial Research, Delhi, India; Faculty of Health (Prof B Gutiérrez PhD), University of the Valley, Cali, Colombia; Department of Community Medicine (P Halder MD, F Samad MPH), Department of Pediatrics (J Kumar MD), Department of Community Medicine & School of Public Health (P Kumar PhD), Post Graduate Institute of Medical Education and Research, Chandigarh, India; Training Center (R M Hama Salih PhD), Kurdistan Higher Council of Medical Specialties, Sulaymaniyah, Iraq; Department of Fixed Prosthodontics (M Hamed DSc), Faculty of Nursing (M Shaban PhD), Cairo University, Cairo, Egypt; Department of Neurosurgery (A M Hamouda MD), Mayo Clinic, Rochester, USA;

Department of Computer and Information Science (M Haq PhD), Majmaah University, Al Majmaah, Saudi Arabia; Faculty of Dentistry (S Hasan MDS), Jamia Millia Islamia, New Delhi, India; Department of Food Technology and Nutrition Science (T Hasan PhD), Noakhali Science and Technology University, Noakhali, Bangladesh; Department of Ophthalmology (H Hasani MD), Alborz University of Medical Sciences, Karaj, Iran; Department of Periodontics (N T Hashim PhD), RAK Medical & Health Sciences University, Ras Al Khaimah, United Arab Emirates; Department of Periodontia & Community Dentistry (A Hashmi MDS), Aligarh Muslim University, Aligarh, India; Department of Pharmaceutical Technology (Prof M S Hasnain PhD), Adamas University, Kolkata, India; Department of Oral and Maxillofacial Surgery (F He PhD), Key Laboratory of Shaanxi Province for Craniofacial Precision Medicine Research, Xi'An, China; Independent Consultant, Santa Clara, CA, USA (G Heidari MD); Departamento de Salud Oral (Department of Oral Health) (B Y Herrera-Serna PhD), Universidad Autónoma de Manizales (Autonomous University of Manizales), Manizales, Colombia; Department of Medical Biochemical Analysis (K Heydaryan PhD), Cihan University-Erbil, Erbil, Iraq; Department of Microbiology (K Hezam PhD), Taiz University, Taiz, Yemen; School of Medicine (K Hezam PhD), Nankai University, Tianjin, China; School of Dentistry (N Hoan DDS), Hanoi Medical University, Hanoi, Viet Nam; Faculty of Health (M Hobbs PhD), University of Canterbury, Christchurch, New Zealand; Kasturba Medical College Mangalore (R Holla MD, M Rao MD, Prof B Unnikrishnan MD), Department of Community Medicine (S Kini B MD), Manipal College of Dental Sciences (Prof R A Radhakrishnan PhD), Manipal College of Dental Sciences, Mangalore (Prof P K Shetty MDS), Department of Oral Pathology (M C Solomon PhD), Department of Pathology (S Sreeram MD), Manipal Academy of Higher Education, Manipal, India; Department of Public Health (S Hossain MPH), University of Science and Technology Chittagong (USTC), Chattogram, Bangladesh; Department of Public Health (S Hossain MPH), Daffodil International University (DIU), Dhaka, Bangladesh; School of Engineering and Technology (Prof M Hosseinzadeh PhD), Institute for Global Health Innovations (C T Nguyen MPH, D C Nguyen MD), Duy Tan University, Da Nang, Viet Nam; Department of AI (Prof M Hosseinzadeh PhD), Galgotias University, Greater Noida, India; Department of Legal Medicine and Bioethics (Prof S Hostiuc PhD), Department of Dermatology (M Tampa PhD), Carol Davila University of Medicine and Pharmacy, Bucharest, Romania; Department of Clinical Legal Medicine (Prof S Hostiuc PhD), National Institute of Legal Medicine Mina Minovici, Bucharest, Romania; Department of Occupational Safety and Health (Prof B Hwang PhD), China Medical University, Taiwan, Taichung, Taiwan; Department of Biology (K S Ibrahim PhD), University of Zakho, Zakho, Iraq; West Africa RCC (O S Ilesanmi PhD), Africa Centre for Disease Control and Prevention, Abuja, Nigeria; Faculty of Medicine (I M Ilic PhD), University of Belgrade, Belgrade, Serbia; Faculty of Medical Sciences (Prof M D Ilic PhD), University of Kragujevac, Kragujevac, Serbia; Department of Health Information Management (D Iqbal PhD), Buraydah Private Colleges, Al-Qassim, Saudi Arabia; School of Pharmacy (M Islam PhD), BRAC University, Dhaka, Bangladesh; Department of General Surgery and Medical-Surgical Specialties (Prof G Isola PhD), Department of Clinical and Experimental Medicine (G La Rosa PhD), University of Catania, Catania, Italy; Department of Hospital Medicine (H Ito PhD), University of Tsukuba, Tsukuba, Japan; College of Medicine and Health Sciences (H Jahrami PhD), Department of Pharmacology and Therapeutics (Prof K Sridharan DM), Arabian Gulf University, Manama, Bahrain; Government Hospitals, Manama, Bahrain (H Jahrami PhD); Department of Health and Safety (A A Jairoun PhD), Dubai Municipality, Dubai, United Arab Emirates; Section of Economic & Social Sciences, Humanities & Arts (Prof M Jakovljevic PhD), The World Academy of Sciences UNESCO-TWAS, Trieste, Italy; Shaanxi University of Technology, Hanzhong, China (Prof M Jakovljevic PhD); Department of Nursing (S Jalal PhD), King Faisal University, Al-Ahsa, Saudi Arabia; Department of Environmental Engineering (Prof R Jalilzadeh Yengejeh PhD), Islamic Azad University, Ahvaz, Iran; Division of Medical Research (R Janardhanan PhD), Sri Ramaswamy Memorial Institute of Science and Technology, Kattankulathur, India; The Medical City for Military and Security Services

School, Muscat, Oman (S Jayapal PhD); Department of Oral Medicine and Periodontology (Prof R D Jayasinghe MS), University of Peradeniya, Peradeniya, Sri Lanka; National Cancer Control Institute (W Jeong PhD), National Cancer Center, Goyang-Si, South Korea; Division of Dentistry (E Joury PhD), University of Manchester, Manchester, UK; Department of Family Medicine and Public Health (J J Jozwiak PhD), University of Opole, Opole, Poland; School of Computer Science and Engineering (T K PhD), RV University, Bengaluru, India; Research Department (Z Kabir PhD), TobaccoFree Research Institute Ireland, Dublin, Ireland; School of Public Health (Z Kabir PhD), University College Cork, Cork, Ireland; Department of Oral and Maxillofacial Pathology and Microbiology (V Kadashetti MDS), Krishna Vishwa Vidyapeeth Deemed to be University, Karad, India; Faculty of Science (Prof A Kadri PhD), Al-Baha University, Saudi Arabia, Al-Baha, Saudi Arabia; Independent Consultant, Pune, India (P V Kakodkar MDS); Department of Periodontology (E Kandaswamy MS), Louisiana State University Health Sciences Center, New Orleans, LA, USA; Office of the Executive Director (Prof K K Kanmodi MPH), Cephas Health Research Initiative Inc., Ibadan, Nigeria; Department of Public Health (Prof K K Kanmodi MPH), Thomas Adewumi University, Oko, Nigeria; The Hansjörg Wyss Department of Plastic and Reconstructive Surgery (R S Kantar MD), Department of Epidemiology, NYU School of Global Public Health (K P Nagdeo DDS), Rory Meyers College of Nursing (X Qi PhD), New York University, New York, NY, USA; Department of Health Policy and Community Health (I Karibayeva PhD), Georgia Southern University, Statesboro, GA, USA; Department of Research Management (I Karibayeva PhD, M Kulimbet MSc), Research Institute of Cardiology and Internal Diseases, Almaty, Kazakhstan; Chair and Department of Medical Microbiology (Prof T M Karpiński DDS), Poznan University of Medical Sciences, Poznan, Poland; Department of Biomedical Sciences (G G Kassa MSc), Aksum University, Aksum, Ethiopia; Clinical Anatomy Department (B Kassie Abeje MSc), Debre Tabor University, Addis Ababa, Ethiopia; Surgery Research Unit (Prof J H Kauppila MD), University of Oulu, Oulu, Finland; Department of Molecular Medicine and Surgery (Prof J H Kauppila MD), Karolinska Institute, Stockholm, Sweden; Center for Molecular Biosciences and Medical Sciences (S A Kayode MSc), Department of Microbiology (Y D Oluwafemi PhD), Department of Mathematical and Computer Sciences (O Peter PhD), University of Medical Sciences, Ondo, Ondo, Nigeria; Amity Institute of Forensic Sciences (H Khajuria PhD, B P Nayak PhD, P Puri PhD), Amity Institute of Public Health and Hospital Administration (A Koul MHA, R Ruhil PhD, M Shannawaz PhD), Amity University, Noida, India; Department of Cardiology (Z Khan MD), University of South Wales, Treforest, UK; Department of Cardiology (Z Khan MD), University of Buckingham, Buckingham, UK; Department of Biotechnology and Applied Clinical Sciences (H Khan PhD), University of L'Aquila, L'Aquila, Italy; Riphah School of Computing & Innovation (Prof M Khan PhD), Riphah International University, Lahore, Pakistan; Department of Pharmacology (S U Khasbage MD), All India Institute of Medical Sciences, Raipur, India; Department of Biochemistry (F Khidri PhD), Liaquat University Of Medical and Health Sciences, Jamshoro, Pakistan; Department of Biomedical Informatics (Y Kim BS), University College (Prof U Saeed PhD), Korea University, Seoul, South Korea; Department of Transdisciplinary Medicine (S Kim PhD), Healthcare AI Research Institute (S Kim PhD), Seoul National University Hospital, Seoul, South Korea; Health and Healing Research, Education, and Service, Inc., Boston, MA, USA (R W Kimokoti MD); Millennium Prevention, Inc., Westwood, MA, USA (R W Kimokoti MD); School of Health Sciences (Prof A Kisa PhD), Kristiania University College, Oslo, Norway; Department of International Health and Sustainable Development (Prof A Kisa PhD), Tulane University, New Orleans, LA, USA (E Lytvyak MD); Department of Otorhinolaryngology Head and Neck Surgery (Prof T Kleinjung MD), University of Zürich, Zurich, Switzerland; Department of Public Health and Community Medicine (P Kodali PhD), Central University of Kerala, Kasaragod, India; Center for Tobacco Control Research and Education (P Kodali PhD), Department of Dental Public Health (R Singhania BDS), University of California San Francisco, San Francisco, CA, USA; Department of Anthropology (Prof K Krishan PhD), Panjab University, Chandigarh, India;

Department of Physiology (B Krishnan MD), Sree Balaji Medical College and Hospital, Chennai, India; Rajendra Institute of Medical Sciences (A Kujur PharmD), Ministry of Health and Family Welfare, Ranchi, India; Health Research Institute (M Kulimbet MSc), Al Farabi Kazakh National University, Almaty, Kazakhstan; Department of Health Administration and Education (E Kumah PhD), University of Education, Winneba, Ghana; Lady Hardinge Medical College (M Kumar MD), Lady Hardinge Medical College, New Delhi, India; Department of Medicine and Surgery (S Kumar MD), Mayo Clinic, Rochester, MN, USA; Department of Biochemistry (A Kumar PhD), Department of Dentistry (Prof A Singh MDS), All India Institute of Medical Sciences, Bhopal, India; Department of Mathematics (Prof K Kumar PhD), Amity University Haryana, Gurugram, India; Dow Medical College (N Kumar BS), Dow University of Health Sciences, Karachi, Pakistan; Department of Community Medicine (D Kumar MD), Rajendra Institute of Medical Sciences, Ranchi, India; Faculty of Education (S Kumar PhD), Shree Guru Gobind Singh Tricentenary University, Gurugram, India; Lincoln Institute of Rural and Coastal Health (O P Kurmi PhD), University of Lincoln, Lincoln, UK; Department of Medicine (O P Kurmi PhD), McMaster University, Hamilton, ON, Canada; Department of Microbiology (P K Kushawaha PhD, A Singh PhD), Central University of Punjab, Bathinda, India; Department of Public Health and Epidemiology (D Kusuma DSc), Khalifa University of Science and Technology, Abu Dhabi, United Arab Emirates; Faculty of Public Health (D Kusuma DSc, Prof I Trihandini PhD), University of Indonesia, Depok, Indonesia; Department of Psychology (Prof T Lajunen PhD), Norwegian University of Science and Technology, Trondheim, Norway; Clinical Studies and Trials Unit (Development Research) (A Satija PhD), Indian Council of Medical Research, New Delhi, India (D K Lal MD); School of Dentistry (R Lalloo PhD), School of Agriculture and Food Sustainability (Prof L Wang PhD), The University of Queensland, Brisbane, QLD, Australia; Department of Otorhinolaryngology (Prof S Lasrado MS), Father Muller Medical College, Mangalore, India; Department of Precision Medicine (Prof S Lee MD), Sungkyunkwan University, Suwon-Si, South Korea; Faculty of Science (E Leong PhD), Universiti Brunei Darussalam (University of Brunei Darussalam), Bandar Seri Begawan, Brunei; Department of Psychiatry & Human Behavior (M LI PhD), University of California Irvine, Irvine, CA, USA; Department of Health Promotion and Health Education (M Li PhD), National Taiwan Normal University, Taipei, Taiwan; Clinical Sciences (A Liaqat FCPS), Shaqra University, Sharqa, Saudi Arabia; Department of Evidence-Based Stomatology (L Liu DDS), Clinical Research Center for Stomatology, Chengdu, China; Faculty of Medicine OWL (E Llanaj PhD), Bielefeld University, Bielefeld, Germany; Faculty of Medicine (A Lövgren PhD), Umeå University, Umeå, Sweden; Department of Medicine (E Lytvyak MD), University of Alberta, Edmonton, AB, Canada; Faculty of Pharmacy (Prof H I M. Amin PhD), Tishk International University, Erbil, Iraq; Department of Pharmaceutical Science (Prof H I M. Amin PhD), University of Eastern Piedmont, Novara, Italy; Faculty of Dental Medicine and Oral Health Sciences (S Ma DDS), McGill University, Montreal, QC, Canada; Center for Global Health (K Ma DDS), Children's Hospital of Philadelphia (B B Tabor MD), University of Pennsylvania, Philadelphia, PA, USA; Department of Periodontology (Prof M Machoy-Rakoczy PhD), Pomeranian Medical University, Szczecin, Poland; Department of Physiology and Biochemistry (M Mackei PhD), University of Veterinary Medicine Budapest, Budapest, Hungary; Center for Integrative Omics Data Science (ciods) (I Madar PhD), Yenepoya Research Centre (B Rekadwad PhD), Yenepoya University, Mangalore, India; Thamar University, Dhamar, Yemen (Prof A A Madfa PhD); Ophthalmology Department (M Magdy Abd El Razek PhD), Dr. Soliman Fakeeh Hospital, Riyadh, Saudi Arabia; Discipline of Pharmacy (M Maharajan PhD), James Cook University, Cairns, QLD, Australia; Research Center (N H Mahmood PhD), Cihan University-Sulaimaniya, Sulaymaniyah, Iraq; Maternal and Child Health Division (MCHD) (T Mallick MSc), International Centre for Diarrhoeal Disease Research, Bangladesh, Dhaka, Bangladesh; College of Medicine and Health Sciences (V Marakala MD), National University of Science and Technology, Oman, Sohar, Oman; Department of Clinical and Experimental Medicine (Prof S Masi PhD), University of Pisa, Pisa, Italy; Department of Health Policy

Research (M R Mathur PhD), Public Health Foundation of India, Gurugram, India; Institute of Population Health Sciences (M R Mathur PhD), University of Liverpool, Liverpool, UK; Department of Community Medicine (M Mathur MD), Geetanjali Medical College and Hospital in Udaipur India, Udaipur, India; Department of Dental Surgery (J Matys PhD), Wroclaw Medical University, Wrocław, Poland; National Heart, Lung, and Blood Institute, Bethesda, MD, USA (Prof R Mehboob PhD); Research and Development Department (Prof R Mehboob PhD), Lahore Medical Research Center, Lahore, Pakistan; Turning Point, Eastern Health Clinical School (S Melwani PhD), Monash University, Melbourne, VIC, Australia; University Centre Varazdin (T Mestrovic PhD), University North, Varazdin, Croatia; Dermatology Unit (A Michelerio PhD), Fondazione IRCCS Policlinico San Matteo, Pavia, Italy; Multidisciplinary Department of Medical-Surgical and Dental Specialties (G Minervini PhD), University of Campania Luigi Vanvitelli, Naples, Italy; Department of Health Education and Promotion (H Mohamadian PhD), Clinical Sciences Research Institute (Z Shokati Eshkiki PhD), Ahvaz Jundishapur University of Medical Sciences, Ahvaz, Iran; Molecular Biology Unit (N S Mohamed MSc), Bio-Statistical and Molecular Biology Department (N S Mohamed MSc), Sirius Training and Research Centre, Khartoum, Sudan; Medical Microbiology Department (Prof Y Mohammed FWACP), Department of Pharmaceutical Microbiology and Biotechnology (S Shuaibu PhD), Usmanu Danfodiyo University, Sokoto, Sokoto, Nigeria; Medical Microbiology Department (Prof Y Mohammed FWACP), Usmanu Danfodiyo University Teaching Hospital, Sokoto, Nigeria; Department of Applied Biology (Y Mohanta PhD), University of Science and Technology Meghalaya, Ri-Bhoi, India; Department of Physiology (H Mondal MD), College of Nursing (V Patidar MSc), All India Institute of Medical Sciences, Deoghar, India; Computer, Electrical, and Mathematical Sciences and Engineering Division (P Moraga PhD), King Abdullah University of Science and Technology, Thuwal, Saudi Arabia; Department of Public Health (Prof R S Moreira PhD), Oswaldo Cruz Foundation, Recife, Brazil; Department of Public Health (Prof R S Moreira PhD), Federal University of Pernambuco, Recife, Brazil; Faculty of Biotechnologies (biotech) (Prof A Mousavi Khaneghah PhD), ITMO University, Saint Petersburg, Russia; Department of Radiology (A Msherghi MD), University of Tripoli, Tripoli, Libya; PMAS Arid Agriculture University Rawalpindi, Rawalpindi, Pakistan (R Mubarak MSc); Unit of Pharmacotherapy, Epidemiology and Economics (Prof S Mubarik PhD), University of Groningen (Rijksuniversiteit Groningen), Groningen, Netherlands; Centre for Early Childhood Caries Research (Prof S Murugan PhD), Sri Ramachandra University, Chennai, India; Santosh University, New Delhi, India (Prof S Murugan PhD); School of Public Health (D Musoke PhD), Makerere University, Kampala, Uganda; Department of Pathology and Microbiology (S I Mustafa PhD), Duhok University, Duhok, Iraq; College of Medicine and Public Health (G R Naik PhD), Department of Nursing and Health Sciences (S Shorofi PhD), Flinders University, Adelaide, SA, Australia; Department of Computer Science and IT (G R Naik PhD), Torrens University, Adelaide, SA, Australia; Community Health Centre (L M Nair MPH), National Health Mission, Vellanikkara, India; Department of Oral and Craniomaxillofacial Surgery (P Nalabothu PhD), Department of Pediatrics Oral Health and Orthodontics (P Nalabothu PhD), University of Basel, Basel, Switzerland; School of Dentistry (Prof G G Nascimento PhD), University of Utah, Salt Lake City, UT, USA; Department of Restorative and Prosthetic Dental Sciences (M Nassani PhD), Dar Al Uloom University, Riyadh, Saudi Arabia; Department of Biological Sciences and Chemistry (Z Naureen PhD), University of Nizwa, Nizwa, Oman; Kent State University (K Nayak MPH), Kent State University, Kent, OH, USA; Department of Pediatrics (J Nazari MD), Arak University of Medical Sciences, Arak, Iran; National Centre for Suicide Research and Prevention (S P Neupane PhD), University of Oslo, Oslo, Norway; Oral Health Centre of Expertise in Rogaland (S P Neupane PhD), Oral Health Center of Expertise in Rogaland, Stavanger, Norway; HSC, Cancer Institute (S Nezam MDS), West Virginia University, Morgantown, WV, USA; Milken Institute School of Public Health (S Nezam MDS), George Washington University, Washington Dc, DC, USA; Institute for Advanced Study in Technology (L H Nguyen PhD), Ton Duc Thang University, Ho Chi Minh City, Viet

Nam; International Institute of Training and Research (instar) (T T Nguyen MPH), University of Medicine and Pharmacy, Vietnam National University (UMP-VNU), Hanoi, Viet Nam; School of Medicine, Health Research Institute (V Niranjan PhD), University of Limerick, Limerick, Ireland; Center for Public Health (L A Nnyanzi PhD), Teesside University, Middlesbrough, UK; Department of Social and Preventive Medicine (M Noushad PhD), University of Malaya, Kuala Lumpur, Malaysia; School of Health Sciences (N Nurelhuda PhD), Hamdan Bin Mohammed Smart University, Dubai, United Arab Emirates; Dalla Lana School of Public Health (N Nurelhuda PhD), University of Toronto, Toronto, ON, Canada; Department of Applied Economics and Quantitative Analysis (Prof B Oancea PhD), University of Bucharest, Bucharest, Romania; Bioinformatics Department (Prof B Oancea PhD), National Institute of Research and Development for Biological Sciences, Bucharest, Romania; One Health Research Group (Prof E Ortiz-Prado PhD), Universidad De Las Américas, Quito, Ecuador; National School of Public Health (A Padron-Monedero PhD), Institute of Health Carlos III, Madrid, Spain; Centre for Biotechnology (S K Panda PhD, A Sahoo PhD), Siksha 'O' Anusandhan University (Prof T R Rautray PhD), Institute of Medical Sciences and SUM Hospital (A Sahoo PhD), Siksha 'O' Anusandhan Deemed to be University, Bhubaneswar, India; Division of Ophthalmology & Visual Sciences (Prof G D Panos MD), University of Nottingham, Nottingham, UK; First Department of Ophthalmology (Prof G D Panos MD), Aristotle University of Thessaloniki, Thessaloniki, Greece; Vision and Eye Research Institute (Prof S Pardhan PhD), Anglia Ruskin University, Cambridge, UK; Division of Health Policy and Management (R R Parikh MD), University of Minnesota, Minneapolis, MN, USA; Medical Big Data Research Center (S Park PhD), Seoul National University, Seoul, South Korea; Department of Forensic Medicine and Toxicology (P Parmar MD), All India Institute of Medical Sciences, Hyderabad, India; Department of Life Sciences and Public Health (E Pascucci MSc), Catholic University of Sacred Heart, Rome, Italy; Department of Computer Science and Bioscience (M Patel PhD), Marwadi University, Rajkot, India; Ingenus Pharmaceuticals, LLC (S Patel MS), Long Island University, Brooklyn, Fairfield, NJ, USA; Department of Physical Therapy (M L Patel DPT), Right Motion Physical Therapy PT PC., Woodbury, NY, USA; Faculty of Medicine and Health (J Patel MChD), University of Leeds, Leeds, UK; College of Dental Medicine (Prof S Patil PhD), Roseman University of Health Sciences, South Jordan, UT, USA; Department of Genetics (S Pawar PhD), Yale University, New Haven, CT, USA; Research Workgroup (P P Pietkiewicz PhD), Polish Dermatoscopy Group, Poznan, Poland; Skin Cancer Unit (P P Pietkiewicz PhD), Zwierzyniecka Medical Center, Poznan, Poland; Department of Dental Public Health (P Porwal MSc), Pushpagiri Institute of Medical Sciences, Tiruvella, India; Centro de Investigaciones Clinicas (Clinical Research Center) (S I Prada PhD), Valle del Lili Foundation (Fundación Valle del Lili), Cali, Colombia; Department of Centro PROESA (S I Prada PhD), Universidad ICESI, Cali, Colombia; Egas Moniz Center for Interdisciplinary Research (ciiem) (Prof L Proença PhD), Instituto Universitário Egas Moniz, Almada, Portugal; Department of Medical Instrumentation Techniques Engineering (N H Qasim DSc), Al-Rafidain University, Baghdad, Iraq; Department of Mathematics and Informatics (N H Qasim DSc), Luhansk Taras Shevchenko National University, Luhansk, Ukraine; Xiangya Hospital (C Quan PhD), Central South University, Changsha, China; Faculty of Nursing (N Qur'aniati PhD), Department of Oral Medicine (A Sarasati PhD), Universitas Airlangga, Surabaya, Indonesia; Research Center in Advancing Community Healthcare, Surabaya, Indonesia (N Qur'aniati PhD); Department of Medical Oncology (Prof V Radhakrishnan MD), Cancer Institute (W.I.A), Chennai, India; Oman Dental College, Muscat, Oman (Prof R A Radhakrishnan PhD); Department of Medical Biology (Prof F Rahim PhD), Kocaeli University, Izmit, Türkiye; Internal Medical Faculty (Prof F Rahim PhD), Osh State University, Osh, Kyrgyzstan; Department of Population Science and Human Resource Development (Prof M Rahman DrPH), University of Rajshahi, Rajshahi, Bangladesh; Faculty of Medicine (H Rahmoune PhD), Lirssei Research Lab (H Rahmoune PhD), University of Setif Algeria, Setif, Algeria; Department of Nursing Science (A S Rajah MSc), Nigerian Institute of Medical Research,

Kano, Nigeria; Department of Chemical Engineering and Processing (Prof R Rakoczy DSc), West Pomeranian University of Technology in Szczecin, Szczecin, Poland; Department of Radiology (S Ramasamy MD), Stanford University, Stanford, CA, USA; All India Institute of Medical Sciences, Bilaspur, India (L Rana MD); Nitte (deemed To Be University), AB Shetty Memorial Institute of Dental Sciences (K Rao PhD), Nitte University, Mangalore, India; Department of Oral Pathology, Microbiology and Forensic Odontology (S J Rao MDS), Sharavathi Dental College and Hospital, Shimogga, India; Department of Family Medicine (Prof D Rathish PhD), Department of Community Medicine (N Wickramasinghe MD), Rajarata University of Sri Lanka, Anuradhapura, Sri Lanka; Department of Primary Care and Public Health (Prof S Rawaf MD), Imperial College London, London, UK; Department of Academic Public Health England (Prof S Rawaf MD), Public Health England, London, UK; Department of Biological Sciences (Prof E M Redwan PhD), King Abdulaziz University, Jeddah, Egypt; Department of Protein Research (Prof E M Redwan PhD), Research and Academic Institution, Alexandria, Egypt; Department of Public Health (A Riad PhD), Masaryk University, Brno, Czech Republic; Faculty of Medicine (M J Rios-Blancas DSc), National Autonomous University of Mexico, Mexico City, Mexico; Department of Digital Health (M J Rios-Blancas DSc), Carlos Slim Foundation, Mexico City, Mexico; Community Health Department (Prof H A L Rocha PhD), Federal University of Ceará, Fortaleza, Brazil; Department of Population Health (Prof C Rojanaworarit PhD), Hofstra University, Hempstead, NY, USA; Faculty of Medicine (Prof K Saad PhD), Assiut University, Assiut, Egypt; Tabriz University of Medical Sciences, Tabriz, Iran (F Sadeghi-Ghyassi PhD); Department of Pharmaceutical Chemistry (Prof M Saeb PhD), International Medical University, Gdańsk, Poland; Széchenyi István University, Győr, Hungary (Prof U Saeed PhD); Community Medicine and Family Medicine (D Sahoo MD), All India Institute of Medical Sciences, Bibinagar, Hyderabad, India; LMU-Munich, Munich, Germany (J W Sakshaug PhD); Institute for Employment Research, Nuremberg, Germany (J W Sakshaug PhD); Department of Nutrition (Prof L B Salaroli PhD), Federal University of Espirito Santo, Vitória, Brazil; Entomology VectorLink (F S Samura MPH), Njala University, Freetown, Sierra Leone; Department of Entomology (A M Samy PhD), Medical Ain Shams Research Institute (MASRI) (A M Samy PhD), Ain Shams University, Cairo, Egypt; Department of Genomics and Biotechnology (S Sankar PhD), Madha Medical College and Research Institute, Chennai, India; National Dental Research Institute Singapore (S Sarawagi MSc), National Dental Centre Singapore, Singapore, Singapore; Health Services and Systems Research Programme (S Sarawagi MSc), Duke-NUS Medical School, Singapore, Singapore; Department of Medical and Surgical Sciences (M Sassano MD), University of Bologna, Bologna, Italy; UGC Centre of Advanced Study in Psychology (Prof M Satpathy PhD), Utkal University, Bhubaneswar, India; Udyam-Global Association for Sustainable Development, Bhubaneswar, India (Prof M Satpathy PhD); Department of Food Processing and Nutrition (J Saulam PhD), Karnataka State Akkamahadevi Women University, Vijayapura, India; Carnegie Mellon University (A Saurabh MS), Carnegie Mellon University, Pittsburgh, PA, USA; Institute of Radiology, Neuroradiology and Interventional Therapy (Prof B M Schaarschmidt MD), University of Erlangen-Nuremberg, Bayreuth, Germany; Clinic for Conservative Dentistry, Periodontology and Digital Dentistry (Prof F Schwendicke PhD), University Hospital Munich, Munich, Germany; Faculty of Dentistry (Prof S Selvaraj PhD), University of Puthisastra, Phnom Penh, Cambodia; Emergency Department (S Senthilkumaran PhD), Manian Medical Centre, Erode, India; Department of Chemistry (H R Shahsavari PhD), Institute for Advanced Studies in Basic Sciences (IASBS), Zanjan, Iran; Faculty of Dentistry (Prof M Shahzad PhD), Zarqa University, Zarqa, Jordan; Institute of Basic Medical Sciences (Prof M Shahzad PhD), Khyber Medical University, Peshawar, Pakistan; Independent Consultant, Karachi, Pakistan (M A Shaikh MD); Department of Pharmacology (M Shamim MBBS, S Singh MD), All India Institute of Medical Sciences, Jodhpur, India; Department of Microbiology and Molecular Genetics (N Sharif MSc), University of Vermont, Burlington, VT, USA; Department of Microbiology (N Sharif MSc),

Jahangirnagar University, Dhaka, Bangladesh; Department for Evidence-Based Medicine and Evaluation (A Sharifan PharmD), University for Continuing Education Krems, Krems, Austria; Department of Chemistry (K Sharma PhD), Graphic Era (Deemed to be University), Dehradun, India; Department of Nursing (B B Shashamo MSc), Arba Minch University, Arba Minch, Ethiopia; Alva's Insitute of Medical Sciences & Research Centre (Prof M Shetty MD), Rajiv Gandhi University of Health Sciences, Moodubidire, India; Department of Biomedical Sciences (N M Shimaponda-Mataa PhD), University of Zambia, Lusaka, Zambia; School of Biotechnology (K Shivangi MSc), Gautam Buddha University, Gautam Buddh Nagar, India; Department of Health Promotion and Behavior (D O Shomuyiwa BPharm), University System of Georgia, Athens, GA, USA; Department of Public Health Medicine (S N Shongwe MPH), University of KwaZulu-Natal, Durban, South Africa; Department of Research and Academics (S Shrestha PhD), Kathmandu Cancer Center, Bhaktapur, Nepal; Person-Centered Research (S Shrestha PhD), Monash University, Box Hill, VIC, Australia; Sport Physical Activity and Health Research & Innovation Center (sprint) (Prof L M L R Silva PhD), Polytechnic Institute of Guarda, Guarda, Portugal; Department of RISE Health (Prof L M L R Silva PhD), University of Beira Interior, Covilhã, Portugal; School of Medicine (Prof J A Singh MD), Baylor College of Medicine, Houston, TX, USA; Department of Medicine Service (Prof J A Singh MD), US Department of Veterans Affairs (VA), Houston, TX, USA; School of Pharmaceutical Sciences, Faculty of Pharmacy (H Singh PhD), IFTM University, Moradabad, India; Institute of Medical Sciences (S Singh PhD), Banaras Hindu University, Varanasi, India; Buck Institute for Research on Aging, Novato, CA, USA (P Singh PhD); The Institute for Drug Research (P Singh PhD), The Hebrew University of Jerusalem, Jerusalem, Israel; Mehta Family School of Biosciences and Biomedical Engineering (S Singh MSc), Indian Institute of Technology Indore, Indore, India; Four States Dental Care, Neosho, MO, USA (R Singhania BDS); Medical Research, SRM Medical College Hospital and Research Centre (G Sivan PhD), Sri Ramaswamy Memorial Institute of Science and Technology, Chengalpettu, India; Bahrain Defence Force Royal Medical Services (G Sivaramakrishnan MSD), Bahrain Defence Force Royal Medical Services in Bahrain, Riffa, Bahrain; Department of Oral and Maxillofacial Surgery (C S N PhD), Jagadguru Sri Shivarathreeswara University, Mysore, India; Department of Systemic Pathology (R Solanki MD), Touro College of Osteopathic Medicine, Middletown, NY, USA; Department of Pathology (R Solanki MD), American University of the Caribbean School of Medicine, Cupecoy, Saint Martin; Department of Diagnostic and Interventional Radiology (Q D Strotzer MD), University Hospital Regensburg, Regensburg, Germany; Institute of Nutrition and Food Science (M Sultana PhD), University of Dhaka, Dhaka, Bangladesh; Data Science Institute (Prof J Sun PhD), University of Technology Sydney, Sydney, NSW, Australia; Heart Research Institute (Prof J Sun PhD), University of Sydney, Sydney, QLD, Australia; School of Dentistry and Oral Health (S K Tadakamadla PhD), Griffith University, Gold Coast, QLD, Australia; Department of Dentistry and Oral Health (J Tadakamadla PhD), La Trobe University, Bendigo, VIC, Australia; Feinstein Institutes for Medical Research (A Tahavvori MD), Urmia University of Medical Sciences, Manhasset, NY, USA; Department of Environmental, Agricultural and Occupational Health (J Taiba PhD), University of Nebraska Medical Center, Omaha, NE, USA; Department of Dermato-Venereology (M Tampa PhD), Dr. Victor Babes Clinical Hospital of Infectious Diseases and Tropical Diseases, Bucharest, Romania; Department of Otorhinolaryngology–head & Neck Surgery (B Tan MBBS), Singapore General Hospital, Singapore, Singapore; College of Pharmacy (R Temsah PharmD), Alfaisal University, Riyadh, Saudi Arabia; Dental Department (A Temsah BDS), Specialized Medical Center Hospital, Riyadh, Saudi Arabia; Outpatient Department (D R Terefa MSc), Wollega University, Bedele Town, Ethiopia; Department of Economics (I Tharwat PhD), The American University in Cairo, Cairo, Egypt; Cardiovascular Diseases Research Center, School of Dentistry (A Tiyuri PhD), School of Dentistry (A Tiyuri PhD), Birjand University of Medical Sciences, Birjand, Iran; Department of Social Determinants of Health (R Toghroli PhD), Hormozgan University of Medical Sciences, Bandar Abbas, Iran; Global Surgery Department (A Touko MSc), Data Science Center for the

Study of Surgery, Injury and Equity in Africa, Buea, Cameroon; Department of Internal Medicine (T H Tran MD), University of Medicine and Pharmacy at Ho Chi Minh City, Ho Chi Minh City, Viet Nam; Department of Business Analytics (T H Tran MD), University of Massachusetts Dartmouth, Dartmouth, MA, USA; Harvard T.h. Chan School of Public Health (M Tuffour Amirikah MD), Harvard University, Boston, MA, USA; Department of Biology and Biochemistry (S Ullah MSc), University of Houston, Houston, TX, USA; Independent Consultant, St. Augustine, Trinidad and Tobago (S Umakanthan MD); North Central Regional Health Authority, Champ Fleurs, Trinidad and Tobago (S Umakanthan MD); Research and Education Department (O Uwishema MD), Oli Health Magazine Organization (OHMO), Kigali, Rwanda; Department of Orthodontics (H Uzunçıbuk PhD), University of Trakya, Edirne, Türkiye; Department of Public Health and Epidemiology (O Varga PhD), University of Debrecen, Debrecen, Hungary; School of Dentistry and Medical Sciences (P Veginadu PhD), Charles Sturt University, Wagga Wagga, NSW, Australia; Population Oral Health (P Veginadu PhD), University of Sydney, Sydney, NSW, Australia; Department of Community Medicine and Family Medicine (U Venkatesh MD), All India Institute of Medical Sciences, Gorakhpur, India; College of Medicine (N Viet-Nhi PhD), Taipei Medical University, Taipei, Taiwan; Centro de Investigación en Nutrición y Salud (Prof V E Villalobos-Daniel PhD), National Institute of Public Health, Mexico City, Mexico; Institute of Family Medicine and Public Health (A Voit MSc), University of Tartu, Tartu, Estonia; Section for Oral Health (A Voit MSc), Heidelberg University Hospital, Heidelberg, Germany; Department of Medical Oncology (S Volovat PhD), University of Medicine and Pharmacy "Grigore T Popa" Iasi, Iaşi, Romania; Department of Medical Oncology (S Volovat PhD), Regional Institute of Oncology, Iaşi, Romania; Department of Education (R Wadhwa PhD), Akal University, Bathinda, India; National Institute of Health Data Science (W Wang PhD), Peking University, Beijing, China; Health Unit Organization (T S Warouw DrPH), National Research and Innovation Agency (BRIN), Bogor, Indonesia; Department of Adult Health Nursing/school of Nursing (D D Weldebrhan MSc), Mekelle University, Mekelle, Ethiopia; Research Organisation (A Wireko MD), Inter-Continental Omni-Research in Medicine Collaborative, Berlin, Germany; School of Public Health Sciences and Technology (P Yadav PhD), Malla Reddy Vishwavidyapeeth, Hyderabad, India; Department of Community Medicine (S Yahoo MD), Apollo Institute of Medical Sciences and Research, Hyderabad, India; Center for Postgraduate Education and Training (N Yamada MD), National Center for Child Health and Development, Tokyo, Japan; Department of Neurosurgery (K Yangi MD), St. Joseph's Hospital and Medical Center, Phoenix, AZ, USA; KHANA Center for Population Health Research, Phnom Penh, Cambodia (Prof S Yi PhD); Department of Pediatrics (Prof D Yon MD), Kyung Hee University, Seoul, South Korea; Department of Epidemiology and Cancer Registry Sector (Prof V Zadnik PhD), Institute of Oncology Ljubljana, Ljubljana, Slovenia; School of Dentistry (Prof M S Zafar PhD), University of Jordan, Amman, Jordan; Research and Development Department (I Zare BSc), Sina Medical Biochemistry Technologies, Shiraz, Iran; Gilbert and Rose-Marie Chagoury School of Medicine (Z Zibara BS), Lebanese American University, Byblos, Lebanon; Department of Biochemistry and Pharmacogenomics (M Zielińska MPharm), Medical University of Warsaw, Warsaw, Poland; Department of Building Engineering and Environment (S H Zyoud PhD), Department of Civil Engineering and Sustainable Structures (S H Zyoud PhD), Palestine Technical University (Kadoorie), Tulkarem, Palestine

Authors' Contributions

Providing data or critical feedback on data sources

Abdullah, Belayneh Jejaw Abate, Hedayat Abbastabar, Hassan A. Abdou, Rizwan Suliankatchi Abdulkader, Richard Gyan Aboagye, Lucas Guimarães Abreu, Sawsan Abuhammad, Anirudh Balakrishna Acharya, Swetha Acharya, Tajudeen Adesanmi Adebisi, Kamoru Ademola Adedokun, Muhammad Sohail Afzal, Piyush Agrawal, Sajjad Ahmad,

Suhaib Ahmad, Tauseef Ahmad, Ali Ahmadi, Ali Ahmed, Ayman Ahmed, Naveed Ahmed, Shakeel Ahmed, Kenni Wojujutari Ajele, Chisom Joyqueenet Akunna, Amer Hashim Al Ani, Salah Al Awaidy, Montaha Al-Iede, Sabah Al-Marwani, Essam Ahmed Al-Moraissi, Ali Salman Al-Shami, Mostafa Alam, Mohammed Albashtawy, Abdelazeem M Algammal, Saeed M. M Alghamdi, Amidu Alhassan, Ashraf Alhumaidi, Syed Shujait Ali, Morteza Alipour, Mohmmad Minwer Alnaeem, Khaldoon Aied Alnawafleh, Margret Beaula Alocious Sukumar, Zaid Altaany, Awais Altaf, Hany Aly, Priscilla O Ameh, Junaid Amin, Saeed Amini, Hubert Amu, A.h. Sruthi Anil Kumar, Kabilan Annadurai, Saeid Anvari, Jalal Arabloo, Jorge Arias De La Torre, Mohammad Saquib Ashraf, Tahira Ashraf, Nyan Min Aung, Manal Awad, Sana Javaid Awan, Usman Ayub Awan, Domenico Azzolino, Huma Bader Ul Ain, Riadh Badraoui, Khlood K Baghlaf, Atif Amin Baig, Marina Balabekova, Raju Balaji, Yatan Pal Singh Balhara, Maciej Banach, Morteza Banakar, Salam Bani Hani, Palash Chandra Banik, Farhat Bano, Amadou Barrow, Shahid Bashir, Mohammad-Mahdi Bastan, Hagar Abd El Naby Bastawy, Oyundari Batsaikhan, Babak Behnam, Nahom Girma Belete, Uzma Iqbal Belgaumi, Ghaith Benhsen, Sonu Bhaskar, Souad Bouaoud, Christopher Boxe, Dejana Braithwaite, Michael Eric Bryan, Sayed Muhammad Ata Ullah Shah Bukhari, Sandra Carvalho, Vijay Kumar Chattu, Sirshendu Chaudhuri, Akhilanand Chaurasia, Junhao Chen, Meng Xuan Chen, Mirza Farhana Iqbal Chowdhury, Dinh-Toi Chu, Natalia Cruz-Martins, Neha Dahiya, Samuel Demissie Darcho, Riya Dave, Premnath Dhanaraj, Elangovan Dilipan, Sadia Din, Orlando Luiz Do Amaral Júnior, Ojas Prakashbhai Doshi, Taiwo Omotayo Dosumu, Jiang Du, Vikash Kumar Dubey, Aziz Eftekharimehrabad, Ebrahim Eini, Michael Ekholuenetale, Iman El Sayed, Ashraf A El-Metwally, Abdelgawad Salah Eltahawy, Purushoth Ethiraj, Devaraj Ezhilarasan, Adeniyi Francis Fagbamigbe, Ayesha Fahim, Nour Fakih, Muhammad Amber Fareed, Anees Ahmed Farooq Mohammed, Fatemeh Farshad, Alireza Feizkhah, Tommaso Filippini, Carlos Forner Álvarez, Takeshi Fukumoto, Peter Andras Gaal, Piyada Gaewkhiew, Silvano Gallus, Fernando Jr. Barroga Garcia, Tsion S Gebre, Nsikakabasi Samuel George, Ahmad Ghashghaee, Sama Ghoba, Elena Ghotbi, Thinh Gia Gia Nguyen, Anil Kumar Goel, Awais Gohar, Pouya Goleij, Mohsen Golkar, Divya Gopinath, Bhuvaneshwari Gunasekar, Bhawna Gupta, Sapna Gupta, Gamze Gökulu, Khalid Rehman Hakeem, Pritam Halder, Samer Hammoudeh, Faizul Hasan, Towhid Hasan, Ahmad Shoeb Hashmi, Simon I Hay, Fei He, Golnaz Heidari, Mohammad Heidari, Nguyen Quoc Hoan, Matthew Hobbs, Mehdi Hosseinzadeh, Weijun Huang, Liliya Ibragimova, Khalid S Ibrahim, Jibran Ikram, Olayinka Stephen Ilesanmi, Gaetano Isola, Haitham Jahrami, Mihajlo Jakovljevic, Sahbanathul Missiriya Jalal, Rajiv Janardhanan, Sathish Kumar Jayapal, Krupal Joshi, Farahnaz Joukar, Easter Joury, Jacek Jerzy Jozwiak, Hema Shree K, Zubair Kabir, Vidya Kadashetti, Adel Kadri, Pradnya Vishal Kakodkar, Kamala Kannan, Rami S Kantar, Neila Karagezova, Mohmed Isaqali Karobari, Tomasz M Karpiński, Gebrehiwot G Kassa, Stella Adejoke Kayode, Yousef Saleh Khader, Himanshu Khajuria, Muhammad Khan, Muhammad Umer Khan, Raees Khan, Zahid Khan, Sameer Uttamaro Khasbage, Khalid A Kheirallah, Feriha Fatima Khidri, Sanjay Kini B, Adnan Kisa, Shivakumar KM, Kewal Krishan, Omar Kujan, Dewesh Kumar, Jeevan Kumar, Jogender Kumar, Kamal Kumar, Pradeep Kumar, Sandeep Kumar, Dian Kusuma, Giusy Rita Maria La Rosa, Dharmesh Kumar Lal, Savita Lasrado, Seung Won Lee, Kakithakara Vajravelu Leela, Mengyi LI, Zhengrui Li, Erand Llanaj, Ellina Lytvyak, Kevin Sheng-Kai Ma, Siyao Ma, Monika Machoy-Rakoczy, Máté Mackei, Inamul Hasan Madar, Mohammed Magdy Abd El Razek, Nozad Hussein Mahmood, Iqra Manzoor, Tahir Maqbool, Vijaya Marakala, Wagner Marcenes, Priyadharshini Marckasagayam, Stefano Masi, Manu Raj Mathur, Medha Mathur, Jacek Matys, Jitendra Kumar Meena, Riffat Mehboob, Vini Mehta, Satish Melwani, Ritesh G Menezes, Andrea Michelerio, Giuseppe Minervini, Tridip Mitra, Kanisha Mittal, Nouh Saad Mohamed, Yahaya Mohammed, Yugal Kishore Mohanta, Ali H Mokdad, Mahdis Morovvati, Rohith Motappa, Amin Mousavi Khaneghah, Ahmed Msherghi, Rabia Mubarak, Sumaira Mubarik, Christopher J L Murray, David Musoke, Saravanan Muthupandian, Ganesh R Naik, Prasad Nalabothu, Rizwan Naqvi, Shumaila Nargus, Zuhair S Natto, Zakira Naureen, Biswa Prakash Nayak, Kinjal Nayak, Javad Nazari,

Sudan Prasad Neupane, Sumaiya Nezam, Cuong Tat Nguyen, Duy Cao Nguyen, Long Hoang Nguyen, Tham Thi Nguyen, Lawrence Achilles Nnyanzi, Bogdan Oancea, Musa Kolapo Oladejo, Yinka Doris Oluwafemi, Mahesh P A, Jagadish Rao Padubidri, Sujogya Kumar Panda, Keerthi Panneer Selvam, Shahina Pardhan, Romil R Parikh, Jay Patel, Mehali Lalitbhai Patel, Mitesh Patel, Shivani Patel, Shankargouda Patil, Shrikant Pawar, Pawel Piotr Pietkiewicz, Thantrira Porntavetus, Amit Porwal, Priyanka Porwal, Bharathi M Purohit, Nameer Hashim Qasim, Asma Saleem Qazi, Xiang Qi, Nuzul Qur'aniati, Venkatraman Radhakrishnan, Amina Suleiman Rajah, Rafał Rakoczy, Shakthi Kumaran Ramasamy, Lokesh Rana, Kumuda Rao, Mithun Rao, Sowmya J Rao, Shree Rath, Tapash Ranjan Rautray, Sujith Sri Surya Ravi, Salman Rawaf, Muhammad Liaquat Raza, Elrashdy M Redwan, Bhagwan Narayan Rekadwad, Hermano Alexandre Lima Rocha, Chanapong Rojanaworarit, Mario Romandini, Khaled Saad, Korosh Saber, Umar Saeed, Fatemeh Saheb Sharif-Askari, Vaibhav Sahni, Alaka Sahoo, Ambika Sahoo, Joseph W Sakshaug, Samreen Saleem, Fasineh Shekuba Samura, Abdallah M Samy, Sathish Sankar, Andari Sarasati, Maheswar Satpathy, Amar Saurabh, Siddharthan Selvaraj, Subramanian Senthilkumaran, Muhammad Shahzad, Masood Ali Shaikh, Muhammad Aaqib Shamim, Mohammed Shannawaz, Nadim Sharif, Amin Sharifan, Kamlesh Sharma, Mahabalesh Shetty, Premalatha K Shetty, Shirindokht Shirazi, Km Shivangi, Deborah Oluwaseun Shomuyiwa, Sunil Shrestha, Suleiman Adeiza Shuaibu, Luís Manuel Lopes Rodrigues Silva, Abhinav Singh, Amit Singh, Harpreet Singh, Jasvinder A Singh, Samer Singh, Siddharth Singh, Gisha Sivan, Gowri Sivaramakrishnan, Farrukh Sobia, Monica Charlotte Solomon, Quirin D Strotzer, Mahwish Suhaib, Marjia Sultana, Seyyed Mohammad Tabatabaei, Amir Tahavvori, Jabeen Taiba, Mircea Tampa, Dufera Rikitu Terefa, Manuel Sebastian Thomas, Amir Tiyuri, Madi Tleshev, Arole Darwin Touko, Marcos Roberto Tovani-Palone, Indang Trihandini, Srikanth Umakanthan, Bhaskaran Unnikrishnan, Amedin Aliyi Usso, Olivier Uwishema, Vishnu Priya Veeraraghavan, Nguyen-Kieu Viet-Nhi, Simona Ruxandra Volovat, Liang Wang, Wanzhou Wang, Chenxuan Wei, Desalegn Desalegn Weldebrhan, Andrew Awuah Wireko, Kivanc Yangi, Thomas Kidanemariam Yewodiaw, Siyan Yi, Vesna Zadnik, Mubashir Zafar, Iman Zare, Jianhui Zhao, Magdalena Zielińska, Shaher H Zyoud

Developing methods or computational machinery

Aleksandr Y Aravkin, Simon I Hay, Ali H Mokdad, Christopher J L Murray

Providing critical feedback on methods or results

Abdullah ., Hazim S Ababneh, Belayneh Jejaw Abate, Hedayat Abbastabar, Sherief Abd-Elsalam, Wael M Abdel-Rahman, Hassan A. Abdou, Meriem Abdoun, Rizwan Suliankatchi Abdulkader, Syed Hani Abidi, Richard Gyan Aboagye, Lucas Guimarães Abreu, Sawsan Abuhammad, Anirudh Balakrishna Acharya, Swetha Acharya, Lawan Hassan Adamu, Tajudeen Adesanmi Adebisi, Babatope Oluwadamilare Adebiyi, Kamoru Ademola Adedokun, Aanuoluwapo Adeyimika Afolabi, Muhammad Sohail Afzal, Percival Delali Agordoh, Salome Wumpini Agordoh, Piyush Agrawal, Aqeel Ahmad, Khabir Ahmad, Sajjad Ahmad, Suhaib Ahmad, Tauseef Ahmad, Ali Ahmadi, Ali Ahmed, Ayman Ahmed, Naveed Ahmed, Shakeel Ahmed, Kenni Wojujutari Ajele, Kasuni Hm Akalanka, Hammad Akram, Chisom Joyqueenet Akunna, Amer Hashim Al Ani, Salah Al Awaidy, Yazan Al-Ajlouni, Mohammed Sultan Al-Ak'hali, Montaha Al-Iede, Sabah Al-Marwani, Sadeq A Al-Maweri, Essam Ahmed Al-Moraissi, Ali Salman Al-Shami, Timothy Olukunle Aladelusi, Mohammad Khursheed Alam, Mostafa Alam, Ruaa A Alamoudi, Mohammed Albashtawy, Abdelazeem M Algammal, Saeed M. M Alghamdi, Mohammed Ahmed Alghauli, Amidu Alhassan, Ashraf Alhumaidi, Kamran Ali, Syed Shujait Ali, Taha Hussein Hussein Ali, Morteza Alipour, Mohmmad Minwer Alnaeem, Khaldoon Aied Alnawafleh, Margret Beaula Alocious Sukumar, Mohammad Alqudah, Mohammad R Alqudimat, Ahmed Yaseen Alqutaibi, Mustafa Haleem Alsaadi, Mohammed

Amjed Alsaegh, Anas Alsalhani, Zaid Altaany, Awais Altaf, Hany Aly, Karem H Alzoubi, Nada A Alzunaidy, Uchenna Anderson Amaechi, Mcking Izeiza Amedari, Priscilla O Ameh, Junaid Amin, Saeed Amini, Hubert Amu, A.h. Sruthi Anil Kumar, Kabilan Annadurai, Saeid Anvari, Anayochukwu Edward Anyasodor, Jalal Arabloo, Mosab Arafat, Gururaj Arakeri, Demelash Areda, Jorge Arias De La Torre, Javed Ashraf, Mohammad Saquib Ashraf, Tahira Ashraf, Esma Asil, Saira Atif, Samuel Babajide Atoyebi, Nyan Min Aung, Manal Awad, Sana Javaid Awan, Usman Ayub Awan, Ashish Awasthi, Aneesa Ayoob, Somayyeh Azimi, Domenico Azzolino, Muhammad Badar, Huma Bader Ul Ain, Ashish D Badiye, Riadh Badraoui, Khlood K Baghlaf, Dania Bahdila, Atif Amin Baig, Mohammad Hassan Baig, Mohamad Amin Bakhshali, Raju Balaji, Wondu Feyisa Balcha, Abdulrahman A. A Balhaddad, Yatan Pal Singh Balhara, Maciej Banach, Morteza Banakar, Salam Bani Hani, Palash Chandra Banik, Farhat Bano, Md. Abul Barkat, Amadou Barrow, Shahid Bashir, Mohammad-Mahdi Bastan, Hagar Abd El Naby Bastawy, Kavita Batra, Oyundari Batsaikhan, Babak Behnam, Almaz Nibret Belay, Enku Shiferaw Belayneh, Nahom Girma Belete, Uzma Iqbal Belgaumi, Ibrahim Olajide Bello, Ghaith Benhsen, Alemshet Yirga Berhie, Ghalia Y Bhadila, Pooja Bhadoria, Pankaj Bhardwaj, Sonu Bhaskar, Ajay Nagesh Bhat, Nada Binmadi, Trupti Bodhare, Souad Bouaoud, Abdelhakim Bouyahya, Christopher Boxe, Maria Laura Braccini Fagundes, Dejana Braithwaite, Michael Eric Bryan, Lin-Lin Bu, Sayed Muhammad Ata Ullah Shah Bukhari, Sanjay C J, Yu Cao, Sandra Carvalho, Pamela Roxana Chacón-Uscamaita, Vijay Kumar Chattu, Sirshendu Chaudhuri, Akhilanand Chaurasia, Junhao Chen, Meng Xuan Chen, Mirza Farhana Iqbal Chowdhury, Dinh-Toi Chu, Natalia Cruz-Martins, Ferran Cuenca-Martínez, Omid Dadras, Namrata Dagli, Lina Dahabiyeh, Neha Dahiya, Samuel Demissie Darcho, Riya Dave, Mauro Henrique Nogueira Guimarães De Abreu, Emina Dervišević, Premnath Dhanaraj, Amol S Dhane, Antonio Di Guardo, Elangovan Dilipan, Sadia Din, Orlando Luiz Do Amaral Júnior, Lingkang Dong, Ojas Prakashbhai Doshi, Taiwo Omotayo Dosumu, Jiang Du, Mi Du, Manisha Dubey, Vikash Kumar Dubey, Siddhartha Dutta, Arkadiusz Marian Dziedzic, Venkata Ravikanth Eddula, Aziz Eftekharimehrabad, Ebrahim Eini, Michael Ekholuenetale, Seraphine Mojoko Eko, Abdelfatteh EL Omri, Iman El Sayed, Ashraf A El-Metwally, Abdelbaset Mohamed Elasbali, Amine Elbouzidi, Mohamed Elhoumed, Omar Abdelsadek Abdou Elmeligy, Rami Elmorsi, Nadia Bassuoni Elsharkawy, Abdelgawad Salah Eltahawy, Heba Emad, Purushoth Ethiraj, Devaraj Ezhilarasan, Amanda Ugochi Ezirim, Adeniyi Francis Fagbamigbe, Ayesha Fahim, Nour Fakih, Muhammad Amber Fareed, Mohsen A Farghaly, Anees Ahmed Farooq Mohammed, Fatemeh Farshad, Alireza Feizkhah, Guangshou Feng, Maria Pia Ferraz, Getahun Fetensa, Tommaso Filippini, Florian Fischer, James L Fisher, Carlos Forner Álvarez, Delfin Lovelina Francis, Takeshi Fukumoto, Peter Andras Gaal, Amol Ramchandra Gadbail, Piyada Gaewkhiew, Márió Gajdács, Fernando Jr. Barroga Garcia, Tsion S Gebre, Nsikakabasi Samuel George, Ahmad Ghashghaee, Elena Ghotbi, Thinh Gia Gia Nguyen, Paramjit Singh Gill, Anil Kumar Goel, Awais Gohar, Mohsen Golkar, Shailesh Madhukar Gondivkar, Divya Gopinath, Barbara Niegia Garcia Goulart, Changhao Gu, Bhuvaneshwari Gunasekar, Bhawna Gupta, Sapna Gupta, Sunanda Gupta, Swati Gupta, Bruno Gutiérrez, Gamze Gökulu, Khalid Rehman Hakeem, Esam Halboub, Pritam Halder, Raz Mohammed Hama Salih, Ibrahim Ismael Hamarash, Mohamed Hamed, Samer Hammoudeh, Abdelrahman M Hamouda, Mohd Anul Haq, Faizul Hasan, Towhid Hasan, Hamidreza Hasani, Ahmad Shoeb Hashmi, Md Saquib Hasnain, Simon I Hay, Fei He, Golnaz Heidari, Mohammad Heidari, Narmin Mohammedsaeed Helal, Brenda Yuliana Herrera-Serna, Kamran Heydaryan, Kamal Hezam, Nguyen Quoc Hoan, Matthew Hobbs, Ramesh Holla, Syed Billal Hossain, Mehdi Hosseinzadeh, Sorin Hostiuc, Weijun Huang, Fernando N Hugo, Bing-Fang Hwang, Liliya Ibragimova, Khalid S Ibrahim, Jibran Ikram, Olayinka Stephen Ilesanmi, Irena M Ilic, Milena D Ilic, Danish Iqbal, Md Rabiul Islam, Gaetano Isola, Hiroshi Ito, Chidozie Declan Iwu, Haitham Jahrami, Ayushi Jain, Ammar Abdulrahman Jairoun, Mihajlo Jakovljevic, Sahbanathul Missiriya Jalal, Reza Jalilzadeh Yengejeh, Rajiv Janardhanan, Sathish Kumar Jayapal, Ruwan Duminda Jayasinghe, Wonjeong Jeong, Nathan T Jibat, Nitin Joseph, Krupal Joshi, Farahnaz Joukar, Jacek Jerzy Jozwiak, Hema

Shree K, Thenmozhi K, Zubair Kabir, Vidya Kadashetti, Adel Kadri, Pradnya Vishal Kakodkar, Eswar Kandaswamy, Kehinde Kazeem Kanmodi, Kamala Kannan, Rami S Kantar, Neila Karagezova, Indira Karibayeva, Mohmed Isaqali Karobari, Tomasz M Karpiński, Gebrehiwot G Kassa, Birhanu Kassie Abeje, Joonas H Kauppila, Stella Adejoke Kayode, Awoke Amare Kebede, Yousef Saleh Khader, Himanshu Khajuria, Hafiz Nadeem Khan, Muhammad Khan, Muhammad Umer Khan, Raees Khan, Zahid Khan, Sameer Uttamaro Khasbage, Khalid A Kheirallah, Feriha Fatima Khidri, Sung Min Kim, Yihyun Kim, Ruth W Kimokoti, Sanjay Kini B, Adnan Kisa, Shivakumar KM, Prakash Babu Kodali, Farzad Kompani, Archana Koul, Kewal Krishan, Bindu Krishnan, Nitya Krishnasamy, Omar Kujan, Anit Kujur, Emmanuel Kumah, Ashok Kumar, Dewesh Kumar, Jeevan Kumar, Jogender Kumar, Kamal Kumar, Manoj Kumar, Neeraj Kumar, Pawan Kumar, Pradeep Kumar, Sandeep Kumar, Sumeet Kumar, Jibin Kunjavara, Om P Kurmi, Pramod Kumar Kushawaha, Dian Kusuma, Giusy Rita Maria La Rosa, Timo Lajunen, Dharmesh Kumar Lal, Ratilal Lalloo, Tamás Lantos, Savita Lasrado, Seung Won Lee, Kakithakara Vajravelu Leela, Elvynna Leong, Mengyi LI, Ming-Chieh Li, Zhengrui Li, Ambreen Liaqat, Liu Liu, Erand Llanaj, Madeeha Shahzad Lodhi, Mariia Lopatkina, Shanjie Luan, Ellina Lytvyak, Anna Lövgren, Hawraz Ibrahim M. Amin, Kevin Sheng-Kai Ma, Siyao Ma, Monika Machoy-Rakoczy, Máté Mackei, Inamul Hasan Madar, Ahmed A Madfa, Mohammed Magdy Abd El Razek, Nozad Hussein Mahmood, Trisha Mallick, Iqra Manzoor, Tahir Maqbool, Vijaya Marakala, Wagner Marcenes, Priyadharshini Marckasagayam, Stefano Masi, Manu Raj Mathur, Medha Mathur, Khurshid A Mattoo, Jacek Matys, Jitendra Kumar Meena, Riffat Mehboob, Vini Mehta, Dalia E Meisha, Satish Melwani, Ritesh G Menezes, Tomislav Mestrovic, Andrea Michelerio, Giuseppe Minervini, Tridip Mitra, Kanisha Mittal, Hashem Mohamadian, Fatima Awad Elkarim Elfaki Mohamed, Nouh Saad Mohamed, Yahaya Mohammed, Yugal Kishore Mohanta, Ali H Mokdad, Himel Mondal, Paula Moraga, Rafael Silveira Moreira, Mahdis Morovvati, Rohith Motappa, Amin Mousavi Khaneghah, Ahmed Msherghi, Rabia Mubarak, Sumaira Mubarik, Christopher J L Murray, Satta Muthu Murugan, David Musoke, Sherzad Ibrahim Mustafa, Saravanan Muthupandian, Kiran P. P Nagdeo, Ganesh R Naik, Lekshmi M Nair, Prasad Nalabothu, Rizwan Naqvi, Shumaila Nargus, Gustavo G Nascimento, Zuhair S Natto, Zakira Naureen, Biswa Prakash Nayak, Kinjal Nayak, Javad Nazari, Sudan Prasad Neupane, Sumaiya Nezam, Cuong Tat Nguyen, Duy Cao Nguyen, Long Hoang Nguyen, Tham Thi Nguyen, Vikram Niranjan, Lawrence Achilles Nnyanzi, Mohammed Noushad, Nazik Nurelhuda, Bogdan Oancea, Musa Kolapo Oladejo, Yinka Doris Oluwafemi, Esteban Ortiz-Prado, Mahesh P A, Jagadish Rao Padubidri, Sujogya Kumar Panda, Keerthi Panneer Selvam, Georgios D Panos, Shahina Pardhan, Romil R Parikh, Pragnesh Parmar, Sameena Parveen, Eleonora Pascucci, Jay Patel, Mehali Lalitbhai Patel, Mitesh Patel, Shivani Patel, Vipin Patidar, Shankargouda Patil, Shrikant Pawar, Olumuyiwa James Peter, Pawel Piotr Pietkiewicz, Thantrira Porntavetus, Amit Porwal, Priyanka Porwal, Sergio I Prada, Harsh Priya, Luís Proença, Pooja Puri, Bharathi M Purohit, Nameer Hashim Qasim, Asma Saleem Qazi, Xiang Qi, Cheng Quan, Nuzul Qur'aniati, Raghu Anekal Radhakrishnan, Venkatraman Radhakrishnan, Fakher Rahim, Mosiur Rahman, Hakim Rahmoune, Amina Suleiman Rajah, Rafał Rakoczy, Venkitachalam Ramanarayanan, Ramasubburayan Ramasamy, Shakthi Kumaran Ramasamy, Lokesh Rana, Shailendra Singh Rana, Kumuda Rao, Mithun Rao, Sowmya J Rao, Prateek Rastogi, Shree Rath, Devarajan Rathish, Tapash Ranjan Rautray, Sujith Sri Surya Ravi, Salman Rawaf, Muhammad Liaquat Raza, Elrashdy M Redwan, Abdul Rehman, Bhagwan Narayan Rekadwad, Nazila Rezaei, Abanoub Riad, Maria Jesus Rios-Blancas, Hermano Alexandre Lima Rocha, Chanapong Rojanaworarit, Mario Romandini, Rohini Ruhil, Khaled Saad, Korosh Saber, Fatemeh Sadeghi-Ghyassi, Mohammad Reza Saeb, Umar Saeed, Fatemeh Saheb Sharif-Askari, Vaibhav Sahni, Alaka Sahoo, Ambika Sahoo, Durgesh Prasad Sahoo, Ankita Saikia, Joseph W Sakshaug, Samreen Saleem, Firdaus Samad, Fasineh Shekuba Samura, Abdallah M Samy, Sathish Sankar, Andari Sarasati, Gargi Sachin Sarode, Sachin C Sarode, Michele Sassano, Aanchal Satija, Maheswar Satpathy, Jennifer Saulam, Amar Saurabh, Falk Schwendicke, Amin Sedigh, Siddharthan Selvaraj,

Subramanian Senthilkumaran, Mostafa Shaban, Arsalan Shahri, Hamid R Shahsavari, Muhammad Shahzad, Masood Ali Shaikh, Muhammad Aaqib Shamim, Alfiya Shamsutdinova, Mohammed Shannawaz, Nadim Sharif, Amin Sharifan, Kamlesh Sharma, Bereket Beyene Shashamo, Mahabalesh Shetty, Shirindokht Shirazi, Km Shivangi, Zahra Shokati Eshkiki, Deborah Oluwaseun Shomuyiwa, Seyed Afshin Shorofi, Sunil Shrestha, Suleiman Adeiza Shuaibu, Gustavo Correia Basto Da Silva, Luís Manuel Lopes Rodrigues Silva, Abhinav Singh, Amit Singh, Harpreet Singh, Jasvinder A Singh, Prince Kumar Singh, Ragini D Singh, Samer Singh, Siddharth Singh, Richa Singhania, Gisha Sivan, Gowri Sivaramakrishnan, Farrukh Sobia, Saraswathy Sreeram, Kannan Sridharan, Quirin D Strotzer, Mahwish Suhaib, Marjia Sultana, Jing Sun, Seyyed Mohammad Tabatabaei, Biniyam Beyene Tabor, Jyothi Tadakamadla, Santosh Kumar Tadakamadla, Amir Tahavvori, Jabeen Taiba, Iman M Talaat, Mircea Tampa, Benjamin Kye Jyn Tan, Shynar Tanabayeva, Abdelghani Tbakhi, Dufera Rikitu Terefa, Tigist Derebe Tesfahun, Azimeraw Arega Tesfu, Ismaeel Tharwat, Manuel Sebastian Thomas, Amir Tiyuri, Madi Tleshev, Razie Toghroli, Arole Darwin Touko, Marcos Roberto Tovani-Palone, Indang Trihandini, Mike Tuffour Amirikah, Biruk Shalmeno Tusa, Saeed Ullah, Srikanth Umakanthan, Bhaskaran Unnikrishnan, Amedin Aliyi Usso, Olivier Uwishema, Hande Uzunçıbuk, Orsolya Varga, Siddhartha Alluri Varma, Vishnu Priya Veeraraghavan, Prabhakar Veginadu, U Venkatesh, Nguyen-Kieu Viet-Nhi, Vignesh Vikram, Victor E Villalobos-Daniel, Ave Voit, Simona Ruxandra Volovat, Liang Wang, Wanzhou Wang, Zhikang Wang, Tati Suryati Warouw, Chenxuan Wei, Desalegn Desalegn Weldebrhan, Nuwan Darshana Wickramasinghe, Andrew Awuah Wireko, Shulan Wu, Patlolla Sriram Yadav, Saba Yahoo, Natsuhiko Yamada, Kivanc Yangi, Thomas Kidanemariam Yewodiaw, Siyan Yi, Dong Keon Yon, Monal Yuwanati, Vesna Zadnik, Mubashir Zafar, Muhammad Sohail Zafar, Mohammed G M Zeariya, Jianhui Zhao, Lei Zheng, Zahraa Zibara, Magdalena Zielińska, Shaher H Zyoud

Drafting the work or revising it critically for important intellectual content

Abdullah ., Belayneh Jejaw Abate, Hedayat Abbastabar, Sherief Abd-Elsalam, Wael M Abdel-Rahman, Hassan A. Abdou, Lucas Guimarães Abreu, Sawsan Abuhammad, Anirudh Balakrishna Acharya, Swetha Acharya, Lawan Hassan Adamu, Tajudeen Adesanmi Adebisi, Babatope Oluwadamilare Adebiyi, Kamoru Ademola Adedokun, Aanuoluwapo Adeyimika Afolabi, Muhammad Sohail Afzal, Percival Delali Agordoh, Salome Wumpini Agordoh, Piyush Agrawal, Trushdeep Agrawal, Khabir Ahmad, Suhaib Ahmad, Tauseef Ahmad, Ali Ahmadi, Ali Ahmed, Ayman Ahmed, Naveed Ahmed, Shakeel Ahmed, Kenni Wojujutari Ajele, Kasuni Hm Akalanka, Sara Akeel, Damilare Michael Akintunde, Hammad Akram, Amer Hashim Al Ani, Salah Al Awaidy, Yazan Al-Ajlouni, Mohammed Sultan Al-Ak'hali, Montaha Al-Iede, Sadeq A Al-Maweri, Essam Ahmed Al-Moraissi, Ali Salman Al-Shami, Timothy Olukunle Aladelusi, Mostafa Alam, Ruaa A Alamoudi, Mohammed Albashtawy, Mohammed S Aldossary, Abdelazeem M Algammal, Saeed M. M Alghamdi, Amidu Alhassan, Ashraf Alhumaidi, Kamran Ali, Syed Shujait Ali, Taha Hussein Hussein Ali, Morteza Alipour, Behrouz Alizadeh Savareh, Mohmmad Minwer Alnaeem, Khaldoon Aied Alnawafleh, Margret Beaula Alocious Sukumar, Nivaldo Alonso, Mohammad Alqudah, Mohammad R Alqudimat, Mustafa Haleem Alsaadi, Mohammed Amjed Alsaegh, Anas Alsalhani, Awais Altaf, Hany Aly, Karem H Alzoubi, Nada A Alzunaidy, Mcking Izeiza Amedari, Priscilla O Ameh, Junaid Amin, Saeed Amini, Mohammad Hosein Amirzade-Iranaq, Hubert Amu, Abhishek Anil, Kabilan Annadurai, Saeid Anvari, Anayochukwu Edward Anyasodor, Jalal Arabloo, Jorge Arias De La Torre, Javed Ashraf, Mohammad Saquib Ashraf, Esma Asil, Saira Atif, Manal Awad, Usman Ayub Awan, Ashish Awasthi, Aneesa Ayoob, Domenico Azzolino, Muhammad Badar, Huma Bader Ul Ain, Ashish D Badiye, Riadh Badraoui, Dania Bahdila, Atif Amin Baig, Abdulrahman A. A Balhaddad, Yatan Pal Singh Balhara, Maciej Banach, Morteza Banakar, Salam Bani Hani, Palash Chandra Banik, Farhat Bano, Md. Abul Barkat, Rocío Barrios-Rodríguez, Amadou Barrow, Shahid Bashir, Azadeh Bashiri, Mohammad-Mahdi Bastan, Hagar Abd El Naby

Bastawy, Oyundari Batsaikhan, Babak Behnam, Nahom Girma Belete, Uzma Iqbal Belgaumi, Ibrahim Olajide Bello, Eduardo Bernabe, Ghalia Y Bhadila, Pooja Bhadoria, Sonu Bhaskar, Ajay Nagesh Bhat, Ashok Kumar Bhati, Nada Binmadi, Tone Bjørge, Srinivasa Rao Bolla, Souad Bouaoud, Abdelhakim Bouyahya, Christopher Boxe, Maria Laura Braccini Fagundes, Dejana Braithwaite, Nikolay Ivanovich Briko, Michael Eric Bryan, Sayed Muhammad Ata Ullah Shah Bukhari, Sanjay C J, Giulia Carreras, Andrea Carugno, Felix Carvalho, Sandra Carvalho, Pamela Roxana Chacón-Uscamaita, Sandip Chakraborty, Vijay Kumar Chattu, Anis Ahmad Chaudhary, Sirshendu Chaudhuri, Akhilanand Chaurasia, Junhao Chen, Meng Xuan Chen, Dinh-Toi Chu, Natalia Cruz-Martins, Ferran Cuenca-Martínez, Namrata Dagli, Lina Dahabiyeh, Neha Dahiya, Mouni Damineni, Samuel Demissie Darcho, Mauro Henrique Nogueira Guimarães De Abreu, Emina Dervišević, Premnath Dhanaraj, Amol S Dhane, Antonio Di Guardo, Sadia Din, Orlando Luiz Do Amaral Júnior, Sushil Dohare, Cátia Domingues, Francesco Dondi, Lingkang Dong, Ojas Prakashbhai Doshi, Taiwo Omotayo Dosumu, Jiang Du, Mi Du, Manisha Dubey, Vikash Kumar Dubey, Siddhartha Dutta, Arkadiusz Marian Dziedzic, Venkata Ravikanth Eddula, Aziz Eftekharimehrabad, Ebrahim Eini, Michael Ekholuenetale, Abdelfatteh EL Omri, Iman El Sayed, Maha El Tantawi, Ashraf A El-Metwally, Amine Elbouzidi, Mohamed Elhoumed, Omar Abdelsadek Abdou Elmeligy, Rami Elmorsi, Abdelgawad Salah Eltahawy, Heba Emad, Purushoth Ethiraj, Amanda Ugochi Ezirim, Adeniyi Francis Fagbamigbe, Ayesha Fahim, Nour Fakih, Ebrahim Farashi, Muhammad Amber Fareed, Mohsen A Farghaly, Anees Ahmed Farooq Mohammed, Fatemeh Farshad, Maria Pia Ferraz, Nuno Ferreira, Getahun Fetensa, Tommaso Filippini, Florian Fischer, James L Fisher, Carlos Forner Álvarez, Delfin Lovelina Francis, Takeshi Fukumoto, Peter Andras Gaal, Amol Ramchandra Gadbail, Piyada Gaewkhiew, Márió Gajdács, Silvano Gallus, Tsion S Gebre, Nsikakabasi Samuel George, Nasim Gholizadeh, Mahsa Ghorbani, Elena Ghotbi, Thinh Gia Gia Nguyen, Paramjit Singh Gill, Anil Kumar Goel, Marília Goettems, Awais Gohar, Mohsen Golkar, Shailesh Madhukar Gondivkar, Divya Gopinath, Giuseppe Gorini, Barbara Niegia Garcia Goulart, Changhao Gu, Giovanni Guarducci, Bhuvaneshwari Gunasekar, Bhawna Gupta, Sapna Gupta, Sunanda Gupta, Swati Gupta, Bruno Gutiérrez, Gamze Gökulu, Heliasadat Haeriboroojeni, Khalid Rehman Hakeem, Esam Halboub, Raz Mohammed Hama Salih, Ibrahim Ismael Hamarash, Mohamed Hamed, Samer Hammoudeh, Abdelrahman M Hamouda, Mohd Anul Haq, Faizul Hasan, Shamimul Hasan, Towhid Hasan, Hamidreza Hasani, Nada Tawfig Hashim, Ahmad Shoeb Hashmi, Md Saquib Hasnain, Simon I Hay, Fei He, Golnaz Heidari, Narmin Mohammedsaeed Helal, Kamran Heydaryan, Kamal Hezam, Nguyen Quoc Hoan, Matthew Hobbs, Ramesh Holla, Syed Billal Hossain, Mehdi Hosseinzadeh, Sorin Hostiuc, Weijun Huang, Fernando N Hugo, Liliya Ibragimova, Khalid S Ibrahim, Jibran Ikram, Olayinka Stephen Ilesanmi, Irena M Ilic, Milena D Ilic, Bilal Irfan, Md Rabiul Islam, Gaetano Isola, Hiroshi Ito, Chidozie Declan Iwu, Haitham Jahrami, Mihajlo Jakovljevic, Sahbanathul Missiriya Jalal, Rajiv Janardhanan, Sathish Kumar Jayapal, Ruwan Duminda Jayasinghe, Nitin Joseph, Farahnaz Joukar, Easter Joury, Jacek Jerzy Jozwiak, Hema Shree K, Vidya Kadashetti, Adel Kadri, Pradnya Vishal Kakodkar, Kehinde Kazeem Kanmodi, Rami S Kantar, Indira Karibayeva, Mohmed Isaqali Karobari, Tomasz M Karpiński, Gebrehiwot G Kassa, Joonas H Kauppila, Yousef Saleh Khader, Himanshu Khajuria, Hafiz Nadeem Khan, Muhammad Khan, Muhammad Umer Khan, Zahid Khan, Sameer Uttamaro Khasbage, Khalid A Kheirallah, Feriha Fatima Khidri, Min Seo Kim, Sung Min Kim, Yihyun Kim, Sanjay Kini B, Adnan Kisa, Tobias Kleinjung, Shivakumar KM, Prakash Babu Kodali, Kewal Krishan, Bindu Krishnan, Mukhtar Kulimbet, Dewesh Kumar, Jeevan Kumar, Jogender Kumar, Kamal Kumar, Neeraj Kumar, Pawan Kumar, Pradeep Kumar, Sandeep Kumar, Om P Kurmi, Dian Kusuma, Giusy Rita Maria La Rosa, Tamás Lantos, Savita Lasrado, Kakithakara Vajravelu Leela, Elvynna Leong, Zhengrui Li, Liu Liu, Erand Llanaj, Madeeha Shahzad Lodhi, Alessandra Lugo, Ellina Lytvyak, Anna Lövgren, Kevin Sheng-Kai Ma, Siyao Ma, Monika Machoy-Rakoczy, Máté Mackei, Inamul Hasan Madar, Mohammed Magdy Abd El Razek, Mari Kannan Maharajan, Nozad Hussein Mahmood, Trisha Mallick, Iqra Manzoor, Vijaya Marakala, Wagner Marcenes, Priyadharshini Marckasagayam, Stefano Masi, Medha Mathur,

Khurshid A Mattoo, Jacek Matys, Jitendra Kumar Meena, Vini Mehta, Dalia E Meisha, Ritesh G Menezes, Tomislav Mestrovic, Andrea Michelerio, Giuseppe Minervini, Tridip Mitra, Kanisha Mittal, Hashem Mohamadian, Fatima Awad Elkarim Elfaki Mohamed, Nouh Saad Mohamed, Yahaya Mohammed, Yugal Kishore Mohanta, Ali H Mokdad, Himel Mondal, Paula Moraga, Rafael Silveira Moreira, Rohith Motappa, Amin Mousavi Khaneghah, Ahmed Msherghi, Christopher J L Murray, Satta Muthu Murugan, David Musoke, Sherzad Ibrahim Mustafa, Saravanan Muthupandian, Kiran P. P Nagdeo, Lekshmi M Nair, Prasad Nalabothu, Rizwan Naqvi, Shumaila Nargus, Gustavo G Nascimento, Mohammad Zakaria Nassani, Zuhair S Natto, Biswa Prakash Nayak, Kinjal Nayak, Javad Nazari, Sudan Prasad Neupane, Sumaiya Nezam, Cuong Tat Nguyen, Duy Cao Nguyen, Tham Thi Nguyen, Vikram Niranjan, Lawrence Achilles Nnyanzi, Mohammed Noushad, Bogdan Oancea, Kanyin Liane Ong, Esteban Ortiz-Prado, Mahesh P A, Alicia Padron-Monedero, Jagadish Rao Padubidri, Sujogya Kumar Panda, Keerthi Panneer Selvam, Shahina Pardhan, Romil R Parikh, Sun Jae Park, Eleonora Pascucci, Jay Patel, Mehali Lalitbhai Patel, Mitesh Patel, Shivani Patel, Vipin Patidar, Shankargouda Patil, Shrikant Pawar, Olumuyiwa James Peter, Pawel Piotr Pietkiewicz, Thantrira Porntavetus, Amit Porwal, Priyanka Porwal, Sergio I Prada, Harsh Priya, Pooja Puri, Bharathi M Purohit, Asma Saleem Qazi, Xiang Qi, Nuzul Qur'aniati, Raghu Anekal Radhakrishnan, Venkatraman Radhakrishnan, Fakher Rahim, Amina Suleiman Rajah, Rafał Rakoczy, Venkitachalam Ramanarayanan, Shakthi Kumaran Ramasamy, Lokesh Rana, Kumuda Rao, Mithun Rao, Sowmya J Rao, Shree Rath, Devarajan Rathish, Tapash Ranjan Rautray, Sujith Sri Surya Ravi, Salman Rawaf, Muhammad Liaquat Raza, Elrashdy M Redwan, Abdul Rehman, Bhagwan Narayan Rekadwad, Abanoub Riad, Hermano Alexandre Lima Rocha, Chanapong Rojanaworarit, Mario Romandini, Rohini Ruhil, Chandan S N, Khaled Saad, Korosh Saber, Umar Saeed, Fatemeh Saheb Sharif-Askari, Amirhossein Sahebkar, Vaibhav Sahni, Alaka Sahoo, Ambika Sahoo, Durgesh Prasad Sahoo, Joseph W Sakshaug, Luciane B Salaroli, Samreen Saleem, Firdaus Samad, Fasineh Shekuba Samura, Abdallah M Samy, Sathish Sankar, Shilpa Sarawagi, Gargi Sachin Sarode, Sachin C Sarode, Michele Sassano, Aanchal Satija, Maheswar Satpathy, Amar Saurabh, Benedikt Michael Schaarschmidt, Falk Schwendicke, Siddharthan Selvaraj, Subramanian Senthilkumaran, Arsalan Shahri, Muhammad Shahzad, Muhammad Aaqib Shamim, Alfiya Shamsutdinova, Mohammed Shannawaz, Nadim Sharif, Amin Sharifan, Kamlesh Sharma, Bereket Beyene Shashamo, Mahabalesh Shetty, Premalatha K Shetty, Nzooma Munkwangu Shimaponda-Mataa, Shirindokht Shirazi, Km Shivangi, Deborah Oluwaseun Shomuyiwa, Sinegugu Nosipho Shongwe, Seyed Afshin Shorofi, Sunil Shrestha, Suleiman Adeiza Shuaibu, Gustavo Correia Basto Da Silva, Luís Manuel Lopes Rodrigues Silva, Abhinav Singh, Jasvinder A Singh, Prince Kumar Singh, Ragini D Singh, Samer Singh, Siddharth Singh, Surjit Singh, Gowri Sivaramakrishnan, Farrukh Sobia, Ranjan Solanki, Saraswathy Sreeram, Kannan Sridharan, Quirin D Strotzer, Mahwish Suhaib, Marjia Sultana, Jyothi Tadakamadla, Santosh Kumar Tadakamadla, Iman M Talaat, Mircea Tampa, Benjamin Kye Jyn Tan, Abdulkarim Temsah, Reem Temsah, Dufera Rikitu Terefa, Azimeraw Arega Tesfu, Ismaeel Tharwat, Manuel Sebastian Thomas, Arole Darwin Touko, Marcos Roberto Tovani-Palone, Thang Huu Tran, Indang Trihandini, Mike Tuffour Amirikah, Srikanth Umakanthan, Bhaskaran Unnikrishnan, Amedin Aliyi Usso, Olivier Uwishema, Hande Uzunçıbuk, Orsolya Varga, Siddhartha Alluri Varma, Vishnu Priya Veeraraghavan, Prabhakar Veginadu, Nguyen-Kieu Viet-Nhi, Vignesh Vikram, Victor E Villalobos-Daniel, Simona Ruxandra Volovat, Rashim Wadhwa, Liang Wang, Wanzhou Wang, Tati Suryati Warouw, Desalegn Desalegn Weldebrhan, Nuwan Darshana Wickramasinghe, Andrew Awuah Wireko, Dawit Habte Woldeyes, Patlolla Sriram Yadav, Saba Yahoo, Kivanc Yangi, Thomas Kidanemariam Yewodiaw, Siyan Yi, Dong Keon Yon, Monal Yuwanati, Vesna Zadnik, Mubashir Zafar, Muhammad Sohail Zafar, Iman Zare, Mohammed G M Zeariya, Zhi-Jiang Zhang, Jianhui Zhao, Hafsa Zia, Zahraa Zibara, Magdalena Zielińska, Shaher H Zyoud

Managing the estimation or publications process

Eduardo Bernabe, Simon I Hay, Christopher J L Murray
